# QTL mapping for flowering-time and photoperiod insensitivity of cotton *Gossypium darwinii* Watt

**DOI:** 10.1371/journal.pone.0186240

**Published:** 2017-10-09

**Authors:** Fakhriddin N. Kushanov, Zabardast T. Buriev, Shukhrat E. Shermatov, Ozod S. Turaev, Tokhir M. Norov, Alan E. Pepper, Sukumar Saha, Mauricio Ulloa, John Z. Yu, Johnie N. Jenkins, Abdusattor Abdukarimov, Ibrokhim Y. Abdurakhmonov

**Affiliations:** 1 Laboratory of Structural and Functional Genomics, Center of Genomics and Bioinformatics, Academy of Sciences of the Republic of Uzbekistan, Tashkent, Uzbekistan; 2 Department of Biology, Texas A&M University, Colleges Station, Texas, United States of America; 3 Crop Science Research Laboratory, United States Department of Agriculture-Agricultural Research Services, Starkville, Mississippi, United States of America; 4 Plant Stress and Germplasm Development Research, United States Department of Agriculture-Agricultural Research Services, Lubbock, Texas, United States of America; 5 Southern Plains Agricultural Research Center, United States Department of Agriculture-Agricultural Research Services, College Station, Texas, United States of America; Dokuz Eylul Universitesi, TURKEY

## Abstract

Most wild and semi-wild species of the genus *Gossypium* are exhibit photoperiod-sensitive flowering. The wild germplasm cotton is a valuable source of genes for genetic improvement of modern cotton cultivars. A bi-parental cotton population segregating for photoperiodic flowering was developed by crossing a photoperiod insensitive irradiation mutant line with its pre-mutagenesis photoperiodic wild-type *G*. *darwinii* Watt genotype. Individuals from the F_2_ and F_3_ generations were grown with their parental lines and F_1_ hybrid progeny in the long day and short night summer condition (natural day-length) of Uzbekistan to evaluate photoperiod sensitivity, i.e., flowering-time during the seasons 2008–2009. Through genotyping the individuals of this bi-parental population segregating for flowering-time, linkage maps were constructed using 212 simple-sequence repeat (SSR) and three cleaved amplified polymorphic sequence (CAPS) markers. Six QTLs directly associated with flowering-time and photoperiodic flowering were discovered in the F_2_ population, whereas eight QTLs were identified in the F_3_ population. Two QTLs controlling photoperiodic flowering and duration of flowering were common in both populations. *In silico* annotations of the flanking DNA sequences of mapped SSRs from sequenced cotton (*G*. *hirsutum* L.) genome database has identified several potential ‘candidate’ genes that are known to be associated with regulation of flowering characteristics of plants. The outcome of this research will expand our understanding of the genetic and molecular mechanisms of photoperiodic flowering. Identified markers should be useful for marker-assisted selection in cotton breeding to improve early flowering characteristics.

## Introduction

One of the most important events in the life cycle of plants is the transition from vegetative to reproductive growth [1]. Floral initiation in plants depends on endogenous (internal) and exogenous (external) factors. External factors that induce the transition to flowering include temperature and photoperiodism [2]. The most important factor that influences this transition is photoperiodism, i.e., the relative duration of light and dark of a day [3]. The mechanisms of photoperiodic flowering have been extensively studied in the model plant *Arabidopsis thaliana* [3, 4]. The transition to flowering is largely controlled through the integration of four major signaling pathways: vernalization, photoperiod, autonomous flowering, and gibberellic acid [5]. The optimal time of flowering tailors the vegetative and reproductive growth for the optimal accumulation and allocation of resources to seed production in plants. This is one of the most important factors determining plant productivity and adaptation to regional environments of different latitudes [6]. Perception of photoperiodic conditions is mediated by the phytochrome and cryptochrome photoreceptor systems [7–11].

Plants are divided into three main groups according to photoperiodic response for flowering: 1) long-day plants—flower when day length increases to reach a critical photoperiod 2) short-day plants—flower when day lengths become less than their critical photoperiods; and 3) day-neutral plants—flower almost simultaneously at any day length and they are considered as photoperiod insensitive plants [12]. Most of wild relatives of crop species, including many wild species and pre-domesticated landrace stocks of *Gossypium* genus, are very photoperiodic plants [12].

Cotton (*Gossypium* genus) is a very important crop for the economy of cotton-growing countries. One of the aims of breeding programs of all cotton-growing countries worldwide is to create new cotton cultivars resistant to various biotic and abiotic factors. Another key aim is to improve technical and economic characteristics such as fiber quality [13–18]. Collections of wild cotton germplasm are a valuable source of genes for the genetic improvement of cotton [19–21]. For example, according to Fryxell [22], a truly wild species of cotton *Gossypium darwinii* Watt (allotetraploid chromosome set of 52, representing the [AD]_5_ genome), is endemic to the Galapagos Islands, and is a close relative to *G*. *barbadense* (also sometimes referred to as *G*. *barbadense* ssp. *darwinii*) [23]. It has genes that confer tolerance to drought, nematode resistance and high fiber quality [24]. In addition, *G*. *darwinii* has the potential to grow in soils with high salinity [25]. According to the classification of cotton germplasm resources, *G*. *darwinii* and *G*. *hirsutum* L. (Upland) cotton belong to the primary germplasm pool and can easily hybridize with each other [24]. Traits of wild *G*. *darwinii* species can be introgressed into Upland cotton germplasm to expand their genetic base. However, as mentioned above, many species of cotton including *G*. *darwinii* are short-day photoperiod-sensitive plants. This creates biological and technical challenges for their use in breeding programs [26]. One of the solutions is a detailed study of genes/loci involved in the mechanism of photoperiodic flowering of cotton, followed by manipulation of expression of the target genes. Such knowledge will be helpful to produce novel cultivars with high yield that are well adapted to diverse growing environments.

Microsatellite or simple sequence repeat (SSR) markers have been successfully used in assessing the genetic diversity of cotton cultivars/accessions, as well as in tagging useful loci using linkage disequilibrium (LD) [20, 27, 28] and QTL-mapping in cotton [29–31]. During the past two decades, some cotton flowering and/or maturity-related QTL mapping reports using SSR markers were published. For instance, cotton geneticists have focused on identifying flowering-related genes using EST-SSRs and found 34 candidate genes that are involved in the process of flowering determination, floral meristem identity, and floral organ development in *G*. *hirsutum* [32]. Researchers presented the tagging of cotton flowering-time QTL, assessed by node of first fruiting branches [33]. Li et al. [34] identified 54 QTLs of early-maturing traits of cotton using 4,083 SSR markers. Furthermore, in a parental cross of the photoperiod-sensitive (NC7018) and photoperiod-insensitive (Pima S-7) lines of *G*. *barbadense*, using SSR markers, Zhu and Kuraparthy [35] succeeded to localize the photoperiod responsive locus *Gb_Ppd1* and several tightly linked SSR markers on cotton chromosome 25. *In silico* analysis of genomic regions of *Gb_Ppd1* using *G*. *raimondii* genome sequence further identified the putative gene *Gorai*.*010G161200* that supported the importance of SSRs markers mapped in this region for molecular breeding of photoperiodic flowering in cotton [35].

However, despite the economic importance of cultivated species of *Gossypium* genus, genes controlling photoperiodic flowering are not sufficiently studied in this crop [35]. Although several researchers have studied photoperiodic response of cotton using different classical methods such as heterosis, hybridization and mutagenesis, molecular mapping of QTL loci regulating cotton flowering-time is limited.

In this context, several photoperiod insensitive cotton mutant lines have been produced by converting wild cottons directly into day-neutral forms using irradiation mutagenesis at the Institute of Genetics and Plant Experimental Biology (IGPEB) Academy of Science of Uzbekistan [26]. In our previous study, examining phylogenetic deviations of these mutant lines from their original forms, forty SSR loci were identified, which are potentially useful in assessing photoperiodic flowering characteristics of cotton [26]. Among the studied mutant genotypes, particularly, one irradiation induced mutant line of *G*. *barbadense* ssp. darwinii [26] was converted from a photoperiod sensitive plant into a day neutral plant. Several other phenotypic trait changes were also apparent in the mutant line. These were not unexpected since the line was developed following a gamma irradiation [26]. The objective of the current study is to map loci linked to day-neutral flowering in an irradiation mutant *G*. *darwinii* line. Toward this goal, genetic (F_2_ and F_3_) populations were created by crossing a wild-type *pre-mutagenesis G*. *darwinii* (photoperiod sensitive) and its irradiation mutant line (insensitive to photoperiod). Further, both populations were subjected to QTL-mapping using 212 SSR and three CAPS markers. Here several QTLs associated with photoperiodic flowering, as well as with other morphological traits, in F_2-3_ populations are reported. *In silico* bioinformatics analyses of mapped marker regions using sequenced cotton genomes identified a number of candidate genes potentially involved in the regulation of photoperiodic flowering. These loci could be used in marker-assisted selection to select for day neutrality in the introgression of alleles from photoperiodic germplasm.

## Materials and methods

### Mapping populations and phenotypic data collection

This study involved individuals of F_2_ and F_3_ populations, which were developed from the cross between wild-type (pre-mutagenesis) *G*. *barbadense* ssp. darwinii (further referred to as *G*. *darwinii)* and its direct γ-irradiation mutant line [26]. Parental genotypes, selected from the cotton germplasm collection at the Institute of Genetics and Plant Experimental Biology (IG&PEB, Uzbekistan), significantly differed in photoperiod sensitivity. The wild type is highly sensitive to photoperiod while the mutant genotype, derived from wild type *G*. *darwinii* using γ-irradiation, is a day-neutral line i.e., not sensitive to photoperiod [26].

**Fig 1:**
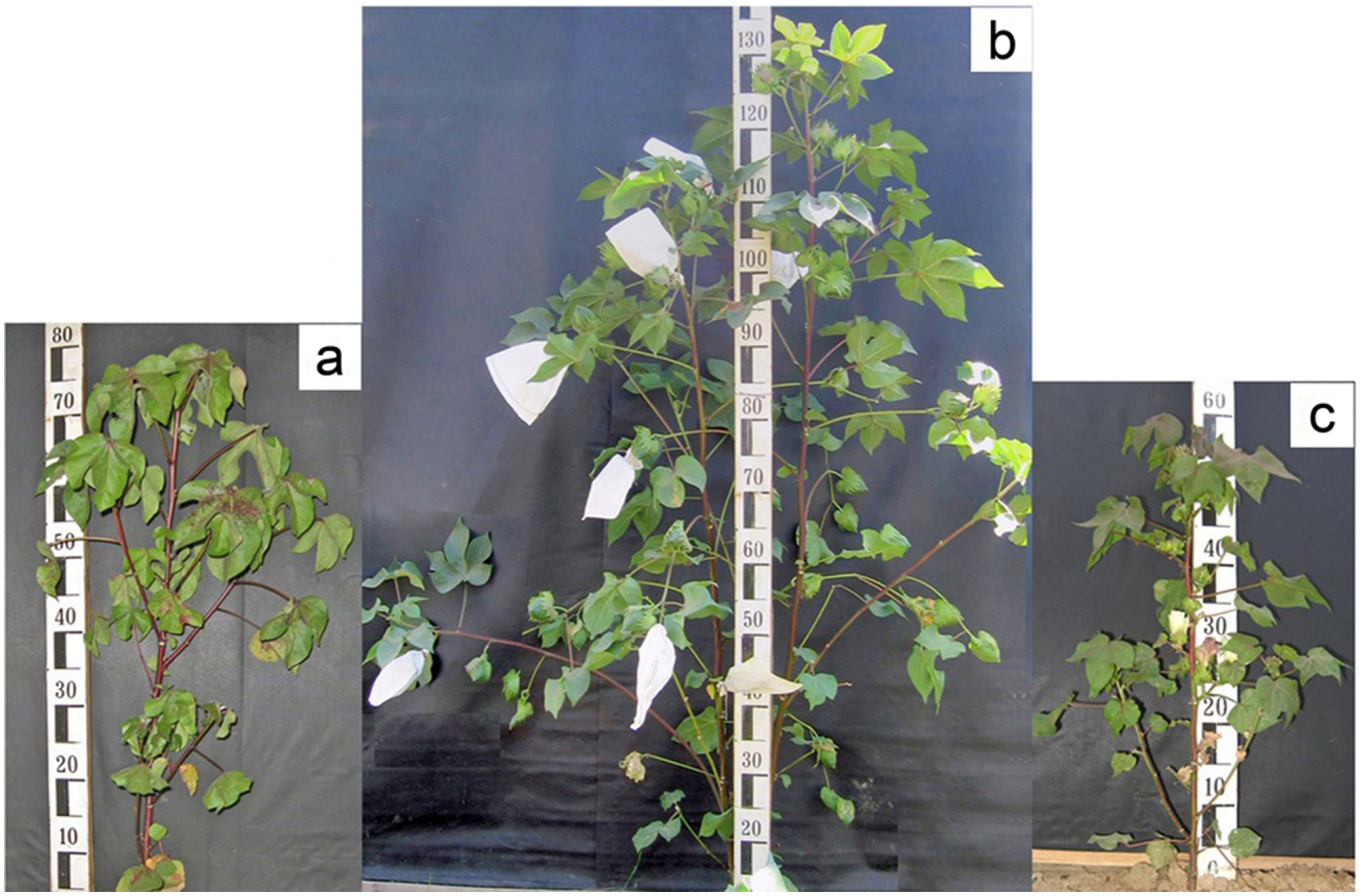
The cross-combination between the wild type of cotton species *G*. *darwinii* Watt with its photoperiod insensitive irradiation mutant line. A) Wild type, B) F_1_ plant, and C) irradiation mutant line.

In 2006, parental samples were grown under two different photoperiod conditions: a photoperiod insensitive irradiation mutant line was grown under conditions of natural day length, and a wild form was forced to flower in artificially created environment 8-hour light, 16-hour dark. Pollen of wild photoperiodic *G*. *darwinii* was used to pollinate its irradiation mutant mutant line. In 2007, the F_1_ hybrids were grown in the nursery at the Institute of Genetics and Plant Experimental Biology, Tashkent, Uzbekistan (long day conditions) and self-pollinated to generate the F_2-3_ populations (Fig 1).

A total of 129 F_2_ individuals, five plants from each parental genotypes and their F1 hybrid were sown late in April 2008 under a natural day length, with a planting density of 60 cm × 20 cm. To study photoperiod sensitivity, all F_2_ individuals were phenotypically evaluated for flowering-time (FT), photoperiodic flowering (PhFl) and flowering duration (FDR). To determine FT, the date of first flower (or matured bud a day before of flowering) emergence was recorded for each individual plants. Further, the date of flowering of individual plants was recorded during a window of 10 days from July 1 to September 24 of the season that detected start of flowering according to shortening of day length (i.e., photoperiodic flowering (PhFl) relative to long and subsequent short day conditions). Additionally, the flowering duration (FDR) from July until the end of vegetation season was also studied that characterized continuity of flowering in mapping populations. FT, PhFl and FDR are somewhat overlapping traits, but allow us to evaluate different aspects of plant flowering in relation to day length.

Moreover, the traits indirectly related to flowering-time and photoperiod sensitivity in cotton, such as node of first fruiting branch (NFB), number of buds (NBD), monopodial (NMB) and sympodial branches (NSB), number of nodes (NND), number of bolls (NBL) number of and opened bolls (NOBL) and plant height (PH) were measured in ontogenesis. In addition, two morphological traits such as the anthocyanin (SA) and hairiness of stem (SH) were studied in this experimental population. In late summer, the F_3_ family seeds were collected from those F_2_ plants that had bloomed and produced the seeds during the growing season. In 2009, the individuals in the F_3_ generation were investigated for only the photoperiod sensitivity trait such as FT, PhFI and FDR. All individuals in both populations were genotyped and used for mapping photoperiodic control of flowering-time and other morphological traits. The experimental area (Qibray district of Tashkent region) lies at 40.1166700 latitude and 64.5666700 longitudes. During the 2008-growing season of the F_2_ population, an average day length (h:m:s) was 15:06:26 in June; 14:47:54 in July; 13:46:01 in August; and 12:26:47 in September. An average day length (h:m:s) in 2009-growing season of the F_3_ population was, 15:06:22 in June; 14:48:15 in July; 13:46:36 in August; and 12:27:27 in September. The average temperature from April to September was 26°C in 2008 versus 24°C in 2009. It was colder spring in 2009 (average of 17°C) compared to 2008 (average of 21°C) growing season.

Estimates of broad-sense heritability (*H*^*2*^) for each trait in both populations were calculated according to Singh *et al*. [36] using the following formula:

$$H^{2}=V_{g}/V_{p},$$

where *V*_*g*_—the genetic variance and *V*_*p*_—phenotypic variance.

The phenotypic variance (*Vp*) and variance of geneotype (*V*_*g*_) were found by the following formulas *V*_*p*_ = *V*_*g*_*+V*_*e*_ and *V*_*g*_ = *V*_*p*_*-V*_*e*_, here *V*_*e*_ is the environmental variance. The environmental variance (*V*_*e*_) was estimated using the formula *V*_*e*_ = (*V*_*P1*_ + *V*_*P2*_)/2, where *V*_*P1*_ and *V*_*P2*_ were the variances of parent genotypes.

### DNA extraction and genotyping

Genomic DNAs were isolated from young leaves using the cetyltrimethylammonium bromide (CTAB) method [37], with minor modifications. In a previous study, 40 polymorphic SSR markers were identified between cotton photoperiod insensitive mutants and their wild types [26]. In this study, a set of 1,060 SSR primer pairs from the seven SSR marker collections (https://www.cottongen.org/sites/default/files/cottongen_download_site/markers/) including BNL, CIR, CM, GH, JESPR, TMB, and NAU was used to identify additional polymorphic markers and tagging targeted QTLs. Additionally, new phytochrome specific *PHYA1*, *PHYB* and *PHYB2* CAPS markers [38] that were polymorphic between parent lines were also used for mapping.

The PCR amplifications were performed in a 10 μl reaction mixture containing 1 μl 10 x PCR buffer with MgCl_2_, 0.5 μl 25 mM of a dATP, dGTP, dTTP, and dCTP mix, 0.5 μl 25 ng/ml of each reverse and forward primer, 1 μl 25 ng/ml template DNA and 0.1 unit Taq DNA polymerase. PCR amplification was performed on a GeneAmp 9700 thermal cycler using a program consisting of an initial denaturation at 94°C for 5 min, 40 cycles of: denaturation at 95°C for 45 sec., annealing at 55–68°C (depending on primers) for 45 sec. and elongation at 72°C for 2 min., and finishing with a final elongation at 72°C for 10 min. In the CAPS marker assays, PCR products were purified using a 26% polyethylene glycol (PEG) solution (PEG 8000, 6.5 mM MgCl2, 0.6 M NaOAc—pH 6.0–7.0) and digested with commercial restriction enzymes. Restriction analysis of each sample was performed in 10 μl of reaction mixture containing 1 μl 10 × restriction enzyme buffer, 2 μl purified PCR product, 0.2 Unit restriction enzyme and 6.5 μl sterile water. The digested products were electrophoresed on 3.5% high-resolution agarose (HiRes Agarose) gel in 0.5 × TBE buffer, with a mode voltage of 5.3 V/cm. After electrophoresis, gels were stained with ethidium bromide (EtBr) solution for 5–10 min and photo-documented using Gel Imaging Documentation System (Alphaimager 2200, Alpha Innotech, USA) with exposure under the UV light.

### Linkage map construction

Genetic linkage maps were constructed from the genotypic data of polymorphic SSR and CAPs marker loci in both populations using the program JoinMap version 3.0 [39]. Assignment of linkage groups to the respective chromosomes was accomplished using data from CottonGen (https://www.cottongen.org/data/download/map) based on previously published molecular maps (S1 and S2 Data). For a graphical representation of QTL maps and linkage groups the program MapCHART version 2.2 was used [40]. Regression analysis was used to identify associations between genotypic data and phenotypic traits (S3 and S4 Data). Thus, two separate QTL-mapping analyses in F_2_ and F_3_ populations were performed using QTL Cartographer v2.5 [41] using Kosambi [42] as a mapping function, applying the algorithm of composite interval mapping (CIM). The average critical threshold LOD levels were identified by QTL Cartographer and Qgene [43] at the 95% significance level after 1000 times permutation test.

### *In silico* PCR annotation

Based on the assumption of significant gene colinearity between *G*. *darwinii* and closely related *G*. *hirsutum* (ref on phylogenetic similarity), *in silico* analysis of genomic regions identified by QTL were performed using whole genome sequences of *G*. *hirsutum* L. [44]. This was for estimation of actual physical genomic positions of loci, and for prediction of linked candidate genes/protein potentially involved in the mechanism of photoperiodic flowering. *In silico* PCR was carried out using the UGENE 1.20.0 [45] bioinformatics software package to find the locations of 'virtual amplicons' on the cotton genome using SSR primer pairs that were associated to the mapped QTL(s). A web-based gene prediction application, AUGUSTUS 3.1.0 [46], was used for identifying the genes on these chromosomes. Predicted amino acid sequences by AUGUSTUS were analyzed using Basic Local Alignment Search Tool search algorithm (www.ncbi.nlm.nih.gov/BLAST).

## Results and discussion

### Phenotypic trait analysis

**Fig 2:**
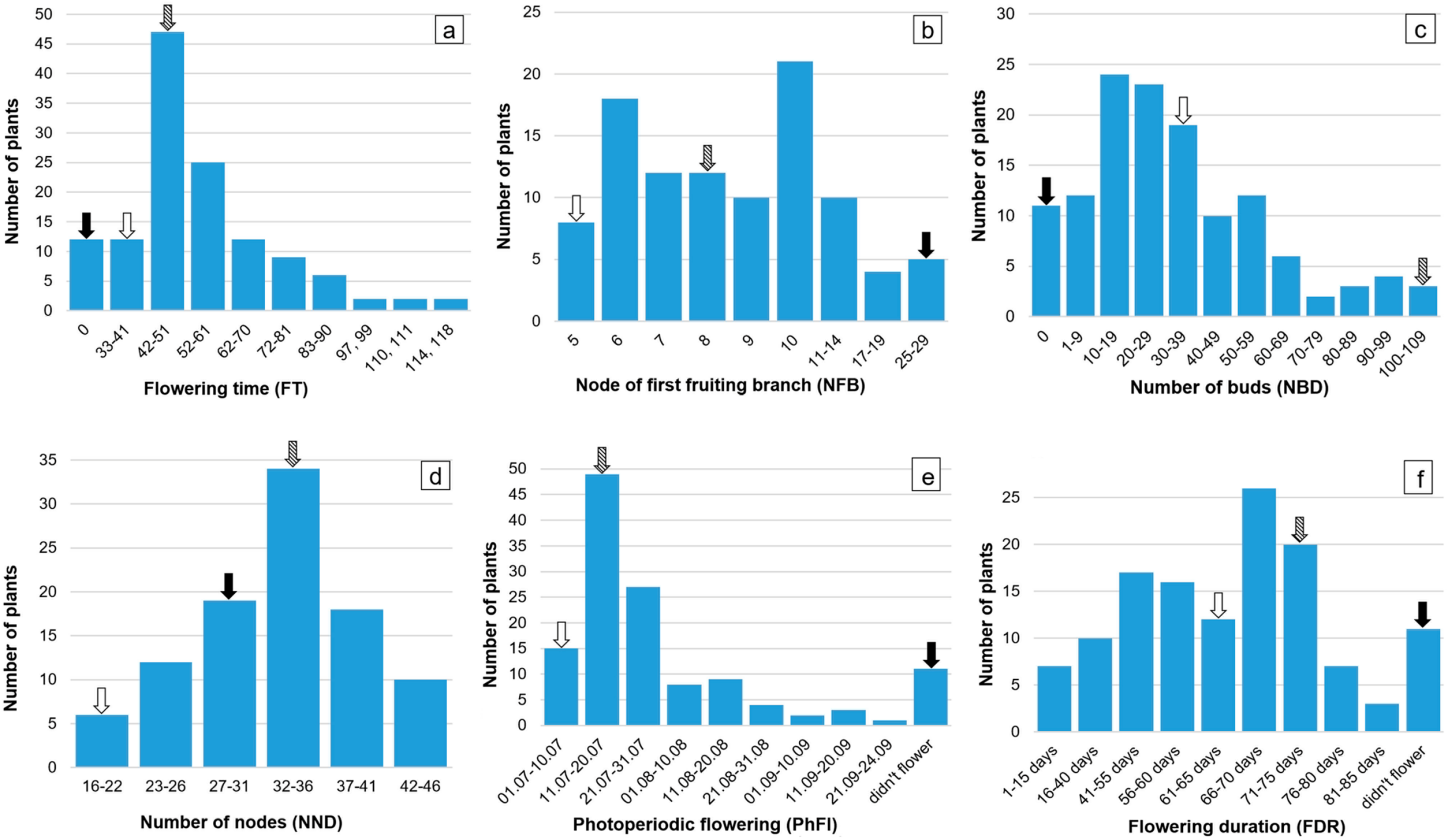
Histogram of all recorded traits of flowering-time, photoperiodic flowering, other related to flowering and some morphological traits. a) flowering-time, b) node of first fruiting branch, c) number of buds, d) number of nodes, e) photoperiodic flowering, f) flowering duration Arrows show means for parental genotypes and F_1_ hybrid; black arrow—wild type, white arrow—irradiation mutant, and arrow with patterned fill—F_1_ plant.

The flowering-time QTL for related to photoperiod sensitivity were mapped in both F_2_ and F_3_ populations. The phenotypic observations in the F_2_ population revealed that 70.5% individuals were insensitive to photoperiod and began to bloom before August 1, 2008, 16.3% of plants began to bloom in the period from August 1 until September 1 2008, 4.7% of plants flowered after September 1, 2008 and 8.5% of plants were unable to flower and did not bloom under natural condition of day length suggesting the possibility that a single dominant mutation may be responsible for the change in photoperiod sensitivity. In addition, the node of first fruiting branch (NFB) was investigated because this trait is also considered as the indirect measures of flowering-time. In the F_2_ population individuals, NFB was varied widely, from 5 (irradiation mutant parent) to 29 (pre-mutagenesis wild-type parent), with the majority falling within 5 to 10 node category (Fig 2), which correlates to flowering characteristics mentioned above. In addition, in the F_2_ population individuals, the morphological traits of monopodial and sympodial branches, number of nodes, number of opened bolls, anthocyanin and hairiness of stem were examined (S1 Fig).

Estimates for broad-sense heritability (*H*^*2*^) for all traits ranged from 0.18 to 0.93% (Table 1). Almost all the traits related to flowering-time (flowering time, photoperiodic flowering and flowering duration) had high heritability (0.89–0.93). In order to study the inheritance of flowering-related traits, only four traits, flowering-time, duration of flowering, photoperiodic flowering and number of buds were evaluated until the end of August in the F_3_ population. The phenotypic observation in the F_3_ generation showed that, 57.8% of individuals began to bloom before August 1, 2009, 39.2% of hybrids began to bloom in the period from August 1 until September 1 2009, and 3% of individuals turned out to be strongly photoperiod sensitive and did not bloom till end of the season.

**Table 1:** Genetic variances and estimated broad-sense heritability of photoperiod related traits in the F_2_ population.

	FT	NFB	NBD	NND	PhFI	FDR
**P**_**1**_	0.00	2.70	0.00	3.35	0.00	0.00
**P**_**2**_	3.40	0.69	7.85	2.27	0.49	5.20
**F**_**2**_	23.13	4.97	26.54	6.59	2.55	24.37
**V**_**g**_	21.43	1.92	22.62	2.11	2.31	21.77
**H**^**2**^	0.93	0.39	0.85	0.32	0.90	0.89

FT—flowering-time; NFB—node of first fruiting branch; NBD—number of buds; NND—number of nodes; PhFl—photoperiodic flowering; FDR—flowering duration; P_1_—wild type pre-mutagenesis parent (*G*. *drawinii*); P_2_—irradiation mutant parent (*G*. *drawinii)*; F_2_—second generation population; V_g_—genotypic effect; and H^2^—broad-sense heritability

These observations are consistent with the segregation of single dominant locus in these population individuals. The difference observed in percentages for plants flowered before and after August 1 in F_2_ and F_3_ population individuals should be associated with differences in 2008 and 2009 growing environments because, as mentioned above, a comparatively colder weather was observed in the spring of 2009 than 2008. The broad-sense heritability estimates of the studied traits in F_3_ generation are presented in Table 2. This shows that one major dominant gene is probably involved in the control of photoperiod insensitivity in the irradiation *G*. *drawinii* line. In contrast, Zhu and Kuraparthy [35] reported that photoperiod-sensitivity trait was segregated as 3:1 ratio in the genetic cross of the photoperiod-sensitive (NC7018) and photoperiod-insensitive (Pima S-7) lines of *G*. *barbadense*, suggesting photoperiod sensitivity in NC7018 could be controlled by a single dominant gene located in chromosome 25.

**Table 2:** Genetic variances and estimated broad-sense heritability of traits in the F_3_ population.

	Number of buds (NBD)	Photoperiodic flowering (PhFl)	Flowering time (FT)	Flowering duration (FDR)
**P**_**1**_	0.00	0.00	0.00	0.00
**P**_**2**_	7.85	0.49	3.40	5.20
**F**_**3**_	14.24	26	11.24	12.51
**V**_**g**_	10.32	25.76	9.54	9.91
**H**^**2**^	0.72	0.99	0.85	0.79

P_1_—wild type pre-mutagenesis parent (*G*. *drawinii*); P_2_—irradiation mutant parent (*G*. *drawinii)*; F_3_—third generation population; V_g_—genotypic effect; and H^2^—broad-sense heritability

This comparison may suggest that irradiation mutant loci mapped from mutant *G*.*darwinii* genome is different from photoperiod-sensitive exotic lines of cotton, which requires further studies.

### Linkage maps

**Fig 3:**
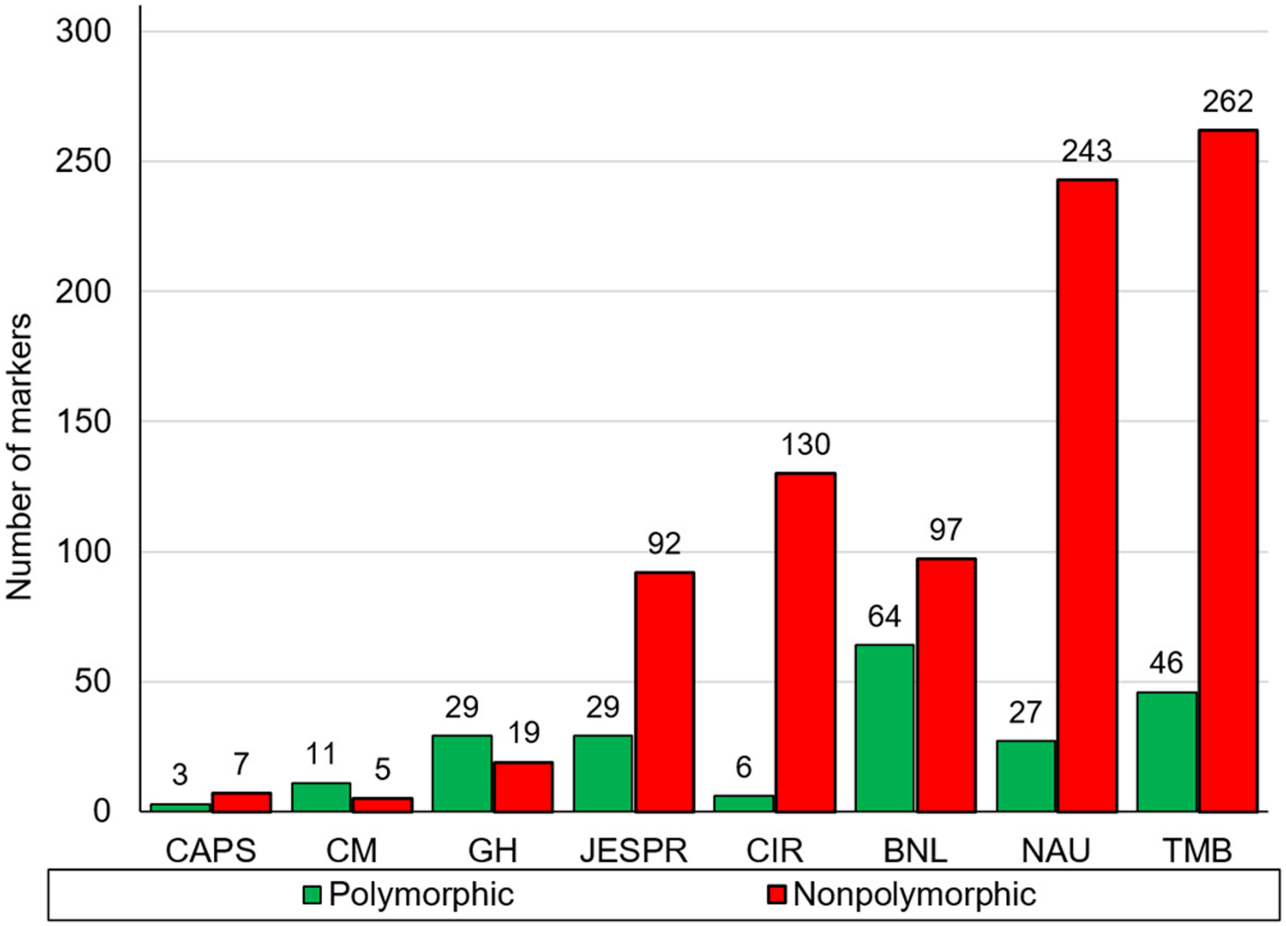
The level of polymorphism of all SSR markers between parental genotypes.

**Fig 4:**
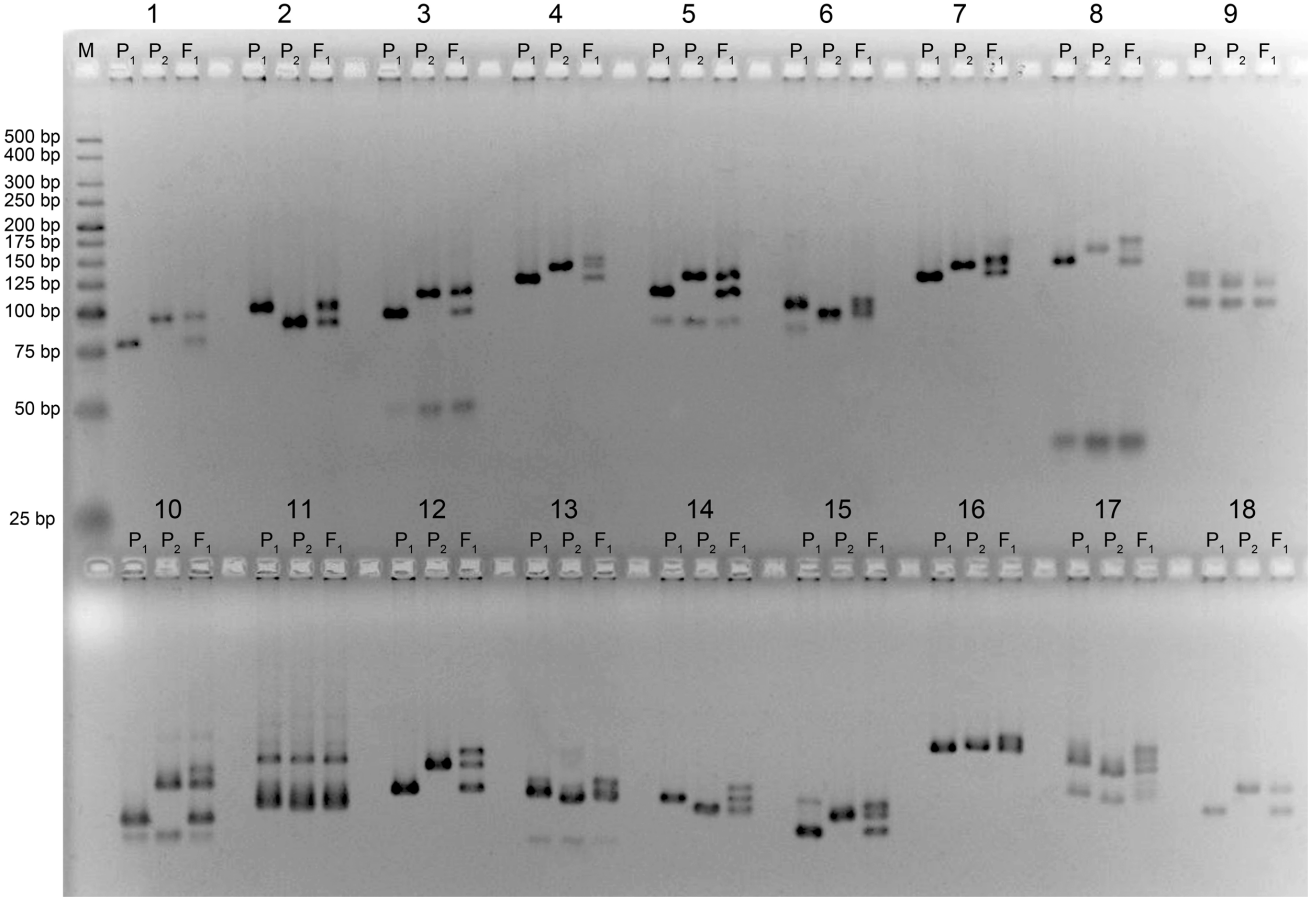
Polymorphic GhSSR markers between parental genotypes. (M)—Molecular-weight size marker, ‘P_1_’ and ‘P_2_’—parent genotypes, F_1_—first-generation hybrid, GhSSRs; 1-GH2, 2-GH27, 3-GH32, 4-GH34, 5-GH39, 6-GH48, 7-GH52, 8-GH54, 9-GH56, 10-GH75, 11-GH77, 12-GH82, 13-GH83, 14-GH98, 15-GH109, 16-GH110, 17-GH112, 18-GH118.

**Fig 5:**
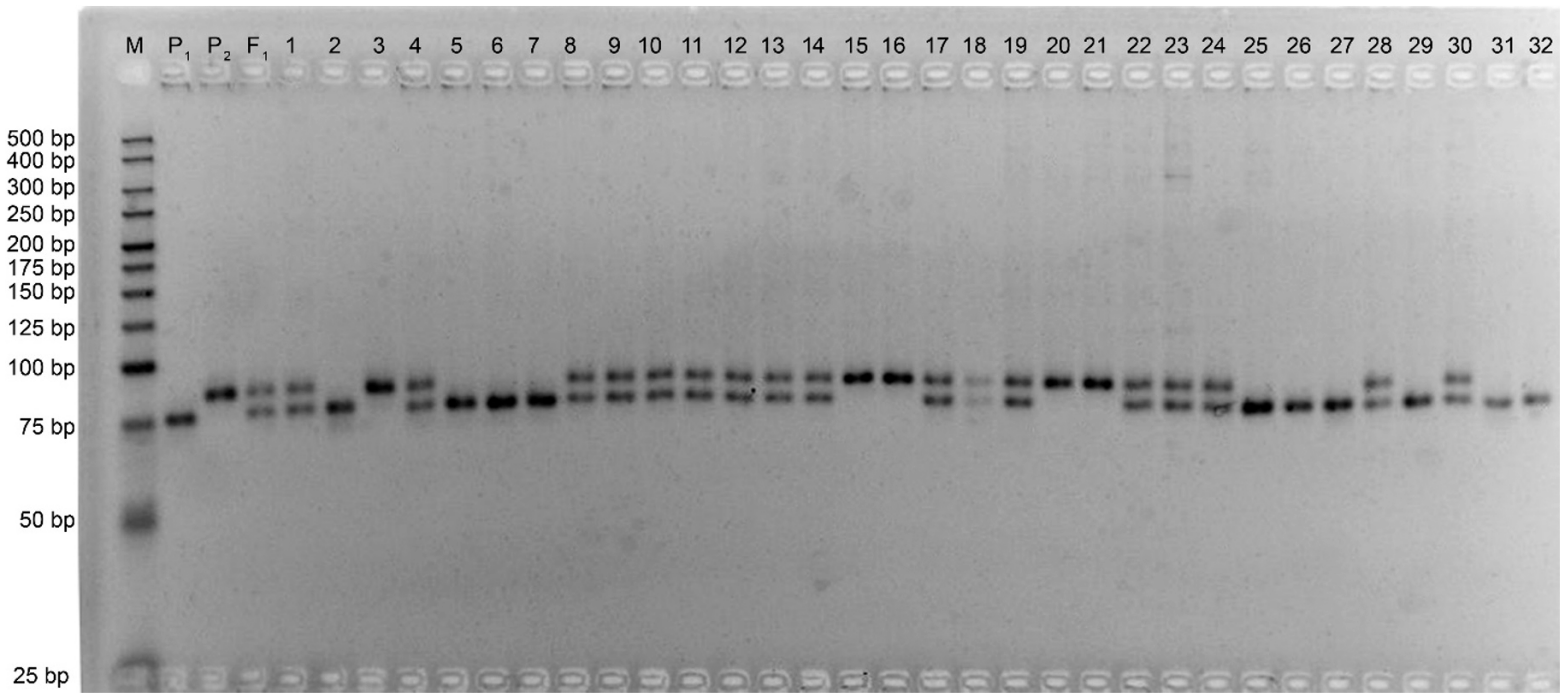
Examples of SSR markers (JESPR270), segregating in F_2_ population. (M)—Molecular-weight size marker, ‘P_1_’ and ‘P_2_’—parents, F_1_—first-generation hybrid, 1–32—individuals of the F_2_ generation.

Out of a total of 1,060 SSR and 10 CAPS markers screened, 212 SSRs (Figs 3 and 4) and three CAPS primer pairs detected polymorphism between the parental genotypes, representing a level of polymorphism of 20% and 30%, respectively. Analysis of polymorphism of parental lines showed that the set of CIR SSR were low in polymorphism, while GH SSR showed a high level of polymorphism relative to other microsatellite markers (Figs 3 and 4). These polymorphic markers were used to genotype the F_2_ (Fig 5) and F_3_ populations. Molecular genetic linkage maps were created that mapped 194 SSR markers out of the polymorphic set (Table 3) in the F_2_ and 158 markers in the F_3_ population.

**Table 3:** Information of mapped SSR and CAPS markers.

#	Types and collection of markers	in F_2_ population		in F_3_ population	
		Number of mapped markers	Number of unmapped markers	Number of mapped markers	Number of unmapped markers
1	BNL SSR	59	5	45	19
2	CIR SSR	4	2	3	3
3	CM SSR	11	0	11	0
4	GH SSR	25	4	18	11
5	JESPR SSR	26	3	23	6
6	NAU SSR	25	2	17	10
7	TMB SSR	41	5	38	8
8	CAPS	3	0	3	0
	**Total**	**194**	**21**	**158**	**57**

**Fig 6:**
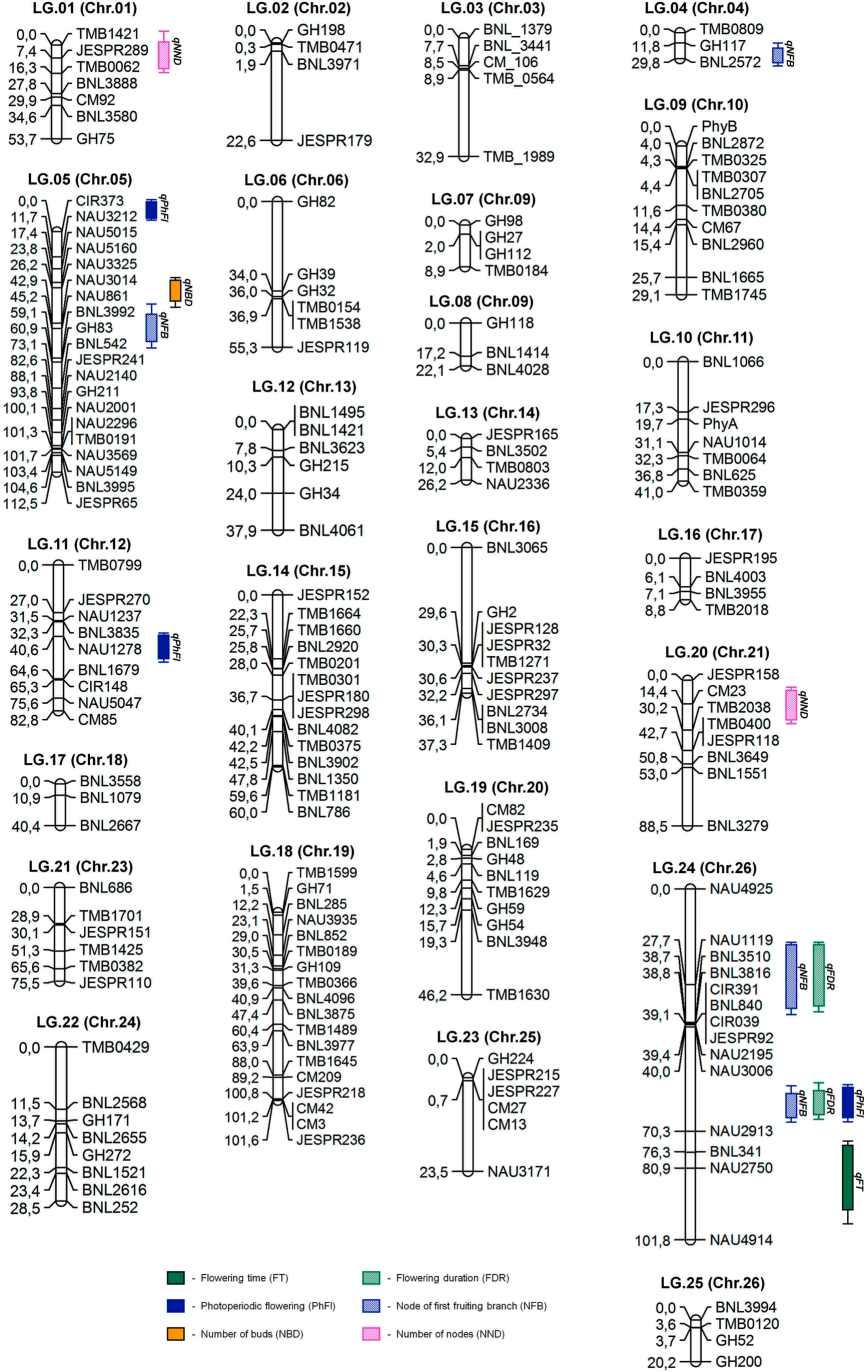
Genetic linkage map of the F_2_ population showing the location of QTL for photoperiodic flowering.

**Fig 7:**
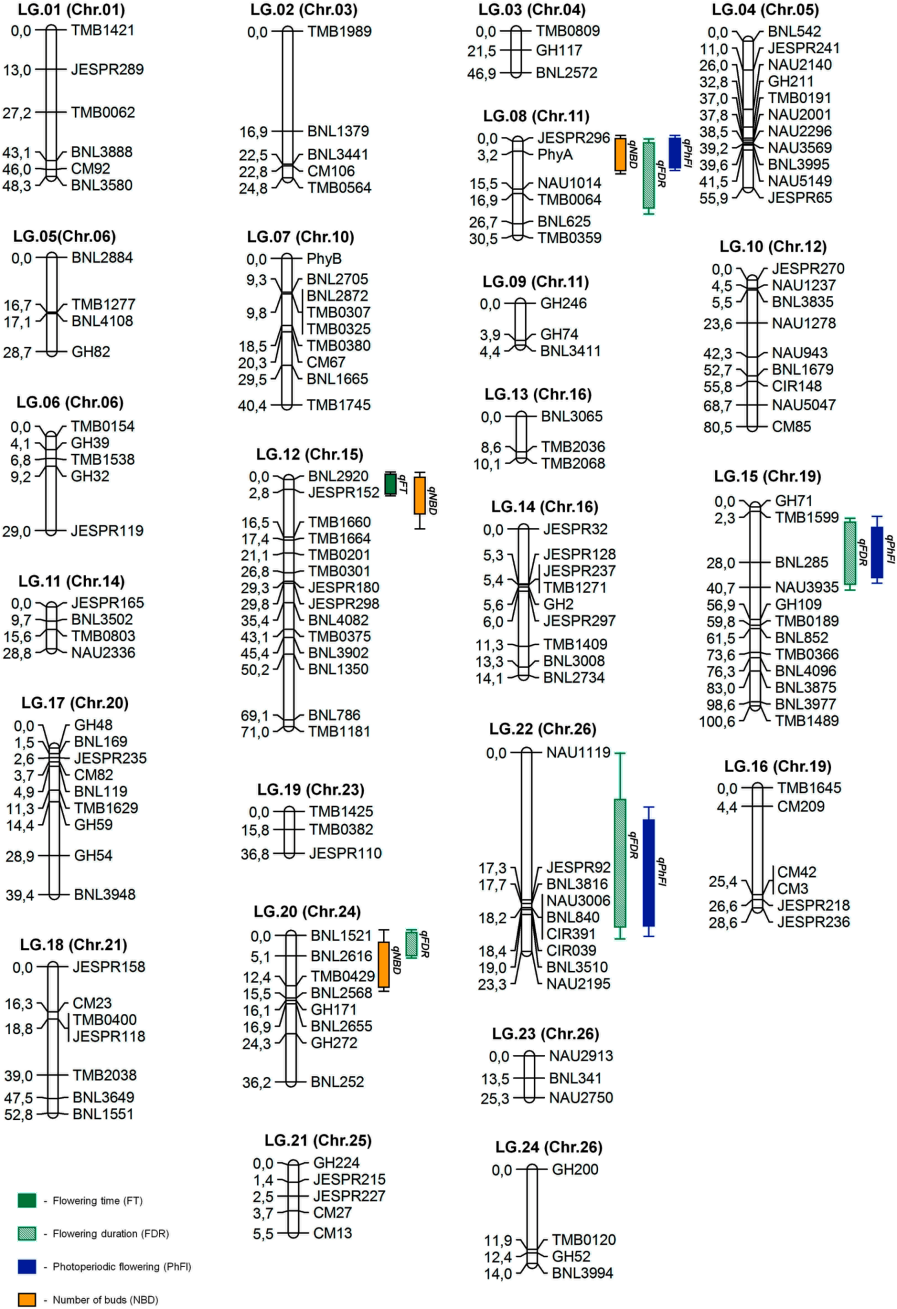
Genetic linkage map of the F_3_ population showing the location of QTL for photoperiodic flowering.

The remaining 18 and 54 SSRs were unmapped in respective F_2_ and F_3_ (Table 3). The linkage maps in F_2_ population formed 25 linkage groups (LGs; Table 4) covering a total of 1158.5 cM with an average distance of 5.97 cM between loci (Fig 6, Table 4), while F_3_ population linkage maps present 24 LGs (Table 5) covering 875.4 cM with an average distance of 5.54 cM between two markers (Fig 7). The F_2_ linkage map represents 23 cotton chromosomes and the linkage map in the F_3_ population represents 18 chromosomes. The chromosomal localization of 155 mapped SSRs and 3 CAPS markers were identified (data not shown). This result is supported with previous published reports [26, 30, 38].

**Table 4:** Linkage group (LG) / chromosome (Chr.) information in the F_2_ population.

#	Linkage groups	Number of mapped marker loci	Total map length (cM)	Mean map distance/Marker
1	LG01 (Chr.01)	7	53.6	7.66
2	LG02 (Chr.02)	4	22.6	5.65
3	LG03 (Chr.03)	5	32.8	6.56
4	LG04 (Chr.04)	3	29.8	9.93
5	LG05 (Chr.05)	20	112.6	5.63
6	LG06 (Chr.06)	6	55.3	9.22
7	LG07 (Chr.09a)	4	8.9	2.23
8	LG08 (Chr.09b)	3	22.1	7.37
9	LG09 (Chr.10)	11	29.1	2.65
10	LG10 (Chr.11)	7	41.0	5.86
11	LG11 (Chr.12)	9	82.8	9.20
12	LG12 (Chr.13)	6	37.9	6.32
13	LG13 (Chr.14)	4	26.2	6.55
14	LG14 (Chr.15)	14	60.0	4.29
15	LG15 (Chr.16)	10	37.3	3.73
16	LG16 (Chr.17)	4	8.8	2.20
17	LG17 (Chr.18)	3	40.4	13.47
18	LG18 (Chr.19)	18	101.6	5.64
19	LG19 (Chr.20)	10	46.2	4.62
20	LG20 (Chr.21)	8	88.5	11.06
21	LG21 (Chr.23)	6	75.5	12.58
22	LG22 (Chr.24)	8	28.5	3.56
23	LG23 (Chr.25)	6	23.5	3.92
24	LG24 (Chr.26a)	14	101.8	7.27
25	LG25 (Chr.26b)	4	20.2	5.05
	**Total**:	**194**	**1187.0**	**6.49**

**Table 5:** Linkage group (LG) / chromosome (Chr.) information in the F_3_ population.

#	Linkage groups	Number of mapped marker loci	Total map length (cM)	Mean map distance/Marker
1	LG01 (Chr.01)	6	48.3	8.1
2	LG02 (Chr.03)	5	24.8	5.0
3	LG03 (Chr.04)	3	46.9	15.6
4	LG04 (Chr.05)	11	55.9	5.1
5	LG05 (Chr.06)	5	29.0	5.8
6	LG06 (Chr.10)	10	40.3	4.0
7	LG07 (Chr.11a)	6	30,5	5.1
8	LG08 (Chr.11b)	3	4.4	1.5
9	LG09 (Chr.12)	9	80.5	8.9
10	LG10 (Chr.14)	4	28.8	7.2
11	LG11 (Chr.15)	14	71.0	5.1
12	LG12 (Chr.16a)	9	14.1	1.6
13	LG13 (Chr.16b)	3	10.1	3.4
14	LG14 (Chr.19a)	6	28.6	4.8
15	LG15 (Chr.19b)	12	100.5	8.4
16	LG16 (Chr.20a)	4	28.6	7.2
17	LG17 (Chr.20b)	9	39.3	4.4
18	LG18 (Chr.21)	7	52.8	7.5
19	LG19 (Chr.23)	3	36.7	12.2
20	LG20 (Chr.24)	8	36.2	4.5
21	LG21 (Chr.25)	5	5.5	1.1
22	LG22 (Chr.26a)	3	25.3	8.4
23	LG23 (Chr.26b)	4	14.0	3.5
24	LG24 (Chr.26c)	9	23.3	2.6
	**Total**:	**158**	**875.4**	**5.54**

### QTL maps

Linkage maps and phenotypic trait evaluation data were used for subsequent mapping of quantitative trait loci (QTL), including photoperiodic flowering in both populations. A total of 24 QTLs were identified and linked with six traits related to photoperiodic respond by composite interval mapping with LOD ≥2.4 (p≤0.05 after 1000 times permutation test; Table 6) that explained at least 18% of the trait variations. Four QTLs for NFB, FDR, and PhFl traits were detected at high threshold of LOD≥5. QTL-mapping analysis revealed 6 QTL associations with flowering-time and photoperiodic flowering (FT, FDR, and PhFl; Fig 6, Table 6) on chromosomes 5, 12, and 26. It should be noted that irradiation mutation is non-specific and it may have randomly generated many changes either related to or unrelated to photoperiodic flowering (S2 Fig; S2 Table) in irradiation mutant genome. However, some QTL regions associated with morphological traits related to flowering and/or maturity characteristics of cotton, such as node of first fruiting branch (NFB), number of buds (NBD) and number of opened bolls (NOBL) explained 6–85% of trait variation (Fig 6, Table 1). QTLs for these NFB, NBD and NOBL traits were found on chromosomes 4, 5, 13, 24, and 26 in the F_2_ population, while two QTLs for number of nodes (NND) were identified on chromosome 1 and 21 with LOD scores of 3.31 and 4.96, respectively. According to Guo *et al*. [47], QTLs for NFB were found on chromosomes 16, 21, and 25 in *G*. *hirsutum* L. There were several QTLs found to be significantly associated with other morphological traits investigated (see S2 Fig; S2 Table).

**Table 6:** Genomic distributions of SSR markers and photoperiod-related QTLs identified in the F_2:3_ mapping population of this study.

#	Pop.	QTL	LGs (Chr)	Linked markers	Position (cM)	LOD	Add. effect	Dom. effect	Literature reports	
									Various QTLs and their chromosomal locations	Reference
1	F_2_	*qNND*	LG01 (Chr.01)	JESPR289_90-TMB0062_280	7.36–16.35	3.31	-11.93	14.09	Fiber length (Chr.01)	Shen et al. [48]
2	F_2_	*qNFB*	LG04 (Chr.04)	GH117_230-BNL2572_240	11.80–29.80	3.1	-3.3	-1.3	Fiber length (Chr.04)	Qin et al. [49]
3	F_2_	*qPhFL*	LG05 (Chr.05)	CIR373_175-NAU3212_190	0.00–11.72	2.74	-0.30	1.32	Lint yield (Chr.05)	Wu et al. [50]
4	F_2_	*qNBD*	LG05 (Chr.05)	NAU3014_210-NAU861_215	42.90–45.20	2.74	-11.93	14.09	-	-
5	F_2_	*qNFB*	LG05 (Chr.05)	GH83_135-BNL542_260	60.90–73.10	3.45	3.42	0.96	Fiber fineness (Chr.05)	Yu et al. [51]
6	F_2_	*qPhFL*	LG11 (Chr.12)	BNL3835_195-NAU1278_240	32.27–40.55	3.45	2.92	0.99	-	-
7	F_2_	*qNND*	LG20 (Chr.21)	CM23_115-TMB2038_125	14.40–30.20	4.96	-1.87	2.89	Verticillium wilt resistance	Bolek et al. [52]
8	F_2_	*qFDR*	LG24 (Chr.26)	BNL840_160-JESPR92_195	39.10–39.40	4.24	226.8	116.9	Fiber length	Abdurakhmonov et al. [53]
9	F_2_	*qNFB*	LG24 (Chr.26)	BNL840_160-JESPR92_195	39.10–39.40	4.33	2.19	2.61	-	-
10	F_2_	*qNFB*	LG24 (Chr.26)	NAU3006_220-NAU2913_245	40.00–70.30	5.51	5.41	5.67	-	-
11	F_2_	*qFDR*	LG24 (Chr.26)	NAU3006_220-NAU2913_245	40.00–70.30	5.7	385.6	381.9	-	-
12	F_2_	*qPhFL*	LG24 (Chr.26)	NAU3006_220-NAU2913_245	40.00–70.30	7.20	3.06	1.46	-	-
13	F_2_	*qFT*	LG24 (Chr.26)	BNL341_130-NAU2750_175	76.30–80.90	2.57	11.71	6.83	Seed cotton yield (Chr.26)	Wu et al. [54]
14	F_3_	*qNND*	LG08 (Chr.11)	JESPR296_205-NAU1014_175	3.20–15.50	3.39	-8.3	11.76	Fiber elongation (Chr.11)	Shen et al. [55]
15	F_3_	*qPhFL*	LG08 (Chr.11)	JESPR296_122-NAU1014_175	3.20–15.50	4	-0.43	0.36	Fiber elongation (Chr.11)	Shen et al. [55]
16	F_3_	*qFDR*	LG08 (Chr.11)	PhyA1_122-BNL625_130	3.20–16.90	3	-2.3	1	Fiber length	Kushanov et al. [38]
17	F_3_	*qFT*	LG12 (Chr.15)	BNL2920_155-JESPR152_175	0.00–2.80	3.22	-2.95	7.99	Node of first fruiting branch (Chr.15)	Guo et al. [47]
18	F_3_	*qNBD*	LG12 (Chr.15)	BNL2920_155-TMB1660_210	0.00–16.54	2.66	8.48	-3.71	Node of first fruiting branch (Chr.15)	Guo et al. [47]
19	F_3_	*qFDR*	LG15 (Chr.19)	TMB1599_135-NAU3935_230	2.29–40.67	3.72	4.41	1.74	-	-
20	F_3_	*qPhFL*	LG15 (Chr.19)	TMB1599_135-NAU3935_230	2.29–40.67	3.50	0.41	0.24	-	-
21	F_3_	*qFDR*	LG20 (Chr.24)	BNL1521_140-BNL2616_145	0.00–5.10	3.49	-2.52	4.88	Fiber micronaire	Shen et al. [56]
22	F_3_	*qNBD*	LG20 (Chr.24)	BNL1521_140-BNL2568_230	2.29–40.67	3.36	2.46	17.88	Fiber micronaire	Shen et al. [56]
23	F_3_	*qFDR*	LG22 (Chr.26)	JESPR92_195-BNL3510_135	17.30–35.07	3.54	0.44	0.51	Fiber length	Abdurakhmonov et al. [53]
24	F_3_	*qPhFL*	LG22 (Chr.26)	JESPR92_195-BNL3510_135	17.30–35.07	2.57	3.95	4.47	Fiber length	Abdurakhmonov et al. [53]

The candidate genes of phytochrome A (*PHYA*) and B (*PHYB*) [38, 57] were localized on the linkage maps on chromosomes 11 and 10, respectively. Although the *PHYA1* CAPS marker was not associated with any photoperiod related traits in F_2_ population, it was associated with the traits of photoperiodic flowering, flowering duration and number of buds of the genetic map for the F_3_ population with LOD ≥3.0 (Fig 7, Table 6) and explained 72–99% of the trait variations. This could be due to differences in flowering plant percentages observed in F_2_ and F_3_ as well as selection of only flowering plants for F_3_ generation evaluation. According to Abdurakhmonov *et al*. [7], somatically regenerated *PHYA1* RNAi plants exhibited early-flowering traits.

The early maturity of cotton, which could be impacted by the photoperiod sensitivity and flowering-time, is one of the primary objectives in cotton breeding program [58]. Investigation of the flowering-time and photoperiod sensitivity traits is important not only in development of early maturing varieties of cotton, but also in introgression of valuable traits from wild species to existing varieties of cotton, and thus expanding the narrow genetic base of *G*. *hirsutum* [26]. Therefore, understanding the molecular mechanisms of flowering habits would greatly accelerate the molecular breeding of cotton. Although the genetic architecture underlying this trait has been investigated in populations specifically designed for QTL detection [24, 35, 47], the genetic control of flowering-time in photoperiodic material remains poorly understood. The results of QTL-mapping and identified molecular markers linked with the flowering-time and photoperiod insensitivity in an irradiation mutant line of *G*. *darwinii* in our study should be important tools and a donor source for marker-assisted selection (MAS) for early maturing cotton, and will facilitate transfer of desirable genes from wild species such as *G*. *darwinii* into cultivated varieties of cotton.

### *In silico* annotation of QTL regions

**Fig 8:**
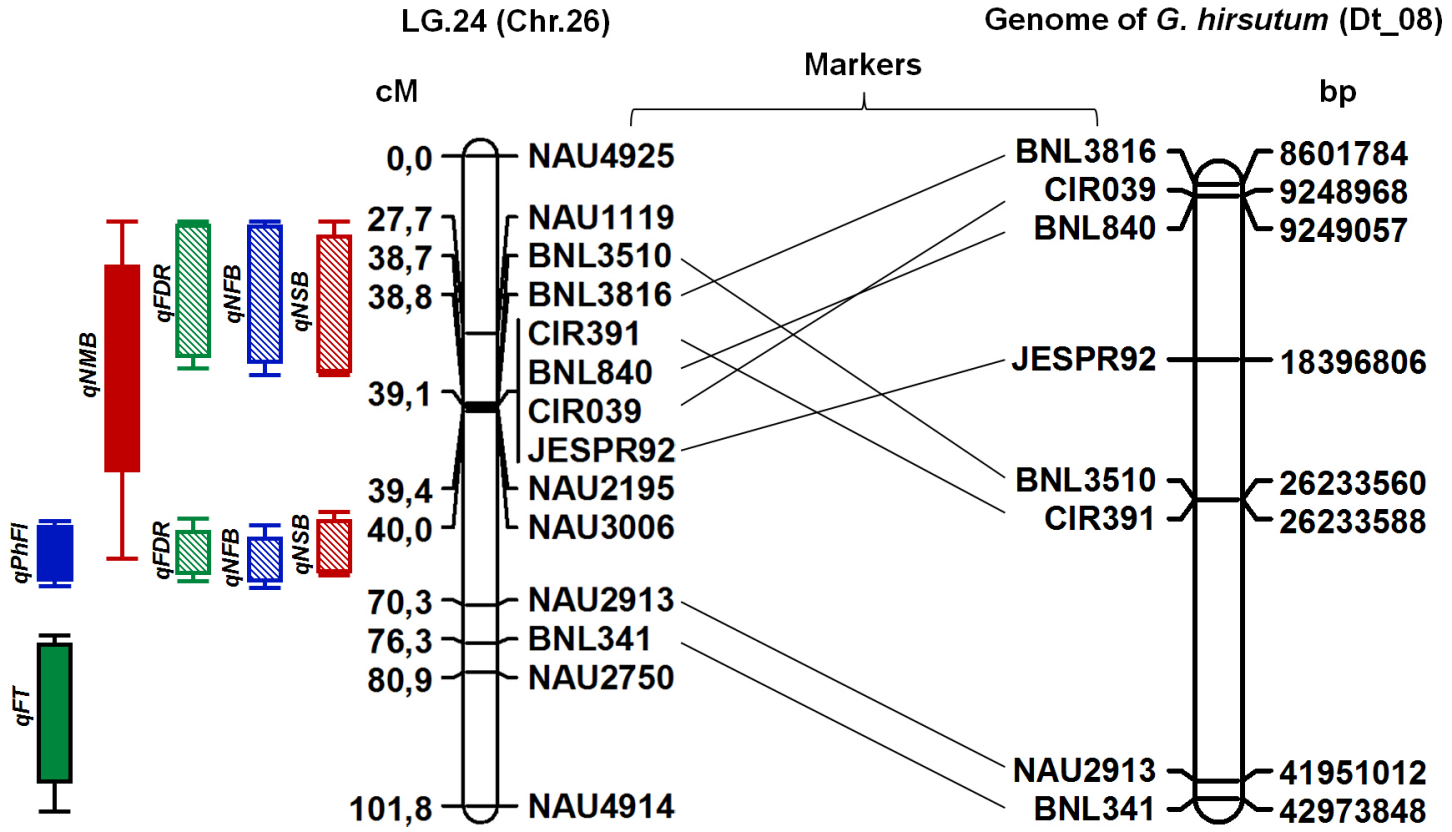
Comparison of mapped QTL markers with actual positions on the genome of *G*.*hirsutum*.

According to *in silico* PCR results, eight out of 14 DNA markers located on linkage group 24 (Chr.26) [30] were virtually amplified from Dt_08 of annotated *G*. *hirsutum* (Fig 8) genome sequence database, which corresponds to the chromosome 24 of *G*. *hirsutum*. The exact cause of marker position difference in linkage mapping and *in silico* amplification is unknown. It could be due to a possible difference in synteny of *G*. *darwinii* and *G*. *hirsutum* genomes and/or surmised that there might be some chromosomal rearrangements in irradiation mutant line genome, which requires further study. Therefore, according to *in silico* amplicon locations, the chromosome 24 was referred in the discussion of candidate genes below.

The Table 7 shows the genetic positions markers on the genome of *G*. *hirsutum*, both nucleotides (based on *in silico* PCR), and in centimorgan (cM) defined by QTL-mapping in the F_2_ population *G*.*darwinii* (Table 7).

**Table 7:** *In silico* PCR annotations for loci located on LG24 (Chr.26).

#	Marked QTL region	Genetic position in F_2_ generation (cM)	Chromosome positions on the genome of *G*.*hirsutum*	Beginning of nucleotide coordinates	Ending of nucleotide coordinates	The size of virtual amplicons (b.p.)
1	BNL3510	38.7	Dt_chr8 (D12)	26 233 560	26 233 695	136
2	BNL3816	38.8	Dt_chr8 (D12)	8 601 784	8 601 982	199
3	JESPR92	39.1	Dt_chr8 (D12)	18 396 806	18 397 937	1132
4	BNL840	39.1	Dt_chr8 (D12)	9 249 057	9 249 206	150
5	CIR391	39.1	Dt_chr8 (D12)	26 233 589	26 233 689	101
6	CIR039	39.1	Dt_chr8 (D12)	9 248 968	9 249 127	160
7	NAU2913	70.3	Dt_chr8 (D12)	41 951 013	41 951 248	236
8	BNL341	76.3	Dt_chr8 (D12)	42 973 849	42 973 980	132
9	NAU4925	0.0	At_chr8 (D12)	42 744 792	42 744 931	140
10	CIR039	39.1	At_chr8 (D12)	19 241 701	19 241 895	195

To identify candidate genes on chromosome 24 (Dt_08 of *G*. *hirsutum*), marker regions between CIR039 and BNL840 (located almost in similar overlapping position in the genomic sequence maps; Table 7), as well as 100,000 bp downstream of marker region of CIR039 and 100,000 bp upstream of marked region by BNL840 were selected. This selection covered the 9,148,968–9,349,206 region with the size of 200,238 bp on chromosome 24, which was then used to identify candidate genes and their further *in silico* annotation. The markers CIR039 and BNL840 which lies on the same position (39.1 cM) on the linkage map separated from one another by only 89 base pairs distance, while other markers JESPR92 and CIR391 were located at coordinates 18,396,806 and 26,233,589 nucleotides on this chromosome. If, 1 cM equals ±500,000 nucleotides [59] then the last two markers are located approximately 18.2 and 33.9 cM apart, respectively, from the first pair of markers. This can be attributed to the difference in the genomes of *G*.*darwinii* and *G*. *hirsutum*. Moreover, CIR039 was virtually amplified from another chromosome that has a homology to 24 on chromosome 08 (At_chr8) with 195 base pair size. Also, NAU4925 which is located on the beginning of the linkage group (0.0 cM) was found on chromosome 08.

Analysis of selected sequences with the help of AUGUSTUS identified 11 genes. Some of these genes have a number of putative transcripts so the total number of biological meaningful and putatively expressed sequences generated was equal to 17.

A comparative analysis of 17 putative amino acid sequences using protein BLAST algorithm has identified only one predicted protein sequence with known function in cotton and other organisms. The remaining 16 putative sequences did not match with any annotated sequence. The matching annotated sequence within 200,238 bp on chromosome 24 (i.e., corresponding to the marker regions CIR039 and BNL840) encoded the alpha subunit of casein kinase II. Casein kinase II (CK2) is a serine/threonine protein kinase that is involved in various physiological processes of plants including circadian rhythms, light signaling, stress response, flowering time, lateral root development, and control of the cell cycle [60, 61]. CK2 is a tetrameric enzyme which composed of two catalytic α subunits and two regulatory β subunits. In plants, unlike animals, the two subunits (α and β) of CK2 often are encoded by multiple genes. For example, *Arabidopsis thaliana* has 8 genes coding for CK2 subunits (four α- and four β-subunits) [62]. α- and β-subunits are involved in the regulation of circadian rhythms by phosphorylation of central components *CIRCADIAN CLOCK ASSOCIATED 1* (*CCA1)* and *LATE ELONGATED HYPOCOTYL* (*LHY)* genes [63, 64, 65]. According to literature reports [60, 61, 66], the highly conserved serine/threonine specific casein kinase (CK) controls various processes in the signal transduction of many eukaryotes including yeast, mammals and plants. In particular, CR involved in the control of flowering-time in plants through a complex interaction between endogenous components of circadian rhythms, environmental factors (including seasonal changes in day length (photoperiod) and temperature [60–68]. For instance, triple mutants for three α-subunits of CK2 [61] expressed late flowering phenotype both in long- and short-day conditions and responded to vernalization treatment. This suggested that CK2 α-subunits may function in the autonomous pathway to regulate flowering time [61]. Furthermore, Sugano et al. [67] reported that transgenic lines of Arabidopsis, overexpressing regulatory β-subunit of CK2 (CKB3), diminished photoperiodic response and flowered early in long- and short-day photoperiods. Therefore, the CK2 gene identified in the photoperiod QTL region of chromosome 24 appears to be a strong candidate for the gene underlying this trait.

Thus, our efforts identified several SSR and CAPS markers closely associated with genes controlling photoperiodic flowering in the cotton populations created in our study. *In silico* analyses found potential support for the biological relevance of mapped markers. Results from this study enhance our understanding of photoperiodic control of flowering-time. The predicted genes, mapped DNA-markers and irradiation mutant donor *G*. *darwinii* line will be useful to improve cotton flowering characteristics using modern marker-assisted selection and biotechnology tools.

## Supporting information

S1 Data F_2_ population map data file. (PDF)

S2 Data F_3_ population map data file. (PDF)

S3 Data F_2_ population genotype and trait data file. (PDF)

S4 Data F_3_ population genotype and trait data file. (PDF)

S1 Fig Histogram of some morphological traits studied in F_2_ mapping population derived from pre-mutagenesis and irradiation mutant lines of *G*. *darwinii*. (TIF)

S2 Fig Genetic linkage map of the F_2_ population showing the location of QTLs for some morphological traits studied in the F_2_ population. (TIF)

S1 Table Genetic variances and estimated broad-sense heritability of several morphological traits in the F_2_ population. (DOC)

S2 Table Genomic distributions of SSR markers and identified QTLs for several morphological traits in the F_2_ mapping population of this study. (DOC)
