# Supplementary material for: QTL mapping for flowering-time and photoperiod insensitivity of cotton Gossypium darwinii Watt
Source: PLoS One. 2017 Oct 9;12(10):e0186240. doi: 10.1371/journal.pone.0186240 (PMC5633191; doi:10.1371/journal.pone.0186240)
Supplement: S1 Data — (PDF) [file pone.0186240.s001.pdf]

[Header]
Study name Photoperiodic flowering
Mating string S
Genotype symbols 12345-
Parent1 Darwinii
Parent2 Mutant

[Locus]
TMB0799 12 0.0
33221311313322233213223331122313113332333121213133333333131231133321233332111332
3212333133233133333133222331123133231133131323232
JESPR270 12 27.0
31231113333333223332233311131311313332333123313112333123133333123331113232111132
22333121312-31233333333233332322313233222131332222
NAU1237 12 31.5
312311133333332233322333111313113133323333233131123331231333333323331113232311122
2233313131233123333331132-332223313233222133332232
BNL3835 12 32.3
312311133333332233322333111313113133323333233131123331231333333323331113232311122
2233313131233123333111322332223313233222133332233
NAU1278 12 40.6
313311133232332232322331111213113133323133233131323331233333333333331113232311121
3233333133233123333211323333323313333322133332233
BNL1679 12 64.6
313321333212212212332333333232332313323113233133332322133332333131213133333331331
3333333132331322233213323333333112333122132333233
CIR148 12 65.3
313321333212232212232333333232332313323113233133332322133332333131213133333331331
333333313233132223321332333333112--3122-323-3233
NAU5047 12 75.6
21332213321233221233232332323233231232113323333313232213333333113121312332311331
33331131323333222132133233333131112333122332333233
CM85 12 82.8
21222213321233231233232332323233231232113323333313233213331333133123312332311331
331311313233333331331313333312111223-122333333233
GH98 09 0.0
32122313312313321112332233312323321213321213133233212233211231333113222213323313
3311333313333323113232122132321121321321313133333
GH27 09 2.0
32123313312313321112332233312323321213321213113233212233211231333113222213323311
3311333313333323113232122322321121321321313133333
GH112 09 2.0
32123313312313321112332233312323321213321213113233212233211231333113222213323311
3311333313333323113232122322321121321321313133333
TMB0184 09 8.9
32123313312333321112332233312333321313331211113233312233213331333113223213123311
131113333113333233132321233221211213213213113333-3
PhyB 10 0.0
322311223333131233333333212233311331112113211131232331113222313333121133333333233
3333233323313332212321332323332333313132333333332
PhyB2 10 0.0
322311223333131233333333212233311331112113211131232331113222313333121133333333233
3333233323313332212321332323332333313132333333332
BNL2872 10 4.0
322313223333131233332333212232311331112113213131232331113222313213121133333333233
1333233333313332212323332323332333323132333333332
TMB0325 10 4.3
3223132233331312333323332122323113311-12-1321--312323-11132223132131211333333332-3
1-332333333313332212323332323332333323132333333332
TMB0307 10 4.4
322313223333131233332333212232311331312113213131232331113222313213121133333333233
1333233333313332212323332323332333323132333333332
BNL2705 10 4.4
322313223333131233332333212232311331312113213131232331113222313213121133333333233
1333233333313333-21232333232333233332313233333332
TMB0380 10 11.6
2223132233331112333323332322232311331313113213331232331313222313213121123232313233
12333333333133323123233323221123333233133333313332

CM67 10 14.4
2223132233331112331233333223231133131311321333123233131322231321212112323231333312233333333--33231232333232221123-32331331333133332
BNL2960 10 15.4
32233123233333133333213233333311333312112121133232333133222313333121133333331233333323333331333321212133232333233131313233333333332
BNL1665 10 25.7
32223222332331133312332333233211311333113213331232311313232313212121123213312333122331233333123331232223222211213323313313331331312
TMB1745 10 29.1
32223322332331133312332312233211313133313213331232311313232313212121123213312333122331213333121331232223222311213323313313331331322
JESPR65 05 0.0
33333313332231333333313111323223133331331231312132323323113333232133231332133233132111211312331213122213332312312332323231231313113
BNL3995 05 7.9
33333313331233333333313131321223133331331231312332323321113333233112231332131223122313313313333213122213332112133332333231232323113
NAU5149 05 9.1
33333313331233333333313131321223133331331231312332323321113333233112231332131223122113313313333213122213332112312332333231231313113
NAU3569 05 10.8
133333133312133333333131313212331333313312313123323233211133332331122313321312231223133-3313333213122213332112112332333231231313113
TMB0191 05 11.2
1333331333121333333333131-2123313333133123131233232332111333323311223133213122312231331331333321312221333211211233233323131313113
NAU2296 05 11.2
133333133312133333333131313212331333313312313123323233211133332331122313321312231223133133133332131222213-3211211233233323131313113
NAU2001 05 12.4
133333133312133333333131313212331333313312313122223233211133332331122313321312231223133133133332131222213-3211211213233323131313113
GH211 05 18.7
33333313331213333333323121321233133331331331313222323321313233233112231212131223122313333313333333122213322312112132333231331313111
NAU2140 05 24.4
33333313331213333333323123323233133331331333113222323321313333233312231213 1-1233123313333313333333132212322332112132333231331313-32
JESPR241 05 30.0
332333133312133333333321--3323233133333333333311322232332131333333231223121313323112311333321 33--33313221232333211213233323131313121
BNL542 05 39.4
3-233313321233233333332112332323312333333333311322232332131133333231223121333313221322333312333333313221222323233311333323131313121
GH83 05 51.6
31233333331222233333332112312323332332333133323323232323131333333231232121333323312311312321333313313321223323233311331323131323 1221
BNL3992 05 53.4
31233313331222233333332112312323332332333133313323232323131333333231232121333323312311312321333313313121223323233311331323131322 1221
NAU861 05 67.3
312133131312-2231333333123112123333232313132113323232331111333333123133312333132-311311312123333213111131233323333331132112133221221
NAU3014 05 69.6
31213313131222231333333123332323333232313132333323232331111333333123133312333132331131131212333321331313123333233333311321121332212 21
NAU3325 05 86.3
31213333133223331233121212333232333322111322313232333311133333331231333123121111331311111123133313313333123333221133333211213322 1233
NAU5160 05 88.7
11313333133223331333121212333232333322111322313332333311133333331231333133121111331311113123133313313333123333221133333211213322 1233
NAU5015 05 95.2
111132321312213331321332132323132313221212222133323331311223333313233111312133133123311312313333311333121312213333332133112321231
NAU3212 05 100.9
11113232131223333233133212332313233322131222213332333331122333313333311131211313

312311131231333333133-121313211333332113133321231
CIR373 05 112.6
1131333213122333133312121233331323332213132231333233331113333331233333133121-113
31231113123133323323333212333122333-31223233312133
TMB1599 19 0.0
1331323333232131333333322333113333333313131333323332133332231131313123313323232131
3223213323331333313133323313333311133313333311331321
GH71 19 1.5
3331323333232131333333322333112333133131313333233321333322311313131233133232321-1
322321332333133331313332331333331113333333311331321
BNL285 19 12.2
33331223132321323333333113333112133333133113333133332133132231131312123313323333331
132321331333133133232322331333331112333333331331311
NAU3935 19 23.1
333312331223312233333332233311223333331331133332333233331322113331112121313332333331
133321332333133133232332331333311112323333331333321
BNL852 19 29.0
33331231122331221333331221331122333333133311333223323231322113311121313133312333331
133321332333133133232332331333311312323333331113323
TMB0189 19 30.5
33331231122333221333331221331122333333113311333223333231322113311121313133312333331
133321332333133133232332331333311212323333331113323
GH109 19 31.3
33331231122333221333331221331122333333113311333223333231322113311121313133312333331
333321332333133133232332331233112123233333311133323
TMB0366 19 39.6
3323133-1333332213333122123112231333133331323223333213323113311123312131123333-1
3-3-2113233313113333233213123311212323333313133-3
BNL4096 19 40.9
332313331331332213333122123112231333133331323223333213323113311123312131123333331
33332113233312113323233213123311213123333331313323
BNL3875 19 47.4
33233323133333321233312212311223133313333112332333331332311331112331213112113331
33332113333313133332233313133231212323333331313323
TMB1489 19 60.4
33233323133233321233312312211333313313323132332331331313313311112211313112133321
3333233133131313331232331311323121212313331213321
BNL3977 19 63.9
33233323133233321233312312211333213313323132332331331313313311112311313112131321
3333233133131213331232111111123121212313331213331
TMB1645 19 88.0
33313331332232321233313122233132313212323133333131333313213113113111313113331331
312223331333332223312123313113213313333331311133331
CM209 19 89.2
23313331332232321233313122333132313212323133333131333313213113111111313113331131
312223331333332223312123313113213313333331311133331
JESPR218 19 100.8
33313331332332323213313112333132313132323123213113333313213111311331113113311333
31222333133-33221312123312113312-13323333131113332
CM42 19 101.2
33313331332332323213313112333132313132323123213113333313213111311331113113311333
31222333133333221312123312113313313323333131113332
CM3 19 101.2
33313331332332323213313112333132313132323123213113333313213111311331113113311333
31222333133333221312123312113313313323333131113332
JESPR236 19 101.6
32313331332332323213313112333132313132323123213113333313213111311331113113311333
312223331333-322131212331211331331332333131113332
NAU4925 26 0.0
231313232212322313233232221333113233321133222322333332233331232333333323321313311
1113123323112233312323313123233313231232233331313
NAU1119 26 27.7
321312333131332312313222233312332223333121323332221232233332121333231333221211323
231332222212323333233111312323331123-332233331233
BNL3510 26 38.7
13131333313133231231323233313332232333313132333222123323333212133322333122311333
2313322323123233332331113133233333211333233221231
BNL3816 26 38.8

13131333313133231231323233313332232333313132333222123323333212133322333122331133 3
231332232112323333233111313323331321133323322123 1
CIR391 26 39.1
131313333131332312313232333133322323333131323332221233233332121333223331223311333
2313322323123233332331113133233313211333233221231
BNL840 26 39.1
131313333131332312313232333133322323333131323332221233233332121333223331223311333
2313322323123233332331113133233313211333233221231
CIR039 26 39.1
131313333131332312313232333133322323333131323332221233233332121333223331223311333
2313322323123233332331113133233313211333233221231
JESPR92 26 39.1
131313333131332312313232333133322323333131323332221233233332121333223331223311333
2313322323123233332331113133233313211333233221231
NAU2195 26 39.4
131313333131332312313232333133322323333131323332221233233332121333223331223311333
2313322323123233332331113133233311211333233221231
NAU3006 26 40.0
131313333131332312313232333133322323333131323332221233233332221333223331223311333
2313322323123233332331113133233313211333233221231
NAU2913 26 70.3
131323332133333113332332111333333232332131333333123333233131332333333231333132323
3313323223121312131333113131213312321332233213212
BNL341 26 76.3
131323332133333113332332111333333232332131333323123333233131322333333231333131123
3313323223221312232331112131213312321332233223212
NAU2750 26 80.9
111323332133313111332332111333333232312121333323123333213133322333333211333131123
3313323223223312232331112131211312321132233223212
NAU4914 26 101.8
111323332131313121312332113313133333231313113332332332111213333212333211313322132
3313323221223332223311133331121111313312333333323312
BNL1066 11 0.0
311121313333333333231-331131311131333233332223132132331131133213312333123221322 1
33222112131312311312333133131321333223322333332321
JESPR296 11 17.3
211123312333331323231313113131111113212233322221321321331311313113133332222213223
12321331111123131133333132131333332221122333333331
PhyA 11 19.7
211123312333331323231313113321111113212233322221321321131311333113133332222313223
12121333111123131133333132131333332221122333333331
NAU1014 11 31.1
233333312311331333233313333333111132122333222213213211333113333112133332222312333
121313111311331313332313313133333223112233333 1321
TMB0064 11 32.3
233333312311331333233313333333111332122333222213213211333113333112133332223112333
1213131113113313133323133131333332231122333331321
BNL625 11 36.8
233333312311331333233313333333111332122313222213212213333313333112333332223112333
1213131113133313333333133331133332 23-322333331321
TMB0359 11 41.0
233333312311331333233333333333111332122313111233212213333313333112333332223112313
12131311133333133333331333311333322313223333331321
TMB0429 24 0.0
332133232333221331321231111122331-223331333112123123232131322313323312132223312 3
323313112331-331113332321211331313233321322311323
BNL2568 24 11.5
332133212333221331321231131123331322331133311232312323213132231332331213222331 23
3231133122321-33311333232121133121323332132231323
GH171 24 13.7
33213321233322133132123113112133132233113 33-123231232321313223133233121322233123
32311331233123333113332321211331-1323332132231323
BNL2655 24 14.2
332133212333221331321231131 12----------------232312323213132231332331213222331 23
32311331233123333113332321211331313233321322-1323
GH272 24 15.9
332133212333221331321231131121331322331133311232212323213132231332331212222331 23
32311333333123333113332321213331313233221322311 23

BNL1521 24 22.3
132133232332221331221211311132331122233133311313332323213132231331331213212331231233333323332333131323232131113131323332132233323
BNL2616 24 23.4
3321332323332213312212113111223311223331333112133123232131322313323-1213212331231233133323332332111323232121132131323332132233-323
BNL252 24 28.5
33213323232322133132123112122311323321211333123221232321313223133233121322233123
12311331213323332113332323213331313213221322321133
TMB1421 01 0.0
31233331233323221133323233311233231323323332312333333312232213222312323122312212
332111332222333321231133322322113331323333111331
JESPR289 01 7.3
3123333121222322113332323331323323112312233233333333331223121322-312323122332212
332111333122313121231133122322133331-233331113311
TMB0062 01 16.3
21233331213232223133323233313223231223122332333333133312231213222212323323332212
33211333312331332123113312212313233332333313333331
BNL3888 01 27.8
21232331213233223132323322331223231223133123333333132312331213222232323323231212
3321133331333133213333331221233323323233231333331
CM92 01 29.9
21232331213233223132323322331223231223133123333333132312311212222232322323231212
332113333133331-3213233331221233323323233233333333
BNL3580 01 34.6
21232331213233223132323322231323231223133123333131132312313213222332323223231312
33211333313331332132333312213333233233232333333333
GH75 01 53.6
33232311313333233122323123221323331232133123331131333313333213221232223221231332
1121133333323233213221131331313221123123233333123
BNL686 23 0.0
32312333123313213311123333311323231323111331333313231312133232311323311331333323
2333233121213333331113121331323332311332123321323
TMB1701 23 28.9
321133211213133133331333211111323231231213233333131323211213332213333231131133333
23132223332332313111113231131322232211222323333333
JESPR151 23 30.1
321133211213133133331233211111323231231213233333131323211213332213333231131133333
31132223332332313111113231131322232211222323333333
TMB1425 23 51.3
31313323121232331333221331111233233333213233313111323213313332312333231131133313
312322333332312233131312312213233332311222331131233
TMB0382 23 65.6
31212133121232331332221313333213213133333233313111323233312332112-33331333132313
3123221333211333313231233233323332-31222331131233
JESPR110 23 75.5
31232133131232331232221333233213213133333233313111323233332333112331131333332313
31232212232133333332312-2233123332131232231123322
JESPR195 17 0.0
2333222333332222333332333332233333233111231131313313333323213311131332133223131311
13312333-3223133331132323332331111331332223111121
BNL4003 17 6.1
23232221333322233333233333223313223111123113133331333333213311131332133223131311
1331233333223133331133323332131131313332223311123
BNL3955 17 7.1
23232221333322233333233333223313223111123113133331333333213311131332133223131311
1333233331223133331133323322131131313332223311123
TMB2018 17 8.8
23232221333322233333233333223313223131123113133331333333213311131332131223133311
1333233333223133331133323322131131313312223311123
BNL3994 26a 0.0
11223313333322131333222332333131123113133222112113321211331213321123333123212232
2333322133211133233311121121213333322233332321212
TMB0120 26a 3.6
112233133333221313332223323331311231131333-3112113321211331213321123333123212232
2323323133212133233311121131313133322233332321212
GH52 26a 3.7
11223313333322131333222332333131123113133333112113321211331213321123333123212232

2323-2313321213323331112113131-133322233332321212
GH200 26a 20.2
13223111233312131333222333231311231111--------12133331311331313121123133123232322
2233323333213313233311131333313123332333232321212
GH198 02 0.0
11331221133322333321313313331323331133322233333122122133233232133321111323233313
1212333333233313131322313333222332133233112312132
TMB0471 02 0.3
11131221133322333321313313331323331133322233333122122133233222133321111323233313
1212333333233313131322313333222332113233132312133
BNL3971 02 1.9
11131221133322333321313313331323331133322233333122122133233222133321111323233313
1212333333233313131322313333222332113233132312133
JESPR179 02 22.6
31131231113322231321311311333121333313333223333123122333232323113323311312333333
121233332232213111322112121222331313231132113131
TMB0809 04 0.0
313231311131322222211332233333233133321331132113321313231321331123332333332231133
2311313233313331232311312121331323333312231331211
GH117 04 11.8
33123133113132232221133123333223313132133113331332121323132333112333233312233123
2311312333313231233313312121231321323331231211211
BNL2572 04 29.8
333233331133333323213331233332331121331331133321123233333332331112331223333212123
233331233311323223331321232123132132332131321333-
GH118 09a 0.0
32223112212333233232133313331333331313313211213221332332333332313333231311123313
11113232332321223133111-112313131322113333131-333
BNL1414 09a 17.2
322231122213332332323221333313233133-3131213213123333132311332313333331311122313
11131333332-23223133211311211333-3233333333313333
BNL4028 09a 22.1
322231122213332332333221333313233133131312133121233331323113323332333331311132313
111313331323212233333311311213333132333333323313333
BNL3065 16 0.0
33313333323132213323312233221231121311233313233123322231131313323133312323233333
311211213312332331323223131323--33132322211332132
GH2 16 29.6
33133331323313331333132311211231131211323312233133112222121333321333133233213212
2332322313123122313332331323231332113322211332332
JESPR128 16 30.3
33133331323313331313132311211231131211323312-33133112222121333321333133233213212
2332322313123122313332331323231332113322231332332
JESPR32 16 30.3
33133331323313331313132311211231131211323312233133112222121333321333133233213212
2332322313123122313332331323231332113322231332332
TMB1271 16 30.3
33133331323313331313132311211231131211323312233133112222121333321333133233213212
2332322313123122313332331323231332113322231332332
JESPR237 16 30.6
33133331323313331313132311211231131211323312233133112222121333321333133233213212
2332322313123122313332331323131332113322231332332
JESPR297 16 32.2
33133331323333331333132311211231131211323312233133112222121313321333113233213212
2332322313123122313332331323231132133222211332332
BNL2734 16 36.1
33133331323313333313132311211333331211323312233123113222321333331331132233213212
2332322333123123313333331323231332113322231332332
BNL3008 16 36.1
33133331323313333313132311211333331211323312233123113222321333331331132233213212
2332322333123123313333331323231332113322231332332
TMB1409 16 37.3
33-33331323313333313132311211233331211323312233123113222221333331331122233213212
233232233312312231333333132323133211332223133-322
JESPR158 21 0.0
33333133231333332311231322332133323331233133332323331331333332132123333311323333
31323133123311211133231111133321333133-3221323312
CM23 21 14.4

3332313323133133331322332213213332233323312331232332123123133212312332331132233 2
3332313312311321113323331112312333313322221132213
TMB2038 21 30.2
133221322313113333132233233321333331332333231123333232332313331331231233332223 32
3232113333311321113223333113113333311333323333213
TMB0400 21 42.7
1332213222131123323332132333333333331132332331123323312322233331333133221332223 32
3232112333311321111222333113113332131313323233313
JESPR118 21 42.7
1332213222131123323332132333333333331132332331123323312322233331333133221332223 32
3232112333311321111222333113113332131313323233313
BNL3649 21 50.8
1332213322111123323232132333333333313323323213233233133322333313332333213222213 2
3232132333311221113322332112113332331313323----
BNL1551 21 53.0
1332213322111123323232132333333333313323321213233233133322333313332333213222213 2
2232132333311221113332333312113332331313323231212
BNL3279 21 88.5
3232313223133133323213132331132233333131333213231233213332333122332333231221233 2
2231232331331322133333113232123313333131133331321
JESPR152 15 0.0
32333233212113223132312213133133-133331112133331333132313312112333111331223331 31
2213333313223323313233323233323123213133133232313
TMB1664 15 22.3
3221322123331332311323221311333331333211123333332311133113121131331333312332313 1
2233312313321313213213133232323133211332333233313
TMB1660 15 25.7
322132212333133231132322111132-3313332331233333323111331131211313313333123323133
2232312313331313213213133232323133211332233233313
BNL2920 15 25.8
32213221233313323113232211113233313332331233333323111331131211313313333123323 1-3
2232312313331313213213133232323133211332233233313
TMB0201 15 28.0
3321322123331332311323221111323331333233123333322311133113121131331333312332312 3
22323123133313132132131332321231 23-1-332233333313
TMB0301 15 36.7
3321332131331332311123231111233313332331233332223131333131211311313332123321122
323231231313-3332132321332331231222113322331 33313
JESPR180 15 36.7
3321332131331332311123231111233313332331233332223131333131211311313332123321122
3232312313131333213232133233123122211332233133313
JESPR298 15 36.7
3321332131331332311123231111233313332331233332223131333131211311313332123321122
3232312313131333213232133233123122211332233133313
BNL4082 15 40.1
3321132131331132111123231111233313232231231322223131333131211311313332323321122
32323123 1-1313322132321332331231222113322331 33313
TMB0375 15 42.2
3321132131131132111123331111233333232233231332323131333131211311313332323321122
3232312312131332213232133233123122211332233133313
BNL3902 15 42.5
3321132131131132111123331111323333323223323133232313133313121131131333232332112 2
3232312312131332213232133233123122211332233133313
BNL1350 15 47.8
3321332131131112113123333111323333323223323133232312133313121131131331232332112 2
3232312312131332213232133223123112231333233333332
TMB1181 15 59.6
2321332333131312113123333313323231311223323133232312131313323131123331212332112 2
22323121131113322133121332231231 1332-333333212331
BNL786 15 60.0
2321332333131312113123333313323231311223323133232312131313323131123331212332112 2
2232312113131332213312133223123113322333333212331
CM82 20 0.0
1132331312333333311333133322222333232233321231333123231133333312333333323113333 3
2312323231323313231333323313313133333133123212313
JESPR235 20 0.0
1132331312333333311333133322222333232233321231333123231133333312333333323113333 3
2312323231323313231333323313313133333133123212313

BNL169 20 1.9
1132331312333333331111331333222223332322333212313333232311333333123333333323133333
231232222313233132313333233133131333331331232323 13
GH48 20 2.8
1122331312333333331111331133222223332322313212313333232311333333123333333323133333
231232222313233132313333233133131333331331232323 13
BNL119 20 4.6
112233131233331333111331133222223332322333212313333232311323333123333333233-3333
23123222231323313211333323313313133333133123 23-313
TMB1629 20 9.8
11223313323332133311133133322232333232213323331333323231132333312333333332-333333
33123222331323313211323323333313123333133323232313
GH59 20 12.3
31223313323332133311133133322232333232213333331333323231133333312333333332333133 3
331232223313233122113223233333-131233333333323232313
GH54 20 15.7
3122331332333213331113313333223232333221333333133332323113333333233333333323333333
331233233132311221132232233333131233133333323232313
BNL3948 20 19.3
312233133233321133311331333333232323332213313333333332323-1333333323333333333133333
331233233132311231132232233333131233133333123232313
TMB1630 20 46.2
2123131233233212112113123331333223213231331131323322323112333312323331333113312 3
3133-123313331133131322323333321232131331232333 31
GH82 06 0.0
21323321211113311323311332333333322112332313213122331223233133331131333331213232
1133313231233311333222133131321 1-3122121312323233
GH39 06 34.0
2313133321131312333321132333312333233232233121332323133332323112111131332123221 2
3211333321223311333222133321323121331311111333313
GH32 06 36.0
231313332113131232332113233331233323323223312133232313333232311211113133212322-2
32113333212233113332221333212231212213111112233 13
TMB0154 06 36.9
2313133321131312323321132333312333233232233121332323133332323112111131332123221 2
3211333321222311333222133321223121321313111223313
TMB1538 06 36.9
2313133321131312323321132333312333233232233121332323133332323112111131332123221 2
3211333321222311333222133321223121321313111223313
JESPR119 06 55.3
23111333311331323213231321233123312312332331212313223323133231323111333231232312
321122133-22333233333232232122313132131311123----
BNL1379 03 0.0
3333233123122232233331323311212123113231331113221131232222133131223333113212132 13
1231223323312223212322333231321133333123322111223
BNL3441 03 7.7
3313233131322232313331323311213123113231331113221131212322213333132333113212132 13
12313233333323333212322333333132112333312332231122 3
CM106 03 8.5
331323313132223231323132331121112311323133111322113121232221333313233311321213213
123132333333233321232233333132112333312332231122 3
TMB0564 03 8.9
33132331313322323132313233112111231132-1331113221131212322213 33-1323331132121321 3
123132333333233321232233333132112333312332231122 3
TMB1989 03 32.8
131113231213232323133113223332133231133313213332231313333223223313233331311231213
13233233333333323333223123112211332313213133133 32
BNL3558 18 0.0
313123213322213132322213222111322132232233231313333122331211323213111113323133
331133131321233333133233333332323-1223313312333112
BNL1079 18 10.9
333123213332211133222313222331322132312233332313133333133331331323213113113323121
23113313122133333213322333332323312333133123333 13
BNL2667 18 40.4
331123213213311233323312222312223212312233333333322313113121331321213312333113221
33131233332113231211323323233322133333113123322 13
GH224 25 0.0
1331322331111332322231333122311113232332311132311232333222333232333313231231131 1

3323331231332311113333232322221231313323323313212
JESPR215 25 0.7
13313223311113323222313331223111132323323111323112323332223332323333132312311311
3323331231331311113333232322221231313323323313212
JESPR227 25 0.7
13313223311113323222313331223111132323323111323112323332223332323333132312311311
3323331231331311113333232322221231313323323313212
CM27 25 0.7
13313223311113323222313331223111132323323111323112323332223332323333132312311311
332333123133131111333323232222212-131332332313212
CM13 25 0.7
1-31322331111332322-231333122311113232332311132-1123233322233-323333132312311311
3323331231331311113333232322221231 31-32332313212
NAU3171 25 23.5
11313323313323333331313121323311133333331133323112323233222333333233323133311132
33233313331331131332233 3-3223212312133233211132 13
BNL1495 13 0.0
13112133233312123132132231233133113333322311313311332333332223123133131312322223
3313311311222133221332331113113132312233312223333
BNL1421 13 0.0
13112133233312123132132231233133113333322311313311332333332223123133131312322223
3313311311222133221332331113113132312233312223333
BNL3623 13 7.8
13112133233312123132132231233133113333322311313311332333332223123133131312322223
3313311311222133221332331113113132312233312223333
GH215 13 10.3
13112133233132122332112223223123133333123311312311332333232223123133131312332333
33133113112-21332213223313111131333122323122233 32
GH34 13 24.0
11112333223331122222112223331333133323121311312311312231332233323133321113332333
3313231311222311321322321113113132312333112223323
BNL4061 13 37.9
21112331223331322323132221333233133323131313312131312231332233323131323133332323
3213333333223323321222333333113332312333112323323
JESPR165 14 0.0
31331332321323333321323113233322322313132332133123232332313131121332223222211323
23313331333133132132331322333231331322 11-31333123
BNL3502 14 5.4
31331332321323233221323313233322322333133332133123232332213131123333222232222 11323
2331233132313312213233132233323123132213333333133
TMB0803 14 12.0
31333332321322233121323313233122332333133332133123232322313131123333222222221332 3
2333233133313233213233132231323133332213332333131
NAU2336 14 26.2
13333333323332233123222313223121333333333332131123223333313131323333222322233323
2333212333313233233233133231323133321211322323133

[Trait]
Buds_number M 8.0 39.0 1.0 10.0 26.0 27.0 11.0 21.0 22.0 1.0 12.0 44.0 46.0 32.0
32.0 59.0 61.0 22.0 20.0 0.0 13.0 31.0 25.0 15.0 44.0 47.0 11.0 14.0 34.0 53.0
32.0 10.0 3.0 23.0 10.0 19.0 56.0 41.0 24.0 4.0 107.0 5.0 34.0 78.0 25.0 11.0
16.0 33.0 65.0 0.0 22.0 32.0 25.0 52.0 65.0 1.0 44.0 8.0 13.0 0.0 94.0 19.0 3.0
12.0 50.0 19.0 50.0 34.0 49.0 28.0 1.0 51.0 0.0 31.0 25.0 68.0 34.0 23.0 27.0
20.0 14.0 28.0 14.0 58.0 84.0 25.0 55.0 89.0 68.0 58.0 0.0 31.0 28.0 48.0 19.0
23.0 16.0 96.0 96.0 0.0 109.0 36.0 26.0 2.0 34.0 56.0 35.0 33.0 15.0 58.0 0.0
12.0 70.0 6.0 48.0 0.0 93.0 82.0 44.0 30.0 27.0 0.0 14.0 12.0 64.0 0.0 105.0
32.0 0.0
flowering_time M 87.0 56.0 82.0 61.0 50.0 47.0 64.0 61.0 55.0 118.0 58.0 42.0
33.0 50.0 45.0 51.0 40.0 86.0 84.0 0.0 47.0 53.0 46.0 44.0 36.0 52.0 63.0 67.0
44.0 46.0 53.0 58.0 97.0 40.0 50.0 51.0 57.0 40.0 71.0 51.0 46.0 110.0 64.0 46.0
47.0 76.0 63.0 50.0 43.0 0.0 54.0 39.0 55.0 46.0 48.0 111.0 44.0 77.0 75.0 0.0
47.0 73.0 59.0 50.0 50.0 65.0 44.0 50.0 52.0 80.0 99.0 48.0 0.0 53.0 47.0 51.0
43.0 50.0 43.0 67.0 38.0 71.0 60.0 50.0 49.0 44.0 54.0 40.0 50.0 58.0 0.0 44.0
49.0 61.0 84.0 51.0 76.0 36.0 39.0 0.0 49.0 53.0 56.0 114.0 47.0 59.0 64.0 60.0
64.0 57.0 0.0 62.0 60.0 76.0 54.0 0.0 46.0 42.0 46.0 44.0 70.0 0.0 87.0 90.0
49.0 0.0 47.0 78.0 0.0
flowering_frame M 17.0 58.0 0.0 58.0 68.0 65.0 51.0 58.0 61.0 0.0 58.0 70.0 85.0
52.0 66.0 68.0 79.0 33.0 34.0 0.0 67.0 66.0 73.0 70.0 83.0 60.0 53.0 48.0 74.0

73.0 67.0 44.0 14.0 71.0 61.0 67.0 62.0 72.0 48.0 62.0 72.0 5.0 56.0 71.0 72.0
44.0 56.0 69.0 76.0 0.0 66.0 73.0 56.0 69.0 71.0 0.0 75.0 38.0 39.0 0.0 73.0
47.0 52.0 69.0 61.0 47.0 70.0 69.0 67.0 39.0 0.0 69.0 0.0 66.0 70.0 69.0 77.0
63.0 74.0 52.0 77.0 49.0 53.0 70.0 69.0 58.0 66.0 78.0 68.0 61.0 0.0 75.0 70.0
59.0 32.0 65.0 43.0 79.0 81.0 0.0 71.0 63.0 61.0 3.0 73.0 56.0 56.0 58.0 48.0
58.0 0.0 57.0 59.0 42.0 65.0 0.0 73.0 77.0 74.0 75.0 49.0 0.0 28.0 28.0 70.0 0.0
72.0 38.0 0.0
Photop_flowering M 6.0 3.0 5.0 3.0 2.0 2.0 3.0 3.0 3.0 9.0 3.0 1.0 1.0 2.0 2.0
2.0 1.0 6.0 5.0 10.0 2.0 2.0 2.0 2.0 1.0 2.0 3.0 4.0 2.0 2.0 2.0 3.0 7.0 1.0 2.0
2.0 3.0 1.0 4.0 2.0 2.0 8.0 3.0 2.0 2.0 5.0 3.0 2.0 1.0 10.0 3.0 1.0 3.0 2.0 2.0
8.0 2.0 5.0 4.0 10.0 2.0 4.0 3.0 2.0 2.0 4.0 2.0 2.0 2.0 5.0 7.0 2.0 10.0 2.0
2.0 2.0 1.0 2.0 1.0 4.0 1.0 4.0 3.0 2.0 2.0 2.0 3.0 1.0 2.0 3.0 10.0 2.0 2.0 3.0
5.0 2.0 5.0 1.0 1.0 10.0 2.0 2.0 3.0 8.0 2.0 3.0 3.0 3.0 3.0 3.0 10.0 3.0 3.0
5.0 3.0 10.0 2.0 1.0 2.0 2.0 4.0 10.0 6.0 6.0 2.0 10.0 2.0 5.0 10.0
Height M 120.0 140.0 30.0 110.0 200.0 100.0 140.0 170.0 190.0 130.0 110.0 90.0
150.0 80.0 100.0 90.0 180.0 190.0 170.0 50.0 160.0 110.0 150.0 130.0 140.0 160.0
160.0 170.0 180.0 100.0 130.0 50.0 120.0 130.0 110.0 100.0 150.0 80.0 120.0
130.0 110.0 130.0 170.0 90.0 130.0 130.0 90.0 110.0 160.0 70.0 120.0 120.0 110.0
150.0 140.0 130.0 200.0 140.0 140.0 30.0 120.0 140.0 100.0 200.0 90.0 70.0 140.0
110.0 150.0 210.0 150.0 140.0 40.0 170.0 160.0 190.0 210.0 170.0 180.0 190.0
140.0 150.0 120.0 190.0 110.0 120.0 150.0 130.0 120.0 160.0 90.0 80.0 100.0
180.0 200.0 100.0 110.0 190.0 160.0 140.0 160.0 140.0 150.0 135.0 135.0 120.0
100.0 160.0 110.0 80.0 80.0 200.0 110.0 180.0 150.0 60.0 170.0 130.0 150.0 110.0
160.0 130.0 150.0 160.0 150.0 120.0 120.0 145.0 110.0
hs M 17.0 7.0 ? 10.0 10.0 ? 10.0 13.0 8.0 27.0 8.0 7.0 6.0 7.0 5.0 6.0 5.0 25.0
8.0 ? 7.0 10.0 7.0 10.0 5.0 10.0 10.0 10.0 6.0 6.0 10.0 ? 17.0 7.0 7.0 10.0 8.0
5.0 10.0 ? 6.0 13.0 ? 6.0 ? ? 5.0 8.0 ? ? 10.0 5.0 5.0 7.0 ? 9.0 6.0 14.0 10.0 ?
9.0 9.0 11.0 6.0 6.0 9.0 6.0 6.0 7.0 7.0 19.0 9.0 ? ? 9.0 10.0 ? 10.0 8.0 10.0
5.0 ? 14.0 10.0 ? ? ? 7.0 10.0 ? 17.0 8.0 6.0 8.0 ? 9.0 13.0 7.0 10.0 ? ? 6.0
13.0 27.0 6.0 12.0 13.0 8.0 12.0 ? ? ? 8.0 10.0 9.0 ? 6.0 6.0 8.0 6.0 9.0 29.0 ?
9.0 8.0 28.0 6.0 10.0 ?
Monopod M 10.0 5.0 ? 6.0 0.0 ? 4.0 4.0 4.0 4.0 0.0 3.0 0.0 0.0 2.0 5.0 3.0 6.0
5.0 ? 1.0 5.0 0.0 4.0 4.0 5.0 0.0 7.0 1.0 3.0 6.0 ? 2.0 0.0 3.0 4.0 2.0 2.0 6.0
? 4.0 7.0 ? 6.0 ? ? 2.0 2.0 ? ? 2.0 3.0 0.0 5.0 ? 2.0 0.0 9.0 5.0 ? 7.0 3.0 5.0
0.0 3.0 2.0 3.0 1.0 5.0 2.0 6.0 3.0 ? ? 3.0 5.0 ? 4.0 1.0 3.0 1.0 ? 7.0 7.0 ? ?
? 4.0 6.0 ? 0.0 6.0 1.0 8.0 ? 4.0 1.0 3.0 11.0 ? ? 1.0 6.0 24.0 3.0 10.0 8.0 4.0
3.0 ? ? ? 5.0 7.0 7.0 ? 3.0 6.0 3.0 1.0 4.0 3.0 ? 5.0 4.0 24.0 4.0 3.0 ?
Simpod M 10.0 36.0 ? 18.0 36.0 ? 26.0 22.0 36.0 3.0 26.0 16.0 36.0 22.0 24.0
14.0 40.0 22.0 26.0 ? 36.0 24.0 30.0 24.0 36.0 30.0 26.0 30.0 32.0 26.0 20.0 ?
10.0 28.0 28.0 24.0 30.0 16.0 26.0 ? 24.0 18.0 ? 18.0 ? ? 22.0 21.0 ? ? 26.0
22.0 26.0 26.0 ? 14.0 36.0 22.0 24.0 ? 22.0 24.0 16.0 34.0 21.0 12.0 41.0 20.0
28.0 40.0 16.0 28.0 ? ? 38.0 32.0 ? 28.0 36.0 32.0 12.0 ? 12.0 30.0 ? ? ? 28.0
26.0 ? 7.0 20.0 22.0 26.0 ? 21.0 16.0 27.0 24.0 ? ? 34.0 18.0 6.0 34.0 14.0 16.0
28.0 21.0 ? ? ? 21.0 30.0 24.0 ? 32.0 20.0 32.0 24.0 28.0 3.0 ? 26.0 26.0 7.0
30.0 32.0 ?
No_Nodes M 26.0 42.0 ? ? 45.0 ? 35.0 34.0 43.0 29.0 33.0 22.0 41.0 28.0 28.0
19.0 44.0 46.0 33.0 ? 42.0 33.0 36.0 33.0 40.0 39.0 35.0 39.0 37.0 31.0 29.0 ?
26.0 34.0 34.0 33.0 37.0 20.0 35.0 ? 29.0 30.0 ? 23.0 ? ? 26.0 28.0 ? ? 35.0
26.0 30.0 32.0 ? 22.0 41.0 35.0 33.0 ? 30.0 32.0 26.0 39.0 26.0 20.0 46.0 25.0
34.0 46.0 34.0 36.0 ? ? 46.0 41.0 ? 37.0 43.0 41.0 16.0 ? 25.0 39.0 ? ? ? 34.0
35.0 ? 23.0 27.0 27.0 33.0 ? 29.0 28.0 33.0 33.0 ? ? 39.0 30.0 32.0 39.0 25.0
28.0 35.0 32.0 ? ? ? 28.0 39.0 32.0 ? 37.0 25.0 39.0 29.0 36.0 31.0 ? 34.0 33.0
34.0 35.0 41.0 ?
No_Bolls M 2.0 3.0 0.0 0.0 28.0 5.0 7.0 8.0 8.0 0.0 1.0 18.0 14.0 10.0 8.0 12.0
0.0 5.0 6.0 0.0 8.0 12.0 4.0 3.0 10.0 26.0 2.0 10.0 28.0 12.0 14.0 2.0 3.0 12.0
2.0 8.0 24.0 5.0 0.0 0.0 47.0 0.0 12.0 24.0 10.0 0.0 10.0 14.0 21.0 0.0 18.0 7.0
21.0 22.0 6.0 0.0 28.0 0.0 4.0 0.0 34.0 0.0 1.0 4.0 24.0 12.0 22.0 17.0 24.0
20.0 3.0 14.0 0.0 10.0 8.0 10.0 5.0 5.0 14.0 1.0 7.0 3.0 6.0 18.0 6.0 2.0 28.0
36.0 26.0 16.0 1.0 4.0 5.0 17.0 0.0 10.0 8.0 34.0 30.0 0.0 6.0 16.0 2.0 0.0 15.0
18.0 18.0 22.0 8.0 0.0 0.0 0.0 26.0 4.0 26.0 0.0 36.0 20.0 26.0 13.0 18.0 0.0
0.0 7.0 22.0 0.0 52.0 22.0 0.0
No_Open_Bolls M 0.0 1.0 0.0 0.0 1.0 2.0 0.0 0.0 0.0 0.0 0.0 2.0 0.0 2.0 1.0 5.0
0.0 0.0 0.0 0.0 1.0 0.0 0.0 0.0 1.0 0.0 0.0 0.0 0.0 0.0 0.0 0.0 0.0 2.0 0.0 2.0
0.0 2.0 0.0 0.0 6.0 0.0 0.0 2.0 0.0 0.0 0.0 0.0 1.0 0.0 2.0 1.0 0.0 1.0 0.0 0.0
6.0 0.0 0.0 0.0 1.0 0.0 0.0 1.0 2.0 1.0 2.0 2.0 2.0 0.0 0.0 2.0 0.0 0.0 0.0 0.0
1.0 1.0 1.0 0.0 4.0 0.0 0.0 0.0 0.0 0.0 0.0 3.0 1.0 0.0 0.0 0.0 3.0 1.0 0.0 1.0
0.0 7.0 0.0 0.0 0.0 4.0 0.0 0.0 0.0 0.0 0.0 0.0 0.0 0.0 0.0 0.0 0.0 0.0 0.0 0.0
4.0 0.0 1.0 1.0 0.0 0.0 0.0 0.0 5.0 0.0 5.0 0.0 0.0

Anthocyan M 2.0 2.0 ? 1.0 3.0 2.0 3.0 3.0 3.0 1.0 1.0 2.0 1.0 1.0 1.0 2.0 3.0
2.0 1.0 1.0 2.0 2.0 3.0 2.0 3.0 2.0 3.0 2.0 3.0 3.0 1.0 1.0 2.0 2.0 1.0 1.0 3.0
2.0 3.0 2.0 3.0 1.0 ? 3.0 3.0 3.0 2.0 3.0 3.0 ? 3.0 2.0 3.0 3.0 3.0 1.0 3.0 3.0
3.0 ? 2.0 2.0 1.0 3.0 3.0 2.0 2.0 3.0 2.0 3.0 2.0 2.0 ? 3.0 3.0 2.0 2.0 3.0 3.0
3.0 3.0 3.0 2.0 3.0 2.0 2.0 3.0 2.0 3.0 3.0 2.0 3.0 3.0 3.0 ? 2.0 2.0 3.0 3.0
3.0 3.0 3.0 2.0 3.0 2.0 2.0 2.0 2.0 3.0 3.0 3.0 3.0 2.0 3.0 3.0 ? 2.0 3.0 2.0
3.0 3.0 3.0 3.0 3.0 3.0 3.0 3.0 3.0 3.0
Stem_hair M 3.0 3.0 ? 1.0 1.0 1.0 ? 1.0 1.0 2.0 1.0 2.0 2.0 1.0 1.0 1.0 1.0 1.0
1.0 1.0 4.0 2.0 1.0 1.0 1.0 4.0 1.0 4.0 1.0 1.0 1.0 1.0 1.0 1.0 1.0 1.0 1.0 1.0
1.0 1.0 1.0 1.0 ? 1.0 1.0 1.0 1.0 1.0 1.0 ? 1.0 1.0 1.0 2.0 1.0 1.0 1.0 2.0 1.0
? 1.0 1.0 1.0 1.0 3.0 1.0 2.0 1.0 3.0 1.0 2.0 1.0 ? 1.0 3.0 3.0 3.0 1.0 1.0 1.0
1.0 1.0 3.0 1.0 3.0 3.0 2.0 1.0 1.0 3.0 1.0 1.0 1.0 1.0 ? 3.0 2.0 2.0 1.0 4.0
1.0 1.0 2.0 1.0 1.0 4.0 1.0 1.0 1.0 1.0 1.0 1.0 1.0 1.0 1.0 ? 1.0 1.0 3.0 1.0
1.0 1.0 3.0 1.0 1.0 1.0 3.0 1.0 1.0
