# Supplementary material for: QTL mapping for flowering-time and photoperiod insensitivity of cotton Gossypium darwinii Watt
Source: PLoS One. 2017 Oct 9;12(10):e0186240. doi: 10.1371/journal.pone.0186240 (PMC5633191; doi:10.1371/journal.pone.0186240)
Supplement: S2 Data — (PDF) [file pone.0186240.s002.pdf]

#FileID 20170429180955
#bychromosome
-type position
-function 1
-Units cM
-chromosomes 25
-maximum 20
-named yes
-case yes
-start
-Chromosome 01
TMB1421 0.0
JESPR289 7.3
TMB0062 16.3
BNL3888 27.8
CM92 29.9
BNL3580 34.6
GH75 53.6
-Chromosome 02
GH198 0.0
TMB0471 0.3
BNL3971 1.9
JESPR179 22.6
-Chromosome 03
BNL1379 0.0
BNL3441 7.7
CM106 8.5
TMB0564 8.9
TMB1989 32.8
-Chromosome 04
TMB0809 0.0
GH117 11.8
BNL2572 29.8
-Chromosome 05
CIR373 0.0
NAU3212 11.7
NAU5015 17.4
NAU5160 23.9
NAU3325 26.3
NAU3014 43.0
NAU861 45.3
BNL3992 59.1
GH83 60.9
BNL542 73.2
JESPR241 82.6
NAU2140 88.2
GH211 93.9
NAU2001 100.2
NAU2296 101.4
TMB0191 101.4
NAU3569 101.7
NAU5149 103.5
BNL3995 104.7
JESPR65 112.6
-Chromosome 06
GH82 0.0
GH39 34.0

GH32 36.0
TMB0154 36.9
TMB1538 36.9
JESPR119 55.3
-Chromosome 09
GH98 0.0
GH27 2.0
GH112 2.0
TMB0184 8.9
-Chromosome 09a
GH118 0.0
BNL1414 17.2
BNL4028 22.1
-Chromosome 10
PhyB 0.0
BNL2872 4.0
TMB0325 4.3
TMB0307 4.4
BNL2705 4.4
TMB0380 11.6
CM67 14.4
BNL2960 15.4
BNL1665 25.7
TMB1745 29.1
-Chromosome 11
BNL1066 0.0
JESPR296 17.3
PhyA 19.7
NAU1014 31.1
TMB0064 32.3
BNL625 36.8
TMB0359 41.0
-Chromosome 12
TMB0799 0.0
JESPR270 27.0
NAU1237 31.5
BNL3835 32.3
NAU1278 40.6
BNL1679 64.6
CIR148 65.3
NAU5047 75.6
CM85 82.8
-Chromosome 13
BNL1495 0.0
BNL1421 0.0
BNL3623 7.8
GH215 10.3
GH34 24.0
BNL4061 37.9
-Chromosome 14
JESPR165 0.0
BNL3502 5.4
TMB0803 12.0
NAU2336 26.2
-Chromosome 15
JESPR152 0.0
TMB1664 22.3

TMB1660 25.7
BNL2920 25.8
TMB0201 28.0
TMB0301 36.7
JESPR180 36.7
JESPR298 36.7
BNL4082 40.1
TMB0375 42.2
BNL3902 42.5
BNL1350 47.8
TMB1181 59.6
BNL786 60.0
-Chromosome 16
BNL3065 0.0
GH2 29.6
JESPR128 30.3
JESPR32 30.3
TMB1271 30.3
JESPR237 30.6
JESPR297 32.2
BNL2734 36.1
BNL3008 36.1
TMB1409 37.3
-Chromosome 17
JESPR195 0.0
BNL4003 6.1
BNL3955 7.1
TMB2018 8.8
-Chromosome 18
BNL3558 0.0
BNL1079 10.9
BNL2667 40.4
-Chromosome 19
TMB1599 0.0
GH71 1.5
BNL285 12.2
NAU3935 23.1
BNL852 29.0
TMB0189 30.5
GH109 31.3
TMB0366 39.6
BNL4096 40.9
BNL3875 47.4
TMB1489 60.4
BNL3977 63.9
TMB1645 88.0
CM209 89.2
JESPR218 100.8
CM42 101.2
CM3 101.2
JESPR236 101.6
-Chromosome 20
CM82 0.0
JESPR235 0.0
BNL169 1.9
GH48 2.8
BNL119 4.6

TMB1629 9.8
GH59 12.3
GH54 15.7
BNL3948 19.3
TMB1630 46.2
-Chromosome 21
JESPR158 0.0
CM23 14.4
TMB2038 30.2
TMB0400 42.7
JESPR118 42.7
BNL3649 50.8
BNL1551 53.0
BNL3279 88.5
-Chromosome 23
BNL686 0.0
TMB1701 28.9
JESPR151 30.1
TMB1425 51.3
TMB0382 65.6
JESPR110 75.5
-Chromosome 24
TMB0429 0.0
BNL2568 11.5
GH171 13.7
BNL2655 14.2
GH272 15.9
BNL1521 22.3
BNL2616 23.4
BNL252 28.5
-Chromosome 25
GH224 0.0
JESPR215 0.7
JESPR227 0.7
CM27 0.7
CM13 0.7
NAU3171 23.5
-Chromosome 26
NAU4925 0.0
NAU1119 27.7
BNL3510 38.7
BNL3816 38.8
CIR391 39.1
BNL840 39.1
CIR039 39.1
JESPR92 39.1
NAU2195 39.4
NAU3006 40.0
NAU2913 70.3
BNL341 76.3
NAU2750 80.9
NAU4914 101.8
-Chromosome 26a
BNL3994 0.0
TMB0120 3.6
GH52 3.7
GH200 20.2

```
-stop


#bycross
-SampleSize 129
-Cross SF2
-traits 13
-missingtrait ?
-case yes
-TranslationTable
AA  2 2
Aa  1 1
aa  0 0
A- 12 4
a- 10 3
-- -1 5
-start markers
TMB1421 1 2 0 1 1 1 1 2 0 1 1 1 0 1 0 0 2 2 1 1 1 0 1 0 1 1 1
2 2 0 1 1 0 1 2 1 0 1 1 0 1 1 1 0 1 2 0 1 1 1 1 1 1 1 2 0 0 1
0 0 2 1 0 0 0 1 2 0 1 0 1 2 0 0 1 2 0 0 2 0 1 1 0 2 2 2 1 1 0
0 0 0 1 1 1 1 0 2 0 1 2 2 1 1 1 0 0 1 0 0 2 2 1 1 1 2 1 0 1 1
1 1 2 2 2 1 1 2 2
JESPR289 1 2 0 1 1 1 1 2 0 2 0 0 0 1 0 0 2 2 1 1 1 0 1 0 1 1 1
2 1 0 1 1 0 1 2 2 0 1 2 0 0 1 1 0 1 1 1 1 1 1 1 1 1 1 2 0 0 1
2 0 2 1 0 0 5 1 2 0 1 0 1 2 0 0 1 1 0 0 2 0 1 1 0 2 2 2 1 1 1
2 0 0 1 2 1 2 0 2 0 1 2 2 1 1 2 0 0 1 0 0 2 1 1 1 1 2 5 0 1 1
1 1 2 2 2 1 1 2 2
TMB0062 0 2 0 1 1 1 1 2 0 2 1 0 1 0 0 0 1 2 1 1 1 0 1 0 1 1 1
2 1 0 0 1 0 1 2 0 0 1 2 0 0 1 1 0 1 1 1 1 1 1 2 1 1 1 2 0 0 1
2 0 2 1 0 0 0 0 2 0 1 0 1 1 0 1 1 1 0 0 2 0 1 1 0 2 2 1 1 1 1
2 0 1 1 2 1 1 0 2 0 1 2 2 1 1 2 0 0 2 0 1 2 1 0 1 1 1 1 0 1 1
1 1 2 1 1 1 1 1 2
BNL3888 0 2 0 1 0 1 1 2 0 2 1 0 1 1 0 0 1 2 1 0 1 0 1 1 0 0 1
1 2 0 0 1 0 1 2 0 0 1 2 1 1 2 0 1 1 1 1 1 1 1 2 1 0 1 2 0 1 1
2 0 2 1 0 0 0 0 1 0 1 0 1 1 0 1 0 1 2 0 2 0 1 1 0 2 2 1 1 1 1
2 1 1 1 2 1 1 0 2 1 1 1 1 1 1 2 0 0 2 0 1 1 1 0 1 1 0 1 0 1 1
0 1 2 1 1 1 1 1 2
CM92 0 2 0 1 0 1 1 2 0 2 1 0 1 1 0 0 1 2 1 0 1 0 1 1 0 0 1 1 2
0 0 1 0 1 2 0 0 1 2 1 1 2 0 1 1 1 1 1 1 1 2 1 0 1 2 0 1 2 2 0
2 0 0 0 0 0 1 0 1 0 0 1 0 1 0 1 2 0 2 0 1 1 0 2 2 1 1 1 1 2 1
1 1 2 5 1 0 2 1 0 1 1 1 1 2 0 0 2 0 1 1 1 0 1 1 0 1 0 1 1 0 1
1 1 1 1 1 1 1
BNL3580 0 2 0 1 0 1 1 2 0 2 1 0 1 1 0 0 1 2 1 0 1 0 1 1 0 0 0
1 2 1 0 1 0 1 2 0 0 1 2 1 1 2 0 1 1 1 1 2 1 2 2 1 0 1 2 0 1 2
1 0 2 1 0 0 0 1 1 0 1 0 1 0 0 1 0 1 2 1 2 0 1 1 0 2 2 1 1 1 1
2 1 1 1 2 1 1 0 2 1 0 1 1 1 1 2 0 0 2 1 1 1 1 0 1 1 0 1 1 0 1
0 1 1 1 1 1 1 1 1
GH75 1 1 0 1 0 1 2 2 1 2 1 1 1 1 0 1 1 2 0 0 1 0 1 2 0 1 0 0 2
1 0 1 1 1 2 0 1 0 2 1 1 2 0 1 1 1 2 2 1 2 1 1 1 1 2 1 1 1 1 0
2 1 0 0 2 0 1 0 0 0 1 0 0 2 0 1 2 1 1 0 2 2 0 2 2 1 1 1 1 1 1
0 1 0 1 1 0 2 1 0 0 2 2 1 2 1 1 2 1 2 1 0 0 2 2 0 1 2 0 1 0 1
1 1 1 1 2 0 1
GH198 2 2 1 1 2 0 0 2 2 1 1 1 0 0 1 1 1 1 0 2 1 2 1 1 2 1 1 1
2 1 0 1 1 1 2 2 1 1 1 0 0 0 1 1 1 1 1 2 0 0 2 0 0 2 1 1 0 1 1
0 1 0 2 1 1 1 0 2 2 2 2 1 0 1 0 1 1 1 2 1 2 0 2 0 1 1 1 1 1 1
0 1 1 1 2 1 2 1 2 1 0 0 1 2 1 1 1 1 0 0 0 1 1 0 2 1 1 0 1 1 2
```

2 0 1 2 0 2 1 0
TMB0471 2 2 2 1 2 0 0 2 2 1 1 1 0 0 1 1 1 1 0 2 1 2 1 1 2 1 1
1 2 1 0 1 1 1 2 2 1 1 1 0 0 0 1 1 1 1 1 2 0 0 2 0 0 2 1 1 0 1
1 0 0 0 2 1 1 1 0 2 2 2 2 1 0 1 0 1 1 1 2 1 2 0 2 0 1 1 1 1 1
1 0 1 1 1 2 1 2 1 2 1 0 0 1 2 1 1 1 1 0 0 0 1 1 0 2 2 1 0 1 1
2 1 0 1 2 0 2 1 1
BNL3971 2 2 2 1 2 0 0 2 2 1 1 1 0 0 1 1 1 1 0 2 1 2 1 1 2 1 1
1 2 1 0 1 1 1 2 2 1 1 1 0 0 0 1 1 1 1 1 2 0 0 2 0 0 2 1 1 0 1
1 0 0 0 2 1 1 1 0 2 2 2 2 1 0 1 0 1 1 1 2 1 2 0 2 0 1 1 1 1 1
1 0 1 1 1 2 1 2 1 2 1 0 0 1 2 1 1 1 1 0 0 0 1 1 0 2 2 1 0 1 1
2 1 0 1 2 0 2 1 1
JESPR179 1 2 2 1 2 0 1 2 2 2 1 1 0 0 0 1 2 1 0 2 1 2 2 1 2 2 1
1 1 2 0 2 1 1 1 1 2 1 1 1 1 0 0 1 1 1 1 2 0 1 2 0 0 1 1 1 0 1
0 1 0 1 2 2 1 1 0 1 1 2 2 1 2 0 1 1 1 1 1 1 2 0 2 0 1 1 1 1 1
0 0 1 0 0 2 1 2 2 2 1 0 0 2 2 0 2 0 2 0 0 0 1 1 2 1 2 1 0 1 2
2 1 0 2 2 1 2 1 2
BNL1379 1 1 1 1 0 1 1 2 0 1 2 0 0 0 1 0 0 1 1 1 1 2 1 0 1 1 2
2 0 2 0 2 0 1 2 2 1 0 1 2 1 1 2 2 2 1 0 0 2 2 1 2 0 1 0 0 0 0
2 1 1 2 1 2 0 0 1 1 1 2 2 1 0 2 0 2 1 0 2 1 2 0 1 2 0 0 1 1 0
1 1 2 0 0 0 1 0 2 0 1 0 0 1 1 1 0 1 2 1 0 2 2 1 1 1 1 1 2 0 1
1 0 0 2 2 2 0 0 1
BNL3441 1 1 2 1 0 1 1 2 1 2 1 0 0 0 1 0 1 2 1 1 1 2 1 0 1 1 2
2 0 2 1 2 0 1 2 2 1 0 1 2 1 1 2 2 2 1 0 0 2 2 1 2 0 2 0 1 0 0
2 1 1 1 1 2 1 0 1 1 1 2 2 1 0 2 0 2 1 0 2 1 2 0 1 2 1 0 1 1 1
1 1 1 0 1 1 1 0 2 0 1 0 0 1 1 1 1 1 2 1 0 2 2 0 1 1 1 1 2 0 1
1 0 0 1 2 2 0 0 1
CM106 1 1 2 1 0 1 1 2 1 2 1 0 0 0 1 0 1 2 1 0 1 2 1 0 1 1 2 2
0 2 2 2 0 1 2 2 1 0 1 2 1 1 2 2 2 1 0 0 2 2 1 2 0 2 0 1 0 0 2
1 1 1 1 2 1 0 1 1 1 2 2 1 0 2 0 2 1 0 2 1 2 0 1 2 1 0 1 1 1 1
1 1 0 1 1 1 0 2 0 1 0 0 1 1 1 1 1 2 1 0 2 2 0 1 1 1 1 2 0 1 1
0 0 1 2 2 0 0 1
TMB0564 1 1 2 1 0 1 1 2 1 2 1 1 0 0 1 0 1 2 1 0 1 2 1 0 1 1 2
2 0 2 2 2 0 1 2 2 1 0 5 2 1 1 2 2 2 1 0 0 2 2 1 2 0 2 0 1 0 0
2 1 1 1 5 2 1 0 1 1 1 2 2 1 0 2 0 2 1 0 2 1 2 0 1 2 1 0 1 1 1
1 1 1 0 1 1 1 0 2 0 1 0 0 1 1 1 1 1 2 1 0 2 2 0 1 1 1 1 2 0 1
1 0 0 1 2 2 0 0 1
TMB1989 2 1 2 2 1 0 1 2 0 2 1 0 1 0 1 0 1 2 1 1 2 2 1 0 0 1 1
1 0 2 1 1 0 1 2 2 1 1 1 2 1 0 2 1 1 1 0 0 1 2 1 2 1 1 1 1 0 0
1 0 0 1 1 2 1 0 1 1 1 1 2 1 2 2 0 1 2 0 2 1 2 1 0 1 1 0 1 1 1
1 1 1 1 1 1 0 1 1 1 1 0 0 1 2 0 1 2 2 0 0 2 2 1 1 0 1 2 1 0 2
1 2 1 1 2 1 1 1 0
TMB0809 1 2 1 0 1 2 1 2 2 2 1 2 1 0 0 0 0 0 0 2 2 1 1 0 0 1 1
1 1 1 0 1 1 2 1 1 1 0 2 1 1 2 2 1 0 2 2 1 1 0 2 1 2 1 0 1 2 1
0 2 1 1 2 2 0 1 1 1 0 1 1 1 1 0 0 1 2 2 1 1 0 1 2 2 1 2 1 0 1
1 1 2 1 1 1 2 0 1 0 1 2 2 1 2 0 2 0 2 1 1 2 1 0 1 1 1 1 1 2 0
0 1 2 1 1 2 0 2 2
GH117 1 1 2 0 1 2 1 1 2 2 1 2 1 0 0 1 0 0 0 2 2 1 1 2 0 1 1 1
1 0 0 1 1 2 1 2 1 0 2 1 1 2 2 1 1 1 2 1 1 0 2 0 2 1 0 1 2 1 0
1 1 1 2 2 0 1 1 1 0 1 1 1 2 0 0 1 1 2 0 1 0 1 2 2 1 2 0 1 1 1
1 2 1 0 1 2 0 1 1 1 2 1 1 2 0 2 0 2 0 1 2 1 0 2 1 0 1 1 1 2 0
1 2 0 2 2 0 2 2
BNL2572 1 1 1 0 1 1 1 1 2 2 1 1 1 1 1 1 0 1 0 2 1 1 1 2 0 1 1
1 1 0 1 1 2 2 0 2 1 1 2 1 1 2 2 1 1 1 0 2 2 0 1 0 1 1 1 1 1 1
0 1 1 2 2 2 0 1 1 2 0 0 1 1 1 1 0 2 0 2 0 1 0 1 1 1 1 2 0 1 1
1 2 2 1 0 1 0 0 1 1 1 2 1 0 2 0 1 0 2 0 1 2 1 0 2 1 0 1 1 0 2
1 2 1 0 2 1 1 1 5
JESPR65 1 1 1 1 1 1 2 1 1 1 0 0 1 2 1 1 1 1 1 1 1 2 1 2 2 2 1

0 1 0 0 1 2 1 1 1 1 2 1 1 2 0 1 2 1 2 0 2 1 0 1 0 1 1 0 1 2 2
1 1 1 1 0 1 0 2 1 1 0 1 2 1 1 0 2 1 1 0 1 1 2 1 0 2 2 2 0 2 2
1 2 0 1 1 2 0 2 1 2 0 0 0 2 1 1 1 0 1 2 0 1 2 0 1 1 0 1 0 1 0
1 2 0 1 2 1 2 2 1
BNL3995 1 1 1 1 1 1 2 1 1 1 2 0 1 1 1 1 1 1 1 1 1 2 1 2 1 2 1
0 2 0 0 1 2 1 1 1 1 2 1 1 2 0 1 2 1 2 0 1 1 0 1 0 1 1 0 2 2 2
1 1 1 1 0 1 1 2 2 0 0 1 2 1 1 0 2 1 2 0 0 1 2 0 0 1 2 1 1 2 1
1 2 1 1 1 1 0 2 1 2 0 0 0 2 1 1 1 0 2 2 0 2 1 1 1 1 0 1 1 1 0
1 2 0 1 0 1 2 2 1
NAU5149 1 1 1 1 1 1 2 1 1 1 2 0 1 1 1 1 1 1 1 1 1 2 1 2 1 2 1
0 2 0 0 1 2 1 1 1 1 2 1 1 2 0 1 2 1 2 0 1 1 0 1 0 1 1 0 2 2 2
1 1 1 1 0 1 1 2 2 0 0 1 2 1 1 0 2 1 2 0 0 1 2 0 0 2 2 1 1 2 1
1 2 1 1 1 1 0 2 1 2 0 0 0 2 1 1 1 0 2 2 0 1 2 0 1 1 0 1 1 1 0
1 2 0 1 2 1 2 2 1
NAU3569 2 1 1 1 1 1 2 1 1 1 2 0 2 1 1 1 1 1 1 1 1 2 1 2 1 2 1
0 2 0 1 1 2 1 1 1 1 2 1 1 2 0 1 2 1 2 0 1 1 0 1 0 1 1 0 2 2 2
1 1 1 1 0 1 1 2 2 0 0 1 2 1 1 0 2 1 2 0 0 1 2 0 0 1 2 1 1 5 1
1 2 1 1 1 1 0 2 1 2 0 0 0 2 1 1 1 0 2 2 0 2 2 0 1 1 0 1 1 1 0
1 2 0 1 2 1 2 2 1
TMB0191 2 1 1 1 1 1 2 1 1 1 2 0 2 1 1 1 1 1 1 1 1 2 1 2 1 2 5
0 2 0 1 1 2 1 1 1 1 2 1 1 2 0 1 2 1 2 0 1 1 0 1 0 1 1 0 2 2 2
1 1 1 1 0 1 1 2 2 0 0 1 2 1 1 0 2 1 2 0 0 1 2 0 0 1 2 1 1 2 1
1 2 1 1 1 1 0 2 1 2 0 0 0 2 1 1 1 0 2 2 0 2 2 0 1 1 0 1 1 1 0
1 2 1 1 2 1 2 2 1
NAU2296 2 1 1 1 1 1 2 1 1 1 2 0 2 1 1 1 1 1 1 1 1 2 1 2 1 2 1
0 2 0 1 1 2 1 1 1 1 2 1 1 2 0 1 2 1 2 0 1 1 0 1 0 1 1 0 2 2 2
1 1 1 1 0 1 1 2 2 0 0 1 2 1 1 0 2 1 2 0 0 1 2 0 0 1 2 1 1 2 1
1 2 1 1 1 1 0 2 1 2 0 0 0 2 1 5 1 0 2 2 0 2 2 0 1 1 0 1 1 1 0
1 2 1 1 2 1 2 2 1
NAU2001 2 1 1 1 1 1 2 1 1 1 2 0 2 1 1 1 1 1 1 1 1 2 1 2 1 2 1
0 2 0 1 1 2 1 1 1 1 2 1 1 2 0 1 2 1 2 0 0 0 0 1 0 1 1 0 2 2 2
1 1 1 1 0 1 1 2 2 0 0 1 2 1 1 0 2 1 2 0 0 1 2 0 0 1 2 1 1 2 1
1 2 1 1 1 1 0 2 1 2 0 0 0 2 1 5 1 0 2 2 0 2 2 0 2 1 0 1 1 1 0
1 2 1 1 2 1 2 2 1
GH211 1 1 1 1 1 1 2 1 1 1 2 0 2 1 1 1 1 1 1 1 1 0 1 2 0 2 1 0
2 0 1 1 2 1 1 1 1 1 2 1 1 2 1 1 2 1 2 1 0 0 0 1 0 1 1 0 2 1 2 1
0 1 1 0 1 1 2 2 0 0 1 2 0 2 0 2 1 2 0 0 1 2 0 0 1 2 1 1 1 1 1
2 1 1 1 1 1 1 1 2 0 0 0 2 1 1 0 0 1 2 0 2 2 0 2 1 0 1 1 1 0 1
2 1 1 2 1 2 2 2
NAU2140 1 1 1 1 1 1 2 1 1 1 2 0 2 1 1 1 1 1 1 1 1 0 1 2 0 1 1
0 1 0 1 1 2 1 1 1 1 2 1 1 2 1 1 1 2 2 1 0 0 0 1 0 1 1 0 2 1 2
1 1 1 1 0 1 1 1 2 0 0 1 2 0 2 1 2 5 2 0 1 1 2 0 1 1 2 1 1 1 1
1 2 1 1 1 1 1 1 1 2 1 0 0 2 0 1 0 0 1 1 0 2 2 0 2 1 0 1 1 1 0
1 2 1 1 2 1 5 1 0
JESPR241 1 1 0 1 1 1 2 1 1 1 2 0 2 1 1 1 1 1 1 1 1 0 2 5 5 1 1
0 1 0 1 1 2 1 1 1 1 1 1 1 1 1 1 1 2 2 1 0 0 0 1 0 1 1 0 2 1 2
1 1 1 1 1 1 0 1 2 0 0 1 2 0 2 1 2 1 1 0 1 2 2 0 1 2 2 1 1 1 1
0 2 1 1 5 5 1 1 1 2 1 0 0 2 0 1 0 1 1 1 0 2 2 0 2 1 0 1 1 1 0
1 2 1 1 2 1 2 0 2
BNL542 1 5 0 1 1 1 2 1 1 0 2 0 1 1 0 1 1 1 1 1 1 0 2 2 0 1 1 0
1 0 1 1 2 0 1 1 1 1 1 1 1 1 1 1 1 2 2 1 0 0 0 1 0 1 1 0 2 1 2 2
1 1 1 1 1 0 1 2 0 0 1 2 0 2 1 1 1 1 2 1 0 0 2 1 0 0 1 1 1 1 2
0 1 1 1 1 1 1 1 2 1 0 0 2 0 0 0 1 0 1 0 1 1 1 2 2 1 1 1 1 0 1
2 1 1 2 1 2 0 2
GH83 1 2 0 1 1 1 1 1 1 1 2 0 0 0 0 1 1 1 1 1 1 0 2 2 0 1 2 0 1
0 1 1 1 0 1 1 0 1 1 1 2 1 1 1 0 1 1 0 1 0 1 0 1 0 1 2 1 2 1 1
1 1 1 1 0 1 2 0 1 0 2 0 2 1 1 1 1 0 1 1 2 0 1 2 2 1 2 0 1 0 2

1 1 1 1 2 1 1 2 1 1 0 2 0 0 1 1 0 1 0 1 1 1 2 2 1 1 2 1 0 1 2
1 0 1 2 0 0 2
BNL3992 1 2 0 1 1 1 2 1 1 1 2 0 0 0 0 1 1 1 1 1 1 0 2 2 0 1 2
0 1 0 1 1 1 0 1 1 0 1 1 1 2 1 1 1 2 1 1 0 1 0 1 0 1 0 1 2 1 2
1 1 1 1 1 1 0 1 2 0 1 0 2 0 2 1 1 1 1 0 1 1 2 0 1 2 2 1 2 0 1
0 2 1 1 1 1 2 1 1 2 1 2 0 2 0 0 1 1 0 1 0 1 1 1 2 2 1 1 2 1 0
1 2 1 0 0 2 0 0 2
NAU861 1 2 0 2 1 1 2 1 2 1 2 0 5 0 0 1 2 1 1 1 1 1 2 0 1 2 2 0
2 0 1 1 1 1 0 1 0 1 2 1 2 1 0 2 2 1 1 0 1 0 1 0 1 1 2 2 2 2 1
1 1 1 1 2 0 1 2 1 1 1 2 0 1 1 1 2 1 0 5 1 2 2 1 2 2 1 2 0 2 0
1 1 1 1 0 2 1 2 2 2 2 1 2 0 1 1 1 0 1 1 1 1 1 2 2 1 0 2 2 0 2
1 1 0 0 2 0 0 2
NAU3014 1 2 0 2 1 1 2 1 2 1 2 0 0 0 0 1 2 1 1 1 1 1 1 2 0 1 1 1
0 1 0 1 1 1 1 0 1 0 1 2 1 2 1 0 1 1 1 1 0 1 0 1 0 1 1 2 2 2 2
1 1 1 1 1 2 0 1 2 1 1 1 2 0 1 1 1 2 1 0 1 1 2 2 1 2 2 1 2 0 2
0 1 1 1 1 0 2 1 1 2 1 2 1 2 0 1 1 1 0 1 1 1 1 1 2 2 1 0 2 2 0
2 1 1 0 0 2 0 0 2
NAU3325 1 2 0 2 1 1 1 1 2 1 1 0 0 1 1 1 2 0 1 1 2 0 2 0 2 0 1
1 1 0 1 1 0 1 1 1 0 0 2 2 2 1 0 0 1 2 1 0 1 0 1 1 1 1 2 2 2 1
1 1 1 1 1 2 0 1 2 1 1 1 2 0 1 2 0 2 2 2 2 1 1 2 1 2 2 2 2 2 2
0 1 2 1 1 1 2 1 1 2 1 1 1 2 0 1 1 1 0 0 2 2 1 1 1 1 1 0 2 2 0
2 1 1 0 0 2 0 1 1
NAU5160 2 2 1 2 1 1 1 1 2 1 1 0 0 1 1 1 2 1 1 1 2 0 2 0 2 0 1
1 1 0 1 1 0 1 1 1 0 0 2 2 2 1 0 0 1 2 1 1 1 0 1 1 1 1 2 2 2 1
1 1 1 1 1 2 0 1 2 1 1 1 2 1 1 2 0 2 2 2 2 1 1 2 1 2 2 2 2 1 2
0 1 2 1 1 1 2 1 1 2 1 1 1 2 0 1 1 1 0 0 2 2 1 1 1 1 1 0 2 2 0
2 1 1 0 0 2 0 1 1
NAU5015 2 2 2 2 1 0 1 0 2 1 2 0 0 2 1 1 1 2 1 0 2 1 1 0 2 1 0
1 0 1 2 1 0 1 2 1 0 0 2 0 2 0 0 0 0 2 1 1 1 0 1 1 1 2 1 2 2 0
0 1 1 1 1 1 2 1 0 1 1 2 2 2 1 2 0 2 1 1 2 1 1 2 0 1 1 2 2 1 2
0 1 2 1 1 1 1 1 2 2 1 1 1 2 0 2 1 2 0 0 2 1 1 1 1 1 1 0 2 1 1
2 2 0 1 0 2 0 1 2
NAU3212 2 2 2 2 1 0 1 0 2 1 2 0 0 1 1 1 1 0 1 1 2 1 1 0 2 0 1
1 0 1 2 1 0 1 1 1 0 0 2 1 2 0 0 0 0 2 1 1 1 0 1 1 1 1 1 2 2 0
0 1 1 1 1 2 1 1 1 1 1 2 2 2 1 2 0 2 2 1 2 1 1 2 0 1 2 2 2 1 2
0 1 2 1 1 1 1 1 1 2 1 1 5 2 0 2 1 2 1 0 2 2 1 1 1 1 1 0 2 2 1
2 1 1 1 0 2 0 1 2
CIR373 2 2 1 2 1 1 1 0 2 1 2 0 0 1 1 1 2 1 1 1 2 0 2 0 2 0 1 1
1 1 2 1 0 1 1 1 0 0 2 1 2 1 0 0 1 2 1 1 1 0 1 1 1 1 2 2 2 1 1
1 1 1 1 2 0 1 1 1 1 1 2 1 1 2 0 2 5 2 2 1 1 2 0 1 2 2 2 1 2 0
1 2 1 1 1 0 1 1 0 1 1 1 0 2 0 1 1 1 2 0 0 1 1 1 5 1 2 0 0 1 0
1 1 1 2 0 2 1 1
GH82 0 2 1 0 1 1 0 2 0 2 2 2 2 1 1 2 2 1 0 1 1 2 2 1 1 0 1 1 1
1 1 1 1 0 0 2 2 0 1 1 0 1 2 1 0 2 1 2 0 0 1 1 2 0 0 1 0 1 1 2
1 1 1 1 2 2 1 2 1 1 1 1 1 1 2 0 2 1 0 1 0 2 2 1 1 1 2 1 0 1 2 0
1 1 1 2 2 1 1 1 0 0 0 2 1 1 2 1 2 1 0 2 2 5 1 2 0 0 2 0 2 1 2
0 1 0 1 0 1 1
GH39 0 1 2 1 2 1 1 1 0 2 2 1 2 1 2 0 1 1 1 1 0 2 2 1 0 1 1 1 1
2 0 1 1 1 0 1 1 0 1 0 0 1 1 2 0 2 1 1 0 1 0 1 2 1 1 1 1 0 1 0
1 2 2 0 2 2 2 2 1 2 1 1 0 2 0 1 0 0 2 0 1 0 2 2 1 1 1 1 0 2 0
0 1 1 2 2 1 1 1 0 0 0 2 1 1 1 0 2 1 0 1 2 0 2 1 1 2 1 2 2 2 2
2 1 1 1 1 2 1
GH32 0 1 2 1 2 1 1 1 0 2 2 1 2 1 2 0 1 0 1 1 0 2 2 1 0 1 1 1 1
2 0 1 1 1 0 1 1 0 1 0 0 1 1 2 0 2 1 1 0 1 0 1 2 1 1 1 1 0 1 0
1 2 2 0 2 2 2 2 1 2 1 1 0 2 0 1 0 0 5 0 1 0 2 2 1 1 1 1 0 2 0
0 1 1 2 2 1 1 1 0 0 0 2 1 1 1 0 2 0 0 1 2 0 2 0 0 2 1 2 2 2 2
2 0 0 1 1 2 1

TMB0154 0 1 2 1 2 1 1 1 0 2 2 1 2 1 2 0 1 0 1 1 0 2 2 1 0 1 1
1 1 2 0 1 1 1 0 1 1 0 1 0 0 1 1 2 0 2 1 1 0 1 0 1 2 1 1 1 1 0
1 0 1 2 2 0 2 2 2 2 1 2 1 1 0 2 0 1 0 0 2 0 1 0 2 2 1 1 1 1 0
2 0 0 0 1 2 2 1 1 1 0 0 0 2 1 1 1 0 2 0 0 1 2 0 2 1 0 2 1 2 1
2 2 2 0 0 1 1 2 1
TMB1538 0 1 2 1 2 1 1 1 0 2 2 1 2 1 2 0 1 0 1 1 0 2 2 1 0 1 1
1 1 2 0 1 1 1 0 1 1 0 1 0 0 1 1 2 0 2 1 1 0 1 0 1 2 1 1 1 1 0
1 0 1 2 2 0 2 2 2 2 1 2 1 1 0 2 0 1 0 0 2 0 1 0 2 2 1 1 1 1 0
2 0 0 0 1 2 2 1 1 1 0 0 0 2 1 1 1 0 2 0 0 1 2 0 2 1 0 2 1 2 1
2 2 2 0 0 1 1 2 1
JESPR119 0 1 2 2 2 1 1 1 1 2 2 1 1 2 1 0 1 0 2 1 0 1 2 1 0 2 0
1 1 2 0 1 1 2 0 1 2 0 1 1 0 1 1 2 0 2 0 1 2 1 0 0 1 1 0 1 2 1
1 0 1 2 1 0 1 2 2 2 1 1 1 0 1 2 0 1 0 1 2 0 1 0 2 2 0 0 2 1 1
5 0 0 1 1 1 0 1 1 1 1 1 0 1 0 0 1 0 2 0 0 1 2 1 2 1 0 2 1 2 1
2 2 2 0 1 5 5 5 5
GH98 1 0 2 0 0 1 2 1 1 2 0 1 2 1 1 0 2 2 2 0 1 1 0 0 1 1 1 2 0
1 0 1 1 0 2 0 2 1 1 0 2 0 2 1 2 1 1 0 1 1 0 2 0 0 1 1 0 2 2 0
1 2 1 1 1 2 2 1 0 0 0 0 2 1 1 0 1 1 2 1 1 1 2 2 1 1 1 1 2 1 1
1 1 1 0 1 2 2 1 0 1 0 2 0 0 2 1 0 1 0 2 2 0 2 1 0 2 1 0 2 1 2
1 2 1 1 1 1 1
GH27 1 0 2 0 1 1 2 1 1 2 0 1 2 1 1 0 2 2 2 0 1 1 0 0 1 1 1 2 0
1 0 1 1 0 2 0 2 1 1 0 2 0 2 1 2 2 1 0 1 1 0 2 0 0 1 1 0 2 2 0
1 2 1 1 1 2 2 1 0 0 0 0 2 1 1 0 1 1 2 2 1 1 2 2 1 1 1 1 2 1 1
1 1 1 0 1 2 2 1 0 1 0 2 0 0 1 0 0 1 0 2 2 0 2 1 0 2 1 0 2 1 2
1 2 1 1 1 1 1
GH112 1 0 2 0 1 1 2 1 1 2 0 1 2 1 1 0 2 2 2 0 1 1 0 0 1 1 1 2
0 1 0 1 1 0 2 0 2 1 1 0 2 0 2 1 2 2 1 0 1 1 0 2 0 0 1 1 0 2 2
0 1 2 1 1 1 2 2 1 0 0 0 0 2 1 1 0 1 1 2 2 1 1 2 2 1 1 1 1 2 1
1 1 1 1 0 1 2 2 1 0 1 0 2 0 0 1 0 0 1 0 2 2 0 2 1 0 2 1 0 2 1
2 1 2 1 1 1 1 1
TMB0184 1 0 2 0 1 1 2 1 1 2 0 1 1 1 1 0 2 2 2 0 1 1 0 0 1 1 1
2 0 1 1 1 1 0 2 1 2 1 1 1 2 0 2 2 2 2 1 0 1 1 1 2 0 0 1 1 0 2
1 1 1 2 1 1 1 2 2 1 0 0 1 0 2 1 2 0 1 1 2 2 2 1 2 2 1 1 1 1 2
2 1 1 1 1 0 1 1 2 1 0 1 0 2 0 1 1 0 0 2 0 2 2 0 2 1 0 2 1 0 2
1 2 2 1 1 1 1 5 1
GH118 1 0 0 0 1 2 2 0 0 2 0 1 1 1 0 1 1 0 1 0 2 1 1 1 2 1 1 1
2 1 1 1 1 1 2 1 2 1 1 2 1 0 2 2 0 2 1 0 0 2 1 1 0 1 1 0 1 1 1
1 1 0 1 2 1 1 1 1 0 1 2 1 2 2 2 0 1 1 2 1 2 2 2 2 1 0 1 0 1 1
0 1 0 2 0 0 1 2 1 1 2 2 2 5 2 2 0 1 2 1 2 1 2 1 0 0 2 2 1 1 1
1 2 1 2 5 1 1 1
BNL1414 1 0 0 0 1 2 2 0 0 0 2 1 1 1 0 1 1 0 1 0 1 0 0 2 1 1 1
1 2 1 0 1 1 2 1 1 5 1 2 1 2 0 2 1 0 2 1 2 0 1 1 1 1 2 1 0 1 2
2 1 1 0 1 2 1 1 1 1 1 1 2 1 2 2 2 0 0 1 2 1 2 2 2 1 2 1 1 1 1
1 0 5 0 1 0 0 1 2 1 1 0 2 2 1 2 2 0 2 2 1 1 1 5 1 0 1 1 1 1 1
1 1 1 1 2 1 1 1 1
BNL4028 1 0 0 0 1 2 2 0 0 0 2 1 1 1 0 1 1 0 1 1 1 0 0 2 1 1 1
1 2 1 0 1 1 2 1 1 2 1 2 1 2 0 2 1 1 2 0 2 0 1 1 1 1 2 1 0 1 2
2 1 1 0 1 1 1 0 1 1 1 1 2 1 2 2 2 1 0 1 2 1 2 2 2 1 2 1 1 1 2
1 0 1 0 2 0 0 1 1 1 1 1 1 2 2 1 2 2 0 2 1 1 1 1 2 1 0 1 1 1 1
1 0 1 1 2 1 1 1 1
PhyB 1 0 0 1 2 2 0 0 1 1 1 1 2 1 2 0 1 1 1 1 1 1 1 0 2 0 0 1 1
1 2 2 1 1 2 2 2 0 2 2 1 0 2 2 2 1 2 0 1 0 1 1 2 2 2 1 0 0 0 1
2 1 1 1 1 2 0 2 2 1 1 1 1 1 1 1 1 0 1 1 1 1 1 1 0 1 1 1 0 1 1
2 1 1 1 0 0 2 0 1 0 2 1 1 0 1 0 1 1 1 0 1 1 1 1 2 1 2 1 0 1 1
1 1 1 1 1 1 0
PhyB2 1 0 0 1 2 2 0 0 1 1 1 1 2 1 2 0 1 1 1 1 1 1 1 0 2 0 0 1
1 1 2 2 1 1 2 2 2 0 2 2 1 0 2 2 2 1 2 0 1 0 1 1 2 2 2 1 0 0 0

1 2 1 1 1 1 2 0 2 2 1 1 1 1 1 1 1 1 0 1 1 1 1 1 1 0 1 1 1 0 1
1 2 1 1 1 0 0 2 0 1 0 2 1 1 0 1 0 1 1 1 0 1 1 1 1 2 1 2 1 0 1
1 1 1 1 1 1 1 0
BNL2872 1 0 0 1 2 1 0 0 1 1 1 1 2 1 2 0 1 1 1 0 1 1 1 0 2 0 0
1 0 1 2 2 1 1 2 2 2 0 2 2 1 0 2 1 2 1 2 0 1 0 1 1 2 2 2 1 0 0
0 1 2 1 0 2 1 2 0 2 2 1 1 1 1 1 1 1 1 0 1 1 2 1 1 1 0 1 1 1 1
1 1 2 1 1 1 0 0 2 0 1 0 1 1 1 0 1 0 1 1 1 0 1 1 1 0 1 1 2 1 0
1 1 1 1 1 1 1 1 0
TMB0325 1 0 0 1 2 1 0 0 1 1 1 1 2 1 2 0 1 1 1 0 1 1 1 0 2 0 0
1 0 1 2 2 1 1 2 5 2 0 5 2 1 0 2 5 5 1 2 0 1 0 1 5 2 2 2 1 0 0
0 1 2 1 0 2 1 2 0 2 2 1 1 1 1 1 1 1 1 0 5 1 2 5 1 1 0 1 1 1 1
1 1 2 1 1 1 0 0 2 0 1 0 1 1 1 0 1 0 1 1 1 0 1 1 1 0 1 1 2 1 0
1 1 1 1 1 1 1 1 0
TMB0307 1 0 0 1 2 1 0 0 1 1 1 1 2 1 2 0 1 1 1 0 1 1 1 0 2 0 0
1 0 1 2 2 1 1 2 1 2 0 2 2 1 0 2 1 2 1 2 0 1 0 1 1 2 2 2 1 0 0
0 1 2 1 0 2 1 2 0 2 2 1 1 1 1 1 1 1 1 0 1 1 2 1 1 1 0 1 1 1 1
1 1 2 1 1 1 0 0 2 0 1 0 1 1 1 0 1 0 1 1 1 0 1 1 1 0 1 1 2 1 0
1 1 1 1 1 1 1 1 0
BNL2705 1 0 0 1 2 1 0 0 1 1 1 1 2 1 2 0 1 1 1 0 1 1 1 0 2 0 0
1 0 1 2 2 1 1 2 1 2 0 2 2 1 0 2 1 2 1 2 0 1 0 1 1 2 2 2 1 0 0
0 1 2 1 0 2 1 2 0 2 2 1 1 1 1 1 1 1 1 0 1 1 2 1 1 1 0 1 1 1 1
1 1 2 1 1 1 5 0 2 0 1 0 1 1 1 0 1 0 1 1 1 0 1 1 1 0 1 1 2 1 0
1 1 1 1 1 1 1 1 0
TMB0380 0 0 0 1 2 1 0 0 1 1 1 1 2 2 2 0 1 1 1 0 1 1 1 0 1 0 0
1 0 1 2 2 1 1 2 1 2 1 2 2 1 0 2 1 1 1 2 0 1 0 1 1 2 1 2 1 0 0
0 1 2 1 0 2 1 2 0 2 2 0 1 0 1 0 1 2 1 0 1 1 2 0 1 1 1 1 1 1 1
1 1 2 1 1 1 0 1 2 0 1 0 1 1 1 0 1 0 0 2 2 0 1 1 1 0 1 1 2 1 1
1 1 1 1 2 1 1 1 0
CM67 0 0 0 1 2 1 0 0 1 1 1 1 2 2 2 0 1 1 2 0 1 1 1 1 1 0 0 1 0
1 2 2 1 1 2 1 2 1 2 2 1 0 2 1 1 1 2 0 1 0 1 1 2 1 2 1 0 0 0 1
2 1 0 2 0 2 0 2 2 0 1 0 1 0 1 2 1 1 1 1 2 0 0 1 1 1 1 1 1 1 1
5 5 1 1 0 1 2 0 1 0 1 1 1 0 1 0 0 2 2 0 1 5 1 0 1 1 2 1 1 2 1
1 1 2 1 1 1 0
BNL2960 1 0 0 1 1 2 0 1 0 1 1 1 1 1 2 1 1 1 1 1 0 2 1 0 1 1 1
1 1 1 2 2 1 1 1 1 2 0 2 2 0 2 0 2 2 1 1 0 1 0 1 1 1 2 1 1 0 0
0 1 2 1 1 1 1 2 0 2 2 1 1 1 1 1 1 1 2 0 1 1 1 1 1 1 0 1 1 1 1
1 1 2 1 1 1 1 0 2 0 2 0 2 1 1 0 1 0 1 1 1 0 1 1 2 1 2 1 2 1 0
1 1 1 1 1 1 1 1 0
BNL1665 1 0 0 0 1 0 0 0 1 1 0 1 1 2 2 1 1 1 2 0 1 1 0 1 1 1 0
1 1 0 2 2 1 2 2 1 1 1 2 2 1 0 2 1 1 1 2 0 1 0 1 2 2 1 2 1 0 1
0 1 2 1 0 2 0 2 0 2 2 0 1 0 2 1 1 2 0 1 1 1 2 0 0 1 1 2 0 1 1
1 1 1 2 0 1 1 1 2 0 1 0 0 0 1 0 0 0 0 2 2 0 2 1 1 0 1 1 2 1 1
2 1 1 1 2 1 1 2 0
TMB1745 1 0 0 0 1 1 0 0 1 1 0 1 1 2 2 1 1 1 2 0 1 1 0 1 2 0 0
1 1 0 2 2 1 2 1 2 1 1 1 2 1 0 2 1 1 1 2 0 1 0 1 2 2 1 2 1 0 1
0 1 2 1 0 2 0 2 0 2 2 0 1 0 2 1 1 2 0 1 1 1 2 0 0 1 1 2 0 2 1
1 1 1 2 0 2 1 1 2 0 1 0 0 0 1 0 0 0 1 2 2 0 2 1 1 0 1 1 2 1 1
2 1 1 1 2 1 1 0 0
BNL1066 1 2 2 2 0 2 1 2 1 1 1 1 1 1 1 1 1 1 0 1 2 5 1 1 2 2 1
2 1 2 2 2 1 2 1 1 1 0 1 1 1 1 0 0 0 1 2 1 0 2 1 0 1 1 2 2 1 2
2 1 1 0 2 1 1 2 0 1 1 1 2 0 1 0 0 2 1 0 0 2 1 1 0 0 2 2 0 2 1
2 1 2 0 1 2 2 1 2 0 1 1 1 2 1 1 2 1 2 1 0 2 1 1 1 0 0 1 1 0 0
1 1 1 1 1 0 1 0 2
JESPR296 0 2 2 2 0 1 1 2 0 1 1 1 1 1 2 1 0 1 0 1 2 1 2 1 2 2 1
2 1 2 2 2 2 2 1 0 2 0 0 1 1 1 0 0 0 0 2 1 0 2 1 0 2 1 1 2 1 2
2 1 2 1 2 2 1 2 1 1 1 1 0 0 0 0 0 2 1 0 0 1 2 0 1 0 2 1 1 2 2
2 2 2 0 1 2 1 2 2 1 1 1 1 2 1 0 2 1 2 1 1 1 1 1 0 0 0 2 2 0 0

1 1 1 1 1 1 1 1 1 2
PhyA 0 2 2 2 2 0 1 1 2 0 1 1 1 1 1 2 1 0 1 0 1 2 1 2 1 2 2 1 1 0
2 2 2 2 2 1 0 2 0 0 1 1 1 0 0 0 0 2 1 0 2 1 0 2 2 1 2 1 2 2 1
1 1 2 2 1 2 1 1 1 1 0 0 0 0 1 2 1 0 0 1 2 0 2 0 2 1 1 1 2 2 2
2 0 1 2 1 2 2 1 1 1 1 2 1 0 2 1 2 1 1 1 1 1 0 0 0 2 2 0 0 1 1
1 1 1 1 1 1 1 2
NAU1014 0 1 1 1 1 1 1 1 2 0 1 2 2 1 1 2 1 1 1 0 1 1 1 2 1 1 1 1
1 1 1 2 2 2 2 1 0 2 0 0 1 1 1 0 0 0 0 2 1 0 2 1 0 2 2 1 1 1 2
2 1 1 1 2 2 0 2 1 1 1 1 0 0 0 0 1 2 0 1 1 1 2 0 2 1 2 1 2 2 2
1 2 2 1 1 2 1 2 1 1 1 0 1 2 1 1 2 1 2 1 1 1 1 1 0 0 1 2 2 0 0
1 1 1 1 1 2 1 0 2
TMB0064 0 1 1 1 1 1 1 1 2 0 1 2 2 1 1 2 1 1 1 0 1 1 1 2 1 1 1 1
1 1 1 2 2 2 1 1 0 2 0 0 1 1 1 0 0 0 0 2 1 0 2 1 0 2 2 1 1 1 2
2 1 1 1 2 2 0 2 1 1 1 1 0 0 0 1 2 2 0 1 1 1 2 0 2 1 2 1 2 2 2
1 2 2 1 1 2 1 2 1 1 1 0 1 2 1 1 2 1 2 1 1 1 1 1 0 0 1 2 2 0 0
1 1 1 1 1 2 1 0 2
BNL625 0 1 1 1 1 1 1 1 2 0 1 2 2 1 1 2 1 1 1 0 1 1 1 2 1 1 1 1 1
1 1 2 2 2 1 1 0 2 0 0 1 2 1 0 0 0 0 2 1 0 2 0 0 2 1 1 1 1 1 2
1 1 1 2 2 0 1 1 1 1 1 0 0 0 1 2 2 0 1 1 1 2 0 2 1 2 1 2 2 2 1
2 1 1 1 2 1 1 1 1 1 1 1 2 1 1 1 1 2 2 1 1 1 1 0 0 1 5 1 0 0 1
1 1 1 1 2 1 0 2
TMB0359 0 1 1 1 1 1 1 1 2 0 1 2 2 1 1 2 1 1 1 0 1 1 1 1 1 1 1 1
1 1 1 2 2 2 1 1 0 2 0 0 1 2 1 2 2 2 0 1 1 0 2 0 0 2 1 1 1 1 1
2 1 1 1 2 2 0 1 1 1 1 1 1 0 0 0 1 2 2 0 1 2 1 2 0 2 1 2 1 2 2 2
1 1 1 1 1 2 1 1 1 1 1 1 1 2 1 1 1 1 2 2 1 1 1 1 0 0 1 2 1 0 0
1 1 1 1 1 2 1 0 2
TMB0799 1 1 0 0 2 1 2 2 1 2 1 1 0 0 0 1 1 0 2 1 0 0 1 1 1 2 2
0 0 1 2 1 2 2 1 1 1 0 1 1 1 2 0 2 0 2 1 2 1 1 1 1 1 1 1 1 2 1
2 0 1 2 2 1 1 1 0 2 0 1 1 1 1 0 2 2 2 1 1 0 1 0 2 0 1 1 1 2 1
1 0 1 1 2 1 1 1 1 1 2 1 1 0 0 0 1 1 2 2 0 1 2 1 1 0 1 2 2 1 1
2 1 2 1 0 1 0 1 0
JESPR270 1 2 0 1 2 2 2 1 1 1 1 1 1 1 0 0 1 1 1 0 0 1 1 1 2 2 2
1 2 1 2 2 1 2 1 1 1 0 1 1 1 2 0 1 1 2 1 2 2 0 1 1 1 2 0 1 2 1
1 1 1 1 2 0 1 1 1 2 2 2 1 0 1 0 2 2 2 2 1 0 0 0 1 1 1 2 0 2 1
2 0 5 1 2 0 1 1 1 1 1 1 1 1 0 1 1 1 0 1 0 0 1 2 1 0 1 1 0 0 0
2 1 2 1 1 0 0 0 0
NAU1237 1 2 0 1 2 2 2 1 1 1 1 1 1 1 0 0 1 1 1 0 0 1 1 1 2 2 2
1 2 1 2 2 1 2 1 1 1 0 1 1 1 1 0 1 1 2 1 2 2 0 1 1 1 2 0 1 2 1
1 1 1 1 1 0 1 1 1 2 2 2 1 0 1 0 1 2 2 2 0 0 0 0 1 1 1 2 1 2 1
2 0 1 1 2 0 1 1 1 1 1 2 2 1 0 5 1 1 0 0 0 1 1 2 1 0 1 1 0 0 0
2 1 1 1 1 0 0 1 0
BNL3835 1 2 0 1 2 2 2 1 1 1 1 1 1 1 0 0 1 1 1 0 0 1 1 1 2 2 2
1 2 1 2 2 1 2 1 1 1 0 1 1 1 1 0 1 1 2 1 2 2 0 1 1 1 2 0 1 2 1
1 1 1 1 1 0 1 1 1 2 2 2 1 0 1 0 1 2 2 2 0 0 0 0 1 1 1 2 1 2 1
2 0 1 1 2 0 1 1 1 1 2 2 2 1 0 0 1 1 0 0 0 1 1 2 1 0 1 1 0 0 0
2 1 1 1 1 0 0 1 1
NAU1278 1 2 1 1 2 2 2 1 1 0 1 0 1 1 0 0 1 0 1 0 0 1 1 2 2 2 2
0 2 1 2 2 1 2 1 1 1 0 1 2 1 1 0 1 1 2 1 2 1 0 1 1 1 2 0 1 1 1
1 1 1 1 1 1 1 1 1 2 2 2 1 0 1 0 1 2 2 2 0 2 1 0 1 1 1 1 1 2 1
1 0 1 1 2 0 1 1 1 1 0 2 2 1 0 1 1 1 1 1 0 1 1 2 1 1 1 1 1 0 0
2 1 1 1 1 0 0 1 1
BNL1679 1 2 1 1 0 2 1 1 1 0 2 0 0 2 0 0 2 0 1 1 0 1 1 1 1 1 1
0 1 0 1 1 0 1 2 1 1 0 1 2 2 1 0 1 1 2 1 1 1 1 0 1 0 0 2 1 1 1
1 0 1 1 1 2 1 2 0 2 1 2 1 1 1 1 1 1 1 2 1 1 2 1 1 1 1 1 1 1 2 1
0 1 1 2 1 0 0 0 1 1 0 2 1 1 0 1 1 1 1 1 1 1 1 2 2 0 1 1 1 2 0 0
2 1 0 1 1 1 0 1 1
CIR148 1 2 1 1 0 2 1 1 1 0 2 0 0 1 0 0 2 0 0 1 0 1 1 1 1 1 1 1 0

1 0 1 1 0 1 2 1 1 0 1 2 2 1 0 1 1 2 1 1 1 1 0 1 0 0 2 1 1 1 1
0 1 1 1 2 1 2 0 2 1 2 1 1 1 1 1 1 2 1 1 2 1 1 1 1 1 1 1 2 1 0
1 1 2 1 0 0 0 1 1 0 2 1 1 0 1 1 1 1 1 1 1 2 2 0 5 5 1 2 0 0 5
1 0 1 5 1 0 1 1
NAU5047 0 2 1 1 0 0 2 1 1 0 2 0 1 1 0 0 2 0 1 1 0 1 0 1 1 0 1
0 1 0 1 1 0 1 2 0 1 0 2 2 1 1 0 1 1 1 1 1 2 1 0 1 0 0 2 1 1 1
1 1 1 1 2 2 1 2 0 2 1 2 0 1 1 0 1 2 2 1 1 2 1 1 1 1 2 2 1 2 1
0 1 1 1 1 0 0 0 2 1 0 2 1 1 0 1 1 1 1 2 1 2 2 2 0 1 1 1 2 0 0
1 1 0 1 1 1 0 1 1
CM85 0 2 0 0 0 0 2 1 1 0 2 0 1 1 0 1 2 0 1 1 0 1 0 1 1 0 1 0 1
0 1 1 0 1 2 0 1 0 2 2 1 1 0 1 1 1 1 1 2 1 0 1 1 0 2 1 1 1 2 1
1 1 2 1 1 2 0 1 1 2 0 1 1 0 1 2 2 1 1 2 1 1 2 1 2 2 1 2 1 0 1
1 1 1 1 1 1 2 1 1 2 1 2 1 1 1 1 1 2 0 2 2 2 0 0 1 5 2 0 0 1 1
1 1 1 1 0 1 1
BNL1495 2 1 2 2 0 2 1 1 0 1 1 1 2 0 2 0 1 2 1 0 2 1 0 0 1 2 0
1 1 2 1 1 2 2 1 1 1 1 1 0 0 1 2 2 1 2 1 1 2 2 1 1 0 1 1 1 1 1
0 0 0 1 2 0 1 2 1 1 2 1 2 1 2 0 1 0 0 0 0 1 1 1 2 1 1 2 2 1 2
2 0 0 0 2 1 1 0 0 2 1 1 0 1 1 2 2 2 1 2 2 1 2 1 0 1 2 0 0 1 1
1 2 0 0 0 1 1 1 1
BNL1421 2 1 2 2 0 2 1 1 0 1 1 1 2 0 2 0 1 2 1 0 2 1 0 0 1 2 0
1 1 2 1 1 2 2 1 1 1 1 1 0 0 1 2 2 1 2 1 1 2 2 1 1 0 1 1 1 1 1
0 0 0 1 2 0 1 2 1 1 2 1 2 1 2 0 1 0 0 0 0 1 1 1 2 1 1 2 2 1 2
2 0 0 0 2 1 1 0 0 2 1 1 0 1 1 2 2 2 1 2 2 1 2 1 0 1 2 0 0 1 1
1 2 0 0 0 1 1 1 1
BNL3623 2 1 2 2 0 2 1 1 0 1 1 1 2 0 2 0 1 2 1 0 2 1 0 0 1 2 0
1 1 2 1 1 2 2 1 1 1 1 1 0 0 1 2 2 1 2 1 1 2 2 1 1 0 1 1 1 1 1
0 0 0 1 2 0 1 2 1 1 2 1 2 1 2 0 1 0 0 0 0 1 1 1 2 1 1 2 2 1 2
2 0 0 0 2 1 1 0 0 2 1 1 0 1 1 2 2 2 1 2 2 1 2 1 0 1 2 0 0 1 1
1 2 0 0 0 1 1 1 1
GH215 2 1 2 2 0 2 1 1 0 1 1 2 1 0 2 0 0 1 1 0 2 2 0 0 0 1 0 0
1 2 0 1 2 1 1 1 1 1 2 0 1 1 2 2 1 2 0 1 2 2 1 1 0 1 1 1 0 1 0
0 0 1 2 0 1 2 1 1 2 1 2 1 2 0 1 1 0 1 1 1 1 1 2 1 1 2 2 1 2 2
0 5 0 2 1 1 0 0 2 1 0 0 1 1 2 1 2 2 2 2 1 2 1 1 1 2 0 0 1 0 1
2 0 0 0 1 1 1 0
GH34 2 2 2 2 0 1 1 1 0 0 1 1 1 2 2 0 0 0 0 0 2 2 0 0 0 1 1 1 2
1 1 1 2 1 1 1 0 1 2 0 2 1 2 2 1 2 0 1 2 2 1 2 0 0 1 2 1 1 0 0
1 1 1 0 1 2 1 1 1 0 2 2 2 1 1 1 0 1 1 1 1 1 2 1 0 1 2 1 2 2 0
0 0 1 2 2 1 0 2 1 0 0 1 0 2 2 2 1 2 2 1 2 1 0 1 2 0 1 1 1 2 2
0 0 0 1 1 0 1
BNL4061 0 2 2 2 0 1 1 2 0 0 1 1 1 2 1 0 0 1 0 1 2 1 0 0 0 2 1
1 1 0 1 1 2 1 1 1 0 1 2 1 2 1 2 1 1 2 0 2 1 2 1 2 0 0 1 2 1 1
0 0 1 1 1 0 1 2 1 2 1 0 1 2 1 1 1 1 0 1 0 1 1 0 2 1 1 1 1 1 1
1 0 0 1 1 0 1 1 0 2 0 0 0 1 1 1 1 1 1 2 2 1 1 1 0 1 2 0 1 1 1
2 2 0 1 0 1 1 0 1
JESPR165 1 2 1 1 2 1 1 0 1 0 2 1 0 1 1 1 1 1 0 2 1 0 1 2 2 1 0
1 1 1 0 0 1 0 0 1 2 1 2 1 0 1 1 0 2 1 1 2 0 1 0 1 0 1 1 0 1 2
1 2 1 2 2 0 2 1 1 0 0 0 1 0 0 0 0 2 2 1 0 1 0 1 1 2 1 1 1 2 1
1 1 2 1 1 2 1 0 2 1 0 1 1 2 1 0 0 1 1 1 0 1 2 1 1 2 1 0 0 2 2
5 1 2 1 1 1 2 0 1
BNL3502 1 2 1 1 2 1 1 0 1 0 2 1 0 1 0 1 1 0 0 2 1 0 1 1 2 1 0
1 1 1 0 0 1 0 0 1 1 1 2 1 1 1 1 0 2 1 1 2 0 1 0 1 0 1 1 0 0 2
1 2 1 2 2 0 1 1 1 0 0 0 0 1 0 0 0 2 2 1 0 1 0 1 1 2 0 1 1 2 1
0 1 2 1 1 2 0 0 2 1 0 1 1 2 1 0 0 1 1 1 0 1 2 0 1 2 1 0 0 2 1
1 1 1 1 1 1 2 1 1
TMB0803 1 2 1 1 1 1 1 0 1 0 2 1 0 0 0 1 1 2 0 2 1 0 1 1 2 1 0
1 1 2 0 0 1 1 0 1 1 1 2 1 1 1 1 0 2 1 1 2 0 1 0 1 0 1 0 0 1 2
1 2 1 2 2 0 1 1 1 0 0 0 0 0 0 0 0 2 1 1 0 1 0 1 1 1 0 1 1 2 1

1 1 2 1 0 1 1 0 2 1 0 1 1 2 1 0 0 1 2 1 0 1 2 1 1 1 1 0 0 2 1
1 1 0 1 1 1 2 1 2
NAU2336 2 1 1 1 1 1 1 1 1 0 1 1 1 0 0 1 1 2 0 1 0 0 0 1 2 1 0
0 1 2 0 2 1 1 1 1 1 1 1 1 1 1 1 0 2 1 2 2 0 1 0 0 1 1 1 1 1 2
1 2 1 2 1 0 1 1 1 0 0 0 0 1 0 0 0 1 1 1 0 1 0 1 1 1 0 2 0 1 1
1 1 2 1 0 1 1 0 1 1 0 1 1 2 1 1 0 1 2 1 0 1 2 1 1 1 0 2 0 2 2
1 0 0 1 0 1 2 1 1
JESPR152 1 0 1 1 1 0 1 1 0 2 0 2 2 1 0 0 1 2 1 0 1 2 0 0 2 1 2
1 1 2 1 1 5 2 1 1 1 1 2 2 2 0 2 1 1 1 1 2 1 1 1 2 1 0 1 2 1 1
2 0 2 2 0 1 1 1 2 2 2 1 1 2 0 0 1 1 1 2 1 2 0 0 2 1 1 1 1 1 2
1 0 0 1 1 0 1 1 2 1 0 1 1 1 0 1 0 1 1 1 0 1 2 0 1 0 2 1 2 1 1
2 1 1 0 1 0 1 2 1
TMB1664 1 0 0 2 1 0 0 2 0 1 1 1 2 1 1 0 1 2 2 1 0 1 0 0 2 1 2
2 1 1 1 1 1 2 1 1 1 0 2 2 2 0 1 1 1 1 1 1 0 1 2 2 2 1 1 2 2 1
2 0 2 2 1 2 1 1 2 1 1 1 1 2 0 1 1 0 1 2 1 2 0 0 1 1 1 2 0 1 2
1 1 0 2 1 2 1 0 2 1 0 2 1 2 1 1 0 1 0 1 0 1 2 1 1 0 2 2 1 1 0
1 1 1 0 1 1 1 2 1
TMB1660 1 0 0 2 1 0 0 2 0 1 1 1 2 1 1 0 1 2 2 1 0 1 0 0 2 2 2
2 1 0 5 1 1 2 1 1 1 0 1 1 2 0 1 1 1 1 1 1 0 1 2 2 2 1 1 2 2 1
2 0 2 2 1 2 1 1 2 1 1 1 1 2 0 1 1 0 1 2 1 1 0 0 1 0 1 2 0 1 2
1 1 1 2 1 2 1 0 2 1 0 2 1 2 1 1 0 1 0 1 0 1 2 1 1 0 2 2 1 1 0
0 1 1 0 1 1 1 2 1
BNL2920 1 0 0 2 1 0 0 2 0 1 1 1 2 1 1 0 1 2 2 1 0 1 0 0 2 2 2
2 1 0 1 1 1 2 1 1 1 0 1 1 2 0 1 1 1 1 1 1 0 1 2 2 2 1 1 2 2 1
2 0 2 2 1 2 1 1 2 1 1 1 1 2 0 1 1 0 1 2 5 1 0 0 1 0 1 2 0 1 2
1 1 1 2 1 2 1 0 2 1 0 2 1 2 1 1 0 1 0 1 0 1 2 1 1 0 2 2 1 1 0
0 1 1 0 1 1 1 2 1
TMB0201 1 1 0 2 1 0 0 2 0 1 1 1 2 1 1 0 1 2 2 1 0 1 0 0 2 2 2
2 1 0 1 1 1 2 1 1 1 0 1 1 2 0 1 1 1 1 1 0 0 1 2 2 2 1 1 2 2 1
2 0 2 2 1 2 1 1 2 1 1 1 1 2 0 1 1 0 1 2 0 1 0 0 1 0 1 2 0 1 2
1 1 1 2 1 2 1 0 2 1 0 2 1 2 1 1 0 1 0 2 0 1 2 0 1 5 2 5 1 1 0
0 1 1 1 1 1 1 2 1
TMB0301 1 1 0 2 1 1 0 2 1 2 1 1 2 1 1 0 1 2 2 2 0 1 0 1 2 2 2
2 2 0 1 1 1 2 1 1 1 0 1 1 2 0 1 1 1 1 0 0 0 1 2 1 2 1 1 1 2 1
2 0 2 2 1 2 2 1 2 1 1 1 0 2 0 1 1 0 2 2 0 0 1 0 1 0 1 2 0 1 2
1 2 1 5 1 1 1 0 2 1 0 1 0 2 1 1 0 1 1 2 0 1 2 0 0 0 2 2 1 1 0
0 1 1 2 1 1 1 2 1
JESPR180 1 1 0 2 1 1 0 2 1 2 1 1 2 1 1 0 1 2 2 2 0 1 0 1 2 2 2
2 2 0 1 1 1 2 1 1 1 0 1 1 2 0 1 1 1 1 0 0 0 1 2 1 2 1 1 1 2 1
2 0 2 2 1 2 2 1 2 1 1 1 0 2 0 1 1 0 2 2 0 0 1 0 1 0 1 2 0 1 2
1 2 1 2 1 1 1 0 2 1 0 1 0 2 1 1 0 1 1 2 0 1 2 0 0 0 2 2 1 1 0
0 1 1 2 1 1 1 2 1
JESPR298 1 1 0 2 1 1 0 2 1 2 1 1 2 1 1 0 1 2 2 2 0 1 0 1 2 2 2
2 2 0 1 1 1 2 1 1 1 0 1 1 2 0 1 1 1 1 0 0 0 1 2 1 2 1 1 1 2 1
2 0 2 2 1 2 2 1 2 1 1 1 0 2 0 1 1 0 2 2 0 0 1 0 1 0 1 2 0 1 2
1 2 1 2 1 1 1 0 2 1 0 1 0 2 1 1 0 1 1 2 0 1 2 0 0 0 2 2 1 1 0
0 1 1 2 1 1 1 2 1
BNL4082 1 1 0 2 2 1 0 2 1 2 1 1 2 2 1 0 2 2 2 2 0 1 0 1 2 2 2
2 2 0 1 1 1 2 1 0 1 0 0 1 2 0 1 2 1 0 0 0 0 1 2 1 2 1 1 1 2 1
2 0 2 2 1 2 2 1 2 1 1 1 0 1 0 1 1 0 2 2 0 0 1 0 1 0 1 2 0 1 2
5 2 1 2 1 1 0 0 2 1 0 1 0 2 1 1 0 1 1 2 0 1 2 0 0 0 2 2 1 1 0
0 1 1 2 1 1 1 2 1
TMB0375 1 1 0 2 2 1 0 2 1 2 2 1 2 2 1 0 2 2 2 2 0 1 1 1 2 2 2
2 2 0 1 1 1 1 1 0 1 0 0 1 1 0 1 2 1 1 0 1 0 1 2 1 2 1 1 1 2 1
2 0 2 2 1 2 2 1 2 1 1 1 0 1 0 1 1 0 2 2 0 0 1 0 1 0 1 2 0 1 2
0 2 1 2 1 1 0 0 2 1 0 1 0 2 1 1 0 1 1 2 0 1 2 0 0 0 2 2 1 1 0
0 1 1 2 1 1 1 2 1

BNL3902 1 1 0 2 2 1 0 2 1 2 2 1 2 2 1 0 2 2 2 2 0 1 1 1 2 2 2
2 1 0 1 1 1 1 1 0 1 0 0 1 1 0 1 2 1 1 0 1 0 1 2 1 2 1 1 1 2 1
2 0 2 2 1 2 2 1 2 1 1 1 0 1 0 1 1 0 2 2 0 0 1 0 1 0 1 2 0 1 2
0 2 1 2 1 1 0 0 2 1 0 1 0 2 1 1 0 1 1 2 0 1 2 0 0 0 2 2 1 1 0
0 1 1 2 1 1 1 2 1
BNL1350 1 1 0 2 1 1 0 2 1 2 2 1 2 2 2 0 2 2 1 2 0 1 1 1 1 2 2
2 1 0 1 1 1 1 1 0 1 0 0 1 1 0 1 2 1 1 0 1 0 1 2 0 2 1 1 1 2 1
2 0 2 2 1 2 2 1 2 1 1 2 0 1 0 1 1 0 2 2 0 0 1 0 1 0 1 2 0 1 2
0 2 1 2 1 1 0 0 2 1 0 1 0 2 1 1 0 0 1 2 0 1 2 2 0 0 1 2 1 1 1
0 1 1 1 1 1 1 1 0
TMB1181 0 1 0 2 1 1 0 1 1 1 2 1 2 1 2 0 2 2 1 2 0 1 1 1 1 1 2
1 1 0 1 0 1 2 1 2 2 0 0 1 1 0 1 2 1 1 0 1 0 1 2 0 2 1 2 1 2 1
1 0 1 2 1 2 2 0 1 1 1 2 0 2 0 1 1 0 2 2 0 0 0 0 1 0 1 2 0 2 2
1 2 2 2 1 1 0 0 2 1 1 2 0 2 1 1 0 0 1 2 0 1 2 2 1 1 0 5 1 1 1
1 1 1 0 2 0 1 1 2
BNL786 0 1 0 2 1 1 0 1 1 1 2 1 2 1 2 0 2 2 1 2 0 1 1 1 1 1 2 1
1 0 1 0 1 2 1 2 2 0 0 1 1 0 1 2 1 1 0 1 0 1 2 0 2 1 2 1 2 1 1
0 1 2 1 2 2 0 1 1 1 2 0 2 0 1 1 0 2 2 0 0 0 0 1 0 1 2 0 2 2 1
2 1 2 1 1 0 0 2 1 1 2 0 2 1 1 0 0 1 2 0 1 2 2 1 1 0 0 1 1 1 1
1 1 0 2 0 1 1 2
BNL3065 1 1 1 2 1 1 1 1 1 0 1 2 1 0 0 2 1 1 0 1 1 2 0 0 1 1 0
0 2 0 1 2 2 0 2 1 2 2 0 1 1 1 2 1 0 1 1 2 0 1 1 0 0 0 1 2 2 1
2 1 2 1 1 0 1 2 1 1 1 2 0 1 0 1 0 1 1 1 1 1 1 2 2 0 2 2 0 2 1
1 2 0 1 1 0 1 1 2 1 0 1 0 0 1 2 1 2 1 0 1 5 5 1 1 2 1 0 1 0 0
0 2 2 1 1 0 2 1 0
GH2 1 1 2 1 1 1 1 2 1 0 1 1 2 1 1 1 2 1 1 1 2 1 0 1 2 2 0 2 2
0 1 2 2 1 2 0 2 2 1 0 1 1 2 0 0 1 1 2 1 1 2 2 0 0 0 0 2 0 2 1
1 1 1 0 2 1 1 1 2 1 1 0 1 1 0 2 1 0 2 0 0 1 1 0 1 0 0 1 2 1 2
0 1 2 0 0 1 2 1 1 1 0 1 1 2 1 0 1 0 1 2 1 1 0 2 2 1 1 0 0 0 2
2 1 1 0 1 1 0
JESPR128 1 1 2 1 1 1 1 2 1 0 1 1 2 1 1 1 2 1 2 1 2 1 0 1 2 2 0
2 2 0 1 2 2 1 2 0 2 2 1 0 1 1 2 0 5 1 1 2 1 1 2 2 0 0 0 0 2 0
2 1 1 1 1 0 2 1 1 1 2 1 1 0 1 1 0 2 1 0 2 0 0 1 1 0 1 0 0 1 2
1 2 0 1 2 0 0 1 2 1 1 1 0 1 1 2 1 0 1 0 1 2 1 1 0 2 2 1 1 0 0
0 1 2 1 1 0 1 1 0
JESPR32 1 1 2 1 1 1 1 2 1 0 1 1 2 1 1 1 2 1 2 1 2 1 0 1 2 2 0
2 2 0 1 2 2 1 2 0 2 2 1 0 1 1 2 0 0 1 1 2 1 1 2 2 0 0 0 0 2 0
2 1 1 1 1 0 2 1 1 1 2 1 1 0 1 1 0 2 1 0 2 0 0 1 1 0 1 0 0 1 2
1 2 0 1 2 0 0 1 2 1 1 1 0 1 1 2 1 0 1 0 1 2 1 1 0 2 2 1 1 0 0
0 1 2 1 1 0 1 1 0
TMB1271 1 1 2 1 1 1 1 2 1 0 1 1 2 1 1 1 2 1 2 1 2 1 0 1 2 2 0
2 2 0 1 2 2 1 2 0 2 2 1 0 1 1 2 0 0 1 1 2 1 1 2 2 0 0 0 0 2 0
2 1 1 1 1 0 2 1 1 1 2 1 1 0 1 1 0 2 1 0 2 0 0 1 1 0 1 0 0 1 2
1 2 0 1 2 0 0 1 2 1 1 1 0 1 1 2 1 0 1 0 1 2 1 1 0 2 2 1 1 0 0
0 1 2 1 1 0 1 1 0
JESPR237 1 1 2 1 1 1 1 2 1 0 1 1 2 1 1 1 2 1 2 1 2 1 0 1 2 2 0
2 2 0 1 2 2 1 2 0 2 2 1 0 1 1 2 0 0 1 1 2 1 1 2 2 0 0 0 0 2 0
2 1 1 1 1 0 2 1 1 1 2 1 1 0 1 1 0 2 1 0 2 0 0 1 1 0 1 0 0 1 2
1 2 0 1 2 0 0 1 2 1 1 1 0 1 1 2 1 0 1 2 1 2 1 1 0 2 2 1 1 0 0
0 1 2 1 1 0 1 1 0
JESPR297 1 1 2 1 1 1 1 2 1 0 1 1 1 1 1 1 2 1 1 1 2 1 0 1 2 2 0
2 2 0 1 2 2 1 2 0 2 2 1 0 1 1 2 0 0 1 1 2 1 1 2 2 0 0 0 0 2 0
2 1 2 1 1 0 2 1 1 1 2 2 1 0 1 1 0 2 1 0 2 0 0 1 1 0 1 0 0 1 2
1 2 0 1 2 0 0 1 2 1 1 1 0 1 1 2 1 0 1 0 1 2 2 1 0 2 1 1 0 0 0
0 2 2 1 1 0 1 1 0
BNL2734 1 1 2 1 1 1 1 2 1 0 1 1 2 1 1 1 1 1 2 1 2 1 0 1 2 2 0
2 2 1 1 1 1 1 2 0 2 2 1 0 1 1 2 0 0 1 1 2 0 1 2 2 1 0 0 0 1 0

2 1 1 1 1 1 2 1 1 2 2 1 0 0 1 1 0 2 1 0 2 0 0 1 1 0 1 0 0 1 1
1 2 0 1 2 0 1 1 2 1 1 1 1 1 1 2 1 0 1 0 1 2 1 1 0 2 2 1 1 0 0
0 1 2 1 1 0 1 1 0
BNL3008 1 1 2 1 1 1 1 2 1 0 1 1 2 1 1 1 1 1 2 1 2 1 0 1 2 2 0
2 2 1 1 1 1 1 2 0 2 2 1 0 1 1 2 0 0 1 1 2 0 1 2 2 1 0 0 0 1 0
2 1 1 1 1 1 2 1 1 2 2 1 0 0 1 1 0 2 1 0 2 0 0 1 1 0 1 0 0 1 1
1 2 0 1 2 0 1 1 2 1 1 1 1 1 1 2 1 0 1 0 1 2 1 1 0 2 2 1 1 0 0
0 1 2 1 1 0 1 1 0
TMB1409 1 1 5 1 1 1 1 2 1 0 1 1 2 1 1 1 1 1 2 1 2 1 0 1 2 2 0
2 2 0 1 1 1 1 2 0 2 2 1 0 1 1 2 0 0 1 1 2 0 1 2 2 1 0 0 0 0 0
2 1 1 1 1 1 2 1 1 2 2 0 0 0 1 1 0 2 1 0 2 0 0 1 1 0 1 0 0 1 1
1 2 0 1 2 0 0 1 2 1 1 1 1 1 1 2 1 0 1 0 1 2 1 1 0 2 2 1 1 0 0
0 1 2 1 1 5 1 0 0
JESPR195 0 1 1 1 0 0 0 1 1 1 1 1 0 0 0 0 1 1 1 1 0 1 1 1 1 1 0
0 1 1 1 1 1 0 1 1 2 2 2 0 1 2 2 1 2 1 2 1 1 2 1 1 1 1 0 1 0 2
1 1 2 2 2 1 2 1 1 0 2 1 1 0 0 1 2 1 2 1 2 2 2 1 1 2 0 1 1 1 5
1 0 0 1 2 1 1 1 1 2 2 1 0 1 0 1 1 1 0 1 1 2 2 2 2 1 1 2 1 1 0
0 0 1 2 2 2 2 0 2
BNL4003 0 1 0 1 0 0 0 2 1 1 1 1 0 0 0 1 1 1 1 1 0 1 1 1 1 1 0
0 1 1 2 1 0 0 1 2 2 2 2 0 1 2 2 1 2 1 1 1 1 2 1 1 1 1 1 1 0 2
1 1 2 2 2 1 2 1 1 0 2 1 1 0 0 1 2 1 2 1 2 2 2 1 1 2 0 1 1 1 1
1 0 0 1 2 1 1 1 1 2 2 1 1 1 0 1 1 1 0 2 1 2 2 1 2 1 2 1 1 1 0
0 0 1 1 2 2 2 0 1
BNL3955 0 1 0 1 0 0 0 2 1 1 1 1 0 0 0 1 1 1 1 1 0 1 1 1 1 1 0
0 1 1 2 1 0 0 1 2 2 2 2 0 1 2 2 1 2 1 1 1 1 2 1 1 1 1 1 1 0 2
1 1 2 2 2 1 2 1 1 0 2 1 1 0 0 1 2 1 2 1 2 2 2 1 1 1 0 1 1 1 1
2 0 0 1 2 1 1 1 1 2 2 1 1 1 0 1 1 0 0 2 1 2 2 1 2 1 2 1 1 1 0
0 0 1 1 2 2 2 0 1
TMB2018 0 1 0 1 0 0 0 2 1 1 1 1 0 0 0 1 1 1 1 1 0 1 1 1 1 1 0
0 1 1 2 1 0 0 1 2 1 2 2 0 1 2 2 1 2 1 1 1 1 2 1 1 1 1 1 1 0 2
1 1 2 2 2 1 2 1 1 0 2 1 2 0 0 1 2 1 1 1 1 2 2 2 1 1 1 0 1 1 1 1
1 0 0 1 2 1 1 1 1 2 2 1 1 1 0 1 1 0 0 2 1 2 2 1 2 1 2 1 1 2 0
0 0 1 1 2 2 2 0 1
BNL3558 1 2 1 2 0 1 0 2 1 1 0 0 0 2 1 2 1 0 1 0 0 0 2 1 0 0 0
2 2 2 1 0 0 2 1 0 0 1 0 0 1 0 1 1 0 1 2 1 2 1 1 1 1 2 0 0 1 1
2 0 2 2 1 0 1 0 2 1 2 2 2 2 2 1 1 0 1 2 1 1 1 1 2 2 1 1 2 1 2
1 0 2 0 1 1 1 1 1 2 1 1 0 1 1 1 1 1 1 0 1 0 1 5 2 0 0 1 1 2 1
1 2 0 1 1 1 2 2 0
BNL1079 1 1 1 2 0 1 0 2 1 1 1 0 0 2 2 2 1 1 0 0 0 1 2 1 0 0 0
1 1 2 1 0 0 2 1 0 1 2 0 0 1 1 1 1 0 1 2 1 2 1 1 1 1 2 1 1 1 1
2 1 1 2 1 0 1 0 2 1 2 2 1 2 2 1 1 0 1 2 0 2 0 1 2 2 1 1 2 1 2
0 0 2 1 1 1 1 1 0 2 1 1 0 0 1 1 1 1 1 0 1 0 1 1 2 0 1 1 1 2 1
1 2 0 1 1 1 1 2 1
BNL2667 1 1 2 2 0 1 0 2 1 0 2 1 1 2 2 0 1 1 1 0 1 1 2 0 0 0 0
1 2 0 0 0 1 0 2 0 1 2 0 0 1 1 1 1 1 1 1 1 0 0 1 2 1 2 2 1 2 0
2 1 1 2 1 0 2 0 2 1 1 2 0 1 1 1 2 2 1 0 0 2 1 1 2 1 2 0 1 1 1
1 0 2 2 1 0 1 2 0 2 2 1 0 1 1 0 1 0 1 1 1 0 0 2 1 1 1 1 1 2 2
1 2 0 1 1 0 0 2 1
TMB1599 2 1 1 2 1 0 1 1 1 1 0 1 0 2 1 2 1 1 1 1 1 1 0 0 1 1 1
2 2 1 1 1 1 1 1 1 2 1 2 1 2 1 1 1 1 0 1 1 1 0 2 1 1 1 1 0 0 1
2 2 1 2 1 2 1 2 0 1 1 2 1 1 0 1 0 1 0 2 1 2 1 0 0 1 0 2 1 1 0
1 1 1 2 1 1 1 1 2 1 2 1 1 1 0 1 1 2 1 1 1 1 2 2 2 1 1 2 1 1 1
1 2 2 1 1 2 1 0 2
GH71 1 1 1 2 1 0 1 1 1 1 0 1 0 2 1 2 1 1 1 1 1 1 0 0 1 1 1 2 2
0 1 1 1 2 1 1 2 1 2 1 2 1 1 1 1 0 1 1 1 0 2 1 1 1 1 0 0 1 2 2
1 2 1 2 1 2 0 1 1 2 1 1 0 1 0 1 0 2 5 2 1 0 0 1 0 2 1 1 0 1 1
1 2 1 1 1 1 2 1 2 1 1 1 0 1 1 2 1 1 1 1 2 2 2 1 1 1 1 1 1 1 2

```
2 1 1 2 1 0 2
BNL285 1 1 1 1 2 0 0 1 2 1 0 1 0 2 1 0 1 1 1 1 1 1 2 2 1 1 1 2
2 0 2 1 1 1 1 1 2 1 1 2 2 1 1 1 1 2 1 1 1 0 2 1 1 2 1 0 0 1 2
2 1 2 1 2 0 2 0 1 1 2 1 1 0 1 1 1 1 1 1 2 2 1 0 1 0 2 1 1 2 1
1 1 2 1 1 2 1 1 0 1 0 1 0 0 1 1 2 1 1 1 1 2 2 2 0 1 1 1 1 1 1
1 2 1 1 2 1 2 2
NAU3935 1 1 1 1 2 0 1 1 2 0 0 1 1 2 0 0 1 1 1 1 1 1 0 0 1 1 1
2 2 0 0 1 1 1 1 1 2 1 1 2 2 1 1 1 1 0 1 1 1 0 1 1 1 2 1 0 0 2
2 1 1 2 2 2 0 2 0 2 1 2 1 1 1 0 1 1 1 1 1 2 2 1 1 1 0 2 1 1 0
1 1 1 2 1 1 2 1 1 0 1 0 1 1 0 1 1 2 1 1 1 2 2 2 2 0 1 0 1 1 1
1 1 2 1 1 1 1 0 2
BNL852 1 1 1 1 2 0 1 2 2 0 0 1 1 2 0 0 2 1 1 1 1 2 0 0 2 1 1 2
2 0 0 1 1 1 1 1 2 1 1 1 2 2 1 1 1 0 0 1 1 0 1 0 1 2 1 0 0 2 2
1 1 2 2 2 0 2 1 2 1 2 1 1 2 0 1 1 1 1 1 2 2 1 1 1 0 2 1 1 0 1
1 1 2 1 1 2 1 1 0 1 0 1 1 0 1 1 2 1 1 1 2 2 1 2 0 1 0 1 1 1 1
1 2 2 2 1 1 0 1
TMB0189 1 1 1 1 2 0 1 2 2 0 0 1 1 1 0 0 2 1 1 1 1 2 0 0 2 1 1
2 2 0 0 1 1 1 1 1 2 2 1 1 2 2 1 1 1 0 0 1 1 1 1 0 1 2 1 0 0 2
2 1 1 2 2 2 0 2 1 2 1 2 1 1 2 0 1 1 1 1 1 2 2 1 1 1 0 2 1 1 0
1 1 1 2 1 1 2 1 1 0 1 0 1 1 0 1 1 2 1 1 1 2 2 0 2 0 1 0 1 1 1
1 1 2 2 2 1 1 0 1
GH109 1 1 1 1 2 0 1 2 2 0 0 1 1 1 0 0 2 1 1 1 1 2 0 0 2 1 1 2
2 0 0 1 1 1 1 1 2 2 1 1 2 2 1 1 1 0 0 1 1 1 1 0 1 2 1 0 0 2 2
1 1 2 2 2 0 2 1 2 1 2 1 1 2 0 1 1 1 1 1 2 1 1 1 1 0 2 1 1 0 1
1 1 2 1 1 2 1 1 0 1 0 1 1 0 1 1 2 0 1 1 2 2 0 2 0 1 0 1 1 1 1
1 2 2 2 1 1 0 1
TMB0366 1 1 0 1 2 1 1 5 2 1 1 1 1 1 0 0 2 1 1 1 1 2 0 0 2 0 1
2 2 0 0 1 2 1 1 1 2 1 1 1 1 2 1 0 1 0 0 1 1 1 1 0 2 1 1 0 1 2
2 1 1 2 2 2 0 1 1 2 0 2 1 2 2 0 1 1 1 1 5 2 1 5 1 5 0 2 2 1 0
1 1 1 2 1 2 2 1 1 1 1 0 1 1 0 2 1 2 0 1 1 2 2 0 2 0 1 0 1 1 1
1 1 2 1 2 1 1 5 1
BNL4096 1 1 0 1 2 1 1 1 2 1 1 2 1 1 0 0 2 1 1 1 1 2 0 0 2 0 1
2 2 0 0 1 2 1 1 1 2 1 1 1 1 2 1 0 1 0 0 1 1 1 1 0 2 1 1 0 1 2
2 1 1 2 2 2 0 1 1 2 0 2 1 2 2 0 1 1 1 1 1 2 1 1 1 1 0 2 2 1 0
1 1 1 2 0 2 2 1 1 0 1 0 1 1 0 2 1 2 0 1 1 2 2 0 2 1 2 0 1 1 1
1 1 2 1 2 1 1 0 1
BNL3875 1 1 0 1 1 1 0 1 2 1 1 1 1 1 1 0 2 0 1 1 1 2 0 0 2 0 1
2 2 0 0 1 2 1 1 1 2 1 1 1 1 2 2 0 1 1 0 1 1 1 1 1 2 1 1 0 1 2
2 1 1 2 2 2 0 1 1 2 0 2 1 2 2 0 2 2 1 1 1 2 1 1 1 1 0 2 2 1 1
1 1 1 2 1 2 1 1 1 1 0 0 1 1 1 2 1 2 1 1 0 1 2 0 2 0 1 0 1 1 1
1 1 2 1 2 1 1 0 1
TMB1489 1 1 0 1 1 1 0 1 2 1 1 0 1 1 1 0 2 0 1 1 1 2 0 1 2 0 0
2 2 1 1 1 1 2 1 1 2 1 1 0 1 2 1 0 1 1 0 1 1 2 1 1 2 1 2 1 1 2
1 1 2 2 2 2 0 0 2 2 1 2 1 2 2 0 2 1 1 1 0 2 1 1 1 1 0 1 1 2 1
1 2 1 2 1 2 1 1 1 2 0 1 0 1 1 2 1 2 2 1 0 1 2 0 2 0 2 0 1 2 1
1 1 2 0 2 1 1 0 2
BNL3977 1 1 0 1 1 1 0 1 2 1 1 0 1 1 1 0 2 0 1 1 1 2 0 1 2 0 0
2 2 1 1 1 0 2 1 1 2 1 1 0 1 2 1 0 1 1 0 1 1 2 1 1 2 1 2 1 1 2
1 1 2 2 2 2 0 1 2 2 1 2 1 2 2 0 2 1 2 1 0 2 1 1 1 1 0 1 1 2 1
1 2 1 2 0 2 1 1 1 2 0 1 0 2 2 2 2 2 2 2 0 1 2 0 2 0 2 0 1 2 1
1 1 2 0 2 1 1 1 2
TMB1645 1 1 1 2 1 1 1 2 1 1 0 0 1 0 1 0 2 0 1 1 1 2 1 2 0 0 0
1 1 2 1 0 1 2 1 0 2 0 1 0 1 2 1 1 1 1 1 2 1 2 1 1 1 1 2 1 0 2
1 2 2 1 2 2 1 2 2 2 1 2 1 2 2 1 1 1 2 1 1 2 1 2 0 0 0 1 1 1 2
1 1 1 1 0 0 0 1 1 2 0 2 0 1 1 2 1 2 2 1 0 2 1 1 2 1 1 1 1 1 1
2 1 2 2 2 1 1 1 2
CM209 0 1 1 2 1 1 1 2 1 1 0 0 1 0 1 0 2 0 1 1 1 2 1 2 0 0 1 1
```

1 2 1 0 1 2 1 0 2 0 1 0 1 2 1 1 1 1 1 2 1 2 1 1 1 1 2 1 0 2 1
2 2 1 2 2 2 2 2 2 1 2 1 2 2 1 1 1 2 2 1 2 1 2 0 0 0 1 1 1 2 1
1 1 1 0 0 0 1 1 2 0 2 0 1 1 2 1 2 2 1 0 2 1 1 2 1 1 1 1 1 1 2
1 2 2 2 1 1 1 2
JESPR218 1 1 1 2 1 1 1 2 1 1 0 1 1 0 1 0 1 0 2 1 1 2 1 2 2 0 1
1 1 2 1 0 1 2 1 2 1 0 1 0 1 2 0 1 0 2 1 2 2 1 1 1 1 1 2 1 0 2
1 2 2 2 1 2 2 1 1 2 2 2 1 2 2 1 1 2 2 1 1 1 1 2 0 0 0 1 1 1 2
1 1 5 1 1 0 0 2 1 2 0 2 0 1 1 2 0 2 2 1 1 2 0 5 2 1 1 0 1 1 1
2 1 2 2 2 1 1 1 0
CM42 1 1 1 2 1 1 1 2 1 1 0 1 1 0 1 0 1 0 2 1 1 2 1 2 2 0 1 1 1
2 1 0 1 2 1 2 1 0 1 0 1 2 0 1 0 2 1 2 2 1 1 1 1 1 2 1 0 2 1 2
2 2 1 2 2 1 1 2 2 2 1 2 2 1 1 2 2 1 1 1 1 2 0 0 0 1 1 1 2 1 1
1 1 1 0 0 2 1 2 0 2 0 1 1 2 0 2 2 1 1 2 1 1 2 1 1 0 1 1 1 2 1
2 2 2 1 1 1 0
CM3 1 1 1 2 1 1 1 2 1 1 0 1 1 0 1 0 1 0 2 1 1 2 1 2 2 0 1 1 1
2 1 0 1 2 1 2 1 0 1 0 1 2 0 1 0 2 1 2 2 1 1 1 1 1 2 1 0 2 1 2
2 2 1 2 2 1 1 2 2 2 1 2 2 1 1 2 2 1 1 1 1 2 0 0 0 1 1 1 2 1 1
1 1 1 0 0 2 1 2 0 2 0 1 1 2 0 2 2 1 1 2 1 1 2 1 1 0 1 1 1 2 1
2 2 2 1 1 1 0
JESPR236 1 0 1 2 1 1 1 2 1 1 0 1 1 0 1 0 1 0 2 1 1 2 1 2 2 0 1
1 1 2 1 0 1 2 1 2 1 0 1 0 1 2 0 1 0 2 1 2 2 1 1 1 1 1 2 1 0 2
1 2 2 2 1 2 2 1 1 2 2 2 1 2 2 1 1 2 2 1 1 1 1 2 0 0 0 1 1 1 2
1 1 1 5 1 0 0 2 1 2 0 2 0 1 1 2 0 2 2 1 1 2 1 1 2 1 1 0 1 1 1
2 1 2 2 2 1 1 1 0
CM82 2 2 1 0 1 1 2 1 2 0 1 1 1 1 1 1 1 2 2 2 1 1 1 2 1 1 1 0 0
0 0 0 1 1 1 0 1 0 0 1 1 1 0 2 0 1 2 1 1 1 2 0 1 0 1 2 2 1 1 1
1 1 1 2 0 1 1 1 1 1 1 1 0 1 2 2 1 1 1 1 0 1 2 0 1 0 1 0 1 2 1
0 1 1 2 1 0 1 2 1 1 1 1 0 1 1 2 1 1 2 1 2 1 1 1 1 1 1 2 1 1 2 0
1 0 2 0 1 2 1
JESPR235 2 2 1 0 1 1 2 1 2 0 1 1 1 1 1 1 1 2 2 2 1 1 1 2 1 1 1
0 0 0 0 0 1 1 1 0 1 0 0 1 1 1 0 2 0 1 2 1 1 1 2 0 1 0 1 2 2 1
1 1 1 1 1 2 0 1 1 1 1 1 1 1 0 1 2 2 1 1 1 1 0 1 2 0 1 0 1 0 1
2 1 0 1 1 2 1 0 1 2 1 1 1 1 0 1 1 2 1 1 2 1 2 1 1 1 1 1 1 2 1 1
2 0 1 0 2 0 1 2 1
BNL169 2 2 1 0 1 1 2 1 2 0 1 1 1 1 1 1 1 2 2 2 2 2 1 1 2 1 1 1 0
0 0 0 0 1 1 1 0 1 0 0 1 1 1 0 2 0 1 2 1 1 1 1 0 1 0 1 2 2 1 1
1 1 1 1 2 0 1 1 1 1 1 1 1 1 0 1 2 1 1 1 1 1 0 1 2 0 1 0 0 0 1 2
1 0 1 1 2 1 0 1 2 1 1 1 1 0 1 1 2 1 1 2 1 2 1 1 1 1 1 1 2 1 1 2
0 1 0 1 0 1 2 1
GH48 2 2 0 0 1 1 2 1 2 0 1 1 1 1 1 1 1 2 2 2 2 1 1 2 2 1 1 0 0
0 0 0 1 1 1 0 1 0 0 1 2 1 0 2 0 1 2 1 1 1 1 0 1 0 1 2 2 1 1 1
1 1 1 2 0 1 1 1 1 1 1 1 0 1 2 1 1 1 1 1 0 1 2 0 1 0 0 0 1 2 1
0 1 1 2 1 0 1 2 1 1 1 1 0 1 1 2 1 1 2 1 2 1 1 1 1 1 1 2 1 1 2 0
1 0 1 0 1 2 1
BNL119 2 2 0 0 1 1 2 1 2 0 1 1 1 1 2 1 1 1 2 2 2 1 1 2 2 1 1 0
0 0 0 0 1 1 1 0 1 0 0 1 1 1 0 2 0 1 2 1 1 1 1 0 1 0 1 2 2 1 0
1 1 1 1 2 0 1 1 1 1 1 1 1 0 1 1 5 1 1 1 1 0 1 2 0 1 0 0 0 1 2
1 0 1 1 2 1 0 2 2 1 1 1 1 0 1 1 2 1 1 2 1 2 1 1 1 1 1 1 2 1 1 2
0 1 0 1 5 1 2 1
TMB1629 2 2 0 0 1 1 2 1 1 0 1 1 1 0 2 1 1 1 2 2 2 1 1 2 1 1 1
0 0 0 1 0 1 1 1 0 1 0 0 2 1 1 0 1 1 1 2 1 1 1 1 0 1 0 1 2 2 1
0 1 1 1 1 2 0 1 1 1 1 1 1 1 0 5 1 1 1 1 1 1 1 1 1 2 0 1 0 0 1 1
2 1 0 1 1 2 1 0 2 2 1 0 1 1 0 1 1 1 1 1 2 1 2 0 1 1 1 1 2 1 1
1 0 1 0 1 0 1 2 1
GH59 1 2 0 0 1 1 2 1 1 0 1 1 1 0 2 1 1 1 2 2 2 1 1 2 1 1 1 0 0
0 1 0 1 1 1 0 1 0 0 2 1 1 1 1 1 1 2 1 1 1 1 0 1 0 1 2 2 1 1 1
1 1 1 2 0 1 1 1 1 1 1 1 0 1 1 1 2 1 1 1 1 1 2 0 1 0 0 1 1 2 1

0 1 1 2 0 0 2 2 1 0 0 1 0 1 1 1 1 5 2 1 2 0 1 1 1 1 1 1 1 1 0
1 0 1 0 1 2 1
GH54 1 2 0 0 1 1 2 1 1 0 1 1 1 0 2 1 1 1 2 2 2 1 1 2 1 1 1 1 0
0 1 0 1 0 1 1 1 0 0 2 1 1 1 1 1 1 2 1 1 1 1 0 1 0 1 2 2 1 1 1
1 1 1 1 0 1 1 1 1 1 1 1 0 1 1 1 1 1 1 1 1 1 1 2 0 1 1 0 1 1 2 1
0 1 2 2 0 0 2 2 1 0 0 1 0 0 1 1 1 1 2 1 2 0 1 1 2 1 1 1 1 1 0
1 0 1 0 1 2 1
BNL3948 1 2 0 0 1 1 2 1 1 0 1 1 1 0 2 2 1 1 1 2 2 1 1 2 1 1 1
1 1 0 1 0 1 0 1 1 1 0 0 2 1 1 2 1 1 1 1 1 1 1 1 0 1 0 1 5 2 1
1 1 1 1 1 1 0 1 1 1 1 1 1 1 1 1 2 1 1 1 1 1 1 1 2 0 1 1 0 1 1
2 1 0 1 2 2 0 1 2 2 1 0 0 1 0 0 1 1 1 1 2 1 2 0 1 1 2 1 1 1 1
2 0 1 0 1 0 1 2 1
TMB1630 0 2 0 1 2 1 2 0 1 1 0 1 1 0 2 0 2 2 0 2 2 1 2 0 1 1 1
2 1 1 1 0 0 1 0 2 1 0 1 2 1 1 2 2 1 2 1 0 1 1 0 0 1 0 1 2 2 0
1 1 1 1 2 0 1 0 1 1 1 2 1 1 1 2 2 1 1 2 0 1 1 2 1 1 5 2 0 1 1
2 1 1 1 2 2 1 1 2 1 2 1 0 0 1 0 1 1 1 1 1 0 2 0 1 0 2 1 2 1 1
2 0 1 0 1 1 1 1 2
JESPR158 1 1 1 1 1 2 1 1 0 1 2 1 1 1 1 1 0 1 2 2 0 1 2 1 0 0 1
1 0 2 1 1 1 0 1 1 1 2 0 1 1 2 1 1 1 1 0 1 0 1 1 1 2 1 1 2 1 1
1 1 1 0 2 1 0 2 0 1 1 1 1 1 2 2 1 0 1 1 1 1 1 2 1 0 1 2 1 1 2
0 1 1 2 2 0 2 2 2 1 1 0 1 2 2 2 2 2 1 1 1 0 2 1 1 1 2 1 1 5 1
0 0 2 1 0 1 1 2 0
CM23 1 1 1 0 1 2 1 1 0 1 2 1 1 2 1 1 1 1 2 1 0 0 1 1 0 0 2 1 0
2 1 1 1 0 0 1 1 1 0 1 1 2 0 1 1 2 0 1 0 1 1 0 2 0 1 2 0 1 2 1
1 0 2 0 1 2 0 1 1 0 1 1 2 2 1 0 0 1 1 0 1 1 1 0 1 2 1 1 2 0 1
2 2 1 0 2 2 2 1 1 0 1 1 1 2 2 2 0 1 2 0 1 1 1 1 2 1 1 0 0 0 0
2 2 1 0 0 2 1
TMB2038 2 1 1 0 0 2 1 0 0 1 2 1 2 2 1 1 1 1 2 1 0 0 1 1 0 1 1
1 0 2 1 1 1 1 1 2 1 1 0 1 1 1 0 1 2 2 0 1 1 1 1 0 1 0 1 1 0 1
2 1 1 1 2 1 1 2 0 1 2 0 1 1 1 1 0 0 0 1 1 0 1 0 1 0 2 2 1 1 1
1 1 2 2 1 0 2 2 2 1 0 0 1 1 1 1 2 2 1 2 2 1 1 1 1 1 1 2 2 1 1 1
1 0 1 1 1 1 0 2 1
TMB0400 2 1 1 0 0 2 1 0 0 0 2 1 2 2 0 1 1 0 1 1 1 0 2 1 0 1 1
1 1 1 1 1 1 1 1 2 2 1 0 1 1 0 1 1 2 2 0 1 1 0 1 1 2 0 1 0 0 0
1 1 1 1 2 1 1 1 2 1 1 0 0 2 1 1 0 0 0 1 1 0 1 0 1 0 2 2 0 1 1
1 1 2 2 1 0 2 2 2 2 0 0 0 1 1 1 2 2 1 2 2 1 1 1 0 2 1 2 1 2 1
1 0 1 0 1 1 1 2 1
JESPR118 2 1 1 0 0 2 1 0 0 0 2 1 2 2 0 1 1 0 1 1 1 0 2 1 0 1 1
1 1 1 1 1 1 1 1 2 2 1 0 1 1 0 1 1 2 2 0 1 1 0 1 1 2 0 1 0 0 0
1 1 1 1 2 1 1 1 2 1 1 0 0 2 1 1 0 0 0 1 1 0 1 0 1 0 2 2 0 1 1
1 1 2 2 1 0 2 2 2 2 0 0 0 1 1 1 2 2 1 2 2 1 1 1 0 2 1 2 1 2 1
1 0 1 0 1 1 1 2 1
BNL3649 2 1 1 0 0 2 1 1 0 0 2 2 2 2 0 1 1 0 1 0 1 0 2 1 0 1 1
1 1 1 1 1 1 1 1 1 2 1 1 0 1 1 0 1 0 2 1 0 1 1 0 1 1 2 1 1 0 0
1 1 1 1 2 1 1 1 0 1 1 1 0 2 1 0 0 0 0 2 1 0 1 0 1 0 2 1 0 1 1
1 1 2 2 0 0 2 2 2 1 1 0 0 1 1 0 2 2 0 2 2 1 1 1 0 1 1 2 1 2 1
1 0 1 0 1 5 5 5 5
BNL1551 2 1 1 0 0 2 1 1 0 0 2 2 2 2 0 1 1 0 1 0 1 0 2 1 0 1 1
1 1 1 1 1 1 1 1 2 1 1 0 1 1 0 2 0 2 1 0 1 1 0 1 1 2 1 1 1 0 0
1 1 1 1 2 1 1 1 0 1 1 1 0 2 1 0 0 0 0 2 1 0 0 0 1 0 2 1 0 1 1
1 1 2 2 0 0 2 2 2 1 1 1 0 1 1 1 1 2 0 2 2 1 1 1 0 1 1 2 1 2 1
1 0 1 0 1 2 0 2 0
BNL3279 1 0 1 0 1 2 1 0 0 1 2 1 1 2 1 1 1 0 1 0 2 1 2 1 0 1 1
2 2 1 0 0 1 1 1 1 1 2 1 2 1 1 1 0 2 1 0 1 2 0 1 1 0 2 1 1 1 0
1 1 1 2 0 0 1 1 0 1 1 1 0 1 2 0 0 2 0 1 1 0 0 0 1 2 0 1 0 1 1
2 1 1 2 1 0 0 2 1 1 1 1 1 2 2 1 0 1 0 2 0 1 1 2 1 1 1 1 2 1 2
2 1 1 1 1 2 1 0 2

BNL686 1 0 1 2 0 1 1 1 2 0 1 1 2 1 0 2 1 1 2 2 2 0 1 1 1 1 1 2
2 1 0 1 0 1 2 1 0 1 2 2 2 2 1 1 2 1 1 1 1 2 1 0 1 2 1 2 0 2 1
1 0 1 0 1 2 2 1 0 1 1 2 2 1 1 2 1 1 1 0 1 0 1 1 1 0 1 1 2 0 2
0 2 1 1 1 1 1 1 2 2 2 1 2 0 2 1 1 2 1 0 1 1 1 0 1 2 2 1 1 0 2
0 1 1 0 2 1 0 1
TMB1701 1 0 2 2 1 1 0 2 2 0 2 1 2 1 1 2 1 1 1 2 1 1 1 0 2 2 2
2 2 1 0 1 0 1 2 0 1 2 0 2 1 0 1 1 1 1 1 2 1 2 1 0 1 0 2 2 0 2
1 1 1 0 0 2 1 1 1 1 0 1 2 2 1 2 2 1 1 1 1 1 0 1 2 1 0 0 0 1 1
1 0 1 1 0 1 2 1 2 2 2 2 1 0 1 2 2 1 2 1 0 0 0 1 0 0 2 2 0 0 0
1 0 1 1 1 1 1 1 1
JESPR151 1 0 2 2 1 1 0 2 2 0 2 1 2 1 1 2 1 1 1 2 0 1 1 0 2 2 2
2 2 1 0 1 0 1 2 0 1 2 0 2 1 0 1 1 1 1 1 2 1 2 1 0 1 0 2 2 0 2
1 1 1 0 0 2 1 1 1 1 0 1 2 2 1 2 2 1 1 1 1 1 1 2 2 1 0 0 0 1 1
1 0 1 1 0 1 2 1 2 2 2 2 1 0 1 2 2 1 2 1 0 0 0 1 0 0 2 2 0 0 0
1 0 1 1 1 1 1 1 1
TMB1425 1 2 1 2 1 1 0 1 2 0 2 0 1 0 1 1 2 1 1 1 0 0 2 1 1 2 2
2 2 0 1 1 0 1 1 1 1 1 0 2 1 0 1 1 1 2 1 2 2 2 1 0 1 0 2 1 1 2
1 1 1 0 1 2 0 1 1 1 0 1 2 2 1 2 2 1 1 1 2 1 1 2 0 1 0 0 1 1 1
1 0 1 2 0 0 1 1 2 1 2 1 2 0 1 2 0 0 2 1 0 1 1 1 0 1 2 2 0 0 0
1 1 2 2 1 2 0 1 1
TMB0382 1 2 0 2 0 2 1 1 2 0 2 0 1 0 1 1 2 1 1 0 0 0 2 1 2 1 1
1 1 0 2 1 0 2 1 2 1 1 1 1 1 0 1 1 1 2 1 2 2 2 1 0 1 0 1 1 1 2
0 1 1 0 2 2 0 5 1 1 1 1 2 1 1 1 2 1 0 1 2 1 1 2 0 1 0 0 2 1 1
1 0 2 2 1 1 1 1 2 1 0 1 2 0 1 1 0 1 1 1 0 1 1 1 0 5 1 2 0 0 0
1 1 2 2 1 2 0 1 1
JESPR110 1 2 0 1 0 2 1 1 2 1 2 0 1 0 1 1 2 0 1 0 0 0 2 1 1 1 0
1 1 0 2 1 0 2 1 2 1 1 1 1 1 0 1 1 1 2 1 2 2 2 1 0 1 0 1 1 1 1
0 1 1 1 2 2 0 1 1 2 2 1 2 1 1 1 1 1 0 1 2 1 1 2 0 1 0 0 2 0 0
1 0 2 1 1 1 1 1 1 1 0 1 2 0 5 0 0 1 1 2 0 1 1 1 0 2 1 2 0 1 0
0 1 2 2 0 1 1 0 0
TMB0429 1 1 0 2 1 1 0 1 0 1 1 1 0 0 2 1 1 2 1 0 2 0 1 2 2 2 2
2 0 0 1 1 2 5 0 0 1 1 1 2 1 1 1 2 2 0 2 0 1 2 0 1 0 1 0 2 1 2
1 0 0 1 2 1 1 0 1 1 2 0 2 1 0 0 0 1 1 2 0 1 1 0 1 1 2 1 2 2 0
1 1 2 5 1 1 2 2 2 1 1 1 0 1 0 2 0 2 2 1 1 2 1 2 1 0 1 1 1 0 2
1 0 0 1 2 2 1 0 1
BNL2568 1 1 0 2 1 1 0 2 0 1 1 1 0 0 2 1 1 2 1 0 2 0 1 2 2 1 2
2 0 1 1 1 2 1 0 0 1 1 2 2 1 1 1 2 2 0 1 0 1 2 0 1 0 1 0 2 1 2
1 0 0 1 2 1 1 0 1 1 2 0 2 1 0 0 0 1 1 2 0 1 1 0 1 2 2 1 1 2 0
0 1 0 2 5 1 1 1 2 2 1 1 1 0 1 0 2 0 2 2 1 1 2 0 2 1 0 1 1 1 0
2 1 0 0 1 2 1 0 1
GH171 1 1 0 2 1 1 0 2 0 1 1 1 0 0 2 1 1 2 1 0 2 0 1 2 2 1 2 2
0 2 1 1 2 1 0 0 1 1 2 2 1 1 1 5 2 0 1 0 1 2 0 1 0 1 0 2 1 2 1
0 0 1 2 1 1 0 1 1 2 0 2 1 0 0 0 1 1 2 0 1 1 0 1 2 2 1 1 2 0 1
1 2 0 1 1 1 1 2 2 1 1 1 0 1 0 2 0 2 2 1 1 2 5 2 1 0 1 1 1 0 2
1 0 0 1 2 1 0 1
BNL2655 1 1 0 2 1 1 0 2 0 1 1 1 0 0 2 1 1 2 1 0 2 0 1 2 2 1 2
2 0 5 5 5 5 5 5 5 5 5 5 5 5 5 5 5 0 1 0 1 2 0 1 0 1 0 2 1 2
1 0 0 1 2 1 1 0 1 1 2 0 2 1 0 0 0 1 1 2 0 1 1 0 1 2 2 1 1 2 0
1 1 2 0 1 1 1 1 2 2 1 1 1 0 1 0 2 0 2 2 1 1 2 1 2 1 0 1 1 1 0
2 1 0 0 5 2 1 0 1
GH272 1 1 0 2 1 1 0 2 0 1 1 1 0 0 2 1 1 2 1 0 2 0 1 2 2 1 2 2
0 2 1 1 2 1 0 0 1 1 2 2 1 1 1 2 2 0 1 0 0 2 0 1 0 1 0 2 1 2 1
0 0 1 2 1 1 0 1 1 2 0 2 0 0 0 0 1 1 2 0 1 1 0 1 2 2 1 1 1 1 1
1 2 0 1 1 1 1 2 2 1 1 1 0 1 0 2 0 2 1 1 1 2 1 2 1 0 1 1 0 0 2
1 0 0 1 2 2 0 1
BNL1521 2 1 0 2 1 1 0 1 0 1 1 0 0 0 2 1 1 2 0 0 2 0 2 2 1 2 2
2 1 0 1 1 2 2 0 0 0 1 1 2 1 1 1 2 2 1 2 1 1 1 0 1 0 1 0 2 1 2

1 0 0 1 2 1 1 2 1 1 2 0 2 1 0 2 0 1 1 2 0 1 2 0 1 1 1 1 1 0
1 1 1 0 1 1 1 2 1 2 1 0 1 0 1 0 2 1 2 2 2 1 2 1 2 1 0 1 1 1 0
2 1 0 0 1 1 1 0 1
BNL2616 1 1 0 2 1 1 0 1 0 1 1 1 0 0 2 1 1 2 0 0 2 0 2 2 1 2 2
2 0 0 1 1 2 2 0 0 1 1 1 2 1 1 1 2 2 0 2 1 1 2 0 1 0 1 0 2 1 2
1 0 0 1 2 1 1 0 1 5 2 0 2 1 0 2 0 1 1 2 0 1 2 0 1 1 2 1 1 1 0
1 1 1 0 1 1 0 2 2 2 1 0 1 0 1 0 2 0 2 2 1 0 2 1 2 1 0 1 1 1 0
2 1 0 0 1 5 1 0 1
BNL252 1 1 0 2 1 1 0 1 0 1 0 1 0 0 2 1 1 2 1 0 2 0 1 2 2 0 2 0
0 1 2 2 1 0 1 1 0 2 0 2 2 1 1 1 2 0 1 0 0 2 0 1 0 1 0 2 1 2 1
0 0 1 2 1 1 0 1 1 2 0 2 1 0 0 0 1 1 2 0 1 2 0 1 2 2 1 1 2 0 2
1 1 0 1 1 1 0 2 2 1 1 1 0 1 0 1 0 2 1 1 1 2 1 2 1 0 2 1 0 0 2
1 0 0 1 0 2 2 1
GH224 2 1 1 2 1 0 0 1 1 2 2 2 2 1 1 0 1 0 0 0 1 2 1 1 1 2 0 0
1 2 2 2 2 1 0 1 0 1 1 0 1 2 2 2 1 0 1 2 2 0 1 0 1 1 1 0 0 0 1
1 1 0 1 0 1 1 1 1 2 1 0 1 2 0 1 2 2 1 2 2 1 1 0 1 1 1 2 0 1 2
1 1 0 1 2 2 2 2 1 1 1 1 0 1 0 1 0 0 0 0 2 0 1 2 1 2 1 1 0 1 1
0 1 1 2 1 0 2 0
JESPR215 2 1 1 2 1 0 0 1 1 2 2 2 2 1 1 0 1 0 0 0 1 2 1 1 1 2 0
0 1 2 2 2 2 1 0 1 0 1 1 0 1 2 2 2 1 0 1 2 2 0 1 0 1 1 1 0 0 0
1 1 1 0 1 0 1 1 1 1 2 1 0 1 2 0 1 2 2 1 2 2 1 1 0 1 1 1 2 0 1
2 1 1 2 1 2 2 2 2 1 1 1 1 0 1 0 1 0 0 0 0 2 0 1 2 1 2 1 1 0 1
1 0 1 1 2 1 0 2 0
JESPR227 2 1 1 2 1 0 0 1 1 2 2 2 2 1 1 0 1 0 0 0 1 2 1 1 1 2 0
0 1 2 2 2 2 1 0 1 0 1 1 0 1 2 2 2 1 0 1 2 2 0 1 0 1 1 1 0 0 0
1 1 1 0 1 0 1 1 1 1 2 1 0 1 2 0 1 2 2 1 2 2 1 1 0 1 1 1 2 0 1
2 1 1 2 1 2 2 2 2 1 1 1 1 0 1 0 1 0 0 0 0 2 0 1 2 1 2 1 1 0 1
1 0 1 1 2 1 0 2 0
CM27 2 1 1 2 1 0 0 1 1 2 2 2 2 1 1 0 1 0 0 0 1 2 1 1 1 2 0 0 1
2 2 2 2 1 0 1 0 1 1 0 1 2 2 2 1 0 1 2 2 0 1 0 1 1 1 0 0 0 1 1
1 0 1 0 1 1 1 1 2 1 0 1 2 0 1 2 2 1 2 2 1 1 0 1 1 1 2 0 1 2 1
1 2 1 2 2 2 2 1 1 1 1 0 1 0 1 0 0 0 0 2 0 5 2 1 2 1 1 0 1 1 0
1 1 2 1 0 2 0
CM13 2 5 1 2 1 0 0 1 1 2 2 2 2 1 1 0 1 0 5 0 1 2 1 1 1 2 0 0 1
2 2 2 2 1 0 1 0 1 1 0 1 2 2 2 1 0 5 2 2 0 1 0 1 1 1 0 0 0 1 1
1 5 1 0 1 1 1 1 2 1 0 1 2 0 1 2 2 1 2 2 1 1 0 1 1 1 2 0 1 2 1
1 2 1 2 2 2 2 1 1 1 1 0 1 0 1 0 0 0 0 2 0 1 2 1 2 5 1 0 1 1 0
1 1 2 1 0 2 0
NAU3171 2 2 1 2 1 1 0 1 1 2 1 1 0 1 1 1 1 1 1 2 1 2 1 2 0 2 1
0 1 1 2 2 2 1 1 1 1 1 1 1 2 2 1 1 1 0 1 2 2 0 1 0 1 0 1 1 0 0
0 0 1 1 1 1 1 1 0 1 1 1 0 1 2 1 1 2 2 2 1 0 1 1 0 1 1 1 2 1 1
1 2 1 1 2 2 1 2 1 1 0 0 1 1 1 5 1 0 0 1 0 2 0 1 2 0 2 1 1 0 1
1 0 2 2 2 1 0 2 1
NAU4925 0 1 2 1 2 1 0 1 0 0 2 0 1 0 0 1 2 1 0 1 1 0 1 0 0 0 2
1 1 1 2 2 1 0 1 1 1 0 2 2 1 1 0 0 0 1 0 0 1 1 1 1 1 1 0 0 1 1 1
1 2 0 1 0 1 1 1 1 1 1 0 1 1 0 2 1 2 1 1 2 2 2 2 2 1 2 0 1 1 0
1 2 2 0 0 1 1 1 2 0 1 0 1 1 2 1 2 0 1 0 1 1 1 2 1 0 1 2 0 1 0
0 1 1 1 1 2 1 2 1
NAU1119 1 0 2 1 2 0 1 1 1 2 1 2 1 1 0 1 2 0 1 2 1 0 0 0 0 1 1
1 2 0 1 1 0 0 0 1 1 1 1 2 0 2 1 0 1 1 1 0 0 0 2 0 1 0 0 1 1 1
0 2 0 2 1 1 1 0 1 2 1 1 1 0 0 2 0 2 2 1 0 1 0 1 2 1 1 0 0 0 0
0 2 0 1 0 1 1 1 1 0 1 1 2 2 2 1 2 0 1 0 1 1 1 2 2 0 1 5 1 1 0
0 1 1 1 1 2 0 1 1
BNL3510 2 1 2 1 2 1 1 1 1 2 1 2 1 1 0 1 2 0 1 2 1 0 1 0 1 1 1
2 1 1 1 0 0 1 0 1 1 1 1 2 1 2 1 0 1 1 1 0 0 0 2 0 1 1 0 1 1 1
0 2 0 2 1 1 1 0 0 1 1 1 2 0 0 1 1 2 2 1 1 1 0 1 2 1 1 0 0 1 0
1 2 0 1 0 1 1 1 1 0 1 1 2 2 2 1 2 1 1 0 1 1 1 1 1 1 0 2 2 1 1 1

0 1 1 0 0 2 0 1 2
BNL3816 2 1 2 1 2 1 1 1 1 2 1 2 1 1 0 1 2 0 1 2 1 0 1 0 1 1 1
2 1 1 1 0 0 1 0 1 1 1 1 2 1 2 1 0 1 1 1 0 0 0 2 0 1 1 0 1 1 1
0 2 0 2 1 1 1 0 0 1 1 1 2 0 0 1 1 2 2 1 1 1 0 1 2 1 1 0 0 1 0
2 2 0 1 0 1 1 1 1 0 1 1 2 2 2 1 2 1 1 0 1 1 1 2 1 0 2 2 1 1 1
0 1 1 0 0 2 0 1 2
CIR391 2 1 2 1 2 1 1 1 1 2 1 2 1 1 0 1 2 0 1 2 1 0 1 0 1 1 1 2
1 1 1 0 0 1 0 1 1 1 1 2 1 2 1 0 1 1 1 0 0 0 2 0 1 1 0 1 1 1 0
2 0 2 1 1 1 0 0 1 1 1 2 0 0 1 1 2 2 1 1 1 0 1 2 1 1 0 0 1 0 1
2 0 1 0 1 1 1 1 0 1 1 2 2 2 1 2 1 1 0 1 1 1 2 1 0 2 2 1 1 1 0
1 1 0 0 2 0 1 2
BNL840 2 1 2 1 2 1 1 1 1 2 1 2 1 1 0 1 2 0 1 2 1 0 1 0 1 1 1 2
1 1 1 0 0 1 0 1 1 1 1 2 1 2 1 0 1 1 1 0 0 0 2 0 1 1 0 1 1 1 0
2 0 2 1 1 1 0 0 1 1 1 2 0 0 1 1 2 2 1 1 1 0 1 2 1 1 0 0 1 0 1
2 0 1 0 1 1 1 1 0 1 1 2 2 2 1 2 1 1 0 1 1 1 2 1 0 2 2 1 1 1 0
1 1 0 0 2 0 1 2
CIR039 2 1 2 1 2 1 1 1 1 2 1 2 1 1 0 1 2 0 1 2 1 0 1 0 1 1 1 2
1 1 1 0 0 1 0 1 1 1 1 2 1 2 1 0 1 1 1 0 0 0 2 0 1 1 0 1 1 1 0
2 0 2 1 1 1 0 0 1 1 1 2 0 0 1 1 2 2 1 1 1 0 1 2 1 1 0 0 1 0 1
2 0 1 0 1 1 1 1 0 1 1 2 2 2 1 2 1 1 0 1 1 1 2 1 0 2 2 1 1 1 0
1 1 0 0 2 0 1 2
JESPR92 2 1 2 1 2 1 1 1 1 2 1 2 1 1 0 1 2 0 1 2 1 0 1 0 1 1 1
2 1 1 1 0 0 1 0 1 1 1 1 2 1 2 1 0 1 1 1 0 0 0 2 0 1 1 0 1 1 1
0 2 0 2 1 1 1 0 0 1 1 1 2 0 0 1 1 2 2 1 1 1 0 1 2 1 1 0 0 1 0
1 2 0 1 0 1 1 1 1 0 1 1 2 2 2 1 2 1 1 0 1 1 1 2 1 0 2 2 1 1 1
0 1 1 0 0 2 0 1 2
NAU2195 2 1 2 1 2 1 1 1 1 2 1 2 1 1 0 1 2 0 1 2 1 0 1 0 1 1 1
2 1 1 1 0 0 1 0 1 1 1 1 2 1 2 1 0 1 1 1 0 0 0 2 0 1 1 0 1 1 1
0 2 0 2 1 1 1 0 0 1 1 1 2 0 0 1 1 2 2 1 1 1 0 1 2 1 1 0 0 1 0
1 2 0 1 0 1 1 1 1 0 1 1 2 2 2 1 2 1 1 0 1 1 1 2 2 0 2 2 1 1 1
0 1 1 0 0 2 0 1 2
NAU3006 2 1 2 1 2 1 1 1 1 2 1 2 1 1 0 1 2 0 1 2 1 0 1 0 1 1 1
2 1 1 1 0 0 1 0 1 1 1 1 2 1 2 1 0 1 1 1 0 0 0 2 0 1 1 0 1 1 1
0 0 0 2 1 1 1 0 0 1 1 1 2 0 0 1 1 2 2 1 1 1 0 1 2 1 1 0 0 1 0
1 2 0 1 0 1 1 1 1 0 1 1 2 2 2 1 2 1 1 0 1 1 1 2 1 0 2 2 1 1 1
0 1 1 0 0 2 0 1 2
NAU2913 2 1 2 1 0 1 1 1 0 2 1 1 1 1 1 2 2 1 1 1 0 1 1 0 2 2 2
1 1 1 1 1 1 0 1 0 1 1 0 2 1 2 1 1 1 1 1 1 2 0 1 1 1 1 0 1 1 2
1 2 1 1 0 1 1 1 1 1 0 1 2 1 1 1 2 1 0 1 0 1 1 1 2 1 1 0 1 0 0
1 2 0 2 1 2 0 2 1 2 1 1 1 2 2 1 2 1 2 0 2 1 1 2 0 1 0 2 1 1 0
0 1 1 0 2 1 0 2 0
BNL341 2 1 2 1 0 1 1 1 0 2 1 1 1 1 1 2 2 1 1 1 0 1 1 0 2 2 2 1
1 1 1 1 1 0 1 0 1 1 0 2 1 2 1 1 1 1 0 1 2 0 1 1 1 1 0 1 1 2 1
2 1 0 0 1 1 1 1 1 0 1 2 1 1 1 2 1 2 2 0 1 1 1 2 1 1 0 1 0 0 1
0 0 2 1 2 0 0 1 0 1 1 2 2 2 0 2 1 2 0 2 1 1 2 0 1 0 2 1 1 0 0
1 1 0 0 1 0 2 0
NAU2750 2 2 2 1 0 1 1 1 0 2 1 1 1 2 1 2 2 2 1 1 0 1 1 0 2 2 2
1 1 1 1 1 1 0 1 0 1 2 0 2 0 2 1 1 1 1 0 1 2 0 1 1 1 1 0 2 1 2
1 1 1 0 0 1 1 1 1 1 0 2 2 1 1 1 2 1 2 2 0 1 1 1 2 1 1 0 1 0 0
1 0 0 1 1 2 0 0 1 0 1 1 2 2 2 0 2 1 2 0 2 2 1 2 0 1 0 2 2 1 0
0 1 1 0 0 1 0 2 0
NAU4914 2 2 2 1 0 1 1 1 0 2 1 2 1 2 1 2 0 2 1 2 0 1 1 0 2 2 1
1 2 1 2 1 1 1 1 0 1 2 1 2 1 2 2 1 1 1 0 1 1 0 1 1 0 2 2 2 0 2
1 1 1 1 0 2 0 1 1 1 0 2 2 1 2 1 1 0 0 2 1 0 1 1 2 1 1 0 1 0 0
2 0 0 1 1 1 0 0 0 1 1 2 2 2 1 1 1 2 2 0 2 2 2 2 1 2 1 1 2 0 1
1 1 1 1 0 1 1 2 0
BNL3994 2 2 0 0 1 1 2 1 1 1 1 1 0 0 2 1 2 1 1 1 0 0 0 1 1 0 1

```
1 1 2 1 2 2 0 1 2 2 1 2 1 1 0 0 0 2 2 0 2 2 1 1 0 2 0 2 2 1 1
2 0 2 1 1 0 2 2 0 1 1 1 1 2 0 1 0 2 0 0 1 0 0 1 1 1 1 0 0 2 1
1 0 2 2 2 1 1 0 1 1 1 2 2 2 0 2 2 0 2 0 2 1 1 1 1 1 0 0 0 1 1
1 1 0 1 0 2 0 2 0
TMB0120 2 2 0 0 1 1 2 1 1 1 1 1 0 0 2 1 2 1 1 1 0 0 0 1 1 0 1
1 1 2 1 2 2 0 1 2 2 1 2 1 1 1 5 1 2 2 0 2 2 1 1 0 2 0 2 2 1 1
2 0 2 1 1 0 2 2 0 1 1 1 1 2 0 1 0 2 0 0 1 0 0 1 0 1 1 0 1 2 1
1 0 2 0 2 1 1 0 1 1 1 2 2 2 0 2 2 1 2 1 2 1 2 1 1 1 0 0 0 1 1
1 1 0 1 0 2 0 2 0
GH52 2 2 0 0 1 1 2 1 1 1 1 1 0 0 2 1 2 1 1 1 0 0 0 1 1 0 1 1 1
2 1 2 2 0 1 2 2 1 2 1 1 1 1 1 2 2 0 2 2 1 1 0 2 0 2 2 1 1 2 0
2 1 1 0 2 2 0 1 1 1 1 2 0 1 0 2 0 0 1 0 0 1 0 1 5 0 1 2 1 1 0
2 0 2 1 1 0 1 1 1 2 2 2 0 2 2 1 2 1 2 5 2 1 1 1 0 0 0 1 1 1 1
0 1 0 2 0 2 0
GH200 2 1 0 0 1 2 2 2 0 1 1 1 2 0 2 1 2 1 1 1 0 0 0 1 1 1 0 1
2 1 2 2 0 1 2 2 2 5 5 5 5 5 5 5 2 0 2 1 1 1 1 2 1 2 2 1 1 2
1 2 1 2 0 2 2 0 1 2 1 1 2 0 1 0 1 0 1 0 0 0 1 1 1 0 1 1 1 1
0 2 1 1 2 1 0 1 1 1 2 2 2 1 2 1 1 1 1 2 1 2 0 1 1 1 0 1 1 1 0
1 0 1 0 2 0 2 0
-stop markers
-start traits
Buds_number 8.0 39.0 1.0 10.0 26.0 27.0 11.0 21.0 22.0 1.0
12.0 44.0 46.0 32.0 32.0 59.0 61.0 22.0 20.0 0.0 13.0 31.0
25.0 15.0 44.0 47.0 11.0 14.0 34.0 53.0 32.0 10.0 3.0 23.0
10.0 19.0 56.0 41.0 24.0 4.0 107.0 5.0 34.0 78.0 25.0 11.0
16.0 33.0 65.0 0.0 22.0 32.0 25.0 52.0 65.0 1.0 44.0 8.0 13.0
0.0 94.0 19.0 3.0 12.0 50.0 19.0 50.0 34.0 49.0 28.0 1.0 51.0
0.0 31.0 25.0 68.0 34.0 23.0 27.0 20.0 14.0 28.0 14.0 58.0
84.0 25.0 55.0 89.0 68.0 58.0 0.0 31.0 28.0 48.0 19.0 23.0
16.0 96.0 96.0 0.0 109.0 36.0 26.0 2.0 34.0 56.0 35.0 33.0
15.0 58.0 0.0 12.0 70.0 6.0 48.0 0.0 93.0 82.0 44.0 30.0 27.0
0.0 14.0 12.0 64.0 0.0 105.0 32.0 0.0
flowering_time 87.0 56.0 82.0 61.0 50.0 47.0 64.0 61.0 55.0
118.0 58.0 42.0 33.0 50.0 45.0 51.0 40.0 86.0 84.0 0.0 47.0
53.0 46.0 44.0 36.0 52.0 63.0 67.0 44.0 46.0 53.0 58.0 97.0
40.0 50.0 51.0 57.0 40.0 71.0 51.0 46.0 110.0 64.0 46.0 47.0
76.0 63.0 50.0 43.0 0.0 54.0 39.0 55.0 46.0 48.0 111.0 44.0
77.0 75.0 0.0 47.0 73.0 59.0 50.0 50.0 65.0 44.0 50.0 52.0
80.0 99.0 48.0 0.0 53.0 47.0 51.0 43.0 50.0 43.0 67.0 38.0
71.0 60.0 50.0 49.0 44.0 54.0 40.0 50.0 58.0 0.0 44.0 49.0
61.0 84.0 51.0 76.0 36.0 39.0 0.0 49.0 53.0 56.0 114.0 47.0
59.0 64.0 60.0 64.0 57.0 0.0 62.0 60.0 76.0 54.0 0.0 46.0 42.0
46.0 44.0 70.0 0.0 87.0 90.0 49.0 0.0 47.0 78.0 0.0
flowering_frame 17.0 58.0 0.0 58.0 68.0 65.0 51.0 58.0 61.0
0.0 58.0 70.0 85.0 52.0 66.0 68.0 79.0 33.0 34.0 0.0 67.0 66.0
73.0 70.0 83.0 60.0 53.0 48.0 74.0 73.0 67.0 44.0 14.0 71.0
61.0 67.0 62.0 72.0 48.0 62.0 72.0 5.0 56.0 71.0 72.0 44.0
56.0 69.0 76.0 0.0 66.0 73.0 56.0 69.0 71.0 0.0 75.0 38.0 39.0
0.0 73.0 47.0 52.0 69.0 61.0 47.0 70.0 69.0 67.0 39.0 0.0 69.0
0.0 66.0 70.0 69.0 77.0 63.0 74.0 52.0 77.0 49.0 53.0 70.0
69.0 58.0 66.0 78.0 68.0 61.0 0.0 75.0 70.0 59.0 32.0 65.0
43.0 79.0 81.0 0.0 71.0 63.0 61.0 3.0 73.0 56.0 56.0 58.0 48.0
58.0 0.0 57.0 59.0 42.0 65.0 0.0 73.0 77.0 74.0 75.0 49.0 0.0
28.0 28.0 70.0 0.0 72.0 38.0 0.0
Photop_flowering 6.0 3.0 5.0 3.0 2.0 2.0 3.0 3.0 3.0 9.0 3.0
1.0 1.0 2.0 2.0 2.0 1.0 6.0 5.0 10.0 2.0 2.0 2.0 2.0 1.0 2.0
3.0 4.0 2.0 2.0 2.0 3.0 7.0 1.0 2.0 2.0 3.0 1.0 4.0 2.0 2.0
```

8.0 3.0 2.0 2.0 5.0 3.0 2.0 1.0 10.0 3.0 1.0 3.0 2.0 2.0 8.0
2.0 5.0 4.0 10.0 2.0 4.0 3.0 2.0 2.0 4.0 2.0 2.0 2.0 5.0 7.0
2.0 10.0 2.0 2.0 2.0 1.0 2.0 1.0 4.0 1.0 4.0 3.0 2.0 2.0 2.0
3.0 1.0 2.0 3.0 10.0 2.0 2.0 3.0 5.0 2.0 5.0 1.0 1.0 10.0 2.0
2.0 3.0 8.0 2.0 3.0 3.0 3.0 3.0 3.0 10.0 3.0 3.0 5.0 3.0 10.0
2.0 1.0 2.0 2.0 4.0 10.0 6.0 6.0 2.0 10.0 2.0 5.0 10.0
Height 120.0 140.0 30.0 110.0 200.0 100.0 140.0 170.0 190.0
130.0 110.0 90.0 150.0 80.0 100.0 90.0 180.0 190.0 170.0 50.0
160.0 110.0 150.0 130.0 140.0 160.0 160.0 170.0 180.0 100.0
130.0 50.0 120.0 130.0 110.0 100.0 150.0 80.0 120.0 130.0
110.0 130.0 170.0 90.0 130.0 130.0 90.0 110.0 160.0 70.0 120.0
120.0 110.0 150.0 140.0 130.0 200.0 140.0 140.0 30.0 120.0
140.0 100.0 200.0 90.0 70.0 140.0 110.0 150.0 210.0 150.0
140.0 40.0 170.0 160.0 190.0 210.0 170.0 180.0 190.0 140.0
150.0 120.0 190.0 110.0 120.0 150.0 130.0 120.0 160.0 90.0
80.0 100.0 180.0 200.0 100.0 110.0 190.0 160.0 140.0 160.0
140.0 150.0 135.0 135.0 120.0 100.0 160.0 110.0 80.0 80.0
200.0 110.0 180.0 150.0 60.0 170.0 130.0 150.0 110.0 160.0
130.0 150.0 160.0 150.0 120.0 120.0 145.0 110.0
hs 17.0 7.0 ? 10.0 10.0 ? 10.0 13.0 8.0 27.0 8.0 7.0 6.0 7.0
5.0 6.0 5.0 25.0 8.0 ? 7.0 10.0 7.0 10.0 5.0 10.0 10.0 10.0
6.0 6.0 10.0 ? 17.0 7.0 7.0 10.0 8.0 5.0 10.0 ? 6.0 13.0 ? 6.0
? ? 5.0 8.0 ? ? 10.0 5.0 5.0 7.0 ? 9.0 6.0 14.0 10.0 ? 9.0 9.0
11.0 6.0 6.0 9.0 6.0 6.0 7.0 7.0 19.0 9.0 ? ? 9.0 10.0 ? 10.0
8.0 10.0 5.0 ? 14.0 10.0 ? ? ? 7.0 10.0 ? 17.0 8.0 6.0 8.0 ?
9.0 13.0 7.0 10.0 ? ? 6.0 13.0 27.0 6.0 12.0 13.0 8.0 12.0 ? ?
? 8.0 10.0 9.0 ? 6.0 6.0 8.0 6.0 9.0 29.0 ? 9.0 8.0 28.0 6.0
10.0 ?
Monopod 10.0 5.0 ? 6.0 0.0 ? 4.0 4.0 4.0 4.0 0.0 3.0 0.0 0.0
2.0 5.0 3.0 6.0 5.0 ? 1.0 5.0 0.0 4.0 4.0 5.0 0.0 7.0 1.0 3.0
6.0 ? 2.0 0.0 3.0 4.0 2.0 2.0 6.0 ? 4.0 7.0 ? 6.0 ? ? 2.0 2.0
? ? 2.0 3.0 0.0 5.0 ? 2.0 0.0 9.0 5.0 ? 7.0 3.0 5.0 0.0 3.0
2.0 3.0 1.0 5.0 2.0 6.0 3.0 ? ? 3.0 5.0 ? 4.0 1.0 3.0 1.0 ?
7.0 7.0 ? ? ? 4.0 6.0 ? 0.0 6.0 1.0 8.0 ? 4.0 1.0 3.0 11.0 ? ?
1.0 6.0 24.0 3.0 10.0 8.0 4.0 3.0 ? ? ? 5.0 7.0 7.0 ? 3.0 6.0
3.0 1.0 4.0 3.0 ? 5.0 4.0 24.0 4.0 3.0 ?
Simpod 10.0 36.0 ? 18.0 36.0 ? 26.0 22.0 36.0 3.0 26.0 16.0
36.0 22.0 24.0 14.0 40.0 22.0 26.0 ? 36.0 24.0 30.0 24.0 36.0
30.0 26.0 30.0 32.0 26.0 20.0 ? 10.0 28.0 28.0 24.0 30.0 16.0
26.0 ? 24.0 18.0 ? 18.0 ? ? 22.0 21.0 ? ? 26.0 22.0 26.0 26.0
? 14.0 36.0 22.0 24.0 ? 22.0 24.0 16.0 34.0 21.0 12.0 41.0
20.0 28.0 40.0 16.0 28.0 ? ? 38.0 32.0 ? 28.0 36.0 32.0 12.0 ?
12.0 30.0 ? ? ? 28.0 26.0 ? 7.0 20.0 22.0 26.0 ? 21.0 16.0
27.0 24.0 ? ? 34.0 18.0 6.0 34.0 14.0 16.0 28.0 21.0 ? ? ?
21.0 30.0 24.0 ? 32.0 20.0 32.0 24.0 28.0 3.0 ? 26.0 26.0 7.0
30.0 32.0 ?
No_Nodes 26.0 42.0 ? ? 45.0 ? 35.0 34.0 43.0 29.0 33.0 22.0
41.0 28.0 28.0 19.0 44.0 46.0 33.0 ? 42.0 33.0 36.0 33.0 40.0
39.0 35.0 39.0 37.0 31.0 29.0 ? 26.0 34.0 34.0 33.0 37.0 20.0
35.0 ? 29.0 30.0 ? 23.0 ? ? 26.0 28.0 ? ? 35.0 26.0 30.0 32.0
? 22.0 41.0 35.0 33.0 ? 30.0 32.0 26.0 39.0 26.0 20.0 46.0
25.0 34.0 46.0 34.0 36.0 ? ? 46.0 41.0 ? 37.0 43.0 41.0 16.0 ?
25.0 39.0 ? ? ? 34.0 35.0 ? 23.0 27.0 27.0 33.0 ? 29.0 28.0
33.0 33.0 ? ? 39.0 30.0 32.0 39.0 25.0 28.0 35.0 32.0 ? ? ?
28.0 39.0 32.0 ? 37.0 25.0 39.0 29.0 36.0 31.0 ? 34.0 33.0
34.0 35.0 41.0 ?
No_Bolls 2.0 3.0 0.0 0.0 28.0 5.0 7.0 8.0 8.0 0.0 1.0 18.0

```
14.0 10.0 8.0 12.0 0.0 5.0 6.0 0.0 8.0 12.0 4.0 3.0 10.0 26.0
2.0 10.0 28.0 12.0 14.0 2.0 3.0 12.0 2.0 8.0 24.0 5.0 0.0 0.0
47.0 0.0 12.0 24.0 10.0 0.0 10.0 14.0 21.0 0.0 18.0 7.0 21.0
22.0 6.0 0.0 28.0 0.0 4.0 0.0 34.0 0.0 1.0 4.0 24.0 12.0 22.0
17.0 24.0 20.0 3.0 14.0 0.0 10.0 8.0 10.0 5.0 5.0 14.0 1.0 7.0
3.0 6.0 18.0 6.0 2.0 28.0 36.0 26.0 16.0 1.0 4.0 5.0 17.0 0.0
10.0 8.0 34.0 30.0 0.0 6.0 16.0 2.0 0.0 15.0 18.0 18.0 22.0
8.0 0.0 0.0 0.0 26.0 4.0 26.0 0.0 36.0 20.0 26.0 13.0 18.0 0.0
0.0 7.0 22.0 0.0 52.0 22.0 0.0
No_Open_Bolls 0.0 1.0 0.0 0.0 1.0 2.0 0.0 0.0 0.0 0.0 0.0 2.0
0.0 2.0 1.0 5.0 0.0 0.0 0.0 0.0 1.0 0.0 0.0 0.0 1.0 0.0 0.0
0.0 0.0 0.0 0.0 0.0 0.0 2.0 0.0 2.0 0.0 2.0 0.0 0.0 6.0 0.0
0.0 2.0 0.0 0.0 0.0 0.0 1.0 0.0 2.0 1.0 0.0 1.0 0.0 0.0 6.0
0.0 0.0 0.0 1.0 0.0 0.0 1.0 2.0 1.0 2.0 2.0 2.0 0.0 0.0 2.0
0.0 0.0 0.0 0.0 1.0 1.0 1.0 0.0 4.0 0.0 0.0 0.0 0.0 0.0 0.0
3.0 1.0 0.0 0.0 0.0 3.0 1.0 0.0 1.0 0.0 7.0 0.0 0.0 0.0 4.0
0.0 0.0 0.0 0.0 0.0 0.0 0.0 0.0 0.0 0.0 0.0 0.0 0.0 0.0 4.0
0.0 1.0 1.0 0.0 0.0 0.0 0.0 5.0 0.0 5.0 0.0 0.0
Anthocyan 2.0 2.0 ? 1.0 3.0 2.0 3.0 3.0 3.0 1.0 1.0 2.0 1.0
1.0 1.0 2.0 3.0 2.0 1.0 1.0 2.0 2.0 3.0 2.0 3.0 2.0 3.0 2.0
3.0 3.0 1.0 1.0 2.0 2.0 1.0 1.0 3.0 2.0 3.0 2.0 3.0 1.0 ? 3.0
3.0 3.0 2.0 3.0 3.0 ? 3.0 2.0 3.0 3.0 3.0 1.0 3.0 3.0 3.0 ?
2.0 2.0 1.0 3.0 3.0 2.0 2.0 3.0 2.0 3.0 2.0 2.0 ? 3.0 3.0 2.0
2.0 3.0 3.0 3.0 3.0 3.0 2.0 3.0 2.0 2.0 3.0 2.0 3.0 3.0 2.0
3.0 3.0 3.0 ? 2.0 2.0 3.0 3.0 3.0 3.0 3.0 2.0 3.0 2.0 2.0 2.0
2.0 3.0 3.0 3.0 3.0 2.0 3.0 3.0 ? 2.0 3.0 2.0 3.0 3.0 3.0 3.0
3.0 3.0 3.0 3.0 3.0 3.0
Stem_hair 3.0 3.0 ? 1.0 1.0 1.0 ? 1.0 1.0 2.0 1.0 2.0 2.0 1.0
1.0 1.0 1.0 1.0 1.0 1.0 4.0 2.0 1.0 1.0 1.0 4.0 1.0 4.0 1.0
1.0 1.0 1.0 1.0 1.0 1.0 1.0 1.0 1.0 1.0 1.0 1.0 1.0 ? 1.0 1.0
1.0 1.0 1.0 1.0 ? 1.0 1.0 1.0 2.0 1.0 1.0 1.0 2.0 1.0 ? 1.0
1.0 1.0 1.0 3.0 1.0 2.0 1.0 3.0 1.0 2.0 1.0 ? 1.0 3.0 3.0 3.0
1.0 1.0 1.0 1.0 1.0 3.0 1.0 3.0 3.0 2.0 1.0 1.0 3.0 1.0 1.0
1.0 1.0 ? 3.0 2.0 2.0 1.0 4.0 1.0 1.0 2.0 1.0 1.0 4.0 1.0 1.0
1.0 1.0 1.0 1.0 1.0 1.0 1.0 ? 1.0 1.0 3.0 1.0 1.0 1.0 3.0 1.0
1.0 1.0 3.0 1.0 1.0
-stop traits
-quit
-end
```
