# Supplementary material for: QTL mapping for flowering-time and photoperiod insensitivity of cotton Gossypium darwinii Watt
Source: PLoS One. 2017 Oct 9;12(10):e0186240. doi: 10.1371/journal.pone.0186240 (PMC5633191; doi:10.1371/journal.pone.0186240)
Supplement: S3 Data — (PDF) [file pone.0186240.s003.pdf]

```
[Header]
Study name Photoperiodic flowering
Mating string S
Genotype symbols 12345-
Parent1 Darwinii
Parent2 Mutant

[Locus]
TMB1181 15 0.000
1322232---332122-1-3221331323221211333222111322111232323122123332333331331313311
1333322-22222311111233132313111331213112211223313112121
BNL786 15 1.964
132223213233212211231213313232212113332221113221112323231-2-233323333313313111311
1322322222222311111233132313111331213112211223313112121
BNL1350 15 20.858
1122232132331122112112133111322121113132211112213323212112111131233233123112222 2
2323321212221311111121112313123332213131213222322222121
BNL3902 15 25.635
1122232132132122112112133112322121113132211112213323212113111131232233123112222 2
2322321212221311111121112313122322213131323222322221121
TMB0375 15 27.948
11222121321321 22-3211213311232212 22-33322111122133232121131111312322331231 1222-2
2323321212211111111311123131223222131313232223222221121
BNL4082 15 35.633
1122212132132122111111111111322121113332111112213323212113 1-1131112113133112232 2
2322321212221311111311123131223222131313232233232221121
JESPR298 15 41.203
3322212132132122111111111111322121313332111112213323212113111131112113133113331 2
1323321212331311111311123131323232131313232233133331121
JESPR180 15 41.717
3322212122132122111111111111322121313332111112213323212113111131112113133113331 2
1323321212331311111311123131323232131313 23-23323331121
TMB0301 15 44.212
3322212122132122111111111111322121313332111112213323212113111131112111111111331 12
1323321212331211111111123121323232111313232211 23331121
TMB0201 15 49.926
3322-32-2213-122--1--1-11-1132212131332211111221222321211111131112113-3311---32
133--222223313-11-12211---1333232-2121313212-333333122-
TMB1664 15 53.589
3322233132132322111111111111322121213322111112112223211113 1-113111211313311333 32
13123222223313111-123111231332232321313131122333333 1223
TMB1660 15 54.480
3322233132132122111111111111322121213322111112112223211113111131112113133113313 2
13323233223313111112311123133323232131313112233333 31223
JESPR152 15 68.216
1322223132212222111111111111223212111121113123122212111131111311121131331131232
3313322223331312111231132333322323122333333132331313 1113
BNL2920 15 71.016
33--231122332-------222--222----212133221111211222321 1----111-11----3221222233 2
13232--22-33--1-11123111231333 2-2321313131122133333 1223
BNL542 05 0.000
233122123312 3--21133323-13332--311212222321332121312313111111111 23121-1221133221
21-13-111132133-33--22331113231123213122131222222112232
JESPR241 05 11.007
23312212331231321133123-1333231311212222321333121332213111 1-111123121333-11131-2
11-13-111131113-333-123311112311232111221212213132222 32
NAU2140 05 26.071
2331221223123132111111111111233211212222131313111322211111 1-11111111121321113312
1111111111331333133132331113131123213122121122213222313
GH211 05 32.830
2331222231123132111111111111233211112222133113213222212111111111111111121221113312
3111111111331332333132331113331123213123121122213222313
TMB0191 05 37.058
2321222221123132111311111111233211112222113113213212212131 1-1111111112123111231 3
3111111112221331331132331113331123213113121122213222313
NAU2001 05 37.859
2331222231123132111111111111233211112222113113213222212111111111111111121231113313
3111111112221331331132331113331123213113121122213222313
```

NAU2296 05 38.549
2321222221123132111111111111123321111222211311321322221211111-111111111121231112313
31111111122213313311323311133311232131131211222132223l3
NAU3569 05 39.268
23212222311231331111111111111233211112222113113213222212111111111111121231112313
3111111112221331331132331113331123213113121122213222311
BNL3995 05 39.635
2321222221123133111111111111123321111222211311321322221211111-11111111121231112313
31111111122-1331331132331113331123213113121122212222311
NAU5149 05 41.557
23212222211231331111111111111233211112222113313213222212111111111111133212311123l2
2111311112221331331132331113331123213113121122213222311
JESPR65 05 55.921
21213232321231331111111111121213233131322111113213222232322-2-231111111313333l2213
3111111112221111111113213-11123112321311312112231322221 2
BNL252 24 0.000
1213312132112133111211111111333233l-111312221322332231233133131111111133331322222
21-1111113223323222132231-13121122213111l-122332322232-
GH272 24 11.936
121233213211213332232323311113123312311312222322332231233133131133133233313222222
2111111313223223222122221112121122213112131223322222333
BNL2655 24 19.333
1232332112112113323323231111131233123113122223223322312331331321332332333112222 2
2311111311221223222122223132123322233212333213322222323
GH171 24 20.107
-23233213211211332332323311113123312311312222322332231233133-321332332333132222 2
2311111311221223222122223132123322233212333213322222323
BNL2568 24 20.699
12323321121121133233232331111312331231131222232233223123313113213333323331322222
231111131122122322212222-132123322233212333213322222323
TMB0429 24 23.776
12323321321121123233232331111312331231131222232233223123313113213313133331322222
2311111311221222222122223132123322233312333212222222123
BNL2616 24 31.112
22323321121131123333222331111312331331131222232231 22-1233111332133131333313 22222
2311311112221222222132223332123322232323333213223222333
BNL1521 24 36.216
22223321321133123333222331112312331331121222232231221323311133233313133331322222
2313311111333222222132223332123322232323333213223222123
NAU2750 26a 0.000
311113233112131122221222212311111133121222121221212112332 2-332122222212221 22222
2211232322221221221321312222322222223221212112222223113
BNL341 26a 11.790
33111323111213113231333333133111111331211221312232121123322333213322221223122222
2222233-22221221221 3213-2222322222223221212212222223122
NAU2913 26a 25.313
33111321111213111231131311133122232331211231312332121123122333213322221223122222
2222233322211221221323112222122221113221212212121223123
JESPR32 16a 0.000
133232133321321111233333223133112221131231133211131133121132213333233333323322222
2313322111222311111221133232121122212333332132222221222
JESPR128 16a 5.362
1-32321-3-2132111123-333223133112221131231133211131133121 13-213333233333323322222
2311322222222311111221133232121122212313333132222221111
JESPR237 16a 5.477
133232133321321111233333223133112221131231133211131133121132213333233333323322222
2313322222222311111221133232121122212313333132222221111
TMB1271 16a 5.477
133232133321321111233333223133112221131231133211131133121132213333233333323322222
2313322222222311111221133232121122212313333132222221111
GH2 16a 5.668
133232133321321111233333223133112221131231133211131133121132213333233333333322222
2313322222222311111221133232121122212313333132222221111
JESPR297 16a 6.086
133232133321-211112333-322313311222113123113321113113312113221333323333332332222 2
231332222222231111122111 3-32121122212313323132222221111
TMB1409 16a 11.365
133232113-2132111123323322333311222111231333211131133121 13-213333213333323322222

23113222222222231121221132233121122212113333122322221111
BNL3008 16a 13.382
1332321133213211112332333233331122211112313332111311331211322133332133333332222
23113222233322111212213332331211222121133331223222221111
BNL2734 16a 14.148
1332321133213211112332333233331122211112313332111311331211322133332133332332222
23113222233322111212213332331211222131133331233222221111
JESPR270 12 0.000
3122211132112122233331331321222323331112323122311123213332332322212313133333233
32222222211123312312332322223222232232132122132233133 23
NAU1237 12 4.582
31222121321121222332313313232222323131112323122211123212332 3-2322212313133333222 2
3222222221112331221233232222322223223213212213223312223
BNL3835 12 5.519
3122212132112122233231331323222323131112323122211123212332 3-2322212313133333222 2
3222222221112331221233232222322223223213212213233312321
NAU1278 12 23.623
2122212132122122231222131323221322131112323112211122222312322122212113133333221 1
3322223331333231223131222222121121223213212123223232321
NAU943 12 42.384
21222221322322-21111-311112122222233112213311222112221231233312311211313223 22223
3222222322223221333131222321122221223211212233333331321
BNL1679 12 52.714
2122222132232222111111111212222223211221331122211222222222222222112113232222223 3
3222222322222223333123322223132221223211212232333333221
CIR148 12 55.854
2222222132232222111121111121222222321122133112221122222222222222211211321222222 33
3222232322222223133123322223132221223211212232131333221
NAU5047 12 68.768
2222222132232222131323232321222222321122333332222222222222222222212223323222222 22
2222212122222223133123322223122222223211212212323223221
CM85 12 80.559
2233322---23---3-21-2-232-212332121233233333332222223232--2-22212223121322 22-22
22222-2322222323 1-3--332-2--1222--22121--12--332122-22-
GH224 25 0.000
1122231322113322123223223333122122121112121132122221211312233223222313333331332 3
3222221321113332112333132-23212213323-2-23231331331223-
JESPR215 25 1.491
11222313321123221232232232331221221211121211321222212113122-3223222313333331332 3
3222221321123332112333112223212213323222232313313312233
JESPR227 25 2.550
1122231-3-1123221232232232331221221211121211321222212 1131-2-32232223133333313323
3222231321113332 1-23331112232--2-332-2--232313313312233
CM27 25 3.713
1122231-32112322123223223233122122121112123132322221211 3122-32232223133333313323
3222221321113332112333112222212213 32-222232313312312233
CM13 25 5.566
1122231333112322123223223233122122121112121132122221211313233223222313333331332 3
3222221321113332112333113232212213323222232312212312233
TMB0809 04 0.000
1222233322322122121123323331122122211122133222211121231212233323112313333331323 3
3222222221112321323233332223212223223223222233323323222
GH117 04 21.568
-2313221332-333232112313333112112321222311322321113113 22-22333231-23133-33311311
1222222-2111232132123133-2232122-11232-1222233313112222
BNL2572 04 46.969
1223322131223222321323133321321323211123232223213133332313133332111213333331131 1
1223122222222223333231333333211311112221323313313112222
JESPR296 11a 0.000
2322132331313133113133133111333333233323311213211331322212133131323133333112222 2
2323311121113111111311132133233222323113332233232222 322
PhyA 11a 3.135
23--112331-13-----3133133311-3--3323332331121321133132 2-1-13-1313-3-333331122222
232321-11-1-1-1-1111333332133233 2-2322113332233232223 23
NAU1014 11a 15.498
1332312131132111113123133311133232233113311332111331332 2131-2133323333333122222
232231111211-31111113133123322332223221133222332322 2222
TMB0064 11a 16.862

1332312131132111113123133311133232231113311332111331332213132133323333332312222
23233111121113111111313331233223322232211332223322222222
BNL625 11a 26.726
123223213123221111121111111133232231313111312123331332211122133113-133223122222
23223111121113111111313321332233222322113322233222-222
TMB0359 11a 30.540
1332231131232211111211111111332322313331113121231313312131221331131133223122222
232331111211131111113123123322332223221133222332222-22
JESPR236 19a 0.000
12222311311-----23112331332212222332111231131233332221231331223132232333333311311
13213123122222121122311332331111213332122332133131113121
JESPR218 19a 2.059
1222231132212122231123313322122223321112311312333322212313313231322323333333311311
1321312312222121-22311332331111213-3212233213313113121
CM3 19a 3.222
1222231132212122231123313322122223321112311312333322212313313231322323333333311311
1322112312222121122311 3-2-31111213332122332133131113121
CM42 19a 3.229
12222311322121222311233133221222233211123113123333222 1231-3-3231322323333333311311
132211231222212112231 13-233111121333212233213313113121
CM209 19a 24.264
2222231-3-21212223312331 3-12222221311132311312132322212113 1-1121122323133112 2222
232-1123122212121122311132331211223332122332133232 23121
TMB1645 19a 28.617
2222231132312122233123313312222221311122311312112322212 1131-112112232313311 22222
2322112332221212112231113233221122333212232213323223322
JESPR119 06 0.000
2113231132132322222212222212222221111132233322123323211 1131-1111221222222-233213
3222323112221221121323313322211322222131323213231312321
GH32 06 19.803
23222311323323222222122222223222211111322222221212222111111-11112212222123113331 3
3112122211111122222333321132211122223231132122321233 1121
TMB1538 06 22.240
232223113233232222221222221232222111113222222212122221 11111-111122122212311333 13
3122122211111122222333331132211112222323113212232123 31121
GH39 06 24.937
2322231132332322222212222212322221111132222322121222211111111111221222123113331 3
31311221111112222233333133221111121323113212222123 31121
TMB0154 06 29.001
232223122233232222222-22-1232222111113222211212122221111111111122122212311333 13
312132211111122222333331332211112 2233-1-32122321-33112-
GH82 20a 0.000
1311123111211211222212222232111313333331233221-2131112313212332222222222 1223211311
12222111111132323221233322221122111232222322222 12111121
BNL4108 20a 11.594
23111221112212112222122222322113133311212332212213131221321-3322222222212 23323221
1222211111132323221233322231122121232222322223 32232121
TMB1277 20a 11.969
2311122111221211222212222232213313331121233221221313122132133322222222212 23323221
1222211111132323221233322231122121232222322223 32232121
BNL2884 20a 28.659
23133221122232222222122222322313131311222333232331233221321333222222233323 32-221
32222111122222323221222322231122232232222122233 32232121
GH246 11b 0.000
3211111311321311313322323122211212221311212131121111121223222232131223233223222 3
3213311113112231312 1-2233-322333232222313331232232 33232
GH74 11b 3.917
321112133122121131332222312221121222131121233112111112122322223213122322222232 223
32133111131123313121322331322333232222313331232222 33232
BNL3411 11b 4.486
321112133122121131332232312221121222131121233112111112122322223213122322222232 223
32113111131123313121322331322333232222313331232222 33232
TMB1745 10 0.000
2123333132322332331312113121332332321112332133131133323223222222211233233221131 1
1333311113212211312131131133213231232111332323313111211
BNL1665 10 10.870
2123333132313232331312313121332332321112332133132233323223222222211223233221121 2
1333311113222222322131133333233233232331332323313311222

CM67 10 20.111
2123232---11222233131231112332232222111233213213222322322-2-2222211323222-213212
3333311112222222222213113-333232233232331332332312113222
TMB0380 10 21.859
21222321321122222231312-11123322322222111233213213222322322122222221332322222213212
33333111122222-23221311-3-33232233232331332-32312113221
TMB0325 10 30.595
132221313211212233133231112332232222111223213212322322222122222231112322322213212
3323311112222222232213113-3332322232321-1233232312112221
TMB0307 10 30.595
132221313211212233133231112332232222111223213212322322222122222231112322322213212
3323311112222222232213113-3332322232321-1233232312112221
BNL2872 10 30.595
132221313211212233133231112332232222111223213212322322222122222231112322322213212
3323311112222222232213113-3332322232321-1233232312112221
BNL2705 10 31.057
1322213132112122331332311123322322221112232132123223222221222222311123223221-212
3322311112-2222232213113233323222323213123323231211221
PhyB2 10 40.376
13222131221123223313323-113332232212111222213232112322222-2222223111232232211 3-1
13222-3322222223222-3113233321222123213123323231211221
PhyB 10 40.376
13222131221123223313323-113332232212111222213232112322222-22222231112322322113-1
13222-3322222223222-3113233321222123213123323231211221
JESPR158 21 0.000
1113133331223232333223123223113132123131311131131133222221222222333132222222223233
121121312111221311132332231323332223333331312231231221 2
CM23 21 16.340
111332313122322231321312322333113212213131112113113122222 22-222233211222222223233
3232213121112212112123333222232222222223121222221233221 2
TMB0400 21 18.847
31133221332322333313113313113333312131313321313111332131111111213133321231111311
1313313321111212312333333333212331212131333232213113313
JESPR118 21 18.847
31133221332322333313113313113333312131313321313111332131111111213133321231111311
1313313321111212312333333333212331212131333232213113313
TMB2038 21 39.071
3113322332213222331311331321331132223111332131331133222222222223122322332211311
1212213321112212212133332223212221222231212233213112213
BNL3649 21 47.558
1113312113332133311311331321133332223111222131311131223221222222313332231221111 1
1313213321112212212333232233212331233131333233213113311
BNL1551 21 52.893
131331213333213331131133132113333222311122213131113122322122222231333222322 11311
1312222221112212212233232233212331232131331113213113311
TMB1489 19b 0.000
22232321321323221333231323112223212111221121222333222121131111212112321331121322
131131233322123233213113133321133311221212213332333221 2
BNL3977 19b 1.998
22222321321323221333231323112223212111221121222333222121131111212112331131121322
131131233322123233213113133121133311321212213312333221 2
BNL3875 19b 17.560
32222321321223222333221321133223232111221121221333222233233-132323123332231213 23
13113321332232333321232311332313131122221221323233322 12
BNL4096 19b 24.274
32222321321223222333221321133223232111221221221332222233233-13232312333223121123
13113321311132232231212311312233131122221221321233322 12
TMB0366 19b 26.997
32222321221--322-33--2-32--31233232-11221221-2113222223-23331323231 23332-3121-23
1313332-11111-2322-1-1231-3122331-11222212-112123332212
BNL852 19b 39.060
32222121221221221111211121113221233111321221121322222233233313211111 13333121123
1313311111113322222131221131222313113222122123121322212
TMB0189 19b 40.795
322221212212212211112111111132212331113212211213222222132333132311111 3333121123
1312311111113322222131231131222313113222122123121322212
GH109 19b 43.639
3222212132122122111121111111322323311132122112132222223323321323111113333121123

```
1313311111113222222121231133222313112222122122323322212
NAU3935 19b 59.882
31222121321221221111111111211221223211221223321322222232232-222211111222322221321
1322211111112222222121233133222313112222112222322323232
BNL285 19b 72.529
311221313212212211111111112112212132232212211211222222322322222211111222222212212
2322211111112222222221-33-33212313312223112222312313232
TMB1599 19b 98.270
311112331122131111111111112111111232133112211111221212322222222221111132232233212
23222321122222222222131233333112333332223112223312313332
GH71 19b 100.556
2111123-1122131111111121112111111232133112211111221212322222222221111132332233-12
23222321122222222222131233133112333332223112223313313332
TMB1421 01 0.000
33222112223222223121233311133223212122123223321211232111121-11313213331231113333
2333211333331222222331131312223312232331133333232122212
JESPR289 01 13.026
232223122232-222222222-22-122223212122122333221211222111121111312212231231111311
1332213331111232122331131312213311232-13133332212113212
TMB0062 01 27.270
22222312223223222222222222122221212122122333221121222111121111312212221321122222
23322333-3331232122321111312223312232133123313223223212
BNL3888 01 43.119
232223122223212221331231321322212321221213333211212222112231133333113123323322222
2333213333333233123331111212223312233133123313223223212
CM92 01 46.077
2322231222232122213312-132112221232122121333321121222311223113333133123323322222
233331323333321112233111-212223312233133123313223223212
BNL3580 01 48.338
23222312222121222133133132112221232322121333321121222313223-13333333123333322222
2333313233333211122331111212223312233133123313223223212
GH200 26b 0.000
32223323331133332111-3-1---311333212333332323323112332222222222221123122222211331
1212222221222223223221132222112221322212222112232333323
TMB0120 26b 11.949
322222333323323121112321122311213232213132323133333332322222222221123122222211311
1212222221222323223221132222112221322212232312212112121
GH52 26b 12.496
322222332323323121112321122311213232213132323133333332322222222221123122222211311
1212222221222323233221132222112221322212232312212112121
BNL3994 26b 14.036
32222233322332332111232112231121123221113232313333333232222-222211231222222211311
1212222221222323233221132222112221322212232312212112121
TMB1989 03 0.000
2222231222232222131323-3231122222333332231233213-22222132333-1311212322222111311
1232211122212222-2132232213112311133331131122312111233
BNL1379 03 16.978
22222212222321221213231222112223221233322222321311222213222323211223332322311311
1222211112221222222132132223212221123232222212312111222
BNL3441 03 22.535
222221122-112--21313-3-222-122232212333221323213112222132-2-21211223332322311-11
122221111222122233213213-22311222112323222221-312111223
CM106 03 22.867
22222112221121221313231222112223231233322132321311222213222321211223332322311311
1222211112221222332132132223112221123232222212312111223
TMB0564 03 24.802
32222112221121221313231222111223231233322132321311232213222321211223332322311311
1222211112221232332132132223112221123232222212312111223
JESPR165 14 0.000
322313222-2222322222-222223222323223222222112322233311233-1-23122212221221333233
11131-222322133322122333111213111311311-121322232321121
BNL3502 14 9.758
32231322332222321322113333313232322322222211332223331123311223131212231221333233
11131222232223132221233331113131123313112121322332321122
TMB0803 14 15.695
3223132233223232132221-333313332322322222311332223331123311323131212231321333-33
111112222322333222123333331213113331333-131322233321122
NAU2336 14 28.815
```

322332221-22333212322333332132323223222233133322223313233-2-33133212231331133233
111312222322231322122131-112131112113131131322213332333
JESPR110 23 0.000
223332113221-233233322-3332221231322111322212313112212111333333333313333332111211
113113113333132322313333--232111212-1121321313313112212
TMB0382 23 21.000
2323121222232231233322233322212323223313232121131123121132-333233213331332311111
12-22111313313333-31-331--23212221223231222333113112212
TMB1425 23 36.794
222312122222-2312333223333222112232333232321211333223212323333233123321332311-11
32-2211131333333233121332223222222223221232313333232212
BNL3065 16b 0.000
1211123311211211112223333233111-122113213131313111111111312333133313322221312333
3111133323221321131321131112211122212112131122222122113
TMB2036 16b 8.608
-3111223112112111132333332331113122113213131312111111321312333133213322231332333
3111131323221223131331131112311122313111131123223122113
TMB2068 16b 10.139
1311122331211211111313333323311131221132131313121111111321312333133213322231332333
311113132322122231-1331131112311122313311131123223122113
NAU1119 26c 0.000
13333231122232122222322222221321332311332222233122212332222-33222222223223223332
1233222223333223221222232222232222232221212222222322332
JESPR92 26c 17.303
133323133-3233-33233333331231321322211331222323112212232222222223322232222223332
12232222233323232212322322222322322232-1212223232122333
BNL3816 26c 17.704
13332313323231133231333331232321322211331222323112212232222222223322232222223332
12232222233332323221232232222232232223221212223232122333
NAU3006 26c 18.270
133323133232311332313333312313213222113312223231122122322222222233222322222223332
12232222233332323221232232222232232223221212223232122333
BNL840 26c 18.270
133323133232311332313333312313213222113312223231122122322222222233222322222223332
12232222233332323221232232222232232223221212223232122333
CIR391 26c 18.270
133323133232311332313333312313213222113312223231122122322222222233222322222223332
12232222233332323221232232222232232223221212223232122333
CIR039 26c 18.481
133323133232311332313333312313213222113312222231122122322222222233222322222223332
12232222233332323221232232222232232223221212223232122333
BNL3510 26c 19.071
1333231332321113323133-3312313213222113312223231122122322222222233222322222223332
12222222233332323221232232222232232223221212223232122333
NAU2195 26c 23.350
133323133232211332313323312313213222113312223231122122322111122233122322222223332
12222222233332323221232232222232232223221212223232122333
BNL3948 20b 0.000
121112313123121113322332113331131123213132332111311312133-3-13323332123223313321
132221122222331133132131-133211321132321222232232312222
GH54 20b 10.468
1212223331223----332233-11323233-122211232332--123--3213323-133-33-21-32-33133--
1--2--3-222-3311331321312332211331122323222232232312222
GH59 20b 24.966
121222132122322313322312113232333122211332332111313332132233133333321333333322222
2323311222223311311321312223221332133333222233322222222
TMB1629 20b 28.087
12122213312332231332233211323233312221133233211111333213223-133333321333333322222
2322311221333311331321312223223132132333222233322222222
BNL119 20b 34.435
131222113123122312323312113232333113211332332111113332132233133333321333333322222
2323322223333331331221312223221132132323212233322222222
CM82 20b 35.634
1312231---21--23123--3-211-232313112213332232111113332132-3-1333333213333332--22
232-3-2--333-3313-122131-2-32211--132-2--122-332222222-
JESPR235 20b 36.706
1312221-2121222312323332113232313113213332332111113332132233133333321333333322222
232332222333332133122131-223221132132323212233322222222

BNL169 20b 37.827
131222112121222312323332113232333113211332332111113333213223313333332133333322222
23233222233313-1111221312223221132132323212233322222222
GH48 20b 39.350
131222112121222312323332113232333113211322132111113333213223313331332133333322222
23233222213313-1111221312223221132132323212233322222222

[Trait]
Flowering_time M 68.0 79.0 80.0 55.0 56.0 56.0 84.0 67.0 70.0 73.0 58.0 53.0
55.0 61.0 79.0 63.0 71.0 65.0 58.0 0.0 79.0 65.0 77.0 68.0 70.0 71.0 59.0 64.0
85.0 49.0 67.0 88.0 70.0 84.0 73.0 69.0 65.0 56.0 67.0 63.0 65.0 56.0 60.0 71.0
59.0 61.0 55.0 53.0 55.0 59.0 55.0 70.0 59.0 58.0 58.0 55.0 48.0 59.0 65.0 50.0
50.0 48.0 47.0 51.0 59.0 53.0 47.0 63.0 61.0 52.0 55.0 58.0 43.0 48.0 56.0 65.0
75.0 58.0 0.0 80.0 81.0 59.0 73.0 52.0 79.0 72.0 70.0 64.0 56.0 65.0 60.0 54.0
49.0 53.0 65.0 56.0 63.0 63.0 60.0 57.0 53.0 68.0 51.0 81.0 49.0 65.0 52.0 44.0
69.0 65.0 59.0 55.0 81.0 65.0 67.0 58.0 55.0 60.0 63.0 64.0 65.0 67.0 57.0 80.0
65.0 57.0 60.0 74.0 51.0 0.0 0.0 58.0 61.0 68.0 63.0
Buds_number M 19.0 1.0 2.0 69.0 114.0 57.0 4.0 33.0 24.0 36.0 41.0 62.0 68.0
24.0 2.0 94.0 33.0 56.0 46.0 0.0 2.0 26.0 12.0 25.0 16.0 11.0 22.0 22.0 5.0 31.0
6.0 1.0 16.0 2.0 10.0 10.0 9.0 25.0 40.0 76.0 47.0 45.0 17.0 11.0 98.0 54.0 32.0
37.0 81.0 51.0 86.0 22.0 55.0 21.0 63.0 33.0 88.0 62.0 25.0 55.0 35.0 50.0 75.0
30.0 45.0 43.0 84.0 63.0 30.0 93.0 17.0 75.0 84.0 30.0 38.0 20.0 12.0 39.0 0.0
6.0 6.0 28.0 11.0 37.0 7.0 20.0 18.0 44.0 31.0 19.0 45.0 49.0 41.0 45.0 28.0
58.0 16.0 34.0 35.0 30.0 80.0 17.0 58.0 8.0 85.0 37.0 28.0 29.0 11.0 19.0 28.0
103.0 3.0 46.0 12.0 81.0 31.0 37.0 19.0 14.0 28.0 18.0 37.0 4.0 6.0 11.0 15.0
4.0 20.0 0.0 0.0 20.0 32.0 21.0 30.0
Flowering_frame M 26.0 0.0 4.0 39.0 38.0 37.0 8.0 27.0 24.0 21.0 36.0 40.0 38.0
30.0 14.0 30.0 22.0 28.0 36.0 0.0 1.0 29.0 17.0 25.0 24.0 23.0 34.0 30.0 6.0
45.0 24.0 0.0 24.0 3.0 18.0 18.0 26.0 38.0 27.0 31.0 29.0 38.0 32.0 22.0 35.0
32.0 28.0 36.0 39.0 35.0 39.0 24.0 35.0 36.0 36.0 38.0 45.0 35.0 28.0 44.0 42.0
44.0 42.0 36.0 25.0 40.0 47.0 31.0 33.0 42.0 34.0 36.0 48.0 43.0 36.0 29.0 18.0
36.0 0.0 14.0 12.0 34.0 18.0 41.0 15.0 22.0 24.0 29.0 38.0 28.0 34.0 40.0 40.0
34.0 26.0 38.0 28.0 31.0 34.0 34.0 41.0 25.0 43.0 13.0 40.0 29.0 32.0 28.0 21.0
29.0 35.0 39.0 12.0 29.0 27.0 36.0 39.0 32.0 26.0 30.0 25.0 27.0 33.0 8.0 23.0
24.0 25.0 20.0 34.0 0.0 0.0 33.0 33.0 26.0 31.0
Photop_flowering M 3.0 4.0 4.0 2.0 2.0 2.0 4.0 3.0 3.0 3.0 2.0 1.0 2.0 2.0 4.0
2.0 3.0 3.0 2.0 6.0 4.0 3.0 4.0 3.0 3.0 3.0 2.0 2.0 5.0 1.0 3.0 5.0 3.0 4.0 3.0
3.0 3.0 2.0 3.0 2.0 3.0 2.0 2.0 3.0 2.0 2.0 2.0 1.0 2.0 2.0 2.0 3.0 2.0 2.0 2.0
2.0 1.0 2.0 3.0 1.0 1.0 1.0 1.0 1.0 2.0 1.0 1.0 2.0 2.0 1.0 2.0 2.0 1.0 1.0 2.0
3.0 4.0 2.0 6.0 4.0 4.0 2.0 3.0 1.0 4.0 3.0 3.0 2.0 2.0 3.0 2.0 2.0 1.0 1.0 3.0
2.0 2.0 2.0 2.0 2.0 1.0 3.0 1.0 4.0 1.0 3.0 1.0 1.0 3.0 3.0 2.0 2.0 4.0 3.0 3.0
2.0 2.0 2.0 2.0 2.0 3.0 3.0 2.0 4.0 3.0 2.0 2.0 3.0 1.0 6.0 6.0 2.0 2.0 3.0 2.0
