# Supplementary material for: QTL mapping for flowering-time and photoperiod insensitivity of cotton Gossypium darwinii Watt
Source: PLoS One. 2017 Oct 9;12(10):e0186240. doi: 10.1371/journal.pone.0186240 (PMC5633191; doi:10.1371/journal.pone.0186240)
Supplement: S4 Data — (PDF) [file pone.0186240.s004.pdf]

#FileID 20170429134821
#bychromosome
-type position
-function 1
-Units cM
-chromosomes 24
-maximum 14
-named yes
-case yes
-start
-Chromosome 01
 TMB1421 0.0
 JESPR289 13.0
 TMB0062 27.2
 BNL3888 43.1
 CM92 46.0
 BNL3580 48.3
-Chromosome 03
 TMB1989 0.0
 BNL1379 16.9
 BNL3441 22.5
 CM106 22.8
 TMB0564 24.8
-Chromosome 04
 TMB0809 0.0
 GH117 21.5
 BNL2572 46.9
-Chromosome 05
 BNL542 0.0
 JESPR241 11.0
 NAU2140 26.0
 GH211 32.8
 TMB0191 37.0
 NAU2001 37.8
 NAU2296 38.5
 NAU3569 39.2
 BNL3995 39.6
 NAU5149 41.5
 JESPR65 55.9
-Chromosome 06
 BNL2884 0.000
 TMB1277 16.7
 BNL4108 17.1
 GH82 28.7
-Chromosome 06b
 TMB0154 0.0
 GH39 4.0
 TMB1538 6.7
 GH32 9.1
 JESPR119 29.0
-Chromosome 10
 PhyB 0.0
 BNL2705 9.3
 BNL2872 9.8
 TMB0307 9.8
 TMB0325 9.8
 TMB0380 18.5

CM67 20.3
BNL1665 29.5
TMB1745 40.4
-Chromosome 11a
JESPR296 0.0
PhyA 3.2
NAU1014 15.5
TMB0064 16.9
BNL625 26.7
TMB0359 30.5
-Chromosome 11b
GH246 0.0
GH74 3.9
BNL3411 4.4
-Chromosome 12
JESPR270 0.0
NAU1237 4.5
BNL3835 5.5
NAU1278 23.6
NAU943 42.3
BNL1679 52.7
CIR148 55.8
NAU5047 68.7
CM85 80.5
-Chromosome 14
JESPR165 0.0
BNL3502 9.7
TMB0803 15.6
NAU2336 28.8
-Chromosome 15
BNL2920 0.0
JESPR152 2.8
TMB1660 16.5
TMB1664 17.4
TMB0201 21.1
TMB0301 26.8
JESPR180 29.3
JESPR298 29.8
BNL4082 35.4
TMB0375 43.1
BNL3902 45.4
BNL1350 50.2
BNL786 69.1
TMB1181 71.0
-Chromosome 16a
BNL3065 0.0
TMB2036 8.6
TMB2068 10.1
-Chromosome 16b
JESPR32 0.0
JESPR128 5.3
JESPR237 5.4
TMB1271 5.4
GH2 5.6
JESPR297 6.0
TMB1409 11.3
BNL3008 13.3

BNL2734 14.1
-Chromosome 19a
TMB1645 0.0
CM209 4.4
CM42 25.4
CM3 25.4
JESPR218 26.6
JESPR236 28.6
-Chromosome 19b
GH71 0.0
TMB1599 2.3
BNL285 28.0
NAU3935 40.7
GH109 56.9
TMB0189 59.8
BNL852 61.5
TMB0366 73.6
BNL4096 76.3
BNL3875 83.0
BNL3977 98.6
TMB1489 100.6
-Chromosome 20
GH48 0.0
BNL169 1.5
JESPR235 2.6
CM82 3.7
BNL119 4.9
TMB1629 11.3
GH59 14.4
GH54 28.9
BNL3948 39.34
-Chromosome 21
JESPR158 0.0
CM23 16.3
TMB0400 18.8
JESPR118 18.8
TMB2038 39.0
BNL3649 47.5
BNL1551 52.8
-Chromosome 23
TMB1425 0.0
TMB0382 15.8
JESPR110 36.8
-Chromosome 24
BNL1521 0.0
BNL2616 5.1
TMB0429 12.4
BNL2568 15.5
GH171 16.1
BNL2655 16.9
GH272 24.3
BNL252 36.2
-Chromosome 25
GH224 0.0
JESPR215 1.4
JESPR227 2.5
CM27 3.7

```
 CM13 5.5
-Chromosome 26a
 NAU1119 0.0
 JESPR92 17.3
 BNL3816 17.7
 NAU3006 18.2
 BNL840 18.2
 CIR391 18.2
 CIR039 18.4
 BNL3510 19.0
 NAU2195 23.3
-Chromosome 26b
 NAU2913 0.000
 BNL341 13.523
 NAU2750 25.313
-Chromosome 26c
 GH200 0.0
 TMB0120 11.9
 GH52 12.4
 BNL3994 14.0
-stop



#bycross
-SampleSize 135
-Cross SF2
-traits 4
-missingtrait ?
-case yes
-TranslationTable
AA  2 2
Aa  1 1
aa  0 0
A- 12 4
a- 10 3
-- -1 5
-start markers
TMB1421 1 1 0 0 0 2 2 0 0 0 1 0 0 0 0 0 1 2 0 2 0 1 1 1 2 2 2
1 1 0 0 1 0 2 0 2 0 0 2 0 1 0 0 1 1 0 2 0 2 2 0 1 0 2 2 2 2 0
2 5 2 2 1 2 1 0 2 1 1 1 2 0 1 2 2 2 1 1 1 1 0 1 1 1 0 2 2 1 1
1 1 1 2 0 0 0 0 0 0 1 1 2 2 1 2 1 2 0 0 0 1 1 2 0 0 1 0 1 1 2
2 1 1 1 1 1 0 1 0 2 0 0 0 2 0
JESPR289 0 1 0 0 0 1 2 0 0 0 1 0 5 0 0 0 0 0 0 0 0 0 5 0 0 5 2
0 0 0 0 1 0 2 0 2 0 0 2 0 0 1 1 1 0 0 2 0 2 2 0 0 0 2 2 2 2 0
2 2 2 2 1 2 0 0 2 0 0 1 2 0 1 2 2 2 2 1 2 2 2 1 1 0 0 2 1 1 1
2 2 2 2 0 1 0 2 0 0 1 1 2 2 1 2 1 2 0 0 2 1 1 2 2 0 1 0 5 2 1
2 1 1 1 1 0 0 2 0 2 2 1 0 2 0
TMB0062 0 0 0 0 0 1 2 0 0 0 1 0 0 1 0 0 0 0 0 0 0 0 0 0 0 0 2
0 0 0 0 2 0 2 0 2 0 0 2 0 0 1 1 1 0 0 2 2 0 2 0 0 0 2 2 2 2 0
2 2 2 2 1 2 0 0 2 0 0 0 2 1 0 2 2 0 0 0 0 0 0 1 1 0 0 1 1 1 5
1 1 1 2 0 1 0 2 0 0 1 0 2 2 2 2 1 2 0 0 0 1 1 2 0 0 1 0 2 1 1
2 0 1 1 2 1 0 0 1 0 0 1 0 2 0
BNL3888 0 1 0 0 0 1 2 0 0 0 0 1 0 2 0 0 0 2 1 1 2 0 1 2 1 0 2
1 0 0 0 2 0 1 0 2 0 0 2 0 2 1 1 1 1 0 2 2 0 2 0 0 0 0 2 2 0 0
1 2 2 1 1 1 1 2 2 1 2 0 1 1 0 1 1 0 0 0 0 0 0 1 1 1 0 2 1 1 1
1 1 1 1 0 1 1 2 0 1 1 1 2 2 2 2 0 2 0 0 0 1 1 2 0 0 1 1 2 1 1
```

2 0 1 1 2 1 0 0 1 0 0 1 0 2 0
CM92 0 1 0 0 0 1 2 0 0 0 0 1 0 2 0 0 0 2 1 1 2 0 5 2 1 0 2 2 0
0 0 2 0 1 0 2 0 0 2 0 2 1 1 1 1 0 2 2 0 2 0 0 0 1 2 2 0 0 1 2
2 1 1 1 1 2 1 1 2 0 1 1 0 1 1 0 0 0 0 0 0 1 1 1 1 2 1 0 1 1 1
1 1 0 2 2 2 0 0 1 1 2 2 2 5 0 2 0 0 0 1 1 2 0 0 1 1 2 1 1 2 0
1 1 2 1 0 0 1 0 0 1 0 2 0
BNL3580 0 1 0 0 0 1 2 0 0 0 0 2 0 2 0 0 0 2 1 1 2 1 1 2 1 0 2
2 0 0 0 2 0 1 0 1 0 0 2 0 2 1 1 1 1 0 2 2 0 2 0 0 0 1 2 1 0 0
1 5 2 1 1 1 1 1 1 1 2 0 1 1 1 1 1 0 0 0 0 0 0 1 1 1 1 2 1 0 1
1 1 1 1 0 2 2 2 0 0 1 1 2 2 2 2 0 2 0 0 0 1 1 2 0 0 1 1 2 1 1
2 0 1 1 2 1 0 0 1 0 0 1 0 2 0
TMB1989 0 0 0 0 0 1 2 0 0 0 0 1 0 0 0 0 2 1 2 1 0 1 5 1 0 1 2
2 0 0 0 0 0 1 1 1 1 1 0 0 1 2 0 1 1 0 2 1 5 0 0 0 0 0 2 1 0 1
1 1 5 2 1 2 2 0 2 0 1 0 0 0 0 0 2 2 2 1 2 2 2 0 1 0 0 2 2 2 2
0 0 0 2 0 0 0 0 5 0 2 1 0 0 1 0 0 2 1 2 2 0 1 2 2 2 1 1 1 1 2
2 1 2 2 0 0 1 2 0 2 2 2 0 1 1
BNL1379 0 0 0 0 0 0 2 0 0 0 0 1 0 2 0 0 2 0 2 1 0 1 2 0 0 0 2
2 0 0 0 1 0 0 2 0 1 1 1 0 0 0 0 0 1 0 2 1 2 2 0 0 0 0 2 1 0 0
0 1 0 1 0 2 2 0 0 1 1 1 0 1 0 0 1 2 2 1 2 2 2 0 0 0 0 2 2 2 2
0 0 0 2 0 0 0 0 0 0 2 1 0 2 1 0 0 0 1 0 2 0 0 0 2 2 0 1 0 1 0
0 0 0 0 2 0 1 2 0 2 2 2 0 0 0
BNL3441 0 0 0 0 0 2 2 0 0 5 2 2 0 5 5 0 2 1 2 1 5 1 5 0 0 0 5
2 0 0 0 1 0 0 2 0 1 1 1 0 0 2 1 0 1 0 2 1 2 2 0 0 0 0 2 1 0 5
0 5 0 2 0 2 2 0 0 1 1 1 0 1 0 0 1 2 2 5 2 2 2 0 0 0 0 2 2 2 2
0 0 0 2 0 0 0 1 1 0 2 1 0 2 1 5 0 0 1 2 2 0 0 0 2 2 0 1 0 1 0
0 0 0 0 2 5 1 2 0 2 2 2 0 0 1
CM106 0 0 0 0 0 2 2 0 0 0 2 2 0 2 0 0 2 1 2 1 0 1 2 0 0 0 2 2
0 0 0 1 0 1 2 0 1 1 1 0 0 2 1 0 1 0 2 1 2 2 0 0 0 0 2 1 0 0 0
1 0 2 0 2 2 0 0 1 1 1 0 1 0 0 1 2 2 1 2 2 2 0 0 0 0 2 2 2 2 0
0 0 2 0 0 0 1 1 0 2 1 0 2 1 0 0 0 1 2 2 0 0 0 2 2 0 1 0 1 0 0
0 0 0 2 0 1 2 0 2 2 2 0 0 1
TMB0564 1 0 0 0 0 2 2 0 0 0 2 2 0 2 0 0 2 1 2 1 0 1 2 0 0 0 2
2 2 0 0 1 0 1 2 0 1 1 1 0 0 2 1 0 1 0 2 1 2 2 0 1 0 0 2 1 0 0
0 1 0 2 0 2 2 0 0 1 1 1 0 1 0 0 1 2 2 1 2 2 2 0 0 0 0 2 2 2 2
0 0 0 2 0 1 0 1 1 0 2 1 0 2 1 0 0 0 1 2 2 0 0 0 2 2 0 1 0 1 0
0 0 0 0 2 0 1 2 0 2 2 2 0 0 1
TMB0809 2 0 0 0 0 1 1 1 0 0 1 0 0 2 0 0 2 0 2 2 0 1 1 0 1 1 1
2 2 0 0 2 0 0 0 2 2 2 0 0 2 1 1 0 0 0 0 2 2 2 0 2 0 1 2 0 2 0
0 1 1 1 0 1 2 2 0 1 2 1 1 1 1 1 1 1 2 1 0 1 1 1 0 0 0 0 0 0 0
2 2 2 0 1 0 2 1 0 1 0 1 1 1 1 0 0 0 1 0 2 0 0 0 1 0 0 1 0 0 1
0 0 0 0 1 1 1 0 1 1 0 1 0 0 0
GH117 5 0 1 2 1 0 0 2 1 1 0 5 1 1 1 0 1 0 2 2 0 1 2 1 1 1 1 2
2 0 2 2 0 1 0 2 0 0 0 1 2 2 1 0 0 1 0 2 2 2 1 2 2 1 0 0 5 0 0
1 1 1 0 1 2 5 0 1 2 1 1 5 1 1 1 2 2 1 2 2 2 0 0 0 0 0 0 5 0 2
2 2 0 1 0 2 1 0 2 0 1 2 1 1 5 0 0 1 0 2 0 0 5 2 2 0 1 0 5 2 0
0 0 0 1 1 1 2 1 2 2 0 0 0 0
BNL2572 2 0 0 1 1 0 0 2 1 2 0 0 1 0 0 0 1 0 2 1 0 1 2 1 1 1 0
2 1 0 2 1 0 1 0 2 2 2 0 1 0 1 0 0 0 1 0 2 1 2 1 1 1 1 0 1 2 1
2 1 1 1 1 0 2 2 2 0 2 1 1 1 1 1 1 2 2 1 2 2 2 0 0 1 2 0 0 0 0
0 0 0 0 0 0 1 1 1 1 0 1 2 1 1 1 1 1 1 0 2 2 1 2 2 2 2 0 0 0 2
1 0 1 1 2 1 1 2 1 2 2 0 0 0 0
BNL542 0 1 1 2 0 0 2 0 1 1 2 0 1 5 5 0 2 2 1 1 1 0 1 5 2 1 1 1
0 5 5 1 2 2 0 2 0 0 0 0 1 0 2 1 1 0 2 0 2 1 2 0 1 2 1 2 2 2 2
2 2 2 2 2 0 1 2 0 2 5 2 0 0 2 2 1 1 0 0 2 0 2 5 2 1 5 2 2 2 2
1 0 2 1 1 5 1 1 5 5 0 0 1 1 2 2 2 1 0 1 2 2 0 1 0 2 1 2 0 0 2
1 2 0 0 0 0 0 0 2 2 0 0 1 0
JESPR241 0 1 1 2 0 0 2 0 1 1 2 0 1 2 1 0 2 2 1 1 2 0 1 5 2 1 1

1 0 1 2 1 2 2 0 2 0 0 0 0 1 0 2 1 1 1 2 0 2 1 1 0 0 2 1 2 2 2
2 5 2 2 2 2 0 1 2 0 2 1 1 1 5 2 2 2 1 2 5 0 2 2 5 2 1 5 2 2 2
2 1 2 2 2 1 5 1 1 1 5 2 0 1 1 2 2 2 2 0 1 2 2 0 1 0 2 2 2 0 0
2 0 2 0 0 2 1 2 1 0 0 0 0 1 0
NAU2140 0 1 1 2 0 0 2 0 0 1 2 0 1 2 1 0 2 2 2 2 2 2 2 2 2 2 2
2 0 1 1 0 2 2 0 2 0 0 0 0 2 1 2 1 2 1 2 2 2 1 0 0 0 2 2 2 2 2
2 5 2 2 2 2 2 2 2 2 2 0 2 1 0 2 2 2 1 1 2 0 2 2 2 2 2 2 2 2 2
2 1 1 2 1 1 1 2 1 1 2 1 0 1 1 2 2 2 1 2 1 2 2 0 1 0 2 1 2 0 0
2 0 2 2 0 0 0 2 1 0 0 0 1 2 1
GH211 0 1 1 2 0 0 0 0 1 2 2 0 1 2 1 0 2 2 2 2 2 2 2 2 2 2 2
0 1 1 0 2 2 2 2 0 0 0 0 2 1 1 2 2 1 0 2 1 0 0 0 0 2 0 2 2 2 2
2 2 2 2 2 2 2 2 2 2 0 2 0 0 2 2 2 1 1 2 0 1 2 2 2 2 2 2 2 2 2
1 1 2 1 1 0 1 1 1 2 1 0 1 1 2 2 2 1 1 1 2 2 0 1 0 2 1 2 0 1 2
0 2 2 0 0 0 2 1 0 0 0 1 2 1
TMB0191 0 1 0 2 0 0 0 0 0 2 2 0 1 2 1 0 2 2 2 1 2 2 2 2 2 2 2
2 0 1 1 0 2 2 2 2 0 0 0 0 2 2 1 2 2 1 0 2 1 0 2 0 0 2 0 2 1 2
2 5 2 2 2 2 2 2 2 2 2 0 2 0 1 2 2 2 0 1 2 1 1 2 2 2 2 2 2 2 2
0 0 0 2 1 1 2 1 1 2 2 1 0 1 1 2 2 2 1 1 1 2 2 0 1 0 2 1 2 2 1
2 0 2 2 0 0 0 2 1 0 0 0 1 2 1
NAU2001 0 1 1 2 0 0 0 0 1 2 2 0 1 2 1 0 2 2 2 2 2 2 2 2 2 2 2
2 0 1 1 0 2 2 2 2 0 0 0 0 2 2 1 2 2 1 0 2 1 0 0 0 0 2 0 2 2 2
2 2 2 2 2 2 2 2 2 2 2 0 2 0 1 2 2 2 1 1 2 1 1 2 2 2 2 2 2 2 2
0 0 0 2 1 1 2 1 1 2 2 1 0 1 1 2 2 2 1 1 1 2 2 0 1 0 2 1 2 2 1
2 0 2 2 0 0 0 2 1 0 0 0 1 2 1
NAU2296 0 1 0 2 0 0 0 0 0 2 2 0 1 2 1 0 2 2 2 2 2 2 2 2 2 2 2
2 0 1 1 0 2 2 2 2 0 0 0 0 2 2 1 2 2 1 0 2 1 0 0 0 0 2 0 2 2 2
2 5 2 2 2 2 2 2 2 2 2 0 2 0 1 2 2 2 0 1 2 1 1 2 2 2 2 2 2 2 2
0 0 0 2 1 1 2 1 1 2 2 1 0 1 1 2 2 2 1 1 1 2 2 0 1 0 2 1 2 2 1
2 0 2 2 0 0 0 2 1 0 0 0 1 2 1
NAU3569 0 1 0 2 0 0 0 0 1 2 2 0 1 2 1 1 2 2 2 2 2 2 2 2 2 2 2
2 0 1 1 0 2 2 2 2 0 0 0 0 2 2 1 2 2 1 0 2 1 0 0 0 0 2 0 2 2 2
2 2 2 2 2 2 2 2 2 2 2 0 2 0 1 2 2 2 0 1 2 1 1 2 2 2 2 2 2 2 2
0 0 0 2 1 1 2 1 1 2 2 1 0 1 1 2 2 2 1 1 1 2 2 0 1 0 2 1 2 2 1
2 0 2 2 0 0 0 2 1 0 0 0 1 2 2
BNL3995 0 1 0 2 0 0 0 0 0 2 2 0 1 2 1 1 2 2 2 2 2 2 2 2 2 2 2
2 0 1 1 0 2 2 2 2 0 0 0 0 2 2 1 2 2 1 0 2 1 0 0 0 0 2 0 2 2 2
2 5 2 2 2 2 2 2 2 2 2 0 2 0 1 2 2 2 0 1 2 1 1 2 2 2 2 2 2 2 2
0 0 5 2 1 1 2 1 1 2 2 1 0 1 1 2 2 2 1 1 1 2 2 0 1 0 2 1 2 2 1
2 0 2 2 0 0 0 2 0 0 0 0 1 2 2
NAU5149 0 1 0 2 0 0 0 0 0 2 2 0 1 2 1 1 2 2 2 2 2 2 2 2 2 2 2
2 0 1 1 0 2 2 2 2 0 0 0 0 2 2 1 1 2 1 0 2 1 0 0 0 0 2 0 2 2 2
2 2 2 2 2 2 2 2 2 1 1 0 2 0 1 2 2 2 0 1 2 0 0 2 2 2 1 2 2 2 2
0 0 0 2 1 1 2 1 1 2 2 1 0 1 1 2 2 2 1 1 1 2 2 0 1 0 2 1 2 2 1
2 0 2 2 0 0 0 2 1 0 0 0 1 2 2
JESPR65 0 2 0 2 1 0 1 0 1 0 2 0 1 2 1 1 2 2 2 2 2 2 2 2 2 2 0
2 0 2 1 0 1 1 2 1 2 1 0 0 2 2 2 2 2 1 0 2 1 0 0 0 0 1 0 1 0 5
0 5 0 1 2 2 2 2 2 2 2 1 2 1 1 1 1 2 0 0 2 1 1 2 2 2 2 2 2 2 2
0 0 0 2 2 2 2 2 2 2 2 1 0 2 1 5 2 2 2 0 1 2 2 0 1 0 2 1 2 2 1
2 0 2 2 0 0 1 2 1 0 0 0 0 2 0
JESPR119 0 2 2 1 0 1 2 2 1 0 2 1 0 1 0 0 0 0 0 0 2 0 0 0 0 0 2
0 0 0 0 0 0 2 2 2 2 2 1 0 0 1 1 1 0 0 2 0 1 1 0 1 0 2 2 2 2 1
2 5 2 2 2 2 0 0 2 0 0 0 0 0 0 5 0 1 1 0 2 1 1 0 0 0 1 0 1 2 2
0 0 0 2 0 0 2 2 0 2 1 0 1 1 2 1 1 0 0 0 2 2 1 0 0 0 0 0 2 1 2
1 0 1 0 2 1 0 1 2 1 2 0 1 0 2
GH32 0 1 0 0 0 1 2 2 1 0 1 1 0 1 0 0 0 0 0 0 2 0 0 0 0 0 0 0 1
0 0 0 0 2 2 2 2 2 1 0 0 0 0 0 0 0 2 0 2 0 0 0 0 2 2 2 2 2 2 5
2 2 2 2 0 0 2 0 0 0 2 0 1 2 2 1 1 1 2 1 1 2 2 0 2 0 0 2 2 2 2

2 2 0 0 0 0 0 1 1 1 1 0 2 2 1 0 0 2 2 2 0 0 0 0 1 0 1 2 2 1 0
2 0 0 1 0 2 0 1 1 2 2 0 2
TMB1538 0 1 0 0 0 1 2 2 1 0 1 1 0 1 0 0 0 0 0 0 2 0 0 0 0 0 2
0 1 0 0 0 0 2 2 2 2 2 1 0 0 0 0 0 0 0 2 0 2 0 0 0 0 2 2 2 2 2
2 5 2 2 2 2 0 0 2 0 0 0 2 0 1 2 2 1 1 1 2 1 1 2 0 0 2 0 0 2 2
2 2 2 2 0 0 0 0 0 1 1 1 1 1 2 2 1 0 0 2 2 2 2 0 0 0 1 0 1 2 2
1 0 2 0 0 1 0 2 0 1 1 2 2 0 2
GH39 0 1 0 0 0 1 2 2 1 0 1 1 0 1 0 0 0 0 0 0 2 0 0 0 0 0 2 0 1
0 0 0 0 2 2 2 2 2 1 0 0 0 0 1 0 0 2 0 2 0 0 0 0 2 2 2 2 2 2 2
2 2 2 2 0 0 2 0 0 0 2 0 1 2 2 1 1 1 2 1 1 2 1 2 2 0 0 2 2 2 2
2 2 0 0 0 0 0 1 1 1 1 1 2 1 1 0 0 2 2 2 2 2 0 2 1 0 1 2 2 1 0
2 0 0 0 0 2 0 1 1 2 2 0 2
TMB0154 0 1 0 0 0 1 2 0 0 0 1 1 0 1 0 0 0 0 0 0 0 0 5 0 0 5 2
0 1 0 0 0 0 2 2 2 2 2 1 0 0 0 0 2 2 0 2 0 2 0 0 0 0 2 2 2 2 2
2 2 2 2 2 2 0 0 2 0 0 0 2 0 1 2 2 1 1 1 2 1 1 2 0 2 1 0 0 2 2
2 2 2 2 0 0 0 0 0 1 1 1 1 1 2 1 1 0 0 2 2 2 2 0 0 0 1 1 5 2 5
1 0 2 0 0 1 0 2 5 1 1 2 2 0 5
TMB1745 0 2 0 1 1 1 1 2 1 0 1 0 0 1 1 0 1 1 2 1 2 0 2 2 1 2 0
2 1 1 0 1 1 0 1 0 2 2 2 0 1 1 0 2 1 1 2 1 2 2 1 1 1 0 1 0 0 1
0 0 0 0 0 0 0 2 2 0 1 1 0 1 1 0 0 2 2 1 2 2 2 1 1 1 1 2 2 2 2
1 0 2 0 0 2 2 1 2 0 2 1 2 2 1 2 2 1 1 0 2 1 0 1 2 0 1 0 2 2 2
1 1 0 1 0 1 1 2 1 2 2 2 0 2 2
BNL1665 0 2 0 1 1 1 1 2 1 0 1 2 1 0 1 0 1 1 2 1 2 0 1 2 1 2 0
2 1 1 0 1 1 0 1 0 2 2 2 0 1 1 0 2 1 1 2 1 0 0 1 1 1 0 1 0 0 1
0 0 0 0 0 0 0 2 2 0 0 1 0 1 1 0 0 2 2 0 2 0 2 1 1 1 1 2 2 2 2
1 0 0 0 0 0 0 1 0 0 2 1 2 2 1 1 1 1 1 0 1 1 0 1 1 0 1 0 1 1 2
1 1 0 1 0 1 1 2 1 1 2 2 0 0 0
CM67 0 2 0 1 0 1 0 5 5 5 2 2 0 0 0 0 1 1 2 1 2 0 1 2 2 2 0 1 1
0 0 1 0 0 0 0 2 2 2 0 1 1 0 2 1 0 2 1 0 0 0 1 0 0 1 0 0 5 0 5
0 0 0 0 0 2 2 1 0 1 0 0 0 5 0 2 1 0 2 0 1 1 1 1 1 2 2 2 2 0 0
0 0 0 0 0 0 0 0 2 1 2 2 1 5 1 1 1 0 1 0 0 1 1 0 1 0 1 1 2 1 1
0 1 1 0 1 2 0 2 2 1 0 0 0
TMB0380 0 2 0 0 0 1 0 2 1 0 2 2 0 0 0 0 0 1 2 1 2 0 5 2 2 2 0
1 1 0 0 1 0 0 0 0 2 2 2 0 1 1 0 2 1 0 2 1 0 0 0 1 0 0 1 0 0 2
0 0 0 0 0 0 0 2 1 1 0 1 0 0 0 0 0 2 1 0 2 0 1 1 1 1 1 2 2 2 2
0 0 0 0 0 5 0 1 0 0 2 1 2 2 5 1 5 1 1 0 1 0 0 1 1 0 1 0 1 1 2
1 1 0 5 1 0 1 2 0 2 2 1 0 0 2
TMB0325 2 1 0 0 0 2 1 2 1 0 2 2 0 2 0 0 1 1 2 1 1 0 1 2 2 2 0
1 1 0 0 1 0 0 0 0 2 2 2 0 0 1 0 2 1 0 2 0 1 0 0 1 0 0 0 0 0 2
0 0 0 0 0 0 1 2 2 2 0 1 0 0 1 0 0 2 1 0 2 0 1 1 0 1 1 2 2 2 2
0 0 0 0 0 0 0 1 0 0 2 1 2 2 1 5 1 1 1 0 1 0 0 0 1 0 1 0 2 5 2
0 1 1 0 1 0 1 2 0 2 2 0 0 0 2
TMB0307 2 1 0 0 0 2 1 2 1 0 2 2 0 2 0 0 1 1 2 1 1 0 1 2 2 2 0
1 1 0 0 1 0 0 0 0 2 2 2 0 0 1 0 2 1 0 2 0 1 0 0 1 0 0 0 0 0 2
0 0 0 0 0 0 1 2 2 2 0 1 0 0 1 0 0 2 1 0 2 0 1 1 0 1 1 2 2 2 2
0 0 0 0 0 0 0 1 0 0 2 1 2 2 1 5 1 1 1 0 1 0 0 0 1 0 1 0 2 5 2
0 1 1 0 1 0 1 2 0 2 2 0 0 0 2
BNL2872 2 1 0 0 0 2 1 2 1 0 2 2 0 2 0 0 1 1 2 1 1 0 1 2 2 2 0
1 1 0 0 1 0 0 0 0 2 2 2 0 0 1 0 2 1 0 2 0 1 0 0 1 0 0 0 0 0 2
0 0 0 0 0 0 1 2 2 2 0 1 0 0 1 0 0 2 1 0 2 0 1 1 0 1 1 2 2 2 2
0 0 0 0 0 0 0 1 0 0 2 1 2 2 1 5 1 1 1 0 1 0 0 0 1 0 1 0 2 5 2
0 1 1 0 1 0 1 2 0 2 2 0 0 0 2
BNL2705 2 1 0 0 0 2 1 2 1 0 2 2 0 2 0 0 1 1 2 1 1 0 1 2 2 2 0
1 1 0 0 1 0 0 0 0 2 2 2 0 0 1 0 2 1 0 2 0 1 0 0 1 0 0 0 0 0 2
0 0 0 0 0 0 1 2 2 2 0 1 0 0 1 0 0 2 5 0 2 0 1 1 0 0 1 2 2 2 2
0 5 0 0 0 0 0 1 0 0 2 1 2 2 1 0 1 1 1 0 1 0 0 0 1 0 1 0 2 1 2
0 1 1 0 1 0 1 2 0 2 2 0 0 0 2

PhyB2 2 1 0 0 0 2 1 2 0 0 2 2 0 1 0 0 1 1 2 1 1 0 1 5 2 2 1 1
1 0 0 1 0 0 2 0 2 2 2 0 0 0 0 2 1 0 1 0 2 2 0 1 0 0 0 0 0 5 0
0 0 0 0 0 1 2 2 2 0 1 0 0 1 0 0 2 2 1 5 2 2 1 0 0 0 5 1 1 0 0
0 0 0 0 0 1 0 0 0 5 1 2 2 1 0 1 1 1 0 2 0 0 0 2 0 1 0 2 1 2 0
1 1 0 1 0 1 2 0 2 2 0 0 0 2
PhyB 2 1 0 0 0 2 1 2 0 0 2 2 0 1 0 0 1 1 2 1 1 0 1 5 2 2 1 1 1
0 0 1 0 0 2 0 2 2 2 0 0 0 0 2 1 0 1 0 2 2 0 1 0 0 0 0 0 5 0 0
0 0 0 0 1 2 2 2 0 1 0 0 1 0 0 2 2 1 5 2 2 1 0 0 0 5 1 1 0 0 0
0 0 0 0 1 0 0 0 5 1 2 2 1 0 1 1 1 0 2 0 0 0 2 0 1 0 2 1 2 0 1
1 0 1 0 1 2 0 2 2 0 0 0 2
JESPR296 0 1 0 0 2 1 0 1 1 2 1 2 1 2 1 1 2 2 1 2 1 1 2 1 1 2 2
2 1 1 1 1 1 1 0 1 1 1 0 1 1 2 2 0 2 1 0 2 2 1 1 2 1 0 0 0 2 0
2 1 1 2 1 2 1 0 1 2 1 1 1 1 1 2 2 0 0 0 0 0 0 1 0 1 1 2 2 2 2
0 2 2 2 1 2 2 2 2 2 2 1 2 2 2 1 0 2 1 1 0 1 1 0 0 0 1 0 1 2 2
1 1 1 0 0 1 1 0 1 0 0 0 1 0 0
PhyA 0 1 5 5 2 2 0 1 1 2 5 2 1 5 5 5 5 5 1 2 1 1 2 1 1 1 2 2 5
1 5 5 1 1 0 1 1 1 0 1 1 2 2 0 2 1 0 2 2 1 1 2 1 0 0 5 2 5 2 1
5 2 1 2 1 5 1 5 1 1 1 1 1 2 2 0 0 0 0 0 0 1 0 1 0 2 5 2 2 5 2
5 2 5 2 5 2 2 2 2 1 1 1 1 1 0 2 1 1 0 1 1 0 5 0 1 0 0 2 2 1 1
1 0 0 1 1 0 1 0 0 0 1 0 1
NAU1014 2 1 1 0 1 2 0 2 1 2 2 1 0 2 2 2 2 2 1 2 0 1 2 1 1 1 2
2 2 1 1 0 1 0 0 1 1 2 2 1 1 2 2 1 1 0 2 2 2 1 1 2 1 1 0 0 2 1
2 5 0 2 1 1 1 0 1 1 1 1 1 1 1 1 2 0 0 0 0 0 0 1 0 0 1 2 2 2 2
0 2 2 5 1 2 2 2 2 2 2 1 2 1 1 2 0 1 1 0 0 1 1 0 0 0 1 0 0 2 2
1 1 0 0 0 1 1 0 1 0 0 0 0 0 0
TMB0064 2 1 1 0 1 2 0 2 1 2 2 1 0 2 2 2 2 2 1 2 0 1 2 1 1 1 2
2 2 1 1 0 1 0 0 1 2 2 2 1 1 2 2 1 1 0 2 2 2 1 1 2 1 1 0 0 2 1
2 1 0 2 1 1 1 0 1 1 1 1 1 1 0 1 2 0 0 0 0 0 0 1 0 1 1 2 2 2 2
0 2 2 2 1 2 2 2 2 2 2 1 2 1 1 2 0 1 1 0 0 1 1 0 0 0 1 0 0 2 2
1 1 0 0 0 1 1 0 0 0 0 0 0 0 0
BNL625 2 0 1 0 0 1 0 2 1 2 0 1 0 0 2 2 2 2 2 2 0 2 2 2 2 2 2 2
2 1 1 0 1 0 0 1 2 1 2 1 2 2 2 1 2 0 2 0 1 1 1 2 1 1 0 0 2 2 2
0 0 2 1 1 2 2 1 5 2 1 1 0 0 1 2 0 0 0 0 0 0 1 0 0 1 2 2 2 2 0
2 2 2 1 2 2 2 2 2 2 1 2 1 1 0 2 1 1 0 0 1 1 0 0 0 1 0 0 2 2 1
1 0 0 0 1 1 0 0 0 0 5 0 0 0
TMB0359 2 1 1 0 0 1 2 2 1 2 0 1 0 0 2 2 2 2 2 2 0 2 2 2 2 2 2
2 2 1 1 0 1 0 0 1 2 1 1 1 1 2 2 2 1 2 0 2 0 1 2 1 2 1 1 2 0 2 1
2 0 0 2 1 1 2 2 1 2 2 1 1 0 0 1 2 0 0 0 0 0 0 1 0 1 1 2 2 2 2
0 2 2 2 1 2 2 2 2 2 2 1 2 0 1 2 0 1 1 0 0 1 1 0 0 0 1 0 0 2 2
1 1 0 0 0 1 1 0 0 0 0 0 5 0 0
GH246 1 0 2 2 2 2 2 1 2 2 1 0 2 1 2 2 1 2 1 1 0 0 1 0 1 2 0 0
0 2 2 0 2 0 0 0 2 1 2 2 0 2 0 2 1 2 2 0 2 2 2 2 2 0 2 0 0 1 0
0 0 0 1 0 2 1 2 0 0 1 0 1 1 0 0 1 0 0 0 1 1 0 2 1 1 2 2 2 2 1
2 2 0 0 1 2 1 2 0 2 5 0 0 1 1 5 1 0 0 1 1 1 0 1 0 0 0 0 1 2 1
1 1 2 0 1 0 0 1 0 1 1 0 1 0
GH74 1 0 2 2 2 0 2 1 1 2 0 0 2 0 2 2 1 2 1 1 0 0 0 0 1 2 0 0 0
2 2 0 2 0 0 0 2 1 2 2 0 2 0 1 1 2 2 0 2 2 2 2 2 0 2 0 0 1 0 0
0 0 1 0 2 1 2 0 0 1 0 0 0 0 0 1 0 0 0 1 1 0 2 1 1 2 2 2 2 1 2
2 0 1 1 2 1 2 0 2 1 0 0 1 1 2 1 0 0 1 1 1 0 1 0 0 0 0 1 2 1 1
1 2 0 1 0 0 0 0 1 1 0 1 0
BNL3411 1 0 2 2 2 0 2 1 1 2 0 0 2 0 2 2 1 2 1 1 0 0 1 0 1 2 0
0 0 2 2 0 2 0 0 0 2 1 2 2 0 2 0 1 1 2 2 0 2 2 2 2 2 0 2 0 0 1
0 0 0 0 1 0 2 1 2 0 0 1 0 0 0 0 0 1 0 0 0 1 1 0 2 2 1 2 2 2 2
1 2 2 0 1 1 2 1 2 0 2 1 0 0 1 1 2 1 0 0 1 1 1 0 1 0 0 0 0 1 2
1 1 1 2 0 1 0 0 0 0 1 1 0 1 0
JESPR270 1 2 0 0 0 2 2 2 1 0 2 2 0 2 0 0 0 1 1 1 1 2 1 1 2 1 0
2 0 0 0 1 0 1 1 1 2 2 2 0 1 0 1 2 0 0 1 2 2 2 0 1 0 2 1 1 1 0

1 1 0 1 0 0 0 2 0 1 2 1 2 1 1 1 1 1 1 0 1 1 1 0 0 0 0 0 0 0 0
2 2 2 0 1 1 2 0 1 2 0 1 1 0 1 0 0 0 0 1 0 0 0 0 1 0 0 1 0 2 1
0 2 0 0 2 1 0 0 1 1 2 1 1 0 1
NAU1237 1 2 0 0 0 2 0 2 1 0 2 2 0 2 0 0 0 1 1 0 1 2 1 1 2 1 0
1 0 0 0 1 0 1 2 1 2 2 2 0 1 0 1 2 0 0 0 2 2 2 0 1 0 2 0 1 1 0
1 5 0 1 0 0 0 2 0 1 2 1 2 1 1 1 1 1 0 0 0 0 1 0 0 0 0 0 0 0 0
2 2 2 0 1 1 2 0 0 2 0 1 1 0 1 0 0 0 0 1 0 0 0 0 1 0 0 1 0 2 1
0 2 0 0 2 1 0 0 1 1 2 0 0 0 1
BNL3835 1 2 0 0 0 2 0 2 1 0 2 2 0 2 0 0 0 1 1 0 1 2 1 1 2 1 0
1 0 0 0 1 0 1 2 1 2 2 2 0 1 0 1 2 0 0 0 2 2 2 0 1 0 2 0 1 1 0
1 5 0 1 0 0 0 2 0 1 2 1 2 1 1 1 1 1 0 0 0 0 1 0 0 0 0 0 0 0 0
2 2 2 0 1 1 2 0 0 2 0 1 1 0 1 0 0 0 0 1 0 0 0 0 1 0 0 1 0 2 1
0 2 0 0 2 1 0 1 1 1 2 0 1 0 2
NAU1278 0 2 0 0 0 2 0 2 1 0 2 0 0 2 0 0 0 1 2 0 0 0 2 1 2 1 0
1 0 0 2 1 0 0 2 1 2 2 2 0 1 0 1 2 2 0 0 2 2 2 0 0 0 0 0 1 2 0
1 0 0 2 0 0 0 2 0 2 2 1 2 1 1 1 1 1 0 0 2 2 1 1 0 0 0 0 1 1 1
2 1 1 1 0 1 2 0 0 1 2 1 2 0 0 0 0 0 0 2 0 2 2 0 2 0 0 1 0 2 1
0 2 0 2 0 1 0 0 1 0 1 0 1 0 2
NAU943 0 2 0 0 0 0 0 2 1 0 0 1 0 0 5 0 2 2 2 2 5 1 2 2 2 2 0 2
0 0 0 0 0 0 1 1 2 2 0 0 2 1 1 2 2 0 0 0 2 2 0 0 0 2 0 1 2 0 1
1 1 2 0 1 2 2 0 2 2 1 2 1 0 0 1 0 0 0 0 1 1 0 0 0 0 0 0 1 0 0
0 0 1 0 0 2 1 1 1 2 1 2 0 0 0 1 0 2 2 0 0 0 0 2 0 0 1 0 2 2 0
2 0 0 1 1 1 1 1 1 1 2 1 0 2
BNL1679 0 2 0 0 0 0 0 2 1 0 0 1 0 0 0 0 2 2 2 2 2 2 2 2 2 2 0
2 0 0 0 0 0 0 1 0 2 2 0 0 2 1 1 2 2 0 0 0 2 2 0 0 0 0 0 0 0 0
0 0 0 0 0 0 2 2 0 2 2 1 0 1 0 0 0 0 0 0 1 1 1 0 0 0 0 0 0 1 0
0 0 0 0 0 0 1 1 1 1 2 0 1 1 0 0 0 0 1 2 1 0 0 0 2 0 0 1 0 2 2
0 2 0 0 1 0 1 1 1 1 1 1 0 0 2
CIR148 0 0 0 0 0 0 0 2 1 0 0 1 0 0 0 0 2 2 2 2 0 2 2 2 2 2 0 2
0 0 0 0 0 0 1 0 2 2 0 0 2 1 1 2 2 0 0 0 2 2 0 0 0 0 0 0 0 0 0
0 0 0 0 0 2 2 0 2 2 1 0 2 0 0 0 0 0 0 1 1 1 0 0 0 0 1 0 1 0 0
0 0 0 0 0 1 2 1 1 2 0 1 1 0 0 0 0 1 2 1 0 0 0 2 0 0 1 0 2 2 0
2 0 0 1 0 2 1 2 1 1 1 0 0 2
NAU5047 0 0 0 0 0 0 0 2 1 0 0 1 0 0 0 0 2 1 2 1 0 1 0 1 0 1 0
2 0 0 0 0 0 0 1 0 2 2 0 0 1 1 1 1 1 0 0 0 0 0 0 0 0 0 0 0 0 0
0 0 0 0 0 0 2 0 0 0 1 1 0 1 0 0 0 0 0 0 0 0 0 0 0 0 0 2 0 2 0
0 0 0 0 0 0 1 2 1 1 2 0 1 1 0 0 0 0 1 2 0 0 0 0 0 0 0 1 0 2 2
0 2 0 0 2 0 1 0 1 0 0 1 0 0 2
CM85 0 0 1 1 1 0 0 5 5 5 0 1 5 5 5 1 5 0 2 5 0 5 0 1 0 5 0 2 0
1 1 0 2 0 2 0 1 1 0 1 1 1 1 1 1 1 0 0 0 0 0 0 1 0 1 0 5 5 0 5
0 0 0 0 2 0 0 0 1 2 0 2 1 0 0 0 0 5 0 0 0 0 0 0 0 5 0 1 0 0 0
0 0 1 0 1 2 5 1 5 5 1 1 0 5 0 5 5 2 0 0 0 5 5 0 0 2 0 2 5 5 2
0 5 5 1 1 0 2 0 0 5 0 0 5
JESPR165 1 0 0 1 2 1 0 0 0 5 0 0 0 0 1 0 0 0 0 0 5 0 0 0 0 0 1
0 0 0 1 0 1 0 0 1 0 0 0 0 0 0 2 2 0 1 0 0 0 1 1 1 2 2 0 1 1 5
2 5 0 1 2 0 0 0 2 0 0 0 2 0 0 2 1 1 1 0 1 1 2 2 2 1 2 5 0 0 0
1 0 0 2 1 1 1 0 0 2 0 0 1 1 1 2 2 2 0 2 1 2 2 2 1 2 2 1 2 2 5
2 0 2 1 0 0 0 1 0 1 0 2 2 0 2
BNL3502 1 0 0 1 2 1 0 0 1 1 0 0 0 0 1 0 2 1 0 0 2 2 1 1 1 1 1
2 1 0 1 0 1 0 0 1 0 0 0 0 0 0 2 2 1 1 0 0 0 1 1 1 2 2 0 1 1 2
2 0 0 1 2 1 2 0 2 0 0 1 2 0 0 2 1 1 1 0 1 1 2 2 2 1 2 0 0 0 0
1 0 0 1 2 1 0 0 0 2 0 1 1 1 1 2 2 2 1 2 1 2 2 0 1 1 2 1 2 2 0
2 0 2 1 0 0 1 1 0 1 0 2 2 0 0
TMB0803 1 0 0 1 2 1 0 0 1 1 0 0 1 0 1 0 2 1 0 0 0 2 5 1 1 1 1
2 1 1 1 0 1 0 0 1 0 0 0 0 0 1 2 2 1 1 0 0 0 1 1 1 2 2 0 1 1 2
2 1 0 1 2 1 2 0 2 0 0 1 2 1 0 2 1 1 1 5 1 1 2 2 2 2 2 0 0 0 0
1 0 0 1 1 1 0 0 0 2 0 1 1 1 1 1 1 2 0 2 1 2 2 1 1 1 2 1 1 1 5

```
2 1 2 1 0 0 0 1 1 1 0 2 2 0 0
NAU2336 1 0 0 1 1 0 0 0 2 5 0 0 1 1 1 0 2 0 1 0 0 1 1 1 1 1 0
2 1 0 1 0 1 0 0 1 0 0 0 0 1 1 2 1 1 1 0 0 0 0 1 1 2 1 0 1 1 5
0 5 1 1 2 1 1 0 2 0 0 1 2 1 1 2 2 1 1 0 1 1 2 2 2 1 2 0 0 0 0
1 0 0 0 1 2 1 0 0 2 0 0 2 1 2 5 2 2 0 2 1 2 2 2 0 2 2 1 2 1 2
2 1 2 1 0 0 0 2 1 1 1 0 1 1 1
TMB1181 2 1 0 0 0 1 0 5 5 5 1 1 0 2 0 0 5 2 5 1 0 0 2 1 1 2 1
0 1 0 0 2 0 2 2 1 1 1 0 0 0 2 2 2 1 0 0 2 2 2 0 1 0 1 0 1 2 0
0 2 0 1 1 1 0 1 1 1 1 1 2 1 1 2 1 2 1 1 2 2 2 1 1 1 1 0 0 5 0
0 0 0 0 1 2 2 2 2 2 0 1 1 2 1 0 1 2 1 2 2 2 1 1 2 0 2 1 2 2 0
0 2 2 0 0 1 1 2 1 2 2 0 2 0 2
BNL786 2 1 0 0 0 1 0 2 1 0 1 1 0 2 0 0 2 2 0 1 2 0 2 1 1 2 1 0
1 0 0 2 0 2 2 1 1 1 0 0 0 2 2 2 1 0 0 2 2 2 0 1 0 1 0 1 2 5 0
5 0 1 1 1 0 1 1 1 1 1 2 1 1 2 1 2 2 1 2 2 2 1 0 0 1 0 0 0 0 0
0 0 0 1 2 2 2 2 2 0 1 1 2 1 0 1 2 1 2 2 2 1 1 2 0 2 1 2 2 0 0
2 2 0 0 1 1 2 1 2 2 0 2 0 2
BNL1350 2 2 0 0 0 1 0 2 1 0 1 1 2 2 0 0 2 2 0 2 2 0 2 1 1 2 2
2 1 0 0 2 0 2 2 2 1 2 1 0 0 2 2 2 2 0 0 2 1 1 0 1 0 2 0 2 2 0
2 2 2 2 1 2 0 1 1 0 1 1 2 0 1 2 2 0 0 0 0 0 0 1 0 1 1 0 2 0 2
0 0 0 2 1 2 2 2 2 2 2 0 2 2 2 0 1 2 1 2 0 1 1 1 0 0 2 1 2 1 2
0 2 1 0 0 0 1 0 0 0 0 0 2 0 2
BNL3902 2 2 0 0 0 1 0 2 1 0 2 1 0 2 0 0 2 2 0 2 2 0 2 1 1 2 2
0 1 0 0 2 0 2 2 2 1 2 1 0 0 2 2 2 2 0 0 2 1 1 0 1 0 2 0 2 2 1
2 2 2 2 1 2 0 1 0 0 1 1 2 0 1 2 2 0 0 0 0 0 0 1 0 0 1 0 2 0 2
0 0 0 2 1 2 2 2 2 2 2 0 2 2 2 0 1 2 1 2 0 0 1 0 0 0 2 1 2 1 2
1 0 1 0 0 0 1 0 0 0 0 2 2 0 2
TMB0375 2 2 0 0 0 2 0 2 1 0 2 1 0 2 0 0 5 1 0 2 2 0 2 1 1 2 2
0 1 0 0 2 0 0 0 5 1 1 1 0 0 2 2 2 2 0 0 2 1 1 0 1 0 2 0 2 2 1
2 2 2 2 1 2 0 1 0 0 1 1 2 0 1 2 2 0 0 0 5 0 0 1 0 1 1 0 2 0 2
0 0 2 2 2 2 2 2 2 2 2 1 2 2 2 0 1 2 1 2 0 0 1 0 0 0 2 1 2 1 2
1 0 1 0 0 0 1 0 0 0 0 2 2 0 2
BNL4082 2 2 0 0 0 2 0 2 1 0 2 1 0 2 0 0 2 2 2 2 2 2 2 2 2 2 2
2 1 0 0 2 0 2 2 2 1 1 1 0 2 2 2 2 2 0 0 2 1 1 0 1 0 2 0 2 2 1
2 5 2 2 1 2 2 2 0 2 2 1 2 1 1 2 2 0 0 1 0 0 0 1 0 0 1 0 2 0 2
0 0 0 2 1 2 2 2 2 2 2 1 2 2 2 0 1 2 1 2 0 0 1 0 0 0 2 1 2 1 2
1 0 1 0 0 1 1 0 1 0 0 2 2 0 2
JESPR298 1 1 0 0 0 2 0 2 1 0 2 1 0 2 0 0 2 2 2 2 2 2 2 2 2 2 2
2 1 0 0 2 0 2 1 2 1 1 1 0 2 2 2 2 2 0 0 2 1 1 0 1 0 2 0 2 2 1
2 2 2 2 1 2 2 2 0 2 2 1 2 1 1 2 2 1 1 1 2 0 2 1 0 1 1 0 2 0 2
0 1 1 2 1 2 2 2 2 2 2 1 2 2 2 0 1 2 1 2 1 0 1 0 1 0 2 1 2 1 2
1 0 1 0 0 1 1 2 1 1 1 2 2 0 2
JESPR180 1 1 0 0 0 2 0 2 0 0 2 1 0 2 0 0 2 2 2 2 2 2 2 2 2 2 2
2 1 0 0 2 0 2 1 2 1 1 1 0 2 2 2 2 2 0 0 2 1 1 0 1 0 2 0 2 2 1
2 2 2 2 1 2 2 2 0 2 2 1 2 1 1 2 2 1 1 1 2 0 2 1 0 1 1 0 2 0 2
0 1 1 2 1 2 2 2 2 2 2 1 2 2 2 0 1 2 1 2 1 0 1 0 1 0 2 1 2 1 2
1 0 1 5 0 1 1 0 1 1 1 2 2 0 2
TMB0301 1 1 0 0 0 2 0 2 0 0 2 1 0 2 0 0 2 2 2 2 2 2 2 2 2 2 2
2 1 0 0 2 0 2 1 2 1 1 1 0 2 2 2 2 2 0 0 2 1 1 0 1 0 2 0 2 2 1
2 2 2 2 1 2 2 2 0 2 2 2 2 2 2 2 2 1 1 2 2 0 2 1 0 1 1 0 2 0 2
0 1 1 2 0 2 2 2 2 2 2 2 2 2 2 0 1 2 0 2 1 0 1 0 1 0 2 2 2 1 2
1 0 1 0 0 2 2 0 1 1 1 2 2 0 2
TMB0201 1 1 0 0 5 1 0 5 0 0 2 1 5 2 0 0 5 5 2 5 5 2 5 2 2 5 2
2 1 0 0 2 0 2 1 2 1 1 0 0 2 2 2 2 2 0 0 2 0 0 0 1 0 2 0 2 2 2
2 2 2 2 1 2 2 2 0 2 2 1 5 1 1 2 2 5 5 5 1 0 2 1 1 5 5 0 0 0 0
0 1 1 2 1 5 2 2 5 2 0 0 2 2 5 5 5 2 1 1 1 0 1 0 5 0 2 0 2 1 2
1 0 2 0 5 1 1 1 1 1 1 2 0 0 5
TMB1664 1 1 0 0 0 1 1 2 1 0 2 1 0 1 0 0 2 2 2 2 2 2 2 2 2 2 2
```

2 1 0 0 2 0 2 0 2 1 1 0 0 2 2 2 2 2 0 2 2 0 0 0 1 0 2 2 2 2 1
2 5 2 2 1 2 2 2 0 2 2 1 2 1 1 2 2 1 1 1 1 0 2 1 2 0 1 0 0 0 0
0 1 1 2 1 2 2 2 5 2 0 1 2 2 2 0 1 2 1 1 0 0 1 0 1 0 2 1 2 1 2
1 2 2 0 0 1 1 1 1 1 1 2 0 0 1
TMB1660 1 1 0 0 0 1 1 2 1 0 2 1 0 2 0 0 2 2 2 2 2 2 2 2 2 2 2
2 1 0 0 2 0 2 0 2 1 1 0 0 2 2 2 2 2 0 2 2 0 0 0 1 0 2 2 2 2 1
2 2 2 2 1 2 2 2 0 2 2 1 2 1 1 2 2 1 1 2 1 0 2 1 1 0 1 0 1 1 0
0 1 1 2 1 2 2 2 2 2 0 1 2 2 2 0 1 2 1 1 1 0 1 0 1 0 2 1 2 1 2
1 2 2 0 0 1 1 1 1 1 1 2 0 0 1
JESPR152 2 1 0 0 0 0 1 2 1 0 0 2 0 0 0 0 2 2 2 2 2 2 2 2 2 2 2
2 2 0 0 1 0 2 0 2 2 2 2 0 2 2 2 1 2 0 1 2 0 0 0 2 0 2 2 2 2 1
2 2 2 2 1 2 2 2 0 2 2 1 2 1 1 2 2 1 2 0 1 0 1 1 2 1 1 0 0 0 0
1 1 1 2 1 2 0 2 2 2 0 1 2 2 1 0 1 1 1 1 0 0 1 0 1 2 0 0 1 1 1
1 1 2 1 0 1 1 2 1 2 1 2 2 2 1
BNL2920 1 1 5 5 0 1 2 2 0 0 1 1 0 5 5 5 5 5 5 5 0 0 0 5 5 0 0
0 5 5 5 5 0 2 0 2 1 1 0 0 2 2 2 2 2 0 2 2 0 0 0 1 0 2 2 5 5 5
5 2 2 2 5 2 2 5 5 5 1 0 0 2 0 0 0 0 1 1 0 2 1 0 1 0 5 5 0 0
5 1 1 5 5 2 5 2 2 2 0 1 2 2 2 0 1 2 1 1 1 0 5 0 1 0 2 1 2 1 2
1 2 2 0 0 2 1 1 1 1 1 2 0 0 1
JESPR32 2 1 1 0 1 0 2 1 1 1 0 2 1 0 2 2 2 2 0 1 1 1 1 1 0 0 1
2 1 1 2 2 0 0 0 2 2 1 2 0 1 2 2 1 1 0 2 2 2 1 2 2 1 1 2 0 2 2
1 0 0 2 1 1 1 1 0 1 1 1 1 1 0 1 1 0 0 0 0 0 0 1 2 1 1 0 0 2 2
2 0 0 0 1 2 2 2 2 2 0 0 2 2 1 1 0 1 0 2 0 2 2 0 0 0 2 0 1 1 1
1 1 0 2 1 0 0 0 0 0 0 2 0 0 0
JESPR128 2 5 1 0 1 0 2 5 1 5 0 2 1 0 2 2 2 2 0 1 5 1 1 1 0 0 1
2 1 1 2 2 0 0 0 2 2 1 2 0 1 2 2 1 1 0 2 2 2 1 2 2 1 1 2 0 2 2
1 5 0 2 1 1 1 1 0 1 1 1 1 1 0 1 1 0 0 0 0 0 0 1 2 2 1 0 0 0 0
0 0 0 0 1 2 2 2 2 2 0 0 2 2 1 1 0 1 0 2 0 2 2 0 0 0 2 0 1 2 1
1 1 1 2 1 0 0 0 0 0 0 2 2 2 2
JESPR237 2 1 1 0 1 0 2 1 1 1 0 2 1 0 2 2 2 2 0 1 1 1 1 1 0 0 1
2 1 1 2 2 0 0 0 2 2 1 2 0 1 2 2 1 1 0 2 2 2 1 2 2 1 1 2 0 2 2
1 0 0 2 1 1 1 1 0 1 1 1 1 1 0 1 1 0 0 0 0 0 0 1 2 1 1 0 0 0 0
0 0 0 0 1 2 2 2 2 2 0 0 2 2 1 1 0 1 0 2 0 2 2 0 0 0 2 0 1 2 1
1 1 1 2 1 0 0 0 0 0 0 2 2 2 2
TMB1271 2 1 1 0 1 0 2 1 1 1 0 2 1 0 2 2 2 2 0 1 1 1 1 1 0 0 1
2 1 1 2 2 0 0 0 2 2 1 2 0 1 2 2 1 1 0 2 2 2 1 2 2 1 1 2 0 2 2
1 0 0 2 1 1 1 1 0 1 1 1 1 1 0 1 1 0 0 0 0 0 0 1 2 1 1 0 0 0 0
0 0 0 0 1 2 2 2 2 2 0 0 2 2 1 1 0 1 0 2 0 2 2 0 0 0 2 0 1 2 1
1 1 1 2 1 0 0 0 0 0 0 2 2 2 2
GH2 2 1 1 0 1 0 2 1 1 1 0 2 1 0 2 2 2 2 0 1 1 1 1 1 0 0 1 2 1
1 2 2 0 0 0 2 2 1 2 0 1 2 2 1 1 0 2 2 2 1 2 2 1 1 2 0 2 2 1 0
0 2 1 1 1 1 0 1 1 1 1 1 1 1 1 0 0 0 0 0 0 1 2 1 1 0 0 0 0 0 0
0 0 1 2 2 2 2 2 0 0 2 2 1 1 0 1 0 2 0 2 2 0 0 0 2 0 1 2 1 1 1
1 2 1 0 0 0 0 0 0 2 2 2 2
JESPR297 2 1 1 0 1 0 2 1 1 1 0 2 5 0 2 2 2 2 0 1 1 1 5 1 0 0 1
2 1 1 2 2 0 0 0 2 2 1 2 0 1 2 2 1 1 0 2 2 2 1 2 2 1 1 2 0 2 2
1 0 0 2 1 1 1 1 0 1 1 1 1 1 0 1 1 0 0 0 0 0 0 1 2 1 1 0 0 0 0
0 0 0 0 1 2 2 2 2 2 0 0 2 2 2 1 5 1 0 2 0 2 2 0 0 0 2 0 1 2 1
1 0 1 2 1 0 0 0 0 0 0 2 2 2 2
TMB1409 2 1 1 0 1 0 2 2 1 5 0 2 1 0 2 2 2 2 0 1 1 0 1 1 0 0 1
1 1 1 2 2 0 0 0 2 2 2 2 0 1 2 1 1 1 0 2 2 2 1 2 2 1 1 2 0 2 2
1 5 0 2 1 1 1 1 0 2 1 1 1 1 0 1 1 0 0 0 0 0 0 1 2 2 1 0 0 0 0
0 0 0 0 0 1 2 2 0 2 0 0 2 2 1 0 0 1 1 2 0 2 2 0 0 0 2 0 2 2 1
1 1 1 2 0 0 1 0 0 0 0 2 2 2 2
BNL3008 2 1 1 0 1 0 2 2 1 1 0 2 1 0 2 2 2 2 0 1 1 0 1 1 1 0 1
1 1 1 2 2 0 0 0 2 2 2 2 0 1 2 1 1 1 0 2 2 2 1 2 2 1 1 2 0 2 2
1 0 0 2 1 1 1 1 0 2 1 1 1 1 1 1 1 0 0 0 0 0 0 1 2 2 1 0 0 0 0

1 1 1 0 0 2 2 2 0 2 0 0 2 1 1 1 0 1 1 2 0 2 2 0 0 0 2 0 2 2 1
1 1 1 2 0 0 1 0 0 0 0 2 2 2 2
BNL2734 2 1 1 0 1 0 2 2 1 1 0 2 1 0 2 2 2 2 0 1 1 0 1 1 1 0 1
1 1 1 2 2 0 0 0 2 2 2 2 0 1 2 1 1 1 0 2 2 2 1 2 2 1 1 2 0 2 2
1 0 0 2 1 1 1 1 0 2 1 1 1 1 0 1 1 0 0 0 0 0 0 1 2 2 1 0 0 0 0
1 1 1 0 0 2 2 2 0 2 0 0 2 1 1 1 0 1 1 2 0 2 2 0 0 0 2 1 2 2 1
1 1 1 2 0 1 1 0 0 0 0 2 2 2 2
BNL3065 2 0 2 2 2 0 1 1 2 2 0 2 2 0 2 2 2 2 0 0 0 1 1 1 1 0 1
1 2 2 2 5 2 0 0 2 2 1 0 2 1 2 1 2 1 2 1 2 2 2 2 2 2 2 2 2 1 2
0 1 1 1 2 1 1 1 2 1 1 0 0 0 0 2 1 2 0 1 1 1 1 2 2 2 2 1 1 1 0
1 0 0 2 1 0 2 2 1 2 1 0 2 2 1 2 2 2 0 0 2 2 2 0 0 0 2 0 2 2 0
2 1 2 2 0 0 0 0 0 2 0 0 2 2 1
TMB2036 5 1 2 2 2 0 0 1 2 2 0 2 2 0 2 2 2 2 1 0 1 1 1 1 1 0 1
1 2 2 2 1 2 0 0 2 2 1 0 2 1 2 1 2 1 2 0 2 2 2 2 2 2 1 0 2 1 2
0 1 1 1 2 1 1 0 2 1 1 0 0 0 1 2 1 1 0 1 1 1 1 2 2 2 2 1 2 1 0
1 0 0 2 0 0 1 2 1 2 1 1 2 2 1 2 2 2 0 1 2 2 2 0 0 1 2 1 2 2 2
2 1 2 2 0 1 0 0 1 2 0 0 2 2 1
TMB2068 2 1 2 2 2 0 0 1 1 2 0 2 2 0 2 2 2 2 1 2 1 1 1 1 1 0 1
1 2 2 2 1 2 0 0 2 2 1 0 2 1 2 1 2 1 2 0 2 2 2 2 2 2 1 0 2 1 2
0 1 1 1 2 1 1 0 2 1 1 0 0 0 1 2 1 1 0 1 1 1 1 2 2 2 2 1 2 1 0
1 0 0 2 0 0 1 2 5 2 1 1 2 2 1 2 2 2 0 1 2 2 2 0 0 1 2 1 1 2 2
2 1 2 2 0 1 0 0 1 2 0 0 2 2 1
JESPR236 2 0 0 0 0 1 2 2 1 2 2 5 5 5 5 5 0 1 2 2 0 1 1 2 1 1 0
0 2 0 0 0 0 1 1 0 2 2 2 0 1 2 2 1 2 0 1 1 1 1 0 0 0 2 0 1 2 1
1 2 0 0 1 2 1 0 0 1 0 1 1 1 1 1 1 2 2 1 2 2 2 1 0 2 1 2 0 1 2
0 0 0 0 0 2 0 2 2 0 0 1 2 2 1 1 0 1 1 2 2 2 2 0 2 1 1 1 0 2 0
0 1 1 0 2 1 1 2 1 2 2 1 2 0 2
JESPR218 2 0 0 0 0 1 2 2 1 0 0 2 0 2 0 0 0 1 2 2 0 1 1 2 1 1 0
0 2 0 0 0 0 1 1 0 2 2 2 0 1 2 2 1 2 0 1 1 1 1 0 0 0 2 0 1 2 1
1 2 1 0 1 2 1 0 0 1 0 1 1 1 1 1 1 2 2 1 2 2 2 1 0 2 1 2 0 1 2
0 0 0 0 0 2 0 2 5 0 0 1 2 2 1 1 0 1 1 2 2 2 2 0 2 1 5 1 0 2 0
0 1 1 0 2 1 1 2 1 2 2 1 2 0 2
CM3 2 0 0 0 0 1 2 2 1 0 0 2 0 2 0 0 0 1 2 2 0 1 1 2 1 1 0 0 2
0 0 0 0 1 1 0 2 2 2 0 1 2 2 1 2 0 1 1 1 1 0 0 0 2 0 1 2 1 1 2
1 0 1 2 1 0 0 1 0 1 1 1 1 1 1 2 2 1 2 2 2 1 0 0 2 2 0 1 2 0 0
0 0 0 2 0 2 2 0 0 1 2 2 1 5 0 5 1 2 2 2 2 0 2 1 1 1 0 2 0 0 1
1 0 2 1 1 2 1 2 2 1 2 0 2
CM42 2 0 0 0 0 1 2 2 1 0 0 2 0 2 0 0 0 1 2 2 0 1 1 2 1 1 0 0 2
0 0 0 0 1 1 0 2 2 2 0 1 2 2 1 2 0 1 1 1 1 0 0 0 2 0 1 2 5 1 5
1 0 1 2 1 0 0 1 0 1 1 1 1 1 1 2 2 1 2 2 2 1 0 0 2 2 0 1 2 0 0
0 0 0 2 0 2 2 0 0 1 2 2 1 5 0 1 1 2 2 2 2 0 2 1 1 1 0 2 0 0 1
1 0 2 1 1 2 1 2 2 1 2 0 2
CM209 0 0 0 0 0 1 2 5 1 5 0 2 0 2 0 0 0 1 1 2 0 1 1 2 1 5 2 0
0 0 0 0 0 2 1 2 2 2 1 0 1 2 2 1 2 0 2 1 0 1 0 0 0 2 0 2 2 1 2
5 2 2 0 2 2 0 0 1 0 1 2 1 1 2 2 0 0 0 0 0 0 1 0 5 2 2 0 1 2 0
0 0 2 0 2 0 2 2 0 0 1 2 2 2 1 0 1 1 2 0 2 2 0 0 1 1 1 0 2 0 0
1 1 0 2 1 1 0 1 0 0 1 2 0 2
TMB1645 0 0 0 0 0 1 2 2 1 0 1 2 0 2 0 0 0 1 1 2 0 1 1 2 1 1 2
0 0 0 0 0 0 2 1 2 2 2 0 0 1 2 2 1 2 0 2 2 0 1 0 0 0 2 0 2 2 1
2 5 2 2 0 2 2 0 0 1 0 1 2 1 1 2 2 0 0 0 0 0 0 1 0 0 2 2 0 1 1
0 0 0 2 0 2 0 2 2 0 0 1 2 2 2 1 0 1 1 0 0 2 2 0 0 1 1 1 0 2 0
0 1 0 0 2 1 1 0 1 0 0 1 1 0 0
TMB1489 0 0 0 1 0 1 0 2 1 0 2 1 0 1 0 0 2 1 1 1 0 1 2 1 0 1 2
2 0 0 0 1 0 2 0 2 2 2 0 0 2 2 0 2 0 0 0 1 1 1 0 0 0 2 0 2 2 1
2 2 2 2 0 2 0 2 2 0 1 0 2 1 1 2 2 0 2 1 0 0 2 1 2 2 1 2 0 1 1
1 0 0 2 0 1 0 1 1 0 2 1 2 2 1 2 1 1 1 0 2 2 1 1 1 2 2 0 0 2 0
2 0 0 2 1 1 1 0 1 1 1 0 0 2 0

BNL3977 0 0 0 0 0 1 0 2 1 0 2 1 0 1 0 0 2 1 1 1 0 1 2 1 0 1 2
2 0 0 0 1 0 2 0 2 2 2 0 0 2 2 0 2 0 0 0 1 1 1 0 0 0 2 0 2 2 1
2 2 2 2 0 2 0 2 2 0 1 1 2 2 1 2 2 0 2 1 0 0 2 1 2 2 1 2 0 1 1
1 0 0 2 0 1 0 1 1 0 2 1 2 2 1 2 1 1 2 0 2 2 1 1 1 2 2 1 0 2 0
2 0 0 2 1 1 2 0 1 1 1 0 0 2 0
BNL3875 1 0 0 0 0 1 0 2 1 0 2 0 0 1 0 0 0 1 1 1 0 0 2 1 0 2 2
1 1 0 0 1 0 1 0 2 2 2 0 0 2 2 0 2 0 0 2 1 1 1 0 0 0 0 1 1 0 1
1 5 2 1 0 1 0 1 2 0 1 1 1 0 0 1 2 0 2 1 0 1 2 1 2 2 1 1 0 2 1
1 0 0 1 0 1 1 1 1 0 2 0 1 0 1 2 2 1 1 0 1 2 1 2 1 2 2 0 0 0 0
2 0 0 2 1 0 1 0 1 1 1 0 0 2 0
BNL4096 1 0 0 0 0 1 0 2 1 0 2 0 0 1 0 0 0 1 1 1 0 0 2 1 0 2 2
1 1 0 0 1 0 1 0 2 2 2 0 0 2 0 0 2 0 0 2 1 1 0 0 0 0 0 1 1 0 1
1 5 2 1 0 1 0 1 2 0 1 1 1 0 0 1 2 0 2 2 0 1 2 1 2 2 1 1 0 2 1
2 2 2 1 0 0 1 0 0 1 2 0 2 0 1 2 2 1 2 0 0 1 1 2 1 2 2 0 0 0 0
2 0 0 2 1 0 2 0 1 1 1 0 0 2 0
TMB0366 1 0 0 0 0 1 0 2 0 0 2 5 5 1 0 0 5 1 1 5 5 0 5 1 0 5 5
1 2 0 1 1 0 1 0 5 2 2 0 0 2 0 0 2 5 0 2 2 1 0 0 0 0 0 1 5 0 1
1 1 2 1 0 1 0 1 2 0 1 1 1 0 5 1 2 0 2 5 0 1 2 1 2 1 1 1 0 5 2
2 2 2 2 5 0 1 0 0 5 2 5 2 0 1 2 5 1 2 0 0 1 1 2 5 2 2 0 0 0 0
2 0 5 2 2 0 2 0 1 1 1 0 0 2 0
BNL852 1 0 0 0 0 2 0 2 0 0 2 0 0 2 0 0 2 2 2 2 0 2 2 2 0 2 2 2
1 0 0 2 0 1 1 2 2 2 1 0 2 0 0 2 2 0 2 1 0 0 0 0 0 0 1 1 0 1 1
1 2 1 0 2 2 2 2 2 2 1 1 1 1 1 1 2 0 2 2 0 1 2 1 2 1 1 2 2 2 2
2 2 1 1 0 0 0 0 0 2 1 2 0 0 2 2 1 2 0 0 0 1 2 1 2 2 1 0 0 0 2
0 0 2 0 1 2 0 2 1 0 0 0 2 0
TMB0189 1 0 0 0 0 2 0 2 0 0 2 0 0 2 0 0 2 2 2 2 0 2 2 2 2 2 2
2 1 0 0 2 0 1 1 2 2 2 1 0 2 0 0 2 2 0 2 1 0 0 0 0 0 0 2 1 0 1
1 1 2 1 0 1 2 2 2 2 2 1 1 1 1 1 2 0 2 2 0 1 2 1 2 0 1 2 2 2 2
2 2 2 1 1 0 0 0 0 0 2 1 2 0 1 2 2 1 2 0 0 0 1 2 1 2 2 1 0 0 0
2 0 0 2 0 1 2 0 2 1 0 0 0 2 0
GH109 1 0 0 0 0 2 0 2 1 0 2 0 0 2 0 0 2 2 2 2 0 2 2 2 2 2 2 2
1 0 0 1 0 1 1 2 2 2 1 0 2 0 0 2 2 0 2 1 0 0 0 0 0 0 1 1 0 1 1
0 2 1 0 1 2 2 2 2 2 1 1 1 1 1 1 2 0 2 2 0 1 2 1 2 1 1 2 2 2 2
2 2 1 0 0 0 0 0 0 2 0 2 0 1 2 2 1 1 0 0 0 1 2 1 2 2 0 0 0 0 2
0 0 2 0 0 1 0 1 1 0 0 0 2 0
NAU3935 1 2 0 0 0 2 0 2 1 0 2 0 0 2 0 0 2 2 2 2 2 2 2 2 2 2 0
2 2 0 0 2 0 0 1 0 2 2 0 0 2 0 0 1 1 0 2 1 0 0 0 0 0 0 1 0 0 1
0 5 0 0 0 0 2 2 2 2 2 0 0 0 1 0 0 0 2 1 0 2 2 1 0 0 0 2 2 2 2
2 2 2 0 0 0 0 0 0 0 2 0 2 0 1 1 2 1 1 0 0 0 1 2 1 2 2 0 0 0 0
2 2 0 0 0 0 1 0 0 1 0 1 0 1 0
BNL285 1 2 2 0 0 2 1 2 1 0 2 0 0 2 0 0 2 2 2 2 2 2 2 2 2 2 0 2
2 0 0 2 0 2 1 0 0 1 0 0 2 0 0 2 2 0 2 2 0 0 0 0 0 0 1 0 0 1 0
0 0 0 0 0 2 2 2 2 2 0 0 0 0 0 0 2 0 0 2 0 0 1 0 0 0 2 2 2 2 2
2 2 0 0 0 0 0 0 0 0 0 2 5 1 1 5 1 1 0 2 0 1 2 1 1 2 0 0 0 1 2
2 0 0 0 0 1 2 0 1 2 1 0 1 0
TMB1599 1 2 2 2 2 0 1 1 2 2 0 0 2 1 2 2 2 2 2 2 2 2 2 2 2 2 0
2 2 2 2 2 2 0 1 0 2 1 1 2 2 0 0 2 2 2 2 2 0 0 2 0 2 0 1 0 0 0
0 0 0 0 0 0 2 2 2 2 2 1 0 0 1 0 0 1 1 0 2 0 0 1 0 0 0 1 0 2 2
0 0 0 0 0 0 0 0 0 0 2 1 2 0 1 1 1 1 1 2 2 0 1 1 1 1 1 0 0 0 1
2 2 0 0 0 1 1 2 0 1 2 1 1 1 0
GH71 0 2 2 2 2 2 0 1 5 2 2 0 0 2 1 2 2 2 2 2 2 2 2 0 2 2 2 0 2 2
2 2 2 2 0 1 0 2 1 1 2 2 0 0 2 2 2 2 2 0 0 2 0 2 0 1 0 0 0 0 0
0 0 0 0 2 2 2 2 2 1 0 1 1 0 0 1 1 5 2 0 0 1 0 0 0 1 0 2 2 0 0
0 0 0 0 0 0 0 0 2 1 2 0 1 1 2 1 1 2 2 0 1 1 1 1 1 0 0 0 1 2 2
0 0 0 1 1 2 1 1 2 1 1 1 0
GH82 2 1 2 2 2 0 1 2 2 2 0 2 2 0 2 2 0 0 0 0 2 0 0 0 0 0 1 0 2
2 2 1 2 1 1 1 1 1 1 2 0 1 1 0 0 2 5 0 2 1 2 2 2 0 1 2 1 0 2 0

1 1 0 0 0 0 0 0 0 0 2 0 0 1 0 2 2 1 2 2 2 0 0 0 0 2 2 2 2 2 2
2 1 0 1 0 1 0 0 2 0 1 1 1 0 0 0 0 2 2 0 0 2 2 2 0 1 0 0 0 0 1
0 0 0 0 0 2 0 2 2 2 2 0 2
BNL4108 0 1 2 2 2 0 0 2 2 2 0 0 2 0 2 2 0 0 0 0 2 0 0 0 0 0 1
0 0 2 2 1 2 1 1 1 2 2 0 2 0 1 1 0 0 2 0 0 2 1 2 1 2 0 0 2 1 0
2 5 1 1 0 0 0 0 0 0 0 0 2 0 0 1 1 0 1 0 0 2 2 0 0 0 0 2 2 2 2
2 2 2 1 0 1 0 1 0 0 2 0 1 1 1 0 0 0 1 2 2 0 0 2 0 2 0 1 0 0 0
0 1 0 0 0 0 1 1 0 0 1 0 2 0 2
TMB1277 0 1 2 2 2 0 0 2 2 2 0 0 2 0 2 2 0 0 0 0 2 0 0 0 0 0 1
0 0 2 1 1 2 1 1 1 2 2 0 2 0 1 1 0 0 2 0 0 2 1 2 1 2 0 0 2 1 0
2 1 1 1 0 0 0 0 0 0 0 0 2 0 0 1 1 0 1 0 0 2 2 0 0 0 0 2 2 2 2
2 2 2 1 0 1 0 1 0 0 2 0 1 1 1 0 0 0 1 2 2 0 0 2 0 2 0 1 0 0 0
0 1 0 0 0 0 1 1 0 0 1 0 2 0 2
BNL2884 0 1 2 1 1 0 0 2 2 0 0 0 1 0 0 0 0 0 0 0 2 0 0 0 0 0 1
0 0 1 2 1 2 1 2 1 2 2 0 0 0 1 1 1 0 1 0 1 1 2 0 1 1 0 0 2 1 0
2 1 1 1 0 0 0 0 0 0 0 1 1 1 0 1 1 0 5 0 0 2 1 0 0 0 0 2 2 2 2
0 0 0 0 0 1 0 1 0 0 2 0 0 0 1 0 0 0 1 2 2 0 0 0 1 0 0 1 0 0 0
0 2 0 0 0 1 1 1 0 0 1 0 2 0 2
BNL3948 2 0 2 2 2 0 1 2 1 2 0 1 2 0 2 2 2 1 1 0 0 1 1 0 2 2 1
1 1 2 2 1 2 2 0 1 0 2 1 2 1 0 1 1 0 2 2 2 1 2 2 1 2 0 2 1 1 5
1 5 2 1 1 0 1 1 1 0 2 0 1 0 0 1 1 2 1 1 0 2 2 1 0 0 0 2 2 0 0
0 0 0 1 1 2 2 1 1 2 1 0 2 1 2 5 2 1 1 0 2 2 1 0 2 2 1 0 1 0 2
0 0 0 0 1 0 0 1 0 1 2 0 0 0 0
GH54 2 0 2 0 0 0 1 1 1 2 0 0 1 5 5 5 5 1 1 0 0 1 1 5 2 2 1 0 1
0 1 1 5 2 0 0 0 2 2 0 1 0 1 1 0 5 5 2 0 1 5 5 1 0 2 1 1 0 1 5
2 1 1 5 1 1 5 0 2 5 1 0 5 1 1 2 1 1 5 5 2 5 5 0 5 5 1 5 0 0 0
5 1 1 2 2 1 1 2 1 0 2 1 2 0 1 1 0 0 2 2 1 1 2 2 0 0 1 0 1 0 0
0 0 1 0 0 1 0 1 2 0 0 0 0
GH59 2 0 2 0 0 0 2 1 0 2 0 0 1 0 0 1 2 1 1 0 0 1 2 0 2 2 1 0 1
0 1 1 1 2 0 0 0 2 2 1 1 0 1 1 0 2 2 2 1 2 1 1 1 0 2 1 0 0 1 1
2 1 1 1 1 1 1 0 2 1 1 1 1 1 1 0 0 0 0 0 0 1 0 1 1 2 2 0 0 0 0
0 1 1 2 2 1 2 2 1 0 2 1 2 0 0 0 1 0 0 2 1 1 0 2 1 1 1 1 1 0 0
0 0 1 1 1 0 0 0 0 0 0 0 0
TMB1629 2 0 2 0 0 0 2 1 1 2 0 1 1 0 0 1 2 1 1 0 0 1 1 0 2 2 1
0 1 0 1 1 1 2 0 0 0 2 2 1 1 0 1 1 0 2 2 2 2 2 1 1 1 0 2 1 0 0
1 5 2 1 1 1 1 1 1 0 2 1 1 1 1 1 1 0 0 0 0 0 0 1 0 0 1 2 2 0 0
2 1 1 1 1 2 2 1 1 2 1 0 2 1 2 0 0 0 1 0 0 1 2 1 0 2 1 0 1 1 1
0 0 0 0 1 1 1 0 0 0 0 0 0 0 0
BNL119 2 1 2 0 0 0 2 2 1 2 0 1 2 0 0 1 2 0 1 0 1 1 2 0 2 2 1 0
1 0 1 1 1 2 2 1 0 2 2 1 1 0 1 1 0 2 2 2 2 2 1 1 1 0 2 1 0 0 1
1 2 1 1 1 1 1 1 0 2 1 1 1 1 1 1 0 0 0 0 0 0 1 0 1 1 0 0 0 0 1
1 1 1 1 1 2 1 1 2 0 0 2 1 2 0 0 0 1 0 0 2 2 1 0 2 1 0 1 0 1 0
2 0 0 1 1 1 0 0 0 0 0 0 0 0
CM82 2 1 2 0 0 1 2 5 5 5 0 2 5 5 0 1 2 0 1 5 5 1 5 0 2 2 5 0 1
0 1 2 1 2 2 0 0 2 1 1 1 0 0 1 0 2 2 2 2 2 1 1 1 0 2 1 0 5 1 5
2 1 1 1 1 1 1 0 2 1 1 1 1 1 1 0 5 5 0 0 0 1 0 5 1 5 0 5 5 1 1
1 5 1 1 2 1 5 2 0 0 2 1 2 5 0 5 1 0 0 2 2 5 5 2 1 0 5 0 5 5 2
0 0 5 1 1 0 0 0 0 0 0 0 5
JESPR235 2 1 2 0 0 0 2 5 0 2 0 2 0 0 0 1 2 0 1 0 1 1 1 0 2 2 1
0 1 0 1 2 1 2 2 1 0 2 1 1 1 0 1 1 0 2 2 2 2 2 1 1 1 0 2 1 0 0
1 1 2 1 1 1 1 1 1 0 2 1 1 1 1 1 1 0 0 0 0 0 0 1 0 1 1 0 0 0 0
1 1 1 1 1 0 2 1 1 2 0 0 2 1 2 5 0 0 1 0 0 2 2 1 0 2 1 0 1 0 1
0 2 0 0 1 1 1 0 0 0 0 0 0 0 0
BNL169 2 1 2 0 0 0 2 2 0 2 0 2 0 0 0 1 2 0 1 0 1 1 1 0 2 2 1 0
1 0 1 1 1 2 2 1 0 2 2 1 1 0 1 1 0 2 2 2 2 2 1 1 1 0 2 1 0 0 1
1 2 1 1 1 1 1 1 0 2 1 1 1 1 1 1 0 0 0 0 0 0 1 0 1 1 0 0 0 0 1
1 1 2 1 5 2 2 2 2 0 0 2 1 2 0 0 0 1 0 0 2 2 1 0 2 1 0 1 0 1 0

2 0 0 1 1 1 0 0 0 0 0 0 0 0
GH48 2 1 2 0 0 0 2 2 0 2 0 2 0 0 0 1 2 0 1 0 1 1 1 0 2 2 1 0 1
0 1 1 1 2 2 1 0 2 2 1 0 0 2 1 0 2 2 2 2 2 1 1 1 0 2 1 0 0 1 1
2 1 1 1 2 1 1 0 2 1 1 1 1 1 1 0 0 0 0 0 0 1 0 1 1 0 0 0 0 2 1
1 2 1 5 2 2 2 2 0 0 2 1 2 0 0 0 1 0 0 2 2 1 0 2 1 0 1 0 1 0 2
0 0 1 1 1 0 0 0 0 0 0 0 0
JESPR158 2 2 2 1 2 1 1 1 1 1 2 0 0 1 0 1 0 1 1 1 0 0 1 2 0 1 0 0
1 2 2 1 2 1 0 2 0 1 2 1 2 1 2 2 2 1 2 2 1 2 2 1 1 0 0 0 0 0 2
0 0 0 0 0 0 1 1 1 2 1 0 0 0 0 0 0 0 1 0 1 1 2 0 2 2 0 2 1 2 0
2 2 2 0 0 2 1 2 2 2 1 0 1 1 0 0 1 2 1 0 1 1 1 0 0 0 1 1 1 1 1
1 2 1 2 0 0 1 2 0 1 2 0 0 2 0
CM23 2 2 2 1 1 0 1 2 1 2 0 0 1 0 0 0 1 2 1 0 2 1 2 0 1 0 0 1 1
1 2 2 1 0 2 0 0 2 1 2 1 2 2 2 0 2 2 1 2 2 1 2 0 0 0 0 0 0 0 5
0 0 0 0 1 1 0 2 2 0 0 0 0 0 0 0 1 0 1 1 1 0 1 0 0 2 1 2 0 2 2
2 0 0 2 0 2 2 0 2 0 1 1 1 0 0 0 0 0 1 0 0 0 0 0 0 0 0 1 2 0 2
0 0 0 0 0 2 0 1 1 0 0 2 0
TMB0400 1 2 2 1 1 0 0 2 1 1 0 1 0 0 1 1 1 1 2 1 2 2 1 1 2 1 2
2 1 1 1 1 1 2 0 2 1 2 1 2 1 1 0 2 1 2 1 2 2 2 1 1 0 2 1 2 2 2
2 2 2 2 0 2 1 2 1 1 1 0 2 0 1 2 2 2 2 1 2 2 2 1 2 1 1 2 1 1 0
2 2 2 2 0 2 0 1 2 0 1 1 1 1 1 1 1 1 1 0 2 0 1 1 2 0 2 0 2 1 2
1 1 1 0 1 0 0 2 1 2 2 1 1 2 1
JESPR118 1 2 2 1 1 0 0 2 1 1 0 1 0 0 1 1 1 1 2 1 2 2 1 1 2 1 2
2 1 1 1 1 1 2 0 2 1 2 1 2 1 1 0 2 1 2 1 2 2 2 1 1 0 2 1 2 2 2
2 2 2 2 0 2 1 2 1 1 1 0 2 0 1 2 2 2 2 1 2 2 2 1 2 1 1 2 1 1 0
2 2 2 2 0 2 0 1 2 0 1 1 1 1 1 1 1 1 1 0 2 0 1 1 2 0 2 0 2 1 2
1 1 1 0 1 0 0 2 1 2 2 1 1 2 1
TMB2038 1 2 2 1 1 0 0 1 1 0 0 2 1 0 0 0 1 1 2 1 2 2 1 1 2 1 0
2 1 1 2 2 1 0 0 0 1 2 2 2 1 1 0 2 1 2 1 1 2 2 1 1 0 0 0 0 0 0
0 0 0 0 0 0 1 2 0 0 1 0 0 1 1 0 0 2 2 1 2 2 2 0 2 0 0 2 1 1 0
2 2 2 0 0 2 0 0 2 0 2 1 1 1 1 0 0 0 1 0 2 0 0 0 2 0 0 0 0 1 2
0 2 0 0 1 1 0 2 1 2 2 0 0 2 1
BNL3649 2 2 2 1 1 2 0 2 2 1 1 1 0 2 1 1 1 2 2 1 2 2 1 1 2 1 0
2 2 1 1 1 1 0 0 0 1 2 2 2 0 0 0 2 1 2 1 2 2 2 1 2 0 0 1 0 0 2
0 0 0 0 0 0 1 2 1 1 1 0 0 1 2 0 0 2 2 2 2 2 2 1 2 1 0 2 1 1 0
2 2 2 0 0 2 0 0 2 0 1 1 1 0 1 0 0 1 1 0 2 0 1 1 2 0 1 1 2 1 2
1 1 1 0 1 1 0 2 1 2 2 1 1 2 2
BNL1551 2 1 2 1 1 2 0 2 1 1 1 1 0 2 1 1 1 2 2 1 2 2 1 1 2 1 0
2 2 1 1 1 1 0 0 0 1 2 2 2 0 0 0 2 1 2 1 2 2 2 1 2 0 0 1 0 0 2
0 0 0 0 0 0 1 2 1 1 1 0 0 0 1 0 0 2 2 1 2 2 2 1 2 0 0 0 0 0 0
2 2 2 0 0 2 0 0 2 0 0 1 1 0 1 0 0 1 1 0 2 0 1 1 2 0 1 0 2 1 2
1 1 2 2 2 1 0 2 1 2 2 1 1 2 2
JESPR110 0 0 1 1 1 0 2 2 1 0 0 2 5 0 1 1 0 1 1 1 0 0 5 1 1 1 0
0 0 2 0 1 2 1 0 0 2 2 2 1 0 0 0 2 0 1 2 1 2 2 0 0 2 0 2 2 2 1
1 1 1 1 1 1 1 1 2 1 1 1 1 1 1 0 2 2 2 0 2 2 2 2 1 2 2 1 2 2 1
1 1 1 2 1 0 1 0 0 1 2 1 1 1 1 5 5 0 1 0 2 2 2 0 2 0 5 2 2 0 2
1 0 2 1 2 1 1 2 1 2 2 0 0 2 0
TMB0382 0 1 0 1 2 0 2 0 0 0 0 1 0 0 1 2 0 1 1 1 0 0 0 1 1 1 0
0 0 2 0 1 0 1 0 0 1 1 2 1 0 1 0 2 0 2 2 1 2 2 0 1 2 0 2 2 1 0
5 1 1 1 0 1 1 0 2 1 1 1 2 1 1 0 1 2 2 2 2 2 2 0 5 0 0 2 2 2 1
2 1 1 2 1 1 1 1 5 1 2 5 1 1 2 5 5 0 1 0 2 0 0 0 2 0 0 1 0 1 2
0 0 0 1 1 1 2 2 1 2 2 0 0 2 0
TMB1425 0 0 0 1 2 0 2 0 0 0 0 0 5 0 1 2 0 1 1 1 0 0 1 1 1 1 0
0 0 2 2 0 0 1 0 1 1 1 0 1 0 1 0 2 0 2 2 1 1 1 0 0 1 0 2 0 1 0
1 1 1 1 0 1 1 2 0 1 1 0 2 1 1 0 1 2 2 5 2 2 1 0 5 0 0 2 2 2 1
2 1 1 1 1 1 1 0 1 1 2 0 2 1 1 0 0 0 1 0 0 0 0 0 0 0 0 1 0 0 2
0 1 0 1 2 1 1 1 1 0 1 0 0 2 0
BNL252 2 0 2 1 1 2 0 2 1 0 2 2 0 2 1 1 2 2 2 2 0 2 2 2 2 2 2 2

1 1 1 0 1 1 2 5 2 2 2 1 2 0 0 0 2 1 0 0 1 1 0 0 1 2 0 1 1 2 1
1 2 1 2 2 2 2 2 2 2 1 1 1 1 2 1 0 0 0 0 0 0 2 5 2 2 2 2 2 2 1
0 0 1 1 0 1 0 0 0 2 1 0 0 1 2 5 2 1 2 0 2 2 0 0 0 2 1 2 2 2 2
5 2 0 0 1 1 0 1 0 0 0 1 0 5
GH272 2 0 2 0 1 1 0 2 1 0 2 2 0 2 1 1 1 0 0 1 0 1 0 1 1 2 2 2
2 1 2 0 1 1 2 0 1 2 2 1 2 0 0 0 0 1 0 0 1 1 0 0 1 2 0 1 1 2 1
1 2 1 2 2 1 1 2 1 1 0 1 1 1 2 1 0 0 0 0 0 0 2 2 2 2 2 2 1 2 1
0 0 1 0 0 1 0 0 0 2 0 0 0 0 2 2 2 0 2 0 2 2 0 0 0 2 1 2 2 0 2
1 2 0 0 1 1 0 0 0 0 0 1 1 1
BNL2655 2 0 1 0 1 1 0 2 2 0 2 2 0 2 2 1 1 0 1 1 0 1 0 1 2 2 2
2 2 1 2 0 1 1 2 0 1 2 2 1 2 0 0 0 0 1 0 0 1 1 0 0 1 2 0 1 1 2
1 1 2 1 0 2 1 1 0 1 1 0 1 1 1 2 2 0 0 0 0 0 0 1 2 2 2 2 2 1 2
2 0 0 2 0 0 1 0 0 0 2 0 0 0 0 1 2 1 0 2 0 1 1 0 0 0 1 1 0 2 0
1 1 1 0 2 1 1 0 0 0 0 0 1 0 1
GH171 5 0 1 0 1 1 0 2 1 0 2 2 0 2 2 1 1 0 1 1 0 1 0 1 1 2 2 2
2 1 2 0 1 1 2 0 1 2 2 1 2 0 0 0 0 1 0 0 1 1 0 0 1 2 0 1 1 2 1
1 5 1 0 2 1 1 0 1 1 0 1 1 1 2 1 0 0 0 0 0 0 1 2 2 2 2 2 1 2 2
0 0 2 0 0 1 0 0 0 2 0 0 0 0 1 2 1 0 2 0 1 1 0 0 0 1 1 0 2 0 1
1 1 0 2 1 1 0 0 0 0 0 1 0 1
BNL2568 2 0 1 0 1 1 0 2 2 0 2 2 0 2 2 1 1 0 1 1 0 1 0 1 1 2 2
2 2 1 2 0 1 1 2 0 1 2 2 1 2 0 0 0 0 1 0 0 1 1 0 0 1 2 0 1 1 2
1 2 2 1 0 2 1 1 1 1 1 0 1 1 1 2 1 0 0 0 0 0 0 1 2 2 2 2 2 1 2
2 0 0 2 0 0 1 0 0 0 2 0 0 0 0 5 2 1 0 2 0 1 1 0 0 0 1 1 0 2 0
1 1 1 0 2 1 1 0 0 0 0 0 1 0 1
TMB0429 2 0 1 0 1 1 0 2 1 0 2 2 0 2 2 0 1 0 1 1 0 1 0 1 1 2 2
2 2 1 2 0 1 1 2 0 1 2 2 1 2 0 0 0 0 1 0 0 1 1 0 0 1 2 0 1 1 2
1 2 2 1 0 2 1 1 2 1 2 1 1 1 1 2 1 0 0 0 0 0 0 1 2 2 2 2 2 1 2
2 0 0 2 0 0 0 0 0 0 2 0 0 0 0 1 2 1 0 2 0 1 1 0 0 0 1 1 1 2 0
1 1 1 0 2 0 0 0 0 0 0 0 2 0 1
BNL2616 0 0 1 0 1 1 0 2 2 0 2 2 1 2 2 0 1 1 1 1 0 0 0 1 1 2 2
2 2 1 2 0 1 1 2 1 1 2 2 1 2 0 0 0 0 1 0 0 1 2 0 0 5 2 0 1 1 2
2 2 1 1 0 2 1 1 2 1 2 1 1 1 1 2 1 0 0 0 0 0 0 1 2 2 1 2 2 2 2
0 0 0 2 0 0 0 0 0 0 2 1 0 0 0 1 1 1 0 2 0 1 1 0 0 0 1 0 1 0 1
1 1 1 0 2 1 0 0 1 0 0 0 1 1 1
BNL1521 0 0 0 0 1 1 0 2 1 0 2 2 1 1 2 0 1 1 1 1 0 0 0 1 1 2 2
2 0 1 2 0 1 1 2 1 1 2 2 0 2 0 0 0 0 1 0 0 1 2 0 0 2 1 0 1 1 2
2 2 1 1 0 1 1 1 2 1 2 1 1 1 1 2 1 0 0 0 0 0 0 1 2 1 1 2 2 2 2
2 1 1 1 0 0 0 0 0 0 2 1 0 0 0 1 1 1 0 2 0 1 1 0 0 0 1 0 1 0 1
1 1 1 0 2 1 0 0 1 0 0 0 2 0 1
GH224 2 2 0 0 0 1 2 1 0 0 2 2 1 1 0 0 2 0 1 0 0 1 0 0 1 1 1 1
2 0 0 2 0 0 2 0 2 2 2 0 2 0 2 2 1 0 2 0 0 0 0 2 0 2 2 1 2 0 0
1 1 0 0 1 0 0 0 1 2 1 1 1 1 1 1 2 1 1 0 1 1 0 0 0 0 0 2 1 0 2
2 2 1 1 1 0 2 2 0 1 1 1 2 1 0 5 0 1 0 2 0 0 2 1 1 0 1 5 0 5 0
1 0 1 2 1 1 2 1 1 2 0 0 1 5
JESPR215 2 2 0 0 0 1 2 1 1 0 2 2 0 1 0 0 2 0 1 0 0 1 0 0 1 0 1
1 2 0 0 2 0 0 2 0 2 2 2 0 2 0 2 2 1 0 2 0 0 0 0 2 0 2 2 1 2 0
0 5 1 0 0 1 0 0 0 1 2 1 1 1 1 1 1 2 1 1 0 1 1 0 0 0 0 0 2 1 0
2 2 0 1 1 1 0 2 2 0 1 1 1 2 2 0 0 0 1 0 2 0 0 2 1 1 0 1 0 0 0
0 1 0 1 2 1 1 2 1 1 2 0 0 1 1
JESPR227 2 2 0 0 0 1 2 5 1 5 2 2 0 1 0 0 2 0 1 0 0 1 0 0 1 0 1
1 2 0 0 2 0 0 2 0 2 2 2 0 2 0 2 2 1 0 2 0 0 0 0 2 0 2 2 1 2 5
0 5 1 0 0 1 0 0 0 1 2 1 1 1 1 1 1 2 1 1 0 1 1 0 0 0 0 1 2 1 0
2 2 2 1 1 1 0 2 5 0 1 1 1 2 2 2 0 0 1 0 5 5 0 5 1 1 0 5 0 5 5
0 1 0 1 2 1 1 2 1 1 2 0 0 1 1
CM27 2 2 0 0 0 1 2 5 1 0 2 2 0 1 0 0 2 0 1 0 0 1 0 0 1 0 1 1 2
0 0 2 0 0 2 0 2 2 2 0 2 0 1 2 1 0 1 0 0 0 0 2 0 2 2 1 2 0 0 5
1 0 0 1 0 0 0 1 2 1 1 1 1 1 1 2 1 1 0 1 1 0 0 0 0 0 2 1 0 2 2

2 1 1 1 0 2 2 0 1 1 1 2 2 0 0 0 0 0 2 0 0 2 1 1 0 5 0 0 0 0 1
0 1 2 1 1 2 0 1 2 0 0 1 1
CM13 2 2 0 0 0 1 2 1 1 1 2 2 0 1 0 0 2 0 1 0 0 1 0 0 1 0 1 1 2
0 0 2 0 0 2 0 2 2 2 0 2 0 2 2 1 0 2 0 0 0 0 2 0 2 2 1 2 1 0 1
1 0 0 1 0 0 0 1 2 1 1 1 1 1 1 2 1 1 0 1 1 0 0 0 0 0 2 1 0 2 2
2 1 1 1 0 2 2 0 1 1 1 2 2 1 0 1 0 0 2 0 0 2 1 1 0 1 0 0 0 0 1
0 1 2 0 0 2 0 1 2 0 0 1 1
NAU2750 1 2 2 2 2 1 0 1 1 2 2 0 2 1 2 2 0 0 0 0 2 0 0 0 0 0 2
0 1 2 2 2 2 2 2 1 1 2 0 2 0 0 0 2 0 2 0 0 2 0 2 0 2 2 0 1 1 0
0 5 1 1 0 2 0 0 0 0 0 0 2 0 0 0 2 0 0 0 0 0 0 0 2 2 0 1 0 1 0
0 0 0 2 0 0 2 0 0 2 1 0 2 1 2 0 0 0 0 1 0 0 0 0 0 0 0 1 0 0 2
0 2 0 2 2 0 0 0 0 0 0 1 2 2 1
BNL341 1 1 2 2 2 1 0 1 2 2 2 0 2 1 2 2 1 0 1 2 1 1 1 1 1 1 2 1
1 2 2 2 2 2 2 1 1 2 0 2 2 0 0 2 1 2 0 0 1 0 2 0 2 2 0 1 1 0 0
1 1 1 0 2 1 1 0 0 0 0 2 0 0 1 2 0 0 0 0 0 0 0 0 0 0 1 1 5 0 0
0 0 2 0 0 2 0 0 2 1 0 2 1 5 0 0 0 0 1 0 0 0 0 0 0 0 1 0 0 2 0
2 0 0 2 0 0 0 0 0 0 1 2 0 0
NAU2913 1 1 2 2 2 1 0 2 2 2 2 2 0 2 1 2 2 2 0 1 2 2 1 2 1 2 2 2
1 1 2 0 0 0 1 0 1 1 2 0 2 2 0 1 2 1 2 0 1 1 0 2 0 2 2 0 1 2 0
0 1 1 1 0 2 1 1 0 0 0 0 2 0 0 1 2 0 0 0 0 0 0 0 0 0 0 1 1 1 0
0 0 2 2 0 0 2 0 0 2 1 0 1 2 2 0 0 0 0 2 0 0 0 0 2 2 2 1 0 0 2
0 2 0 0 2 0 2 0 2 0 0 1 2 0 1
GH200 1 0 0 0 1 1 0 1 1 1 2 2 1 1 1 1 0 2 2 2 5 1 5 2 5 5 5 1
2 2 1 1 1 0 2 0 1 1 1 1 1 0 1 0 1 1 0 1 2 2 0 1 1 0 0 0 0 0 0
0 0 0 0 0 2 2 0 1 2 0 0 0 0 0 0 2 2 1 1 2 2 0 2 0 0 0 0 0 0 2
0 0 0 0 0 1 0 0 1 0 0 2 2 1 0 0 0 0 2 2 0 0 0 2 1 0 0 0 2 0 0
0 0 2 2 0 0 1 0 1 1 1 1 0 1
TMB0120 1 0 0 0 0 0 1 1 1 1 0 1 1 0 1 2 0 2 2 2 0 1 0 2 2 0 0
1 2 2 0 2 1 0 1 0 0 2 1 2 1 0 1 0 1 2 1 1 1 1 1 1 1 0 1 0 0 0
0 0 0 0 0 0 2 2 0 1 2 0 0 0 0 0 0 2 2 1 2 2 2 0 2 0 0 0 0 0 0
2 0 0 0 1 0 1 0 0 1 0 0 2 2 1 0 0 0 0 2 2 0 0 0 2 1 0 0 0 2 0
0 1 0 1 2 0 0 2 0 2 2 0 2 0 2
GH52 1 0 0 0 0 0 1 1 0 1 0 1 1 0 1 2 0 2 2 2 0 1 0 2 2 0 0 1 2
2 0 2 1 0 1 0 0 2 1 2 1 0 1 0 1 2 1 1 1 1 1 1 1 0 1 0 0 0 0 0
0 0 0 0 2 2 0 1 2 0 0 0 0 0 0 2 2 1 2 2 2 0 2 0 0 0 0 0 0 2 0
0 0 1 0 1 0 1 1 0 0 2 2 1 0 0 0 0 2 2 0 0 0 2 1 0 0 0 2 0 0 1
0 1 2 0 0 2 0 2 2 0 2 0 2
BNL3994 1 0 0 0 0 0 1 1 1 0 0 1 1 0 1 1 0 2 2 2 0 1 0 2 2 0 0
1 2 2 0 2 2 0 1 0 0 2 2 2 1 0 1 0 1 2 1 1 1 1 1 1 1 0 1 0 0 0
0 5 0 0 0 0 2 2 0 1 2 0 0 0 0 0 0 2 2 1 2 2 2 0 2 0 0 0 0 0 0
2 0 0 0 1 0 1 0 1 1 0 0 2 2 1 0 0 0 0 2 2 0 0 0 2 1 0 0 0 2 0
0 1 0 1 2 0 0 2 0 2 2 0 2 0 2
NAU1119 2 1 1 1 1 0 1 2 2 0 0 0 1 0 2 0 0 0 0 0 1 0 0 0 0 0 0
0 2 1 0 2 1 1 0 1 2 2 1 1 0 0 0 0 0 1 1 2 0 0 0 2 0 1 1 0 0 0
0 5 1 1 0 0 0 0 0 0 0 0 1 0 0 1 0 0 1 1 1 0 2 0 1 1 0 0 0 0 0
1 1 1 1 0 0 1 0 0 2 0 0 0 0 1 0 0 0 0 0 1 0 0 0 0 0 0 1 0 0 2
0 2 0 0 0 0 0 0 0 1 0 0 1 1 0
JESPR92 2 1 1 1 0 1 2 1 1 5 1 0 1 1 5 1 1 0 1 1 1 1 1 1 1 2 0
1 2 1 0 2 1 0 0 0 2 2 1 1 2 0 0 0 1 0 1 2 2 0 0 2 0 0 1 0 0 0
0 0 0 0 0 0 1 1 0 0 0 1 0 0 0 0 0 0 1 1 1 0 2 0 0 1 0 0 0 0 0
1 1 1 0 1 0 1 0 0 2 0 1 0 0 1 0 0 0 0 0 1 0 0 1 0 0 0 1 0 5 2
0 2 0 0 0 1 0 1 0 2 0 0 1 1 1
BNL3816 2 1 1 1 0 1 2 1 1 0 1 0 1 2 2 1 1 0 1 2 1 1 1 1 1 2 0
1 0 1 0 2 1 0 0 0 2 2 1 1 2 0 0 0 1 0 1 2 2 0 0 2 0 0 1 0 0 0
0 0 0 0 0 0 1 1 0 0 0 1 0 0 0 0 0 0 1 1 1 0 2 0 0 1 0 0 0 0 0
1 1 1 0 1 0 1 0 0 2 0 1 0 0 1 0 0 0 0 0 1 0 0 1 0 0 0 1 0 0 2
0 2 0 0 0 1 0 1 0 2 0 0 1 1 1

NAU3006 2 1 1 1 0 1 2 1 1 0 1 0 1 2 2 1 1 0 1 2 1 1 1 1 1 2 0
1 2 1 0 2 1 0 0 0 2 2 1 1 2 0 0 0 1 0 1 2 2 0 0 2 0 0 1 0 0 0
0 0 0 0 0 0 1 1 0 0 0 1 0 0 0 0 0 0 1 1 1 0 2 0 0 1 0 0 0 0 0
1 1 1 0 1 0 1 0 0 2 0 1 0 0 1 0 0 0 0 0 1 0 0 1 0 0 0 1 0 0 2
0 2 0 0 0 1 0 1 0 2 0 0 1 1 1
BNL840 2 1 1 1 0 1 2 1 1 0 1 0 1 2 2 1 1 0 1 2 1 1 1 1 1 2 0 1
2 1 0 2 1 0 0 0 2 2 1 1 2 0 0 0 1 0 1 2 2 0 0 2 0 0 1 0 0 0 0
0 0 0 0 0 1 1 0 0 0 1 0 0 0 0 0 0 1 1 1 0 2 0 0 1 0 0 0 0 0 1
1 1 0 1 0 1 0 0 2 0 1 0 0 1 0 0 0 0 0 1 0 0 1 0 0 0 1 0 0 2 0
2 0 0 0 1 0 1 0 2 0 0 1 1 1
CIR391 2 1 1 1 0 1 2 1 1 0 1 0 1 2 2 1 1 0 1 2 1 1 1 1 1 2 0 1
2 1 0 2 1 0 0 0 2 2 1 1 2 0 0 0 1 0 1 2 2 0 0 2 0 0 1 0 0 0 0
0 0 0 0 0 1 1 0 0 0 1 0 0 0 0 0 0 1 1 1 0 2 0 0 1 0 0 0 0 0 1
1 1 0 1 0 1 0 0 2 0 1 0 0 1 0 0 0 0 0 1 0 0 1 0 0 0 1 0 0 2 0
2 0 0 0 1 0 1 0 2 0 0 1 1 1
CIR039 2 1 1 1 0 1 2 1 1 0 1 0 1 2 2 1 1 0 1 2 1 1 1 1 1 2 0 1
2 1 0 2 1 0 0 0 2 2 1 1 2 0 0 0 0 0 1 2 2 0 0 2 0 0 1 0 0 0 0
0 0 0 0 0 1 1 0 0 0 1 0 0 0 0 0 0 1 1 1 0 2 0 0 1 0 0 0 0 0 1
1 1 0 1 0 1 0 0 2 0 1 0 0 1 0 0 0 0 0 1 0 0 1 0 0 0 1 0 0 2 0
2 0 0 0 1 0 1 0 2 0 0 1 1 1
BNL3510 2 1 1 1 0 1 2 1 1 0 1 0 2 2 2 1 1 0 1 2 1 1 5 1 1 2 0
1 2 1 0 2 1 0 0 0 2 2 1 1 2 0 0 0 1 0 1 2 2 0 0 2 0 0 1 0 0 0
0 0 0 0 0 0 1 1 0 0 0 1 0 0 0 0 0 0 1 1 1 0 2 0 0 0 0 0 0 0 0
1 1 1 0 1 0 1 0 0 2 0 1 0 0 1 0 0 0 0 0 1 0 0 1 0 0 0 1 0 0 2
0 2 0 0 0 1 0 1 0 2 0 0 1 1 1
NAU2195 2 1 1 1 0 1 2 1 1 0 1 0 0 2 2 1 1 0 1 2 1 1 0 1 1 2 0
1 2 1 0 2 1 0 0 0 2 2 1 1 2 0 0 0 1 0 1 2 2 0 0 2 0 0 1 0 0 2
2 2 2 0 0 0 1 1 2 0 0 1 0 0 0 0 0 0 1 1 1 0 2 0 0 0 0 0 0 0 0
1 1 1 0 1 0 1 0 0 2 0 1 0 0 1 0 0 0 0 0 1 0 0 1 0 0 0 1 0 0 2
0 2 0 0 0 1 0 1 0 2 0 0 1 1 1
-stop markers
-start traits
Flowering_time 68.0 79.0 80.0 55.0 56.0 56.0 84.0 67.0 70.0
73.0 58.0 53.0 55.0 61.0 79.0 63.0 71.0 65.0 58.0 0.0 79.0
65.0 77.0 68.0 70.0 71.0 59.0 64.0 85.0 49.0 67.0 88.0 70.0
84.0 73.0 69.0 65.0 56.0 67.0 63.0 65.0 56.0 60.0 71.0 59.0
61.0 55.0 53.0 55.0 59.0 55.0 70.0 59.0 58.0 58.0 55.0 48.0
59.0 65.0 50.0 50.0 48.0 47.0 51.0 59.0 53.0 47.0 63.0 61.0
52.0 55.0 58.0 43.0 48.0 56.0 65.0 75.0 58.0 0.0 80.0 81.0
59.0 73.0 52.0 79.0 72.0 70.0 64.0 56.0 65.0 60.0 54.0 49.0
53.0 65.0 56.0 63.0 63.0 60.0 57.0 53.0 68.0 51.0 81.0 49.0
65.0 52.0 44.0 69.0 65.0 59.0 55.0 81.0 65.0 67.0 58.0 55.0
60.0 63.0 64.0 65.0 67.0 57.0 80.0 65.0 57.0 60.0 74.0 51.0
0.0 0.0 58.0 61.0 68.0 63.0
Buds_number 19.0 1.0 2.0 69.0 114.0 57.0 4.0 33.0 24.0 36.0
41.0 62.0 68.0 24.0 2.0 94.0 33.0 56.0 46.0 0.0 2.0 26.0 12.0
25.0 16.0 11.0 22.0 22.0 5.0 31.0 6.0 1.0 16.0 2.0 10.0 10.0
9.0 25.0 40.0 76.0 47.0 45.0 17.0 11.0 98.0 54.0 32.0 37.0
81.0 51.0 86.0 22.0 55.0 21.0 63.0 33.0 88.0 62.0 25.0 55.0
35.0 50.0 75.0 30.0 45.0 43.0 84.0 63.0 30.0 93.0 17.0 75.0
84.0 30.0 38.0 20.0 12.0 39.0 0.0 6.0 6.0 28.0 11.0 37.0 7.0
20.0 18.0 44.0 31.0 19.0 45.0 49.0 41.0 45.0 28.0 58.0 16.0
34.0 35.0 30.0 80.0 17.0 58.0 8.0 85.0 37.0 28.0 29.0 11.0
19.0 28.0 103.0 3.0 46.0 12.0 81.0 31.0 37.0 19.0 14.0 28.0
18.0 37.0 4.0 6.0 11.0 15.0 4.0 20.0 0.0 0.0 20.0 32.0 21.0
30.0
Flowering_frame 26.0 0.0 4.0 39.0 38.0 37.0 8.0 27.0 24.0 21.0

```
36.0 40.0 38.0 30.0 14.0 30.0 22.0 28.0 36.0 0.0 1.0 29.0 17.0
25.0 24.0 23.0 34.0 30.0 6.0 45.0 24.0 0.0 24.0 3.0 18.0 18.0
26.0 38.0 27.0 31.0 29.0 38.0 32.0 22.0 35.0 32.0 28.0 36.0
39.0 35.0 39.0 24.0 35.0 36.0 36.0 38.0 45.0 35.0 28.0 44.0
42.0 44.0 42.0 36.0 25.0 40.0 47.0 31.0 33.0 42.0 34.0 36.0
48.0 43.0 36.0 29.0 18.0 36.0 0.0 14.0 12.0 34.0 18.0 41.0
15.0 22.0 24.0 29.0 38.0 28.0 34.0 40.0 40.0 34.0 26.0 38.0
28.0 31.0 34.0 34.0 41.0 25.0 43.0 13.0 40.0 29.0 32.0 28.0
21.0 29.0 35.0 39.0 12.0 29.0 27.0 36.0 39.0 32.0 26.0 30.0
25.0 27.0 33.0 8.0 23.0 24.0 25.0 20.0 34.0 0.0 0.0 33.0 33.0
26.0 31.0
Photop_flowering 3.0 4.0 4.0 2.0 2.0 2.0 4.0 3.0 3.0 3.0 2.0
1.0 2.0 2.0 4.0 2.0 3.0 3.0 2.0 6.0 4.0 3.0 4.0 3.0 3.0 3.0
2.0 2.0 5.0 1.0 3.0 5.0 3.0 4.0 3.0 3.0 3.0 2.0 3.0 2.0 3.0
2.0 2.0 3.0 2.0 2.0 2.0 1.0 2.0 2.0 2.0 3.0 2.0 2.0 2.0 2.0
1.0 2.0 3.0 1.0 1.0 1.0 1.0 1.0 2.0 1.0 1.0 2.0 2.0 1.0 2.0
2.0 1.0 1.0 2.0 3.0 4.0 2.0 6.0 4.0 4.0 2.0 3.0 1.0 4.0 3.0
3.0 2.0 2.0 3.0 2.0 2.0 1.0 1.0 3.0 2.0 2.0 2.0 2.0 2.0 1.0
3.0 1.0 4.0 1.0 3.0 1.0 1.0 3.0 3.0 2.0 2.0 4.0 3.0 3.0 2.0
2.0 2.0 2.0 2.0 3.0 3.0 2.0 4.0 3.0 2.0 2.0 3.0 1.0 6.0 6.0
2.0 2.0 3.0 2.0
-stop traits
-quit
-end
```
