# Supplementary material for: QTL mapping for flowering-time and photoperiod insensitivity of cotton Gossypium darwinii Watt
Source: PLoS One. 2017 Oct 9;12(10):e0186240. doi: 10.1371/journal.pone.0186240 (PMC5633191; doi:10.1371/journal.pone.0186240)
Supplement: S1 Table — (DOC) [file pone.0186240.s007.doc]

**Table S1.** Genetic variances and estimated broad-sense heritability of traits in the F_2_ population

|  | NMB | NSB | PhFl | NOBL | SA | SH | PH |
| --- | --- | --- | --- | --- | --- | --- | --- |
| P_1_ | 2,55 | 2,17 | 0,00 | 0,00 | 0,00 | 0,00 | 8,37 |
| P_2_ | 1,07 | 2,27 | 0,49 | 2,69 | 0,53 | 0,38 | 8,16 |
| F_2_ | 3,77 | 8,31 | 2,55 | 1,43 | 0,70 | 0,87 | 38,25 |
| V_g_ | 0,68 | 5,01 | 2,31 | 0,08 | 0,44 | 0,68 | 25,80 |
| H^2^ | 0,18 | 0,60 | 0,90 | 0,06 | 0,62 | 0,78 | 0,67 |

NMB - number of monopodial branches; NSB - number of sympodial branches; NOBL - number of opened bolls; SA - stem anthocyanin; SH - stem hairiness; PH - plant height; P_1_ - wild type pre-mutagenesis parent (*G. drawinii*); P_2_ - irradiation mutant parent (*G. drawinii)*; F_2_ – second generation population; V_g_ - genotypic effect; and H^2^ – broad-sense heritability
