# Supplementary material for: QTL mapping for flowering-time and photoperiod insensitivity of cotton Gossypium darwinii Watt
Source: PLoS One. 2017 Oct 9;12(10):e0186240. doi: 10.1371/journal.pone.0186240 (PMC5633191; doi:10.1371/journal.pone.0186240)
Supplement: S2 Table — (DOC) [file pone.0186240.s008.doc]

**Table S2. Genomic distributions of SSR markers and identified QTLs for several morphological traits in the F_2_** mapping population of this study

| **#** | **Pop.** | **QTL** | **LGs (Chr)** | **Linked markers** | **Position (cM)** | **LOD** | **Add. effect** | **Dom. effect** | **Literature reports** | |
| --- | --- | --- | --- | --- | --- | --- | --- | --- | --- | --- |
|  |  |  |  |  |  |  |  |  | **Various QTLs and their chromosomal locations** | **Reference** |
| 1 | F_2_ | *qPH* | LG01 (Chr.01) | TMB0062_280-BNL3888_180 | 16.35-27.82 | 2.53 | 14.65 | 3.70 | Fiber length (Chr.01) | Shen et al. [48] |
| 2 | F_2_ | *qSA* | LG07 (Chr.09) | GH98_128-GH112_160 | 0.00-2.00 | 2.7 | 0.2 | 0.3 | - | - |
| 3 | F_2_ | *qSA* | LG08 (Chr.09) | BNL1414_135-BNL4028_140 | 17.20-22.10 | 3.31 | 0.2 | 0.23 | Fiber traits (Chr.23) | Wang et al. [69] |
| 4 | F_2_ | *qSH* | LG11 (Chr.12) | NAU5047_230-CIR148_155 | 7.20-17.40 | 3.2 | 0.2 | 0.4 | Fiber elongation (Chr.12) | Zhang et al. 54] |
| 5 | F_2_ | *qNOBL* | LG12 (Chr.13) | BNL1495_200-BNL3623_240 | 0.00-7.80 | 2.93 | 0.48 | 0.29 | Lint cotton yield ((Chr.13) | Wu et al. [50] |
| 6 | F_2_ | *qPH* | LG19 (Chr.20) | GH54_160-BNL3948_95 | 15.70-19.30 | 2.55 | 9.71 | 8.22 | Fiber length uniformity (Chr.20) | Wang et al. [70] |
| 7 | F_2_ | *qNSB* | LG19 (Chr.20) | BNL169_210-GH48_96 | 1.90-2.80 | 3.28 | 2.79 | 2.06 | - | - |
| 8 | F_2_ | *qPH* | LG20 (Chr.21) | CM23_115-TMB2038_125 | 14.40-30.20 | 5.85 | 14.62 | 12.57 | Verticillium wilt resistance | Bolek et al. [52] |
| 9 | F_2_ | *qNSB* | LG20 (Chr.21) | CM23_115-TMB2038_125 | 14.40-30.20 | 3.53 | -1.33 | 4.49 | Verticillium wilt resistance | Bolek et al. [52] |
| 10 | F_2_ | *qSA* | LG21 (Chr.23) | TMB1701_180-BNL686_145 | 46.60-75.40 | 2.76 | 0.5 | 0.4 | Fiber micronaire (Chr.23) | Li et al. [71] |
| 11 | F_2_ | *qNOBL* | LG22 (Chr.24) | BNL2568_150-GH171_285 | 11.50-13.70 | 2.72 | 0.07 | 0.73 | - | - |
| 12 | F_2_ | *qNMB* | LG24 (Chr.26) | BNL840_160-NAU3006_220 | 39.10-40.00 | 3.25 | 1.17 | 2.43 | - | - |
| 13 | F_2_ | *qNSB* | LG24 (Chr.26) | BNL840_160-NAU3006_220 | 39.10-40.00 | 3.7 | 1.61 | 5.56 | - | - |
| 14 | F_2_ | *qNSB* | LG24 (Chr.26) | NAU3006_220-NAU2913_245 | 40.00-70.30 | 4.42 | 4.17 | 9.06 | - | - |

Additional references:

1. Wang F, Gong Y, Zhang C, Liu G, Wang L, Xu Z, Zhang J. Genetic effects of introgression genomic components from Sea Island cotton (*Gossypium* *barbadense* L.) on fiber related traits in Upland cotton (*G. hirsutum* L.). Euphytica. 2011; 181: 41–53.
2. Wang H, Huang C, Guo H, Li X, ZhaoW, Dai B, et al. QTL mapping for fiber and yield traits in Upland cotton under multiple environments. PLoS ONE. 2015; 10: 1-14. e0130742. doi: 10.1371/journal. pone.0130742 PMID 26110526
3. Li X, Yuan D, Zhang J, Lin Z, Zhang X. Genetic mapping and characteristics of genes specifically or preferentially expressed during fiber development in cotton. PLoS One. 2013; 8: 1-9. doi: 10.1371/journal.pone.0054444 PMID: 23372723
